# *Cis/Trans* Energetics in Epoxide, Thiirane, Aziridine and Phosphirane Containing Cyclopentanols: Effects of Intramolecular OH⋯O, S, N and P Contacts

**DOI:** 10.3390/molecules24142523

**Published:** 2019-07-10

**Authors:** Ben E. Smith, Jeremy M. Carr, Gregory S. Tschumper

**Affiliations:** 1 Department of Chemistry and Biochemistry, University of Mississippi, 322 Coulter Hall, University, MS 38677-1848, USA; 2 Chemistry Department, Central Alabama Community College, 1675 Cherokee Road, Alexander City, AL 35010, USA

**Keywords:** intramolecular hydrogen bonding, semipinacol rearrangement, vibrational frequencies, density functional theory (DFT), coupled-cluster theory

## Abstract

A recent computational analysis of the stabilizing intramolecular OH⋯O contact in 1,2-dialkyl-2,3-epoxycyclopentanol diastereomers has been extended to thiiriane, aziridine and phosphirane analogues. Density functional theory (DFT), second-order Møller-Plesset perturbation theory (MP2) and CCSD(T) coupled-cluster computations with simple methyl and ethyl substituents indicate that electronic energies of the cis isomers are lowered by roughly 3 to 4 kcal mol^−1^ when the OH group of these cyclopentanol systems forms an intramolecular contact with the O, S, N or P atom on the adjacent carbon. The results also suggest that S and P can participate in these stabilizing intramolecular interactions as effectively as O and N in constrained molecular environments. The stabilizing intramolecular OH⋯O, OH⋯S, OH⋯N and OH⋯P contacts also increase the covalent OH bond length and significantly decrease the OH stretching vibrational frequency in every system with shifts typically on the order of −41 cm^−1^.

## 1. Introduction

**Figure 1:**
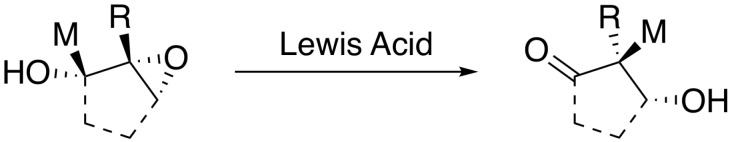
Lewis Acid-mediated Type III semipinacol rearrangement reaction converts 2,3-epoxyalcohols into the corresponding 1,3-ketol with concurrent 1,2-M group migration

Previously known as the epoxy alcohol-aldol rearrangement [1], the Type III semipinacol rearrangement reaction [2,3] is the Lewis acid-mediated conversion of 2,3-epoxyalcohols to the corresponding β-hydroxycarbonyl (Figure 1). This conversion, which can be accomplished with a wide range of Lewis acids [4,5,6,7,8,9], is often accompanied by 1,2-alkyl migration, a transformation that is historically regarded as a convenient route to accessing chiral quaternary centers. Apart from its presence in numerous synthetic methodologies, the reaction’s appeal is evident in industrial and commercial applications [10] as well as in the preparation of a variety of natural products [11,12,13,14]. Despite its utility, however, the mechanistic details of the semipinacol rearrangement are poorly understood. Chemists generally agree that an antiperiplanar arrangement between the migrating group (M in Figure 1) and the adjacent, epoxide CO bond is necessary to drive the reaction [10,15], an arrangement that explains why *trans* diastereomers fail to react [7,16]. However, additional, proposed mechanistic details for Type III semipinacol rearrangements [17,18,19,20] are not supported by experimental or theoretical evidence. This apparent void sparked an initial interest in investigating why 2,3-epoxyalcohols rearrange to the corresponding ketols.

**Figure 2:**
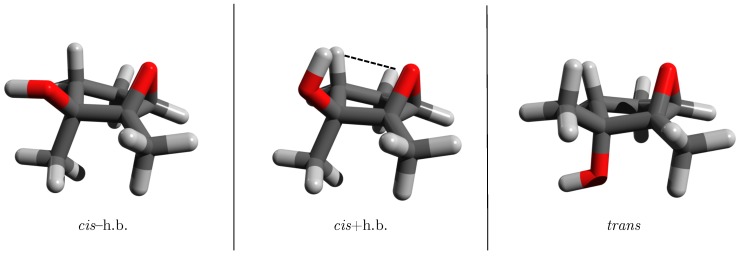
Three structural motifs of the 1,2-dimethyl-2,3-epoxycyclopentanols examined in Reference [21]: *cis* isomer without hydrogen bond (**left**), *cis* isomer with hydrogen bond (**center**), *trans* isomer (**right**).

**Figure 3:**
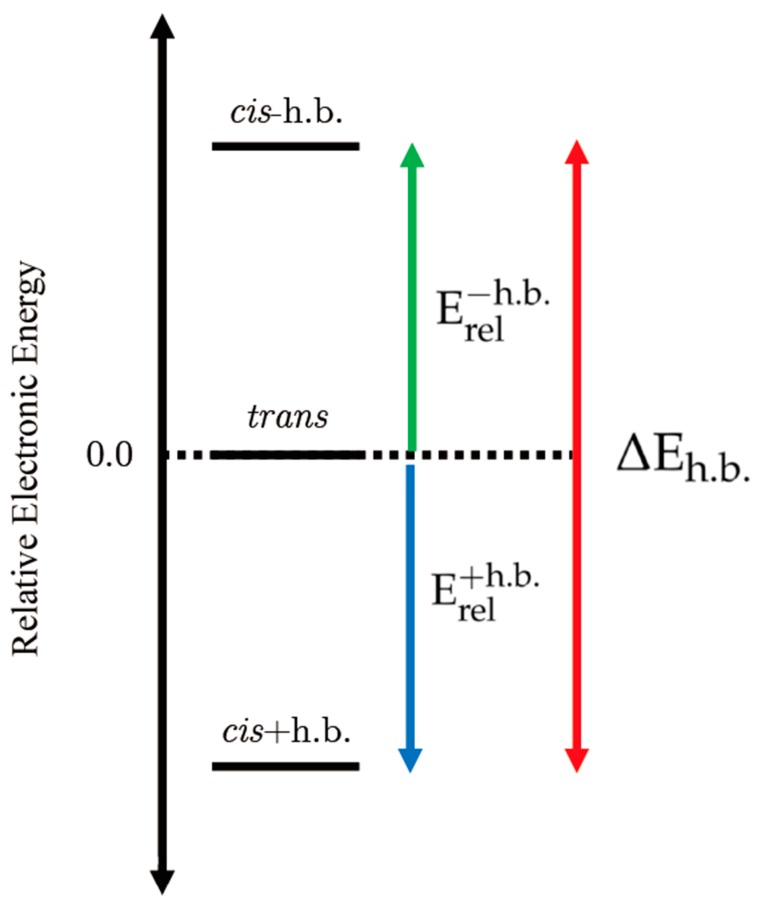
A schematic of the relative energies of the three different structural motifs: *cis* isomer without hydrogen bond, *cis* isomer with hydrogen bond, *trans* isomer.

To gain insight into these reactions, we recently examined the *cis/trans* energy differences in simple 1,2-dialkyl-2,3-epoxycyclopentanols [21]. Our quantum mechanical electronic structure computations from that study indicated that the *trans* diastereomers (right panel in Figure 2) had lower electronic energies than the corresponding *cis* structures in which the OH group pointed away from the epoxide (left panel in Figure 2). The corresponding relative energy is denoted $E_{\mathrm{rel}}^{-h.b.}$ and depicted schematically in the top half of Figure 3. However, when the OH group was rotated toward the epoxide O atom in the *cis* diastereomers (center panel in Figure 2), the substrate exhibited significant stabilization, yielding electronic energies lower than those of the *trans* structures. This relative energy is labeled $E_{\mathrm{rel}}^{+h.b.}$ and illustrated in the bottom half of Figure 3. To our knowledge, the OH⋯O interaction identified in that study was the first instance of a stabilizing intramolecular contact between epoxide and alcohol moieties to be reported. Furthermore, those findings offered compelling evidence indicating that intramolecular proton transfer may be involved in the mechanism responsible for converting the 1,2-dialkyl-2,3-epoxyalcohol to the corresponding 2,2-dialkyl-1,3-ketol.

**Figure 4:**
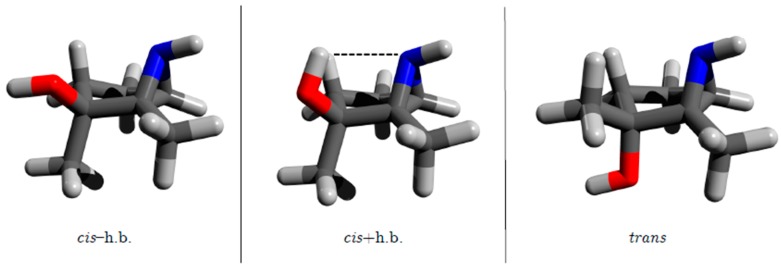
Three structural motifs of the 1,2-dimethyl-2,3-aziridine cyclopentanols: *cis* isomer without hydrogen bond (**left**), *cis* isomer with hydrogen bond (**center**), *trans* isomer (**right**).

The present study seeks to further characterize this intramolecular contact and any potential effects on the *cis/trans* energy differences of the aziridine, phosphirane and thiirane analogs of 1,2-dialkyl-2,3-epoxycyclopentanol. Specifically, our goal is to quantify any relative energy changes resulting from an intramolecular OH⋯N, OH⋯P or OH⋯S contact because N, P and S atoms can also potentially act as hydrogen bond acceptors. An extensive literature search revealed some stabilization in molecules containing alcohol and aziridine functional groups through an OH⋯N contact [22,23,24,25,26,27]. Because this interaction was similar to the OH⋯O contact reported in Reference [21], we anticipated that 1,2-dialkyl-2,3-aziridinylcyclopentanol would also adopt three comparable arrangements as shown in Figure 4. No instances of corresponding intramolecular OH⋯P or OH⋯S stabilizing contacts for phosphirane- and thiirane-containing systems have been reported, but the capacity of P and S atoms to accept intramolecular hydrogen bonds is well established both experimentally and theoretically [25,28,29,30,31,32,33].

This investigation also provides some important theoretical extensions to the analysis presented in Reference [21]. In this work, the electronic structure computations are expanded to probe the effects of adding diffuse functions to the atomic orbital Gaussian basis sets. More importantly, quantification of these intramolecular contacts is extended beyond a simple relative energy scheme, which can overestimate the strength of the interaction [34], in contrast to intermolecular hydrogen bonds where direct computation of the hydrogen bond strength is relatively straightforward [35,36,37,38]. Thus, this investigation also reports physical characteristics, such as changes in OH stretching frequencies and other metrics widely considered among chemists to be distinctive features of hydrogen bonding.

In this paper, the term “hydrogen bond” is utilized to describe all reported OH⋯A contacts, where A = O, S, N and P. This semantic decision is predicated on the well-established role that intramolecular hydrogen bonding plays in conformer stabilization [39,40,41,42,43,44,45,46,47]. Although the “qualifying features of a hydrogen bond are contentious” [48], particularly in constrained intramolecular environments, such as between functional groups located on adjacent carbon atoms, there is strong experimental and theoretical evidence supporting intramolecular hydrogen bonding in instances that do not fit the formal definition [34,48,49,50]. Ultimately, this designation has no impact on the results presented in this manuscript. It merely simplifies the discussion of the relative energetics of the various *cis* and *trans* structures.

## 2. Computational Methods

To probe the ability of the thiirane, aziridine and phosphirane groups to accept an intramolecular hydrogen bond in the same manner as the epoxide group in the 1,2-dialkyl-2,3-epoxycyclopentanol systems, this work employs methyl (Me) and ethyl (Et) alky substituents in three different substitution patterns at the M/R positions (Figure 1): Me/Me, Me/Et and Et/Me. For these twelve different systems, the following three distinct configurations are examined: *trans* configuration; *cis* configuration with the intramolecular hydrogen bond (*cis*+h.b.); *cis* configuration without the intramolecular hydrogen bond (*cis*−h.b.). These variations give a total of 36 unique structures examined in this study, 6 of which are shown in Figure 2 and Figure 4.

Full geometry optimizations were performed on all 36 structures with the M06-2X [51] global hybrid density functional theory (DFT) method and two sets of correlation consistent triple zeta basis sets, one without and one with diffuse functions on all atoms (cc-pVTZ [52] and aug-cc-pVTZ [53,54] simply denoted TZ and aTZ, respectively, hereafter). M06-2X harmonic vibrational frequencies were also computed with the TZ and aTZ basis sets for every optimized structure to ensure they are minima with no imaginary frequencies ($n_{i}=0$). For systems containing S and P, M06-2X computations were also performed using the cc-pV(T+*d*)Z basis set [55] for those centers and the TZ basis set for all other atoms, but those results have been relegated to the Supplementary Material because they are virtually identical to the M06-2X/TZ data discussed in detail in the next section. The nuclear magnetic resonance (NMR) chemical shielding constants were also calculated [56] at the M06-2X/TZ level of theory using the gauge-independent atomic orbital (GIAO) method [57].

Although prior studies [58,59] have shown the M06-2X functional can provide reliable conformational energetics for systems exhibiting intramolecular hydrogen bonding, MP2 [60] geometry optimizations and harmonic vibrational frequency computations were also carried out in this work with the same TZ basis set to provide an additional estimate of the energetics associated with these inter and intramolecular interactions. Using the MP2/TZ geometries, a subsequent set of single point energy computations were carried out with the CCSD(T) coupled-cluster method that includes all single and double substitutions along with a perturbative estimate of connected triple excitations.

To examine the intrinsic energetics of these intramolecular contacts, all computations were carried out on the isolated molecular species. The M06-2X and MP2 computations were performed with the Gaussian 09 software package [61]. All structures were optimized without constraints, and the M06-2X computations employed the default numerical integration grid in Gaussian 09. The residual Cartesian forces of the optimized structures did not exceed $2.0\times 10^{-5}$ $E_{h}$ a.u.^−1^. The CCSD(T) energies were computed with Molpro 2015 [62,63]. The *1s*-like core orbitals of C, N and O and the *1s*, *2s* and *2p*-like core orbitals of P and S were frozen during all MP2 and CCSD(T) computations.

It should be noted that NH⋯O and PH⋯O contacts were also examined in the *cis* configurations of the aziridine and phosphirane systems. Preliminary M06-2X computations, however, indicate that the structures in which the hydroxyl group accepts what could be described as an intramolecular hydrogen bond from NH or PH are not stabilized to the extent of the corresponding conformations in which the OH group acts as the hydrogen bond donor. As such, only results associated with the OH⋯A interactions are reported and discussed here.

## 3. Results and Discussion

### 3.1. Energetics

In this study, the proposed stabilizing effects of the intramolecular OH⋯O, OH⋯S, OH⋯N and OH⋯P interactions were investigated in a series of 1,2-dialkyl-2,3-epoxy, thiiranyl, aziridinyl and phosphiranyl cyclopentanols. Because the *cis/trans* energetics appear to play such an important role in the underlying chemistry of Type III semipinacol rearrangement reactions, the same relative energies utilized in Reference [21] have been adopted for the current study ($E_{\mathrm{rel}}^{+h.b.}$, $E_{\mathrm{rel}}^{-h.b.}$ and $\Delta E_{h.b.}$). These terms are explicitly defined in Equations (1)–(3) and depicted schematically in Figure 3.

(1) $$E_{\mathrm{rel}}^{-h.b.}=E_{cis-h.b.}-E_{trans}$$

(2) $$E_{\mathrm{rel}}^{+h.b.}=E_{cis+h.b.}-E_{trans}$$

(3) $$\Delta E_{h.b.}E_{cis-h.b.}-E_{cis+h.b.}=E_{\mathrm{rel}}^{-h.b.}-E_{\mathrm{rel}}^{+h.b.}$$

The relative *cis/trans* electronic energies of the isolated (*in vacuo*) species are reported in Table 1 for the TZ basis set. (The data for other basis sets is available in the Supplementary Material). All 3 methods give remarkably consistent results, and they indicate that the electronic energy of the *trans* configuration are significantly lower than those of the non-hydrogen bonded *cis* diastereomers. The corresponding M06-2X, MP2 and CCSD(T) $E_{\mathrm{rel}}^{-h.b.}$ values (Equation (1)) range from +2.0 to +3.6 kcal mol^−1^ for these systems. The identity of the bridging heteroatom (A) only has modest effect on these *cis/trans* energy differences, increasing $E_{\mathrm{rel}}^{-h.b.}$ by approximately 0.8 kcal mol^−1^ from the smallest values (for A = P and O) to the largest (for A = N). Replacing the Me group with Et at either the M or R position has a similar effect and can increase $E_{\mathrm{rel}}^{-h.b.}$ by roughly same amount: from +2.2 to +2.9 kcal mol^−1^ in the epoxide substrates (A = O), from +2.5 to +3.3 kcal mol^−1^ in the thiirane systems (A = S), from +2.8 to +3.6 kcal mol^−1^ in the aziridine compounds (A = N) and from +2.0 to +2.9 kcal mol^−1^ in the phosphirane analogs (A = P).

The M06-2X, MP2 and CCSD(T) $E_{\mathrm{rel}}^{+h.b.}$ data provided in Table 1 and defined in Equation (2) show that all but one of the *cis* isomers become lower in energy than their *trans* counterparts when the OH group is oriented in the *cis* configuration to donate a hydrogen bond to the O, S, N or P atom. For the Me and Et substituted 1,2-dialkyl-2,3-epoxycyclopentanols, the cis+h.b. structures have lower electronic energies with the TZ basis set than the trans isomers by −0.2 to −0.7 kcal mol^−1^ when A = O. The $E_{\mathrm{rel}}^{+h.b.}$ values are quite similar for A = S (−0.1 to −0.8 kcal mol^−1^) and for A = N (−0.4 to −0.9 kcal mol^−1^). Although the trend holds in the phosphirane systems, the trans and cis+h.b. systems become isoenergetic when the Et substituent is at position 1 (i.e., M = Et). Diffuse functions have a negligible impact on the M06-2X energetics reported in Table 1. The corresponding results obtained with the aTZ basis set can be found in the Supplementary Material.

The aforementioned relative energies provide insight into the magnitude of stabilization imparted by the intramolecular OH⋯A contacts in these systems as defined by $\Delta E_{h.b.}$ in Equation (3). The M06-2X, MP2 and CCSD(T) values for the Me and Et substituted 1,2-dialkyl-2,3-epoxycyclopentanols range from −2.7 to −3.5 kcal mol^−1^ for the OH⋯O interactions. The $\Delta E_{h.b.}$ values reported in Table 1 reveal that both the OH⋯S and OH⋯N intramolecular contacts in the systems examined here are potentially stronger, ranging from −3.3 to −3.9 kcal mol^−1^ for the former and −3.6 to −4.1 kcal mol^−1^ for the latter. Although slightly smaller in magnitude than the $\Delta E_{h.b.}$ values for the analogous OH⋯O contacts, these estimates of the stabilization from the intramolecular OH⋯P interaction still approach −3 kcal mol^−1^.

The $\Delta E_{h.b.}$ values reported in Table 1 are entirely consistent with those published elsewhere for intramolecular vicinal hydrogen bonds with an OH donor. For example, an analogous computational analysis of 2-substituted ethanols (with fluoro, amino and nitro groups) yielded corresponding energy differences near 2 kcal mol^−1^ [49]. The *cis/trans* energy difference for 2-fluoro and 2-chlorophenol were found to be slightly larger and on the order of 3 or 4 kcal mol^−1^ from DFT and MP2 computations [50], which is perhaps not surprising given that phenols are significantly more acidic than cyclopentanols. Subsequent analyses of these constrained intramolecular contacts based on the electron density, orbitals and/or electrostatic potential [64,65,66,67,68,69,70,71,72,73] are avoided in the present study because they have been shown to yield rather inconsistent results [34,48].

### 3.2. Bond Lengths, Vibrational Frequencies and NMR Chemical Shielding Constants

When a hydroxyl group forms a typical intermolecular hydrogen bond, the vibrational frequency associated with the covalent OH bond stretch shifts to a lower energy (commonly referred to as a “red shift”). In the gas phase, for example, when one water molecule donates a hydrogen bond to another to form the water dimer, the donor OH stretching frequency shifts to 3602 cm^−1^ which is 55 cm^−1^ lower than the symmetric OH stretch of the water monomer and 154 cm^−1^ lower than the asymmetric stretch [74,75]. Similar changes in spectroscopic and geometrical parameters are known to accompany intramolecular hydrogen bond formation [70,71,72,73,76,77].

Table 2 reports the analogous gas phase OH harmonic stretching frequencies computed with the M06-2X functional and the TZ basis set for all 36 optimized structures. The first three columns list the OH stretching frequencies for the trans, *cis*−h.b. and *cis*+h.b. systems, respectively. Although the OH groups adopt significantly different orientations in the trans and *cis*−h.b. configurations, the corresponding harmonic vibrational frequencies never differ by more than 5 cm^−1^ with the cis−h.b. frequency typically having a slightly larger value by +2 cm^−1^. These differences are tabulated in the penultimate column of data denoted $\Delta \omega_{-h.b.}$ and are defined just like the energy difference in Equation (1).

In stark contrast, the OH stretching frequency is significantly perturbed in the *cis*+h.b. structures all of which exhibit the intramolecular OH⋯A contacts. The last column of data in Table 2 gives the OH stretching frequency difference between the trans and *cis*+h.b. structures (analogous to the energy difference in Equation (2)). The intramolecular OH⋯A contacts cause the frequency to decrease by at least 25 cm^−1^ and by as much as 51 cm^−1^ (with an average change of 41 cm^−1^). The same trends are observed in the M06-2X/aTZ and MP2/TZ harmonic frequency computations within the Supplementary Material. As with the conformational energy differences, the OH stretching frequency shifts reported here are consistent with the other computational studies of 2-substituted alcohols (e.g., −36 cm^−1^ for 2-fluoroethanol and −60 cm^−1^ for 2-nitroethanol) [49,78]. The shifts can be larger for less constrained intramolecular OH⋯A contacts. The experimental difference between the free and hydrogen bonded OH stretches of 1,3-propanediol is −76 cm^−1^ [42], but that is still appreciably smaller than the corresponding −103 cm^−1^ shift induced by the formation of the analogous intermolecular hydrogen bond between methanol and dimethylether [31].

Table 3 provides the related geometrical parameters for each gas phase optimized structure. As expected, the trends in the covalent OH bond lengths reported in the first 3 columns of data are congruent with those for the OH stretching frequencies. The OH bond lengths are nearly identical for the corresponding trans and *cis*−h.b. structures. They never differ by more than 0.0004 Å ($\Delta R_{-h.b.}$), and the cis−h.b. bond length is almost always slightly shorter by approximately 0.0002 Å. In contrast, the differences in the bond lengths are an order of magnitude larger when comparing values for the trans and *cis*+h.b. structures ($\Delta R_{+h.b.}$). The OH bond lengths in Table 3 are typically 0.003 Å longer in the structures exhibiting the intramolecular OH⋯A contacts in accord with the appreciably lower OH stretching frequencies in Table 2. The same bond length changes can be seen for the M06-2X/aTZ and MP2/TZ data in the Supplementary Material.

The corresponding gas phase M06-2X/TZ isotropic NMR chemical shielding constants (σ) for the H atom in the OH functional groups are reported in the first 3 columns of data in Table 4 for the trans, *cis*−h.b. and *cis*+h.b. systems, respectively. The isotropic shielding constants range from 30.40 to 31.96 ppm with the *trans* isomers consistently giving the largest σ values. Without exception, the isotropic shielding constants for the *cis*−h.b. are smaller by −0.26 to −0.65 ppm as indicated by the $\Delta \sigma_{-h.b.}$ column of data. However, when the OH group rotates to form an OH⋯A contact, σ tends to decrease further, by roughly a factor of 2, giving $\Delta \sigma_{+h.b.}$ values that grow to as much as −1.56 ppm (last column of Table 4). In other words, σ is generally smaller for the *cis*+h.b. structures and larger for the *cis*−h.b. conformations. The two exceptions to this trend occur when M = Et and A = S or P. Overall, both the sign and magnitude of these changes are consistent with the formation of intramolecular hydrogen bonds in similar systems. [71,72] The same trends are observed in the M06-2X/aTZ and MP2/TZ NMR data reported in the Supplementary Material. However, it should be noted that changes NMR chemical shifts (often denoted Δδ) have the opposite sign as those associated with isotropic shielding constants (Δσ).

The properties discussed in this section, and changes thereof, are qualitatively consistent with hydrogen bond formation, and the Supplementary Material includes graphs that explore these relationships by plotting the relative electronic energies of the *cis*+h.b. structures ($E_{\mathrm{rel}}^{+h.b.}$ or $\Delta E_{h.b.}$) versus various metrics from Table 2, Table 3 and Table 4. Only the $R_{+h.b.}$ and $\Delta R_{+h.b.}$ covalent OH bond length parameters in Table 3 have a clear correlation with $E_{\mathrm{rel}}^{+h.b.}$ or $\Delta E_{h.b.}$, for which the coefficient of determination ($r^{2}$) from a simple linear regression ranges from 0.83 to 0.89. This value does not exceed 0.66 for any of the other relationships examined in the Supplementary Material. Trends for certain subsets of data could emerge (e.g., for a given hydrogen bond acceptor) as additional systems are investigated, but no general relationships across all systems are apparent from the data plotted in the Supplementary Material apart from those involving the covalent OH bond length. This result is perhaps not too surpising given the highly constrained nature of these intramolecular OH⋯A contacts and the diversity of hydrogen bond accepts (A = O, S, N and P).

## 4. Conclusions

The DFT, MP2 and CCSD(T) computations performed in this study reveal that all of the systems examined exhibit stabilizing OH⋯A intramolecular interactions *in vacuo*, where A = O, S, N and P. A total of 36 unique structures were characterized to probe the relative cis/trans energetics with and without the intramolecular OH⋯A interaction. With simple Me and Et substituents, the cis conformers electronic energies are stabilized by to 2.5 to 4.1 kcal mol^−1^ when the OH group rotates toward the adjacent O, S, N or P atoms. Consequently, the *cis* configurations exhibiting these intramolecular contacts have lower electronic energies than their *trans* counterparts. The intramolecular OH⋯A contacts in the systems studied here also induce OH covalent bond elongation along with a commensurate decrease in the OH stretching frequency and the isotropic NMR chemical shielding constant for the hydroxyl H atom. These findings represent the first theoretical evidence describing a stabilizing intramolecular interaction between hydroxyl and thiirane/phosphirane moieties exhibiting many of the characteristics commonly associated intramolecular hydrogen bonding. From a synthetic perspective, the results also suggest that the *cis* diastereomers can potentially serve as a fascinating starting point toward accessing interesting β-keto alcohols, amines, thiols, and phosphines via Type III semipinacol rearrangement reaction. Future work will probe the distance and directional dependencies of these constrained intramolecular interactions in these cyclic and analogous acyclic systems. 

## Figures and Tables

**Table 1:** Relative electronic energies ($E_{\mathrm{rel}}^{-h.b.}$, $E_{\mathrm{rel}}^{+h.b.}$, $\Delta E_{h.b.}$ all in kcal mol^−1^) computed with the TZ basis set.

A	M	R	M06-2X			MP2			CCSD(T)//MP2		
			$E_{\mathrm{rel}}^{-h.b.}$	$E_{\mathrm{rel}}^{+h.b.}$	$\Delta E_{h.b.}$	$E_{\mathrm{rel}}^{-h.b.}$	$E_{\mathrm{rel}}^{+h.b.}$	$\Delta E_{h.b.}$	$E_{\mathrm{rel}}^{-h.b.}$	$E_{\mathrm{rel}}^{+h.b.}$	$\Delta E_{h.b.}$
O	Me	Me	+2.2	−0.6	−2.8	+2.2	−0.6	−2.7	+2.2	−0.5	−2.8
O	Et	Me	+2.8	−0.7	−3.5	+2.7	−0.6	−3.2	+2.7	−0.4	−3.1
O	Me	Et	+2.6	−0.5	−3.1	+2.8	−0.3	−3.1	+2.9	−0.2	−3.1
S	Me	Me	+2.5	−0.8	−3.3	+2.6	−0.8	−3.3	+2.6	−0.7	−3.3
S	Et	Me	+3.3	−0.6	−3.9	+3.3	−0.4	−3.7	+3.3	−0.1	−3.4
S	Me	Et	+2.9	−0.6	−3.6	+2.7	−0.7	−3.5	+2.7	−0.6	−3.5
N	Me	Me	+2.8	−0.9	−3.7	+2.8	−0.9	−3.6	+2.8	−0.9	−3.7
N	Et	Me	+3.5	−0.4	−3.9	+3.5	−0.4	−3.9	+3.4	−0.5	−3.9
N	Me	Et	+3.2	−0.8	−4.1	+3.6	−0.6	−4.1	+3.5	−0.6	−4.1
P	Me	Me	+2.0	−0.5	−2.5	+2.2	−0.5	−2.7	+2.1	−0.4	−2.5
P	Et	Me	+2.9	−0.0	−2.9	+2.9	+0.1	−2.8	+2.4	−0.0	−2.4
P	Me	Et	+2.4	−0.3	−2.7	+2.8	−0.2	−3.0	+2.7	−0.1	−2.7

**Table 2:** Absolute and relative M06-2X/TZ harmonic OH stretching frequencies (ω and Δω in cm^−1^).

A	M	R	$\omega_{\mathrm{trans}}$	$\omega_{-h.b.}$	$\omega_{+h.b.}$	$\Delta \omega_{-h.b.}$	$\Delta \omega_{+h.b.}$
O	Me	Me	3874	3872	3843	−2	−31
O	Et	Me	3880	3881	3841	+1	−40
O	Me	Et	3870	3872	3843	+2	−27
S	Me	Me	3873	3874	3830	+1	−42
S	Et	Me	3878	3882	3827	+4	−51
S	Me	Et	3869	3874	3828	+5	−41
N	Me	Me	3877	3874	3831	−3	−45
N	Et	Me	3883	3884	3832	+1	−50
N	Me	Et	3867	3873	3826	+1	−45
P	Me	Me	3871	3871	3837	−0	−34
P	Et	Me	3877	3878	3836	+1	−41
P	Me	Et	3868	3872	3836	+4	−32

**Table 3:** Absolute and relative M06-2X/TZ covalent OH bond lengths (*R* and ΔR in Å).

A	M	R	$R_{\mathrm{trans}}$	$R_{-h.b.}$	$R_{+h.b.}$	$\Delta R_{-h.b.}$	$\Delta R_{+h.b.}$
O	Me	Me	0.9609	0.9605	0.9633	−0.0004	+0.0024
O	Et	Me	0.9606	0.9605	0.9636	−0.0001	+0.0030
O	Me	Et	0.9612	0.9610	0.9635	−0.0002	+0.0022
S	Me	Me	0.9611	0.9610	0.9640	−0.0001	+0.0030
S	Et	Me	0.9607	0.9605	0.9641	−0.0002	+0.0034
S	Me	Et	0.9613	0.9610	0.9641	−0.0003	+0.0029
N	Me	Me	0.9608	0.9609	0.9642	+0.0001	+0.0034
N	Et	Me	0.9604	0.9602	0.9641	−0.0002	+0.0037
N	Me	Et	0.9611	0.9609	0.9644	−0.0001	+0.0034
P	Me	Me	0.9612	0.9611	0.9633	−0.0000	+0.0021
P	Et	Me	0.9608	0.9607	0.9633	−0.0001	+0.0024
P	Me	Et	0.9614	0.9611	0.9634	−0.0003	+0.0020

**Table 4:** Absolute and relative M06-2X/TZ isotropic NMR chemical shielding constants for the hydroxyl H atom (σ and Δσ in ppm.)

A	M	R	$\sigma_{\mathrm{trans}}$	$\sigma_{-h.b.}$	$\sigma_{+h.b.}$	$\Delta \sigma_{-h.b.}$	$\Delta \sigma_{+h.b.}$
O	Me	Me	31.90	31.42	31.01	−0.48	−0.90
O	Et	Me	31.12	30.86	30.72	−0.26	−0.40
O	Me	Et	31.89	31.47	30.88	−0.42	−1.01
S	Me	Me	31.84	31.35	30.84	−0.49	−1.00
S	Et	Me	31.14	30.62	30.71	−0.52	−0.43
S	Me	Et	31.80	31.34	30.72	−0.46	−1.08
N	Me	Me	31.90	31.51	30.57	−0.39	−1.32
N	Et	Me	31.22	30.90	30.54	−0.32	−0.68
N	Me	Et	31.96	31.59	30.40	−0.37	−1.56
P	Me	Me	31.96	31.42	31.11	−0.54	−0.86
P	Et	Me	31.27	30.62	31.12	−0.65	−0.15
P	Me	Et	31.94	31.49	31.01	−0.45	−0.94

## Supplementary Materials

The Supplementary Materials are available online.
