# Supplementary material for: Cis/Trans Energetics in Epoxide, Thiirane, Aziridine and Phosphirane Containing Cyclopentanols: Effects of Intramolecular OH⋯O, S, N and P Contacts
Source: Molecules. 2019 Jul 10;24(14):2523. doi: 10.3390/molecules24142523 (PMC6680949; doi:10.3390/molecules24142523)
Supplement: Supplementary file 1 [file molecules-24-02523-s001.pdf]

Supplementary Material for:

Cis/Trans Energetics in Epoxide, Thiirane, Aziridine and Phosphirane Containing Cyclopentanols: Effects of Intramolecular OH···O, S, N and P Contacts

Ben Edward Smith,[†] Jeremy M. Carr[‡] and Gregory S. Tschumper[†*]

[†] Department of Chemistry and Biochemistry, University of Mississippi, University, MS 38677-1848
[‡] Chemistry Department, Central Alabama Community College, Alexander City, AL 35010
[*] tschumper@olemiss.edu

Table 1: Optimized bond lengths and angles associated with the intramolecular OH$\cdots$A contacts (R(H$\cdots$A) in Å and θ(OH$\cdots$A) in degrees, respectively).

			M06-2X/cc-pVTZ		M06-2X/aug-cc-pVTZ		MP2/cc-pVTZ	
A	M	R	R(H$\cdots$A)	θ(OH$\cdots$A)	R(H$\cdots$A)	θ(OH$\cdots$A)	R(H$\cdots$A)	θ(OH$\cdots$A)
O	Me	Me	2.59	104	2.61	103	2.53	105
O	Et	Me	2.50	106	2.50	106	2.47	108
O	Me	Et	2.54	105	2.54	105	2.53	107
S	Me	Me	2.78	112	2.79	111	2.76	113
S	Et	Me	2.70	114	2.70	113	2.65	116
S	Me	Et	2.72	113	2.73	112	2.69	115
NH	Me	Me	2.53	107	2.55	106	2.52	108
NH	Et	Me	2.51	108	2.46	108	2.50	109
NH	Me	Et	2.48	108	2.50	108	2.44	110
PH	Me	Me	2.83	113	2.83	113	2.80	115
PH	Et	Me	2.74	115	2.74	115	2.69	118
PH	Me	Et	2.79	114	2.79	114	2.74	116

Table 2: Absolute and relative M06-2X covalent OH bond lengths (R and ΔR in Å) computed with the cc-pV(T+d)Z basis set.

A	M	R	R_{trans}	$R_{-\text{h.b.}}$	$R_{+\text{h.b.}}$	$\Delta R_{-\text{h.b.}}$	$\Delta R_{+\text{h.b.}}$
S	Me	Me	0.96105	0.96099	0.96339	−0.0001	+0.0023
S	Et	Me	0.96070	0.96051	0.96405	−0.0002	+0.0033
S	Me	Et	0.96125	0.96097	0.96408	−0.0003	+0.0028
PH	Me	Me	0.96118	0.96113	0.96329	+0.0000	+0.0021
PH	Et	Me	0.96082	0.96071	0.96323	−0.0001	+0.0024
PH	Me	Et	0.96131	0.96108	0.96334	−0.0002	+0.0020

Table 3: Absolute and relative M06-2X harmonic OH stretching frequencies (ω and $\Delta\omega$ in cm^{-1}) computed with the cc-pV(T+d)Z basis set.

A	M	R	ω_{trans}	$\omega_{-\text{h.b.}}$	$\omega_{+\text{h.b.}}$	$\Delta\omega_{-\text{h.b.}}$	$\Delta\omega_{+\text{h.b.}}$
S	Me	Me	3874	3831	3873	−42	−43
S	Et	Me	3882	3828	3878	−50	−54
S	Me	Et	3874	3829	3869	−41	−45
PH	Me	Me	3871	3838	3871	−33	−33
PH	Et	Me	3878	3836	3877	−41	−42
PH	Me	Et	3871	3836	3868	−32	−35

Table 4: Absolute and relative M06-2X harmonic OH stretching frequencies (ω and $\Delta\omega$ in cm^{-1}) computed with the aTZ basis set.

A	M	R	ω_{trans}	$\omega_{-\text{h.b.}}$	$\omega_{+\text{h.b.}}$	$\Delta\omega_{-\text{h.b.}}$	$\Delta\omega_{+\text{h.b.}}$
O	Me	Me	3869	3872	3840	+3	−29
O	Et	Me	3875	3876	3838	+1	−38
O	Me	Et	3865	3868	3841	+3	−25
S	Me	Me	3868	3870	3825	+2	−43
S	Et	Me	3873	3876	3823	+3	−51
S	Me	Et	3870	3870	3824	−0	−46
N	Me	Me	3872	3870	3829	−2	−44
N	Et	Me	3874	3873	3823	−1	−52
N	Me	Et	3867	3867	2824	+0	−43
P	Me	Me	3867	3867	3833	+1	−33
P	Et	Me	3872	3875	3831	+3	−41
P	Me	Et	3862	3868	3831	+5	−31

Table 5: Absolute and relative M06-2X covalent OH bond lengths (R and ΔR in Å) computed with the aTZ basis set.

A	M	R	R_{trans}	$R_{-\text{h.b.}}$	$R_{+\text{h.b.}}$	$\Delta R_{-\text{h.b.}}$	$\Delta R_{+\text{h.b.}}$
O	Me	Me	0.96087	0.96086	0.96310	−0.0000	+0.0022
O	Et	Me	0.96052	0.96038	0.96337	−0.0001	+0.0028
O	Me	Et	0.96111	0.96088	0.96325	−0.0002	+0.0021
S	Me	Me	0.96099	0.96089	0.96395	−0.0001	+0.0030
S	Et	Me	0.96066	0.96040	0.96402	−0.0003	+0.0034
S	Me	Et	0.96093	0.96085	0.96405	−0.0001	+0.0031
NH	Me	Me	0.96070	0.96082	0.96398	+0.0001	+0.0033
NH	Et	Me	0.96057	0.96070	0.96428	+0.0001	+0.0037
NH	Me	Et	0.96095	0.96098	0.96419	−0.0000	+0.0032
PH	Me	Me	0.96110	0.96098	0.96324	−0.0001	+0.0021
PH	Et	Me	0.96076	0.96057	0.96320	−0.0002	+0.0024
PH	Me	Et	0.96129	0.96093	0.96329	−0.0004	+0.0020

Table 6: Absolute and relative MP2 covalent OH bond lengths (R and ΔR in Å) computed with the TZ basis set.

A	M	R	R_{trans}	$R_{-\text{h.b.}}$	$R_{+\text{h.b.}}$	$\Delta R_{-\text{h.b.}}$	$\Delta R_{+\text{h.b.}}$
O	Me	Me	0.9635	0.9633	0.9655	−0.0002	+0.0020
O	Et	Me	0.9631	0.9628	0.9661	−0.0004	+0.0030
O	Me	Et	0.9638	0.9634	0.9658	−0.0004	+0.0020
S	Me	Me	0.9639	0.9636	0.9667	−0.0003	+0.0029
S	Et	Me	0.9636	0.9631	0.9673	−0.0005	+0.0037
S	Me	Et	0.9639	0.9636	0.9671	−0.0003	+0.0031
NH	Me	Me	0.9635	0.9633	0.9666	−0.0001	+0.0032
NH	Et	Me	0.9631	0.9627	0.9668	−0.0004	+0.0037
NH	Me	Et	0.9639	0.9635	0.9671	−0.0004	+0.0033
PH	Me	Me	0.9641	0.9638	0.9663	−0.0003	+0.0022
PH	Et	Me	0.9637	0.9634	0.9668	−0.0003	+0.0031
PH	Me	Et	0.9646	0.9639	0.9666	−0.0007	+0.0020

Table 7: Absolute and relative MP2 harmonic OH stretching frequencies (ω and $\Delta\omega$ in cm^{-1}) computed with the TZ basis set.

A	M	R	ω_{trans}	$\omega_{-\text{h.b.}}$	$\omega_{+\text{h.b.}}$	$\Delta\omega_{-\text{h.b.}}$	$\Delta\omega_{+\text{h.b.}}$
O	Me	Me	3830	3801	3826	−26	−29
O	Et	Me	3838	3792	3832	−40	−46
O	Me	Et	3828	3796	3820	−24	−31
S	Me	Me	3826	3776	3821	−45	−50
S	Et	Me	3834	3764	3826	−62	−70
S	Me	Et	3824	3809	3814	−5	−15
NH	Me	Me	3859	3814	3862	−48	−45
NH	Et	Me	3870	3815	3868	−53	−55
NH	Me	Et	3859	3810	3856	−46	−48
PH	Me	Me	3857	3819	3856	−36	−37
PH	Et	Me	3864	3817	3861	−44	−47
PH	Me	Et	3857	3818	3851	−33	−39

Table 8: Absolute and relative M06-2X/aTZ isotropic NMR chemical shielding constants for the hydroxyl H atom (σ and $\Delta\sigma$ in ppm).

A	M	R	σ_{trans}	$\sigma_{-\text{h.b.}}$	$\sigma_{+\text{h.b.}}$	$\Delta\sigma_{-\text{h.b.}}$	$\Delta\sigma_{+\text{h.b.}}$
O	Me	Me	31.74	31.32	30.94	−0.42	−0.80
O	Et	Me	31.00	30.79	30.74	−0.21	−0.27
O	Me	Et	31.64	31.30	30.88	−0.34	−0.76
S	Me	Me	31.65	31.18	30.83	−0.46	−0.81
S	Et	Me	30.95	30.54	30.67	−0.41	−0.28
S	Me	Et	31.58	31.16	30.66	−0.42	−0.92
NH	Me	Me	31.91	30.57	30.64	−1.35	−1.27
NH	Et	Me	30.99	30.72	30.34	−0.27	−0.65
NH	Me	Et	31.74	31.32	30.44	−0.42	−1.30
PH	Me	Me	31.76	31.24	31.11	−0.52	−0.65
PH	Et	Me	31.07	30.50	30.95	−0.58	−0.12
PH	Me	Et	31.77	31.25	30.93	−0.53	−0.84

Table 9: Absolute and relative MP2/TZ isotropic NMR chemical shielding constants for the hydroxyl H atom (σ and $\Delta\sigma$ in ppm).

A	M	R	σ_{trans}	$\sigma_{-\text{h.b.}}$	$\sigma_{+\text{h.b.}}$	$\Delta\sigma_{-\text{h.b.}}$	$\Delta\sigma_{+\text{h.b.}}$
O	Me	Me	31.47	31.39	30.58	−0.08	−0.89
O	Et	Me	31.11	30.43	30.55	−0.68	−0.56
O	Me	Et	31.77	31.02	30.68	−0.74	−1.08
S	Me	Me	31.37	30.90	30.63	−0.47	−0.74
S	Et	Me	31.09	30.71	30.45	−0.39	−0.65
S	Me	Et	31.68	31.38	30.62	−0.30	−1.06
NH	Me	Me	31.87	31.09	30.48	−0.78	−1.39
NH	Et	Me	31.23	30.97	30.44	−0.26	−0.78
NH	Me	Et	31.88	31.54	30.25	−0.34	−1.63
PH	Me	Me	31.74	31.40	30.87	−0.34	−0.87
PH	Et	Me	31.28	30.32	30.90	−0.96	−0.38
PH	Me	Et	31.88	31.53	30.91	−0.34	−0.97

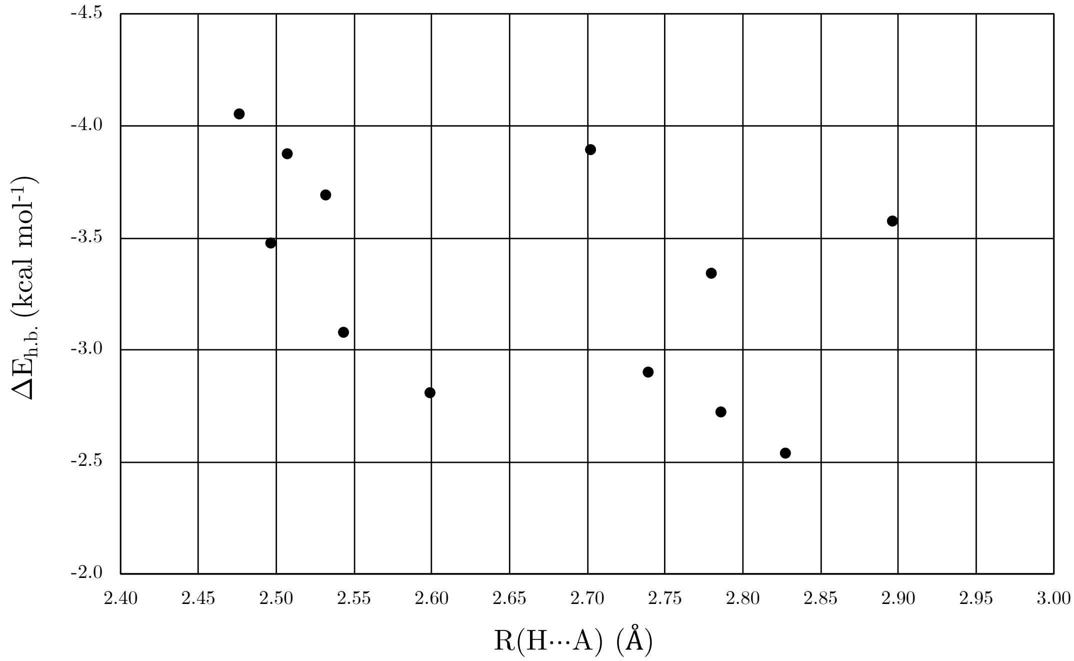

Figure 1: M06-2X/TZ relative electronic energies of the *cis*+h.b. structures ($\Delta E_{\text{h.b.}}$ in kcal mol^{-1} as defined by Equation 3) versus the H···A distances (R(H···A) in Å) associated with the intramolecular hydrogen bonds. Trend lines and coefficients of determination are not shown when $r^2 < 0.5$.

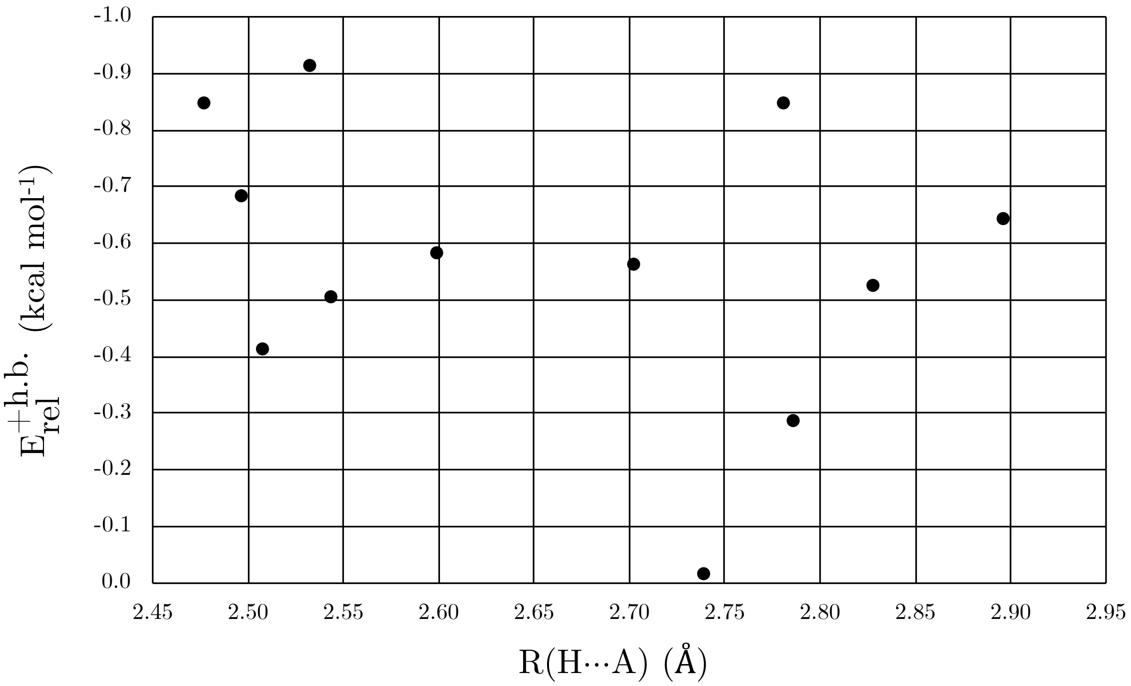

Figure 2: M06-2X/TZ relative electronic energies of the *cis*+h.b. structures ($E_{\text{rel}}^{+\text{h.b.}}$ in kcal mol^{-1} as defined by Equation 2) versus the H···A distances (R(H···A) in Å) associated with the intramolecular hydrogen bonds. Trend lines and coefficients of determination are not shown when $r^2 < 0.5$.

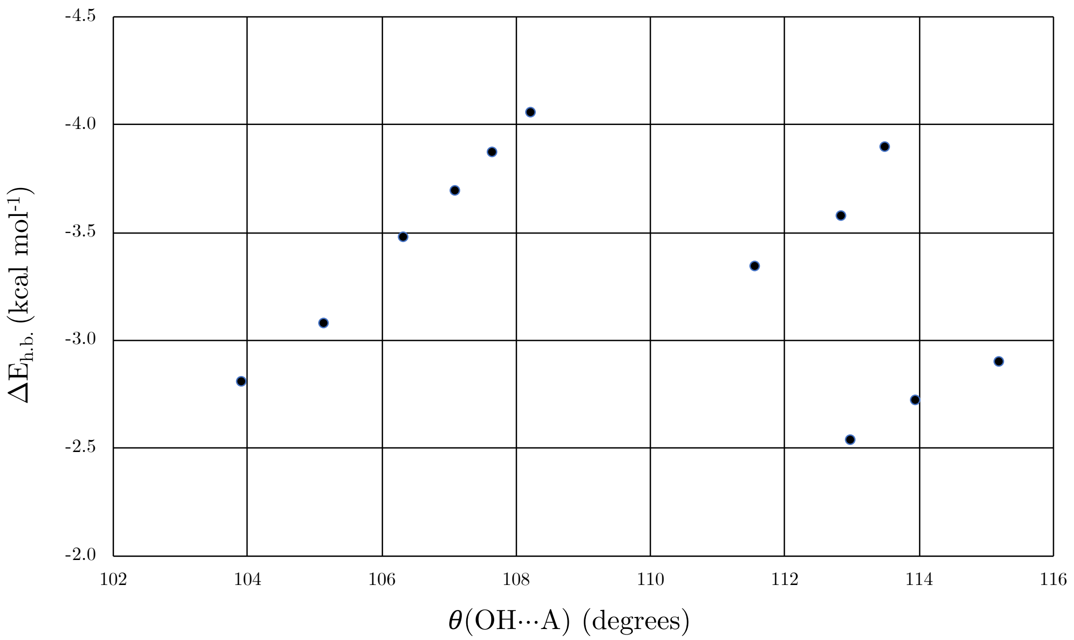

Figure 3: M06-2X/TZ relative electronic energies of the *cis*+h.b. structures ($\Delta E_{h.b.}$ in kcal mol^{-1} as defined by Equation 3) versus the OH$\cdots$A bond angles (θ(OH$\cdots$A) in degrees) associated with the intramolecular hydrogen bonds. Trend lines and coefficients of determination are not shown when $r^2 < 0.5$.

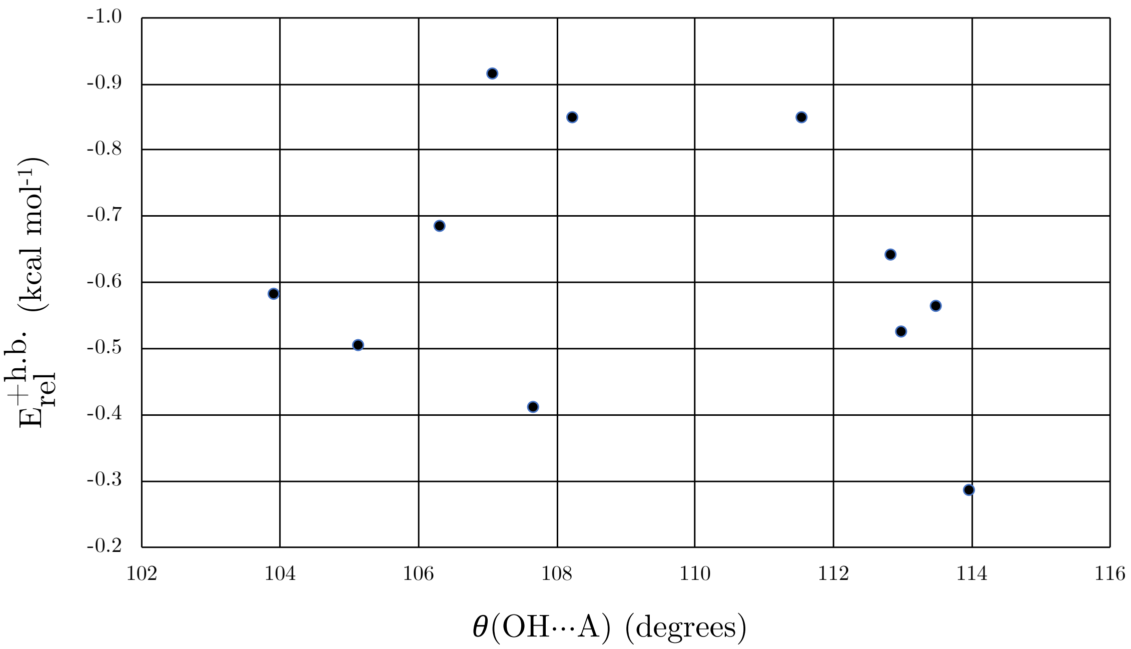

Figure 4: M06-2X/TZ relative electronic energies of the *cis*+h.b. structures ($E_{rel}^{+h.b.}$ in kcal mol^{-1} as defined by Equation 2) versus the OH$\cdots$A bond angles (θ(OH$\cdots$A) in degrees) associated with the intramolecular hydrogen bonds. Trend lines and coefficients of determination are not shown when $r^2 < 0.5$.

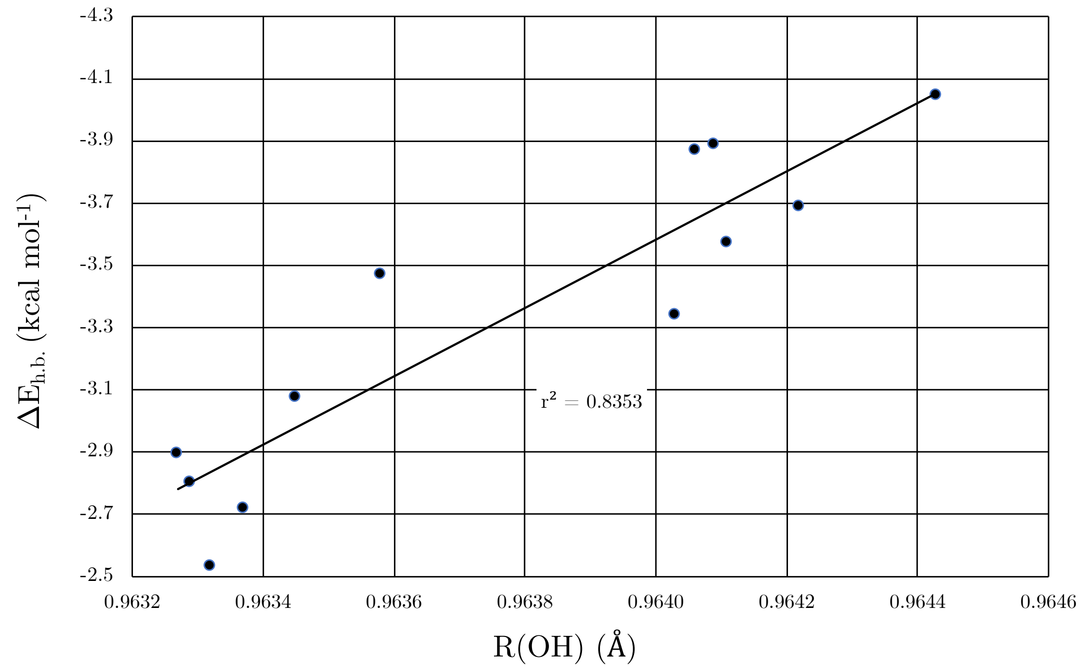

Figure 5: M06-2X/TZ relative electronic energies of the *cis*+h.b. structures ($\Delta E_{h.b.}$ in kcal mol^{-1} as defined by Equation 3) versus the *cis*+h.b. covalent OH bond lengths (R(OH) in Å). Trend lines and coefficients of determination are shown when $r^2 > 0.5$.

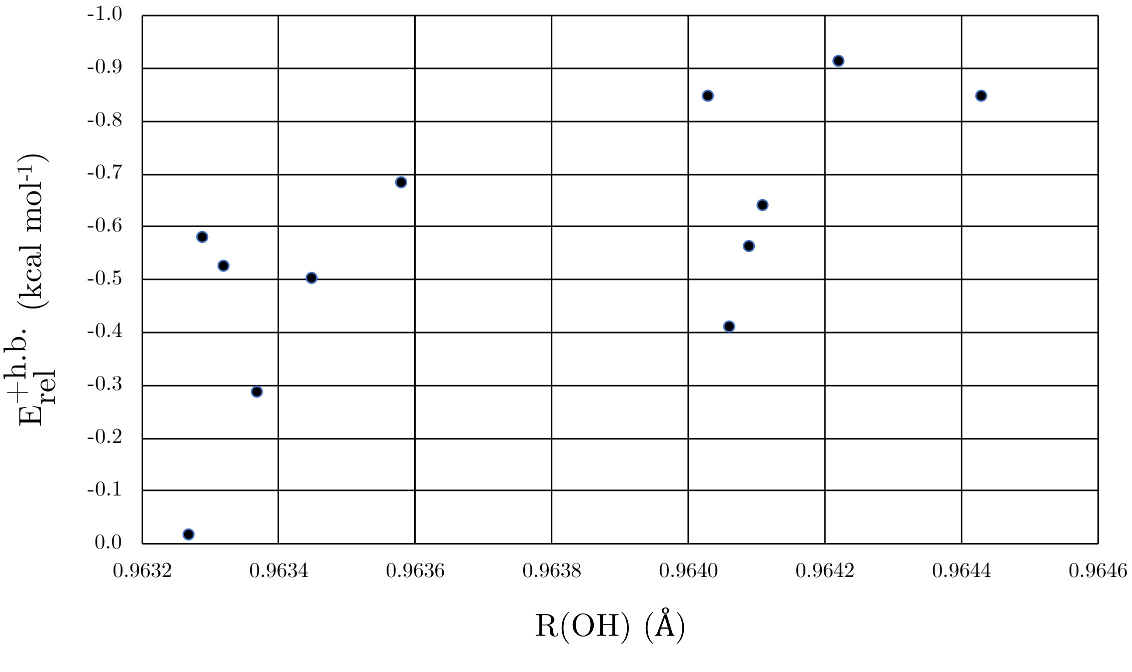

Figure 6: M06-2X/TZ relative electronic energies of the *cis*+h.b. structures ($E_{rel}^{+h.b.}$ in kcal mol^{-1} as defined by Equation 2) versus the *cis*+h.b. covalent OH bond lengths (R(OH) in Å). Trend lines and coefficients of determination are not shown when $r^2 < 0.5$.

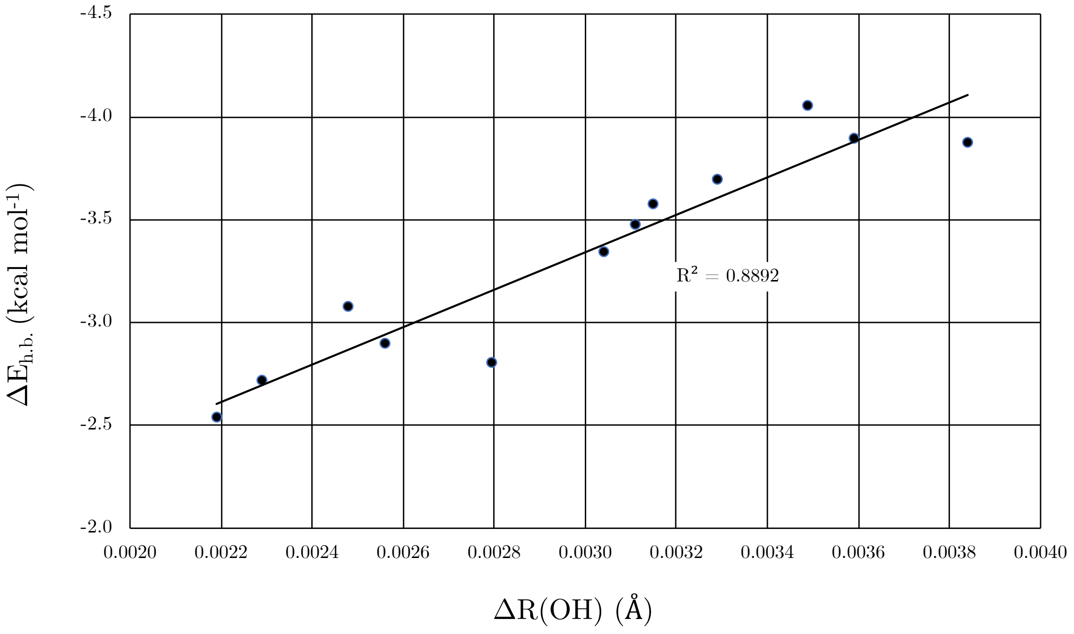

Figure 7: M06-2X/TZ relative electronic energies of the *cis*+h.b. structures ($\Delta E_{\text{h.b.}}$ in kcal mol^{-1} as defined by Equation 3) versus the corresponding changes in the covalent OH bond lengths (ΔR(OH) in Å). Trend lines and coefficients of determination are shown when $r^2 > 0.5$.

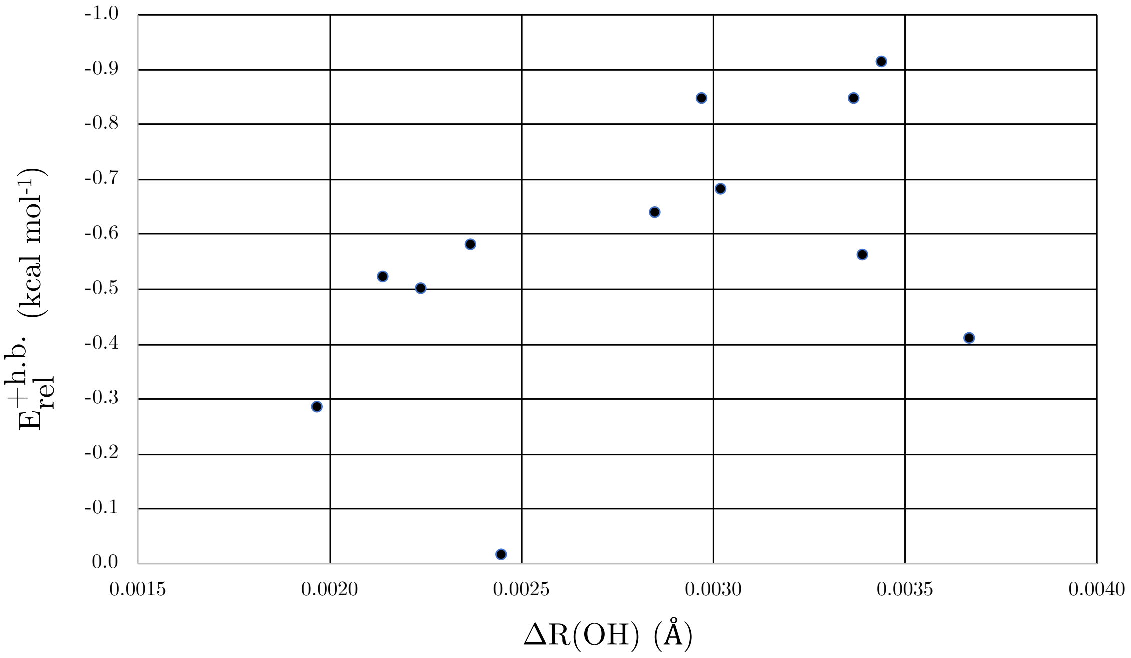

Figure 8: M06-2X/TZ relative electronic energies of the *cis*+h.b. structures ($E_{\text{rel}}^{+\text{h.b.}}$ in kcal mol^{-1} as defined by Equation 2) versus the corresponding changes in the covalent OH bond lengths (ΔR(OH) in Å). Trend lines and coefficients of determination are not shown when $r^2 < 0.5$.

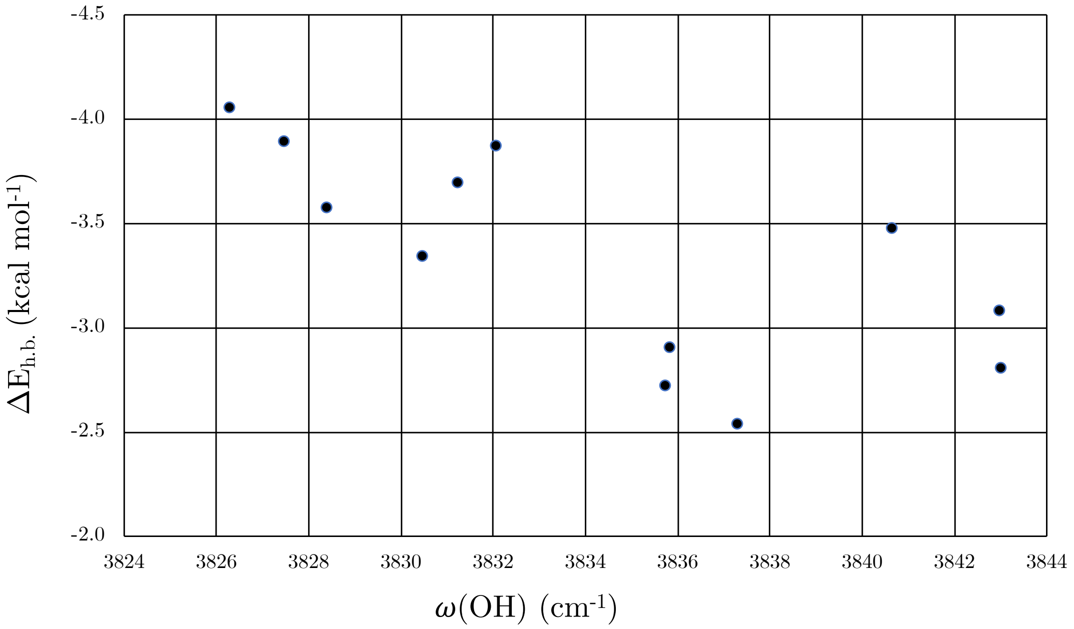

Figure 9: M06-2X/TZ relative electronic energies of the *cis*+h.b. structures ($\Delta E_{h.b.}$ in kcal mol^{-1} as defined by Equation 3) versus the *cis*+h.b. harmonic OH stretching frequencies (ω(OH) in cm^{-1}). Trend lines and coefficients of determination are not shown when $r^2 < 0.5$.

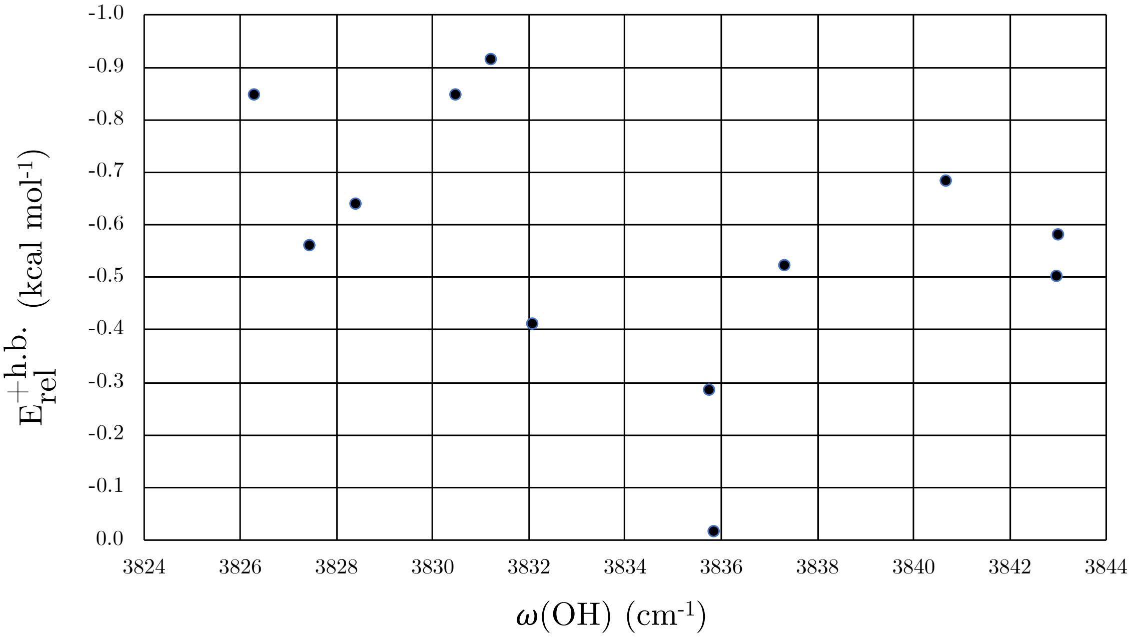

Figure 10: M06-2X/TZ relative electronic energies of the *cis*+h.b. structures ($E_{rel}^{+h.b.}$ in kcal mol^{-1} as defined by Equation 2) versus the *cis*+h.b. harmonic OH stretching frequencies (ω(OH) in cm^{-1}). Trend lines and coefficients of determination are not shown when $r^2 < 0.5$.

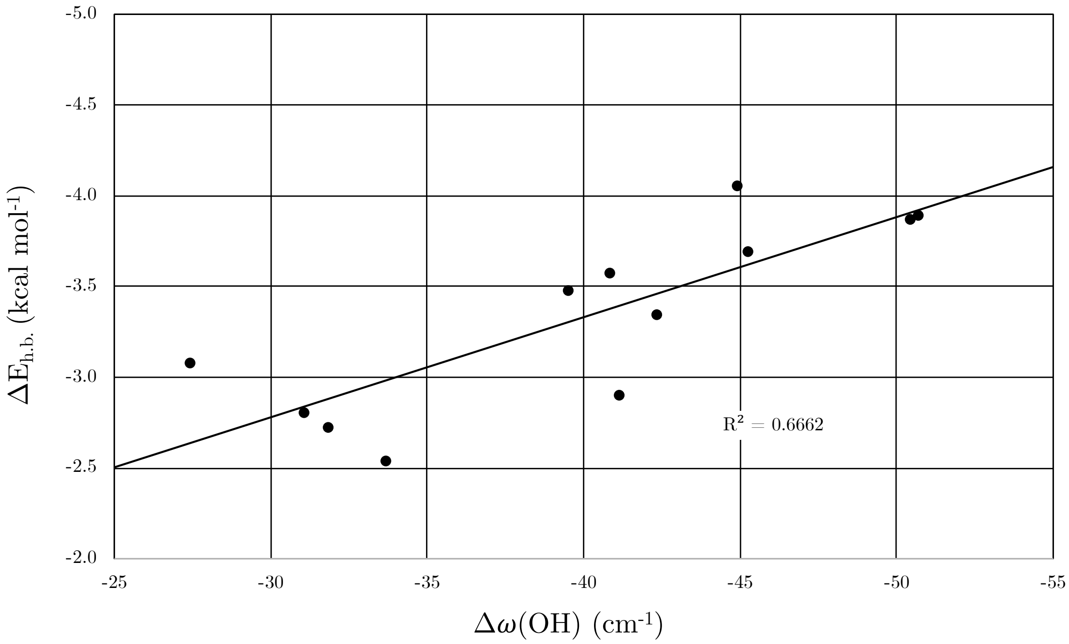

Figure 11: M06-2X/TZ relative electronic energies of the *cis*+h.b. structures ($\Delta E_{h.b.}$ in kcal mol^{-1} as defined by Equation 3) versus the corresponding changes in the harmonic OH stretching frequencies ($\Delta\omega$(OH) in cm^{-1}). Trend lines and coefficients of determination are shown when $r^2 > 0.5$.

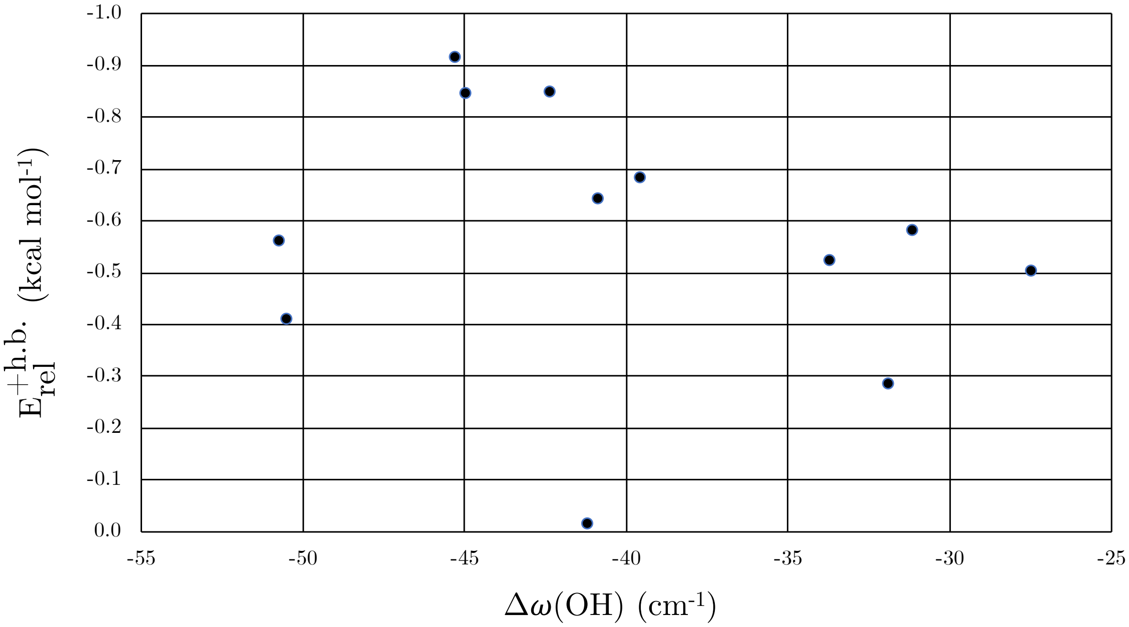

Figure 12: M06-2X/TZ relative electronic energies of the *cis*+h.b. structures ($E_{rel}^{+h.b.}$ in kcal mol^{-1} as defined by Equation 2) versus the corresponding changes in the harmonic OH stretching frequencies ($\Delta\omega$(OH) in cm^{-1}). Trend lines and coefficients of determination are not shown when $r^2 < 0.5$.

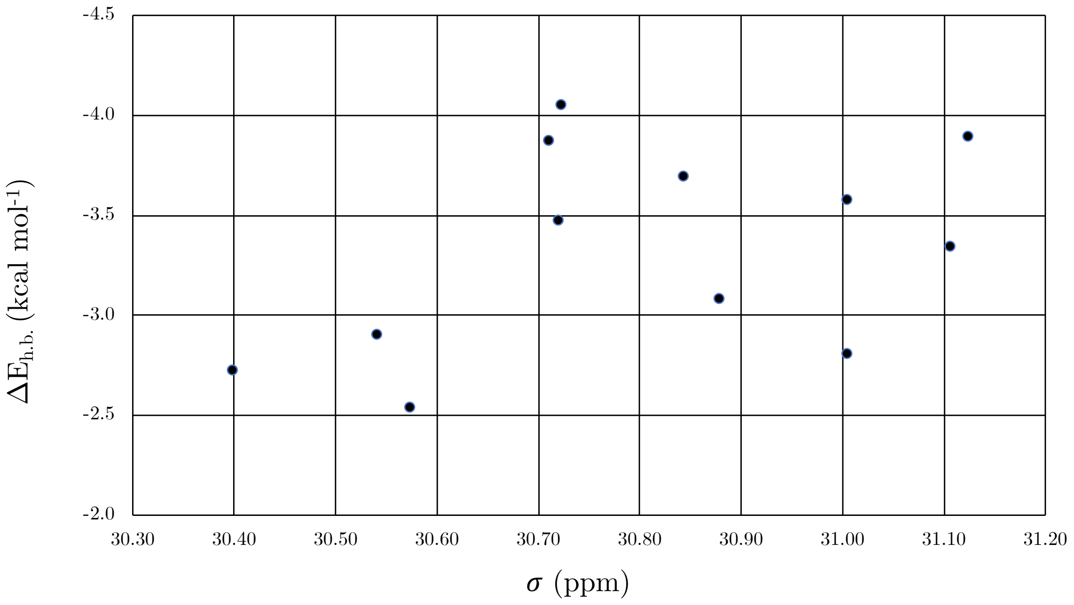

Figure 13: M06-2X/TZ relative electronic energies of the *cis*+h.b. structures ($\Delta E_{h.b.}$ in kcal mol^{-1} as defined by Equation 3) versus the *cis*+h.b. isotropic NMR chemical shielding constants for the hydroxyl H atom (σ in ppm). Trend lines and coefficients of determination are not shown when $r^2 < 0.5$.

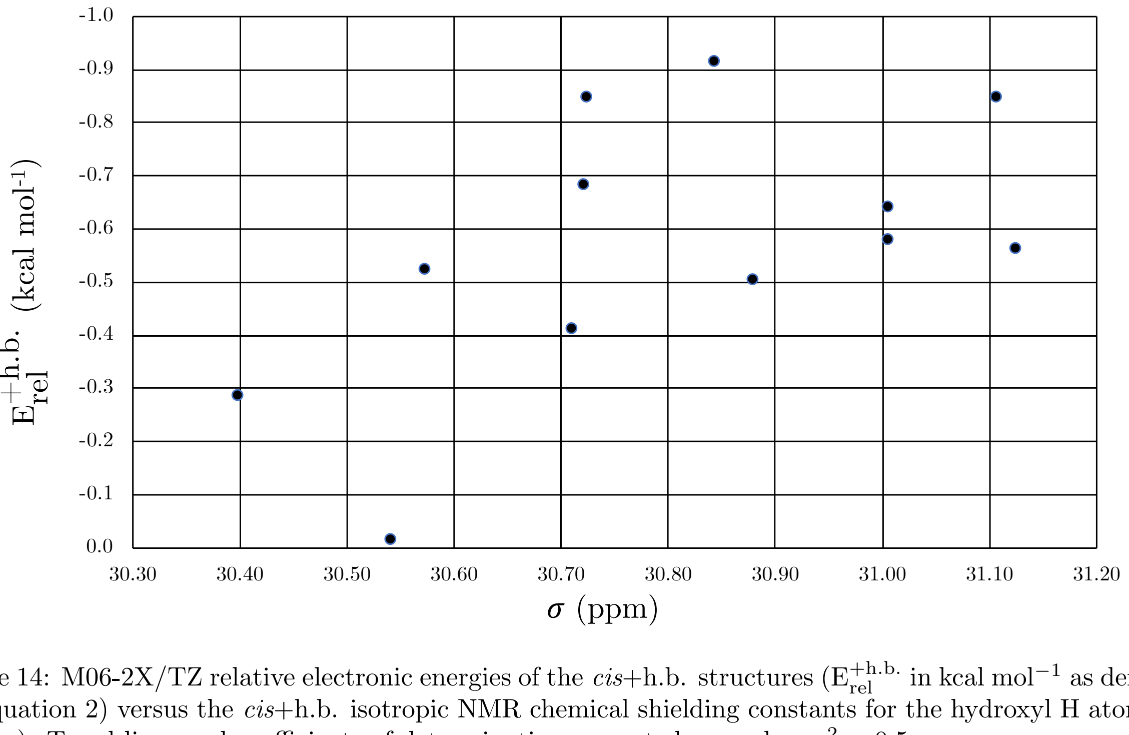

Figure 14: M06-2X/TZ relative electronic energies of the *cis*+h.b. structures ($E_{rel}^{+h.b.}$ in kcal mol^{-1} as defined by Equation 2) versus the *cis*+h.b. isotropic NMR chemical shielding constants for the hydroxyl H atom (σ in ppm). Trend lines and coefficients of determination are not shown when $r^2 < 0.5$.

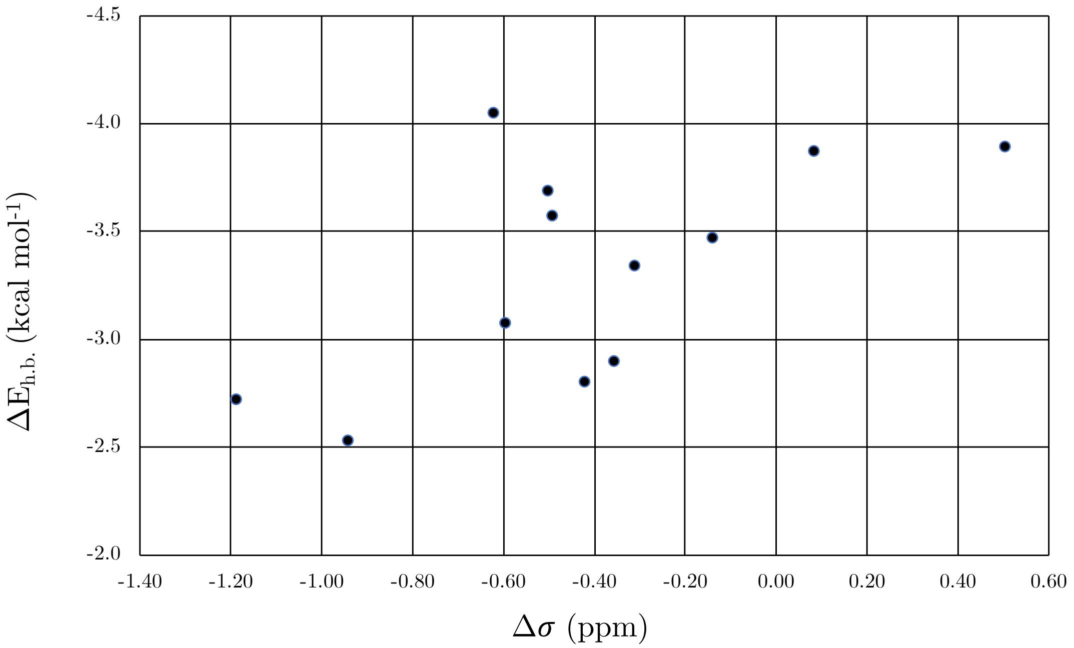

Figure 15: M06-2X/TZ relative electronic energies of the *cis*+h.b. structures ($\Delta E_{h.b.}$ in kcal mol^{-1} as defined by Equation 3) versus the corresponding changes in the isotropic NMR chemical shielding constants for the hydroxyl H atom ($\Delta\sigma$ in ppm). Trend lines and coefficients of determination are not shown when $r^2 < 0.5$.

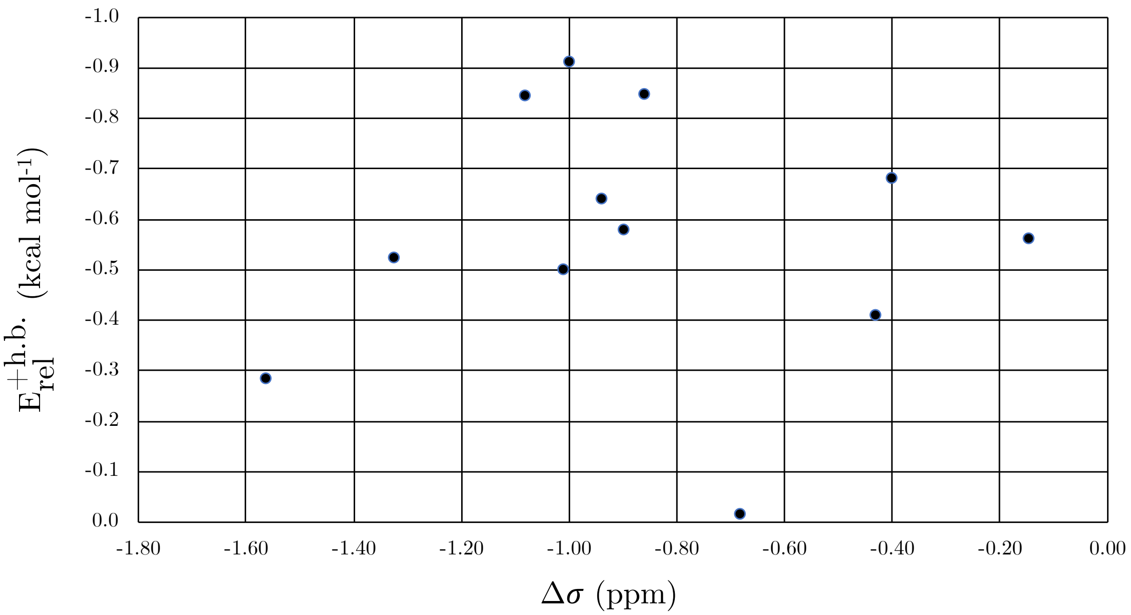

Figure 16: M06-2X/TZ relative electronic energies of the *cis*+h.b. structures ($E_{rel}^{+h.b.}$ in kcal mol^{-1} as defined by Equation 2) versus the corresponding changes in the isotropic NMR chemical shielding constants for the hydroxyl H atom ($\Delta\sigma$ in ppm). Trend lines and coefficients of determination are not shown when $r^2 < 0.5$.

Table 10: M06-2X optimized geometry of *cis*-2,3-epoxy-1,2-dimethylcyclopentan-1-ol (not H-bonded) (in Å) with the TZ basis set

Atom	x	y	z
C	0.257707	0.783401	0.023501
C	1.555838	0.187343	0.350531
C	1.430101	−1.316467	0.425951
C	0.123092	−1.588119	−0.335669
C	−0.756641	−0.345461	−0.099039
C	−1.566261	−0.468483	1.186701
H	−2.239359	−1.325524	1.120141
H	−0.914940	−0.618912	2.049321
H	−2.165522	0.425936	1.349861
O	−1.610321	−0.053383	−1.192309
H	−2.263680	−0.753724	−1.270069
H	0.323072	−1.650529	−1.402719
H	−0.371117	−2.505660	−0.016729
H	2.285192	−1.828065	−0.014829
H	1.359642	−1.622387	1.471721
O	1.288007	0.688413	−0.953779
H	2.313497	0.723515	0.908871
C	−0.186496	2.168070	0.379261
H	−0.614616	2.202539	1.380491
H	0.663493	2.846082	0.337191
H	−0.941187	2.504788	−0.331969

Table 11: M06-2X optimized geometry of *cis*-2,3-epoxy-1,2-dimethylcyclopentan-1-ol (H-bonded) (in Å) with the TZ basis set

Atom	x	y	z
C	−0.018659	−0.003960	0.020192
C	1.526907	−0.031572	0.022235
C	1.982671	1.434395	0.015061
C	0.855625	2.141364	−0.698264
C	−0.351005	1.317219	−0.666379
C	−1.758863	1.820755	−0.717933
H	−2.415337	1.045402	−1.115205
H	−2.113695	2.093131	0.275473
H	−1.814680	2.694687	−1.363873
O	0.414365	1.451721	−1.868034
H	0.814239	3.221685	−0.762574
H	2.066016	1.835291	1.027268
H	2.944169	1.573789	−0.478192
H	1.915835	−0.596758	0.867110
H	1.861265	−0.523472	−0.889503
O	−0.578319	−1.098214	−0.684220
H	−0.269862	−1.040915	−1.594991
C	−0.592203	−0.053746	1.423381
H	−0.304556	−0.990604	1.898396
H	−0.213784	0.775826	2.022698
H	−1.679648	−0.002979	1.394568

Table 12: M06-2X optimized geometry of *trans*-2,3-epoxy-1,2-dimethylcyclopentan-1-ol (in Å) with the TZ basis set

Atom	x	y	z
C	0.724709	−0.411326	0.005455
C	−0.228458	−1.587610	0.280719
C	−1.570646	−1.183894	−0.348661
C	−1.566362	0.318299	−0.217065
C	−0.199550	0.800157	−0.015101
O	−1.124149	0.764707	1.066146
H	−2.328335	0.932729	−0.679865
H	−1.604658	−1.442717	−1.407632
H	−2.422662	−1.648393	0.146468
H	0.175099	−2.511972	−0.132194
H	−0.343889	−1.717250	1.354971
C	0.324530	2.141380	−0.418269
H	−0.491807	2.858745	−0.477817
H	1.044317	2.503844	0.317371
H	0.820879	2.072957	−1.384305
C	1.845109	−0.293867	1.021539
H	2.415062	−1.224395	1.073482
H	2.525265	0.511382	0.744558
H	1.435716	−0.089996	2.010890
O	1.240252	−0.481898	−1.322745
H	1.870299	−1.206222	−1.364824

Table 13: M06-2X optimized geometry of *cis*-2,3-epoxy-1-ethyl-2-methylcyclopentan-1-ol (not H-bonded) (in Å) with the TZ basis set

Atom	x	y	z
C	−0.919335	0.587805	−0.105864
C	−1.714982	−0.547726	−0.580181
C	−0.910517	−1.821030	−0.469953
C	0.193213	−1.441145	0.529669
C	0.450601	0.063094	0.309093
C	1.466448	0.323903	−0.809881
C	2.890346	−0.094544	−0.462711
H	2.966012	−1.158480	−0.238236
H	3.563572	0.111686	−1.293433
H	3.260766	0.465060	0.396840
H	1.131757	−0.191968	−1.714265
H	1.461957	1.392150	−1.030104
O	0.834487	0.740452	1.494536
H	1.585419	0.285089	1.883443
H	−0.175048	−1.564986	1.545648
H	1.091854	−2.043897	0.411116
H	−1.509907	−2.664286	−0.127897
H	−0.498936	−2.077733	−1.448093
O	−1.965520	0.040491	0.690186
H	−2.506030	−0.434995	−1.311508
C	−1.100621	2.014742	−0.524215
H	−0.586807	2.227141	−1.460654
H	−2.161428	2.219954	−0.653055
H	−0.705771	2.677121	0.246750

Table 14: M06-2X optimized geometry of *cis*-2,3-epoxy-1-ethyl-2-methylcyclopentan-1-ol (H-bonded) (in Å) with the TZ basis set

Atom	x	y	z
C	0.426520	0.099420	0.662900
C	−0.652340	0.602520	−0.291320
C	−1.373630	−0.542420	−0.844730
C	−0.757220	−1.831560	−0.362850
C	0.080290	−1.399430	0.852190
H	0.977360	−2.003150	0.983310
H	−0.510290	−1.493920	1.761350
H	−0.137140	−2.246080	−1.158950
H	−1.511070	−2.575550	−0.105970
O	−1.940670	0.230810	0.212300
H	−1.905950	−0.487310	−1.786630
C	−0.555070	1.961750	−0.908560
H	−1.475390	2.193480	−1.441010
H	−0.400250	2.709880	−0.129820
H	0.280730	2.017430	−1.605990
O	0.366310	0.816900	1.884570
H	−0.543150	0.766280	2.198910
C	1.830770	0.330480	0.121310
C	2.120020	−0.382360	−1.194060
H	1.400950	−0.108560	−1.968380
H	3.113420	−0.125820	−1.558520
H	2.084390	−1.465730	−1.076350
H	1.975240	1.406940	0.012300
H	2.529970	0.000050	0.891510

Table 15: M06-2X optimized geometry of *trans*-2,3-epoxy-1-ethyl-2-methylcyclopentan-1-ol (in Å) with the TZ basis set

Atom	x	y	z
C	−0.466810	−0.011006	0.210317
C	0.887405	0.635761	−0.057835
C	1.903359	−0.416542	−0.034021
C	1.260645	−1.747127	0.261963
C	−0.217233	−1.502761	−0.081207
H	−0.892634	−2.133212	0.496717
H	−0.392966	−1.700320	−1.137421
H	1.388237	−1.969842	1.322050
H	1.694004	−2.563532	−0.314768
O	1.462958	0.154154	−1.267478
H	2.934718	−0.212055	0.224679
C	1.115784	2.075621	0.276337
H	2.164752	2.329382	0.135133
H	0.519372	2.716286	−0.374882
H	0.830187	2.268414	1.308690
C	−1.583112	0.601170	−0.626394
C	−2.929122	−0.091624	−0.445971
H	−2.878304	−1.141821	−0.734652
H	−3.271270	−0.040242	0.589835
H	−3.696283	0.383167	−1.055630
H	−1.672484	1.654783	−0.353893
H	−1.277645	0.558670	−1.674106
O	−0.684835	0.199731	1.604233
H	−1.500330	−0.241547	1.855064

Table 16: M06-2X optimized geometry of *cis*-2-ethyl-2,3- epoxy-1-methylcyclopentan-1-ol (not H-bonded) (in Å) with the TZ basis set

Atom	x	y	z
C	−0.397314	−0.292295	0.373495
C	0.080917	−1.566391	−0.173497
C	1.418305	−1.370674	−0.849021
C	1.915653	−0.049653	−0.241718
C	0.646346	0.772170	0.058084
C	0.223650	1.607817	−1.143875
H	1.005668	2.332839	−1.379130
H	0.066946	0.984512	−2.025688
H	−0.694414	2.153378	−0.928589
O	0.780442	1.600020	1.201488
H	1.415993	2.294097	1.007073
H	2.412799	−0.242664	0.705881
H	2.603276	0.485625	−0.896322
H	2.102446	−2.198723	−0.665737
H	1.271117	−1.290289	−1.927954
O	0.135437	−1.306468	1.222428
H	−0.594946	−2.358036	−0.473396
C	−1.841911	0.079003	0.594352
C	−2.711145	0.000168	−0.658982
H	−2.714896	−1.012186	−1.063507
H	−3.741106	0.271100	−0.429125
H	−2.359086	0.668684	−1.443120
H	−2.231906	−0.600233	1.352025
H	−1.875678	1.082817	1.023193

Table 17: M06-2X optimized geometry of *cis*-2-ethyl-2,3- epoxy-1-methylcyclopentan-1-ol (H-bonded) (in Å) with the TZ basis set

Atom	x	y	z
C	−1.845264	−0.014939	0.595361
C	−0.390291	−0.311371	0.348504
C	0.137800	−1.572330	−0.171262
C	1.431841	−1.325640	−0.908389
C	1.895804	0.028599	−0.354342
C	0.615089	0.798161	0.047337
C	0.138400	1.722117	−1.056327
H	−0.787127	2.219523	−0.768642
H	0.895837	2.484911	−1.232214
H	−0.025929	1.168573	−1.981850
O	0.812587	1.613761	1.189169
H	1.046131	1.030333	1.919450
H	2.495192	−0.126874	0.540711
H	2.496805	0.596182	−1.062339
H	2.163102	−2.117722	−0.749821
H	1.223097	−1.273371	−1.978898
O	0.221084	−1.271861	1.220738
H	−0.504716	−2.408504	−0.419264
H	−2.208070	−0.759030	1.304772
H	−1.920483	0.957570	1.087789
C	−2.707654	−0.046291	−0.664658
H	−2.388361	0.694174	−1.395854
H	−2.659170	−1.026342	−1.140011
H	−3.749886	0.155677	−0.420514

Table 18: M06-2X optimized geometry of *trans*-2-ethyl-2,3- epoxy-1-methylcyclopentan-1-ol (in Å) with the TZ basis set

Atom	x	y	z
C	0.557678	0.797773	−0.018912
C	1.889144	0.081243	−0.312846
C	1.505105	−1.321498	−0.806793
C	0.220107	−1.595969	−0.066691
C	−0.378980	−0.337859	0.383169
C	−1.854256	−0.129501	0.593710
H	−2.007212	0.767604	1.198773
C	−2.665042	−0.036645	−0.696588
H	−2.509549	−0.924932	−1.309987
H	−3.728676	0.034491	−0.471072
H	−2.372533	0.829445	−1.283449
H	−2.201312	−0.972227	1.193385
O	0.275409	−1.207928	1.305518
H	−0.379666	−2.474223	−0.272295
H	1.300878	−1.322792	−1.878276
H	2.276588	−2.063115	−0.602649
H	2.470871	0.644689	−1.042255
H	2.472166	0.007140	0.602783
C	0.683359	1.870592	1.046981
H	0.969558	1.423474	1.998672
H	1.449248	2.597773	0.767248
H	−0.261997	2.398333	1.170293
O	0.003741	1.333798	−1.219152
H	0.545624	2.078588	−1.494048

Table 19: M06-2X optimized geometry of *cis*-2,3- aziridine-1,2-dimethylcyclopentan-1-ol (not H-bonded) (in Å) with the TZ basis set

Atom	x	y	z
C	0.970972	0.007922	0.224872
C	−0.330233	0.607568	−0.292114
C	−1.106667	−0.479796	−0.922787
C	−0.344802	−1.782364	−0.806068
C	0.655747	−1.497394	0.325768
C	−0.361893	2.041166	−0.733923
N	−1.493866	0.040110	0.395380
C	2.108116	0.268013	−0.757671
O	1.268300	0.598801	1.479778
H	3.011133	−0.246699	−0.423492
H	1.859206	−0.101905	−1.753839
H	2.326163	1.332950	−0.821821
H	2.088898	0.222902	1.809489
H	0.182209	−1.668960	1.289216
H	1.556210	−2.108419	0.259471
H	−0.994368	−2.627692	−0.579929
H	0.168094	−1.993260	−1.746490
H	−1.750131	−0.286485	−1.770958
H	0.322840	2.221948	−1.561993
H	−1.364511	2.315934	−1.063599
H	−0.078573	2.693020	0.093687
H	−2.274758	0.679418	0.296589

Table 20: M06-2X optimized geometry of *cis*-2,3- aziridine-1,2-dimethylcyclopentan-1-ol (H-bonded) (in Å) with the TZ basis set

Atom	x	y	z
C	0.301646	−0.754350	−0.055112
C	−0.788641	0.302371	0.118797
C	0.000449	1.628529	0.234225
C	1.320376	1.414362	−0.519702
C	1.564369	−0.070957	−0.381727
C	−0.061511	−2.141016	−0.494167
C	−1.747358	0.316732	−1.056376
N	1.321342	−0.562632	0.987325
O	−1.565317	0.039107	1.273594
H	−0.798598	−2.569788	0.187093
H	−0.486780	−2.140691	−1.497411
H	0.818981	−2.784675	−0.502355
H	2.327438	−0.562840	−0.970491
H	1.217838	1.661360	−1.578232
H	2.133811	2.014894	−0.112966
H	−0.576015	2.470711	−0.144752
H	0.205211	1.810250	1.287364
H	−0.941766	−0.054298	2.003104
H	−2.476104	1.114407	−0.919356
H	−1.207422	0.489704	−1.988861
H	−2.283666	−0.628494	−1.127133
H	1.824145	−1.432629	1.125097

Table 21: M06-2X optimized geometry of *trans*-2,3- aziridine-1,2-dimethylcyclopentan-1-ol (in Å) with the TZ basis set

Atom	x	y	z
N	−1.175409	0.692722	1.102511
C	−1.568682	0.254708	−0.248791
C	−1.479835	−1.243694	−0.417532
C	−0.132047	−1.587410	0.236145
C	0.760781	−0.356286	0.002138
C	−0.222449	0.807332	−0.005288
C	1.864476	−0.208535	1.032407
O	1.297904	−0.377393	−1.321326
C	0.240448	2.155415	−0.469330
H	−2.314897	0.829832	−0.780297
H	−1.467648	−1.481144	−1.481773
H	−2.311987	−1.772627	0.046225
H	0.329951	−2.481831	−0.182690
H	−0.264288	−1.739357	1.305178
H	−0.605180	2.838404	−0.560320
H	0.949804	2.587438	0.239612
H	0.730645	2.073805	−1.437408
H	2.486666	−1.106110	1.061513
H	2.501113	0.641543	0.786486
H	1.432681	−0.058936	2.021648
H	1.933656	−1.095705	−1.375543
H	−1.570189	1.608221	1.289174

Table 22: M06-2X optimized geometry of *cis*-2,3- aziridine-1-ethyl-2-methylcyclopentan-1-ol (not H-bonded) (in Å) with the TZ basis set

Atom	x	y	z
N	−1.997241	−0.000316	0.685301
C	−0.919628	0.601231	−0.106554
C	0.443700	0.045340	0.294841
C	0.176421	−1.467184	0.430760
C	−0.906566	−1.787895	−0.611651
C	−1.716960	−0.511596	−0.662450
C	−1.048508	2.031917	−0.543372
C	1.478258	0.356467	−0.794704
C	2.884770	−0.137794	−0.478135
O	0.830431	0.653174	1.518012
H	2.931090	−1.221761	−0.376962
H	3.577316	0.144944	−1.269783
H	3.253023	0.309192	0.446015
H	1.132108	−0.069077	−1.740981
H	1.514589	1.438782	−0.923926
H	1.526720	0.127436	1.919022
H	−0.217668	−1.640182	1.429592
H	1.075149	−2.067614	0.301037
H	−1.505281	−2.653797	−0.329623
H	−0.468395	−1.985421	−1.591903
H	−2.440656	−0.342596	−1.448941
H	−0.480091	2.232760	−1.450526
H	−2.092919	2.271905	−0.746285
H	−0.688025	2.697207	0.242621
H	−2.803223	0.614322	0.659344

Table 23: M06-2X optimized geometry of *cis*-2,3- aziridine-1-ethyl-2-methylcyclopentan-1-ol (H-bonded) (in Å) with the TZ basis set

Atom	x	y	z
N	−1.961265	0.074979	0.724283
C	−0.926095	0.610798	−0.174227
C	0.433095	0.051550	0.252674
C	0.177977	−1.472303	0.363263
C	−0.981809	−1.791090	−0.590357
C	−1.775805	−0.506459	−0.617790
C	−1.042592	2.019761	−0.674651
C	1.516533	0.404101	−0.761017
C	2.903376	−0.101988	−0.383134
O	0.832342	0.616978	1.489722
H	2.957633	−1.189918	−0.386010
H	3.648329	0.264973	−1.088190
H	3.170866	0.247207	0.612387
H	1.215424	0.014015	−1.737910
H	1.551776	1.491461	−0.846760
H	0.096759	0.481271	2.097919
H	−0.120184	−1.688724	1.387045
H	1.070085	−2.056165	0.150692
H	−1.570852	−2.644973	−0.256021
H	−0.619477	−2.006966	−1.597452
H	−2.557104	−0.354295	−1.350813
H	−0.509449	2.154854	−1.615076
H	−2.088602	2.279584	−0.842293
H	−0.625604	2.714776	0.056386
H	−2.764217	0.694461	0.713151

Table 24: M06-2X optimized geometry of *trans*-2,3- aziridine-1-ethyl-2-methylcyclopentan-1-ol (in Å) with the TZ basis set

Atom	x	y	z
N	1.483849	0.103015	−1.302289
C	0.894161	0.640998	−0.072608
C	−0.461172	0.002293	0.206294
C	−0.212027	−1.499262	−0.029877
C	1.259520	−1.733638	0.348210
C	1.917244	−0.418646	0.005945
C	−1.574434	0.585011	−0.655071
C	−2.925407	−0.089627	−0.445889
O	−0.694438	0.254438	1.592792
C	1.125377	2.071170	0.313565
H	−0.899337	−2.108886	0.557085
H	−0.368149	−1.729783	−1.082157
H	1.361384	−1.913867	1.418917
H	1.693638	−2.577602	−0.187222
H	2.920197	−0.195143	0.344059
H	2.179962	2.328510	0.206100
H	0.546404	2.744803	−0.321326
H	0.828624	2.235430	1.347632
H	−2.880417	−1.152269	−0.686462
H	−3.268203	0.011465	0.586003
H	−3.688951	0.361037	−1.078280
H	−1.657693	1.650063	−0.427223
H	−1.266567	0.494499	−1.698625
H	−1.491474	−0.212869	1.854899
H	2.172918	0.761408	−1.649501

Table 25: M06-2X optimized geometry of *cis*-2,3- aziridine-1-ethyl-2-methylcyclopentan-1-ol (not H-bonded) (in Å) with the TZ basis set

Atom	x	y	z
N	−0.233528	−1.411638	−1.158806
C	0.387396	−0.338805	−0.370799
C	−0.628793	0.755045	−0.059803
C	−1.888186	−0.030133	0.357526
C	−1.368534	−1.312649	1.027065
C	−0.073877	−1.579368	0.289704
C	1.837535	0.024947	−0.601223
C	2.715408	−0.007068	0.648528
C	−0.147181	1.655997	1.071805
O	−0.818246	1.525448	−1.236321
H	−0.914371	2.399931	1.297013
H	0.041701	1.083592	1.981224
H	0.763782	2.181824	0.787948
H	−1.445964	2.227535	−1.045409
H	−2.433304	−0.281597	−0.548717
H	−2.535927	0.555794	1.010026
H	−2.075189	−2.137995	0.941987
H	−1.161783	−1.153026	2.087206
H	0.630425	−2.314483	0.656255
H	2.724587	−1.004258	1.089143
H	3.743565	0.258890	0.403280
H	2.364468	0.686633	1.410083
H	2.256920	−0.662262	−1.338949
H	1.867104	1.014160	−1.063657
H	0.496273	−1.921892	−1.643287

Table 26: M06-2X optimized geometry of *cis*-2,3- aziridine-1-ethyl-2-methylcyclopentan-1-ol (H-bonded) (in Å) with the TZ basis set

Atom	x	y	z
N	0.253964	−1.319030	1.236671
C	−0.406237	−0.338863	0.355266
C	0.610243	0.761327	0.036671
C	1.862603	−0.019425	−0.433115
C	1.359135	−1.359022	−0.988019
C	0.094691	−1.609217	−0.198630
C	−1.865658	−0.011293	0.556742
C	−2.697651	−0.005402	−0.724278
C	0.124297	1.726753	−1.027001
O	0.868837	1.541026	1.190782
H	−0.783072	2.236143	−0.704191
H	0.894054	2.478090	−1.198936
H	−0.071779	1.204081	−1.964106
H	1.094863	0.917167	1.890668
H	2.496127	−0.196019	0.433522
H	2.439584	0.551888	−1.158388
H	2.092394	−2.157384	−0.874969
H	1.107287	−1.281518	−2.047661
H	−0.586405	−2.400470	−0.484070
H	−2.294012	−0.744018	1.244967
H	−1.932860	0.955966	1.061213
H	−2.350861	0.745308	−1.430942
H	−2.648086	−0.975949	−1.218790
H	−3.743552	0.204437	−0.501638
H	−0.452865	−1.824575	1.758983

Table 27: M06-2X optimized geometry of *trans*-2,3- aziridine-1-ethyl-2-methylcyclopentan-1-ol (in Å) with the TZ basis set

Atom	x	y	z
N	0.241360	−1.180391	1.375337
C	0.057740	−1.600563	−0.023111
C	1.317295	−1.444776	−0.842158
C	1.845908	−0.070776	−0.402758
C	0.596624	0.764178	−0.062256
C	−0.412555	−0.273551	0.421666
C	0.870906	1.853265	0.958360
O	0.035469	1.319321	−1.252731
C	−1.871257	0.075309	0.591605
C	−2.671763	0.131651	−0.707712
H	−1.952094	1.028475	1.120501
H	−2.588556	−0.812354	−1.247506
H	−3.726664	0.307414	−0.496647
H	−2.306560	0.921513	−1.356967
H	−2.317213	−0.675793	1.249500
H	−0.647319	−2.393864	−0.234478
H	1.055035	−1.441489	−1.900828
H	2.039553	−2.241541	−0.666385
H	2.436657	0.424912	−1.173591
H	2.463405	−0.176879	0.486270
H	1.181105	1.408158	1.903060
H	1.669885	2.511947	0.609604
H	−0.021961	2.457008	1.121058
H	0.646427	1.980586	−1.589040
H	−0.468555	−1.614756	1.954320

Table 28: M06-2X optimized geometry of *cis*-2,3- phosphirane-1,2-dimethylcyclopentan-1-ol (not H-bonded) (in Å) with the TZ basis set

Atom	x	y	z
C	0.980181	−0.011698	0.249408
C	−0.343303	0.591596	−0.230557
C	−1.116803	−0.507470	−0.883629
C	−0.292046	−1.784831	−0.835451
C	0.701997	−1.526049	0.302287
C	−0.320284	1.995780	−0.775894
P	−1.814127	0.013846	0.772373
C	2.104633	0.294480	−0.737480
O	1.298743	0.534681	1.519816
H	3.015084	−0.224200	−0.429463
H	1.850339	−0.042230	−1.743611
H	2.310614	1.362864	−0.766938
H	2.182524	0.247266	1.764937
H	0.247417	−1.752642	1.267343
H	1.614865	−2.115033	0.214319
H	−0.894372	−2.677244	−0.670951
H	0.235426	−1.911326	−1.784110
H	−1.710323	−0.297344	−1.762935
H	0.303031	2.059231	−1.670571
H	−1.322293	2.325062	−1.045191
H	0.077462	2.685736	−0.030965
H	−2.690681	0.976302	0.216826

Table 29: M06-2X optimized geometry of *cis*-2,3- phosphirane-1,2-dimethylcyclopentan-1-ol (H-bonded) (in Å) with the TZ basis set

Atom	x	y	z
C	0.969753	−0.000071	0.241285
C	−0.341864	0.599669	−0.294699
C	−1.106794	−0.501494	−0.938017
C	−0.288177	−1.781752	−0.857913
C	0.687521	−1.515642	0.292767
C	−0.306928	1.997521	−0.853517
P	−1.839123	0.036900	0.699380
C	2.128966	0.300259	−0.698147
O	1.350036	0.535595	1.494112
H	3.023632	−0.198334	−0.327618
H	1.916474	−0.057064	−1.706506
H	2.324483	1.370111	−0.734866
H	0.587864	0.481021	2.080714
H	0.218575	−1.752984	1.249623
H	1.604387	−2.098251	0.226220
H	−0.898085	−2.669673	−0.698304
H	0.254429	−1.916047	−1.796640
H	−1.697474	−0.305238	−1.822644
H	0.333088	2.043837	−1.737015
H	−1.302083	2.330212	−1.143572
H	0.085998	2.694635	−0.112539
H	−2.700657	1.007965	0.140067

Table 30: M06-2X optimized geometry of *trans*-2,3- phosphirane-1,2-dimethylcyclopentan-1-ol (in Å) with the TZ basis set

Atom	x	y	z
C	−0.301998	0.610113	−0.272068
C	1.028557	−0.009432	0.168549
C	0.700947	−1.498400	0.357114
C	−0.346401	−1.796336	−0.722373
C	−1.137559	−0.502491	−0.813277
C	1.657900	0.642476	1.385620
O	1.884776	0.143408	−0.968390
P	−1.743863	0.152930	0.831970
C	−0.250625	1.967135	−0.923832
H	−1.768024	−0.328637	−1.674290
H	0.147089	−1.972856	−1.679444
H	−0.961269	−2.663478	−0.486547
H	1.598942	−2.109725	0.266732
H	0.286391	−1.665524	1.352832
H	−1.232426	2.245985	−1.304352
H	0.063803	2.734929	−0.215679
H	0.457487	1.954358	−1.751916
H	2.595363	0.143297	1.642060
H	1.874828	1.690794	1.181313
H	0.991969	0.580109	2.245498
H	2.746265	−0.219161	−0.744240
H	−2.607859	1.117057	0.261481

Table 31: M06-2X optimized geometry of *cis*-2,3- phosphirane-1-ethyl-2-methylcyclopentan-1-ol (not H-bonded) (in Å) with the TZ basis set

Atom	x	y	z
C	0.406318	−1.476309	0.330985
C	0.639096	0.047509	0.290452
C	−0.680081	0.599536	−0.267754
C	−1.376417	−0.527092	−0.959401
C	−0.508844	−1.771353	−0.862315
C	1.803206	0.422049	−0.640429
C	3.170074	−0.069325	−0.175417
O	0.853350	0.604250	1.578394
C	−0.695801	2.003429	−0.817095
P	−2.180571	−0.034783	0.655986
H	3.220338	−1.154722	−0.096360
H	3.942305	0.241214	−0.877965
H	3.436698	0.357389	0.792833
H	1.589664	0.037661	−1.641618
H	1.835783	1.508736	−0.715853
H	1.649743	0.218263	1.952199
H	−0.101720	−1.712091	1.266937
H	1.331383	−2.049039	0.305621
H	−1.084291	−2.686799	−0.730880
H	0.077809	−1.877277	−1.778249
H	−1.927217	−0.338753	−1.870921
H	−0.052887	2.091819	−1.695183
H	−1.702734	2.289805	−1.115235
H	−0.349078	2.711660	−0.063801
H	−3.063356	0.893630	0.054674

Table 32: M06-2X optimized geometry of *cis*-2,3- phosphirane-1-ethyl-2-methylcyclopentan-1-ol (H-bonded) (in Å) with the TZ basis set

Atom	x	y	z
C	−0.365506	1.473232	0.738952
C	−0.569109	−0.057974	0.673385
C	0.480742	−0.526909	−0.349725
C	0.961245	0.661516	−1.102266
C	0.215513	1.896154	−0.617866
O	−0.405684	−0.681667	1.935475
C	−1.991386	−0.438985	0.256555
C	−2.488316	0.186221	−1.040490
C	0.306552	−1.882152	−0.983771
P	2.219190	0.030009	0.129018
H	−1.290483	1.991660	0.988786
H	0.346936	1.686496	1.537494
H	−0.577233	2.133772	−1.327682
H	0.859905	2.770716	−0.538169
H	1.215232	0.574621	−2.150362
H	1.136906	−2.113125	−1.649034
H	0.256142	−2.656378	−0.217347
H	−0.614494	−1.925783	−1.568733
H	0.524669	−0.618732	2.177086
H	−1.803628	0.004910	−1.870974
H	−3.457739	−0.228887	−1.312641
H	−2.612323	1.264452	−0.941335
H	−2.041303	−1.527022	0.201010
H	−2.641397	−0.144721	1.082553
H	2.859935	−0.854423	−0.768287

Table 33: M06-2X optimized geometry of *trans*-2,3- phosphirane-1-ethyl-2-methylcyclopentan-1-ol (in Å) with the TZ basis set

Atom	x	y	z
P	1.645722	0.077625	−1.764594
C	0.886648	0.630195	−0.143863
C	−0.473132	−0.013960	0.150446
C	−0.223074	−1.517825	−0.052992
C	1.224531	−1.729506	0.407830
C	1.917667	−0.437432	0.013601
C	−1.615878	0.552796	−0.684750
C	−2.971376	−0.070735	−0.371425
O	−0.691498	0.242910	1.541335
C	1.109133	2.034941	0.353394
H	−0.937376	−2.120884	0.507289
H	−0.334274	−1.773734	−1.108706
H	1.256628	−1.826419	1.494301
H	1.679932	−2.616650	−0.029609
H	2.874634	−0.196084	0.455311
H	2.147308	2.330901	0.207497
H	0.480124	2.749812	−0.178349
H	0.872428	2.093823	1.415459
H	−2.963215	−1.148434	−0.539700
H	−3.269019	0.112598	0.662650
H	−3.748979	0.355119	−1.003715
H	−1.658836	1.630209	−0.512098
H	−1.374355	0.403802	−1.738651
H	−1.480115	−0.233217	1.814408
H	2.641383	1.082697	−1.750097

Table 34: M06-2X optimized geometry of *cis*-2-ethyl-2,3-phosphirane–methylcyclopentan-1-ol (not H-bonded) (in Å) with the TZ basis set

Atom	x	y	z
C	−0.317919	1.018819	−0.311864
C	0.328843	−0.368214	−0.198181
C	−0.290587	−1.044766	0.984005
C	−1.278061	−0.088154	1.634858
C	−1.616233	0.895540	0.509225
C	1.798087	−0.508800	−0.562803
C	2.767403	−0.304103	0.601905
P	−0.873667	−1.735628	−0.650258
C	0.570077	2.114710	0.273270
O	−0.554598	1.291056	−1.684974
H	1.518368	2.177868	−0.257945
H	0.060463	3.076754	0.180711
H	0.766360	1.943823	1.331872
H	−0.843939	2.203413	−1.771924
H	−2.391090	0.484695	−0.138724
H	−1.962492	1.861753	0.876035
H	−2.159736	−0.589747	2.031686
H	−0.788892	0.429581	2.463849
H	0.304766	−1.690175	1.614560
H	1.979766	−1.496541	−0.983556
H	2.008666	0.199453	−1.367210
H	2.677091	0.683388	1.049577
H	2.590174	−1.040806	1.385906
H	3.796776	−0.423967	0.263728
H	0.131479	−2.730866	−0.703635

Table 35: M06-2X optimized geometry of *cis*-2-ethyl-2,3-phosphirane-methylcyclopentan-1-ol (H-bonded) (in Å) with the TZ basis set

Atom	x	y	z
C	−0.365889	0.999877	−0.267727
C	0.302390	−0.384251	−0.142655
C	−0.291710	−1.058748	1.043967
C	−1.271282	−0.105055	1.712171
C	−1.639666	0.874762	0.594049
C	1.762516	−0.524025	−0.538028
C	2.750165	−0.280097	0.603423
P	−0.912564	−1.765504	−0.572097
C	0.519566	2.117772	0.264711
O	−0.635248	1.352553	−1.611431
H	1.456847	2.170681	−0.286333
H	−0.006083	3.062972	0.133751
H	0.733391	1.978063	1.324187
H	−1.078791	0.605690	−2.028028
H	−2.443242	0.464852	−0.020390
H	−1.972384	1.844015	0.960814
H	−2.138650	−0.611124	2.133521
H	−0.761326	0.415639	2.526376
H	0.310405	−1.712100	1.660225
H	1.945303	−1.520665	−0.937362
H	1.950354	0.168664	−1.361655
H	2.652378	0.716425	1.028216
H	2.597542	−1.001147	1.406924
H	3.774373	−0.393635	0.248269
H	0.091634	−2.755101	−0.668920

Table 36: M06-2X optimized geometry of *trans*-2-ethyl-2,3-phosphirane-methylcyclopentan-1-ol (in Å) with the TZ basis set

Atom	x	y	z
C	−0.368458	−0.295732	0.248356
C	0.460238	0.996151	0.192808
C	1.684435	0.623009	−0.658807
C	1.154904	−0.400944	−1.667820
C	0.100966	−1.158377	−0.878948
C	0.820245	1.575399	1.549339
O	−0.354484	1.927221	−0.527607
P	0.657987	−1.766781	0.797895
C	−1.835557	−0.176321	0.629458
C	−2.763628	0.085322	−0.556123
H	−1.934248	0.633828	1.354640
H	−2.733615	−0.744219	−1.263300
H	−3.793780	0.189088	−0.214383
H	−2.475793	0.992473	−1.080481
H	−2.159351	−1.083536	1.139140
H	−0.606705	−1.777987	−1.411388
H	0.667539	0.112184	−2.498984
H	1.938206	−1.041796	−2.069745
H	2.115040	1.508039	−1.127060
H	2.454000	0.175078	−0.027680
H	1.413024	0.868574	2.128144
H	1.404547	2.490580	1.425640
H	−0.081757	1.820999	2.109530
H	0.102998	2.772624	−0.544706
H	−0.463590	−2.606887	0.989258

Table 37: M06-2X optimized geometry of *cis*-2,3-thiirane- 1,2-dimethylcyclopentan-1-ol (not H-bonded) (in Å) with the TZ basis set

Atom	x	y	z
C	−0.266651	0.791570	0.050147
C	−1.575606	0.194592	−0.305768
C	−1.415763	−1.308267	−0.448306
C	−0.117836	−1.613698	0.310273
C	0.748377	−0.350682	0.142920
C	1.569606	−0.425478	−1.143419
H	2.227156	−1.296260	−1.106797
H	0.926747	−0.528207	−2.019371
H	2.184357	0.465017	−1.260182
O	1.602730	−0.105898	1.245161
H	2.292671	−0.774774	1.254917
H	−0.318388	−1.748880	1.370295
H	0.386528	−2.503980	−0.064845
H	−2.270976	−1.861809	−0.064684
H	−1.312917	−1.548573	−1.509740
S	−1.490458	0.817439	1.398326
H	−2.254880	0.727425	−0.958124
C	0.191361	2.127853	−0.468473
H	0.523551	2.050294	−1.505067
H	−0.620963	2.849310	−0.417444
H	1.021822	2.496374	0.133770

Table 38: M06-2X optimized geometry of *cis*-2,3-thiirane- 1,2-dimethylcyclopentan-1-ol (H-bonded) (in Å) with the TZ basis set

Atom	x	y	z
C	−0.010287	−0.026340	−0.018720
C	1.532685	−0.040610	0.038545
C	1.957812	1.431143	0.046487
C	0.859316	2.136071	−0.725404
C	−0.349932	1.290459	−0.739067
C	−1.744307	1.843250	−0.638131
H	−2.467788	1.095053	−0.962692
H	−1.974219	2.117636	0.392624
H	−1.849852	2.725215	−1.266011
S	0.475912	1.438318	−2.367923
H	0.769221	3.213103	−0.672661
H	1.966433	1.828883	1.064657
H	2.945919	1.592950	−0.380405
H	1.891903	−0.590004	0.906590
H	1.912998	−0.537863	−0.851663
O	−0.549194	−1.153291	−0.675599
H	−0.228172	−1.135009	−1.584420
C	−0.608301	−0.033037	1.380434
H	−0.301686	−0.944422	1.891336
H	−0.262555	0.827046	1.955943
H	−1.695428	−0.015509	1.333293

Table 39: M06-2X optimized geometry of *trans*-2,3-thiirane- 1,2-dimethylcyclopentan-1-ol (in Å) with the TZ basis set

Atom	x	y	z
C	0.721591	−0.397856	0.058979
C	−0.219542	−1.593338	0.276318
C	−1.539890	−1.174462	−0.382154
C	−1.577453	0.328294	−0.193776
C	−0.206499	0.823021	0.064587
S	−1.310244	0.918067	1.510372
H	−2.266785	0.927763	−0.772952
H	−1.510706	−1.377922	−1.454410
H	−2.406468	−1.679873	0.039787
H	0.203613	−2.496254	−0.163980
H	−0.360043	−1.769459	1.340540
C	0.314312	2.117142	−0.496524
H	−0.476209	2.864992	−0.506556
H	1.134666	2.499819	0.111084
H	0.681129	1.958288	−1.509552
C	1.860880	−0.321927	1.056263
H	2.462568	−1.232624	1.013196
H	2.507178	0.525579	0.828889
H	1.474428	−0.215720	2.069109
O	1.228495	−0.424993	−1.278834
H	1.825699	−1.173877	−1.357264

Table 40: M06-2X optimized geometry of *cis*-2,3-thiirane- 1-ethyl-2-methylcyclopentan-1-ol (not H-bonded) (in Å) with the TZ basis set

Atom	x	y	z
C	−0.943194	0.592819	−0.037696
C	−1.742075	−0.550538	−0.538229
C	−0.896463	−1.808488	−0.481496
C	0.213922	−1.460352	0.517658
C	0.438992	0.056592	0.357252
C	1.447102	0.368164	−0.761267
C	2.871469	−0.087676	−0.464080
H	2.944242	−1.164793	−0.317730
H	3.532624	0.173988	−1.289033
H	3.263492	0.407215	0.425440
H	1.094499	−0.086294	−1.691694
H	1.457219	1.447043	−0.914578
O	0.837832	0.693453	1.557885
H	1.636536	0.269748	1.882099
H	−0.122573	−1.644851	1.535227
H	1.119782	−2.038608	0.346598
H	−1.466150	−2.688877	−0.189171
H	−0.481476	−1.995377	−1.475440
S	−2.289460	0.075823	1.076044
H	−2.441741	−0.408489	−1.351449
C	−1.059712	1.976808	−0.619535
H	−0.556094	2.040274	−1.585176
H	−2.107178	2.234310	−0.758972
H	−0.610067	2.706002	0.054571

Table 41: M06-2X optimized geometry of *cis*-2,3-thiirane- 1-ethyl-2-methylcyclopentan-1-ol (H-bonded) (in Å) with the TZ basis set

Atom	x	y	z
C	0.405238	0.098892	0.698443
C	−0.696157	0.622419	−0.241158
C	−1.380355	−0.537409	−0.844216
C	−0.719132	−1.820407	−0.381353
C	0.086797	−1.406794	0.857834
H	0.996165	−1.993189	0.982735
H	−0.511466	−1.536968	1.757372
H	−0.065809	−2.176897	−1.179624
H	−1.442362	−2.606107	−0.169844
S	−2.379269	0.314892	0.422932
H	−1.786392	−0.470758	−1.845291
C	−0.499963	1.934406	−0.949195
H	−1.392742	2.198889	−1.511754
H	−0.301083	2.722275	−0.222384
H	0.345863	1.883838	−1.636688
O	0.411488	0.786604	1.932965
H	−0.479994	0.729841	2.295594
C	1.798219	0.341181	0.115690
C	2.086306	−0.373300	−1.198855
H	1.337993	−0.148386	−1.961184
H	3.056451	−0.070491	−1.589867
H	2.111071	−1.454997	−1.067521
H	1.933028	1.417909	0.005388
H	2.509906	0.018608	0.877490

Table 42: M06-2X optimized geometry of *trans*-2,3-thiirane- 1-ethyl-2-methylcyclopentan-1-ol (in Å) with the TZ basis set

Atom	x	y	z
C	−0.466466	−0.007869	0.137146
C	0.891424	0.650555	−0.138306
C	1.919242	−0.410393	−0.049514
C	1.251220	−1.724776	0.294976
C	−0.216246	−1.507926	−0.096105
H	−0.898635	−2.114466	0.499119
H	−0.375508	−1.753851	−1.144394
H	1.338089	−1.873273	1.373173
H	1.703136	−2.578996	−0.205649
S	1.632434	0.202973	−1.741541
H	2.897296	−0.181034	0.351581
C	1.121697	2.049201	0.364175
H	2.154380	2.343489	0.186947
H	0.474539	2.758864	−0.151311
H	0.905327	2.097065	1.430517
C	−1.606113	0.574290	−0.688726
C	−2.954438	−0.078219	−0.405397
H	−2.930616	−1.148356	−0.614502
H	−3.258110	0.061122	0.633941
H	−3.735185	0.361523	−1.023958
H	−1.665089	1.644672	−0.480788
H	−1.351110	0.458963	−1.743518
O	−0.673880	0.231510	1.532417
H	−1.458996	−0.249566	1.806477

Table 43: M06-2X optimized geometry of *cis*-2-ethyl-2,3- thiirane-1-methylcyclopentan-1-ol (not H-bonded) (in Å) with the TZ basis set

Atom	x	y	z
C	−0.470402	−0.222336	0.057678
C	0.005181	−1.392204	−0.719834
C	1.451705	−1.170942	−1.123441
C	1.946811	−0.100665	−0.143308
C	0.705536	0.757039	0.167501
C	0.561778	1.879283	−0.860275
H	1.449668	2.513947	−0.830233
H	0.469576	1.481602	−1.872382
H	−0.308192	2.495796	−0.643265
O	0.714189	1.302829	1.474304
H	1.396809	1.977593	1.521412
H	2.284814	−0.558405	0.783035
H	2.760314	0.497942	−0.552746
H	2.042894	−2.084394	−1.088019
H	1.474534	−0.799496	−2.151349
S	−0.253205	−1.739177	1.043177
H	−0.664144	−1.910391	−1.393991
C	−1.858992	0.367574	−0.067940
C	−3.007873	−0.629136	−0.024740
H	−3.078233	−1.106849	0.949489
H	−3.949278	−0.121486	−0.231645
H	−2.881514	−1.416143	−0.769285
H	−1.976476	1.107234	0.727406
H	−1.898908	0.915402	−1.013190

Table 44: M06-2X optimized geometry of *cis*-2-ethyl-2,3- thiirane-1-methylcyclopentan-1-ol (H-bonded) (in Å) with the TZ basis set

Atom	x	y	z
C	−1.855618	0.006910	0.574472
C	−0.380790	−0.316772	0.440674
C	0.123092	−1.577106	−0.140461
C	1.377541	−1.293610	−0.944116
C	1.886962	0.036129	−0.380180
C	0.627776	0.797113	0.091852
C	0.105023	1.702207	−1.013445
H	−0.815868	2.196959	−0.709643
H	0.854235	2.467403	−1.211742
H	−0.073404	1.139517	−1.930069
O	0.875637	1.649076	1.189937
H	1.150925	1.091973	1.927072
H	2.534033	−0.140712	0.476431
H	2.449230	0.616786	−1.109042
H	2.111754	−2.094565	−0.879035
H	1.091109	−1.188315	−1.994040
S	0.316437	−1.491651	1.670908
H	−0.566558	−2.341077	−0.473982
H	−2.297222	−0.691493	1.283133
H	−1.943414	1.000412	1.019910
C	−2.638778	−0.068885	−0.736718
H	−2.289602	0.649255	−1.474732
H	−2.562537	−1.064469	−1.174832
H	−3.694145	0.130221	−0.552906

Table 45: M06-2X optimized geometry of *trans*-2-ethyl-2,3- thiirane-1-methylcyclopentan-1-ol (in Å) with the TZ basis set

Atom	x	y	z
C	0.676163	0.892131	0.113874
C	1.968099	0.123477	−0.203446
C	1.516922	−1.036892	−1.097957
C	0.122551	−1.356739	−0.599301
C	−0.418696	−0.186748	0.130316
C	−1.855628	0.282257	0.059923
H	−1.903875	1.047107	−0.715742
C	−2.877655	−0.810024	−0.221411
H	−2.817014	−1.607557	0.517659
H	−3.884774	−0.396500	−0.197350
H	−2.726427	−1.246631	−1.208745
H	−2.097076	0.776602	1.003833
S	−0.025430	−1.610370	1.200699
H	−0.528573	−1.983809	−1.191975
H	1.436158	−0.707506	−2.135706
H	2.188739	−1.892318	−1.059940
H	2.689678	0.778110	−0.692395
H	2.417075	−0.250317	0.714021
C	0.750061	1.726028	1.377936
H	0.928592	1.089841	2.243970
H	1.568122	2.446586	1.308111
H	−0.178970	2.276859	1.523456
O	0.328838	1.712309	−1.006657
H	0.983369	2.412360	−1.077651

Table 46: M06-2X/cc-pVTZ Frequencies and IR Intensities of cis-2,3-epoxy-1,2-dimethylcyclopentan-1-ol (not H-bonded)

Frequency	IR Intensity
130.2	0.7
186.7	1.3
213.5	0.8
228.8	78.7
242.1	21.9
267.6	0.4
310.5	0.6
362.7	2.0
380.6	3.0
422.6	1.7
482.3	13.4
534.7	13.5
587.7	5.9
661.5	1.7
691.4	3.9
813.3	3.5
853.7	6.7
894.6	15.8
944.1	6.5
949.4	2.4
955.5	9.8
994.9	1.5
1011.5	6.2
1046.2	7.5
1088.0	13.9
1102.1	62.0
1127.5	12.2
1169.4	17.1
1212.0	12.5
1227.1	37.9
1242.5	26.5
1261.0	27.6
1306.3	7.0
1333.5	4.0
1343.7	11.8
1402.4	18.5
1405.4	6.1
1412.6	8.5
1474.4	4.3
1484.2	7.2
1490.4	6.6
1496.1	10.6
1499.9	3.2
1507.6	8.4
1522.4	5.5
3055.0	12.1
3070.6	15.3
3072.5	21.2
3092.4	25.8
3118.7	16.5
3122.5	22.8
3139.4	12.4
3147.7	10.8
3152.2	11.2
3156.7	10.8
3178.1	20.1
3872.3	32.3

Table 47: M06-2X/cc-pVTZ Frequencies and IR Intensities of cis-2,3-epoxy-1,2-dimethylcyclopentan-1-ol (H-bonded)

Frequency	IR Intensity
112.7	3.0
198.3	0.1
213.9	1.1
232.7	1.3
266.6	0.8
305.9	0.6
354.6	3.9
373.1	2.9
417.4	29.4
441.1	49.9
483.1	33.2
539.4	14.6
594.6	5.2
664.7	2.6
686.8	4.3
810.2	0.6
846.3	5.7
896.4	13.7
944.0	8.3
951.2	4.7
963.1	5.4
995.3	14.1
1014.5	8.6
1046.7	28.2
1084.1	13.5
1117.4	6.8
1126.0	24.0
1170.9	37.1
1183.9	3.0
1228.1	48.9
1246.8	11.2
1295.8	19.2
1314.7	1.7
1336.5	3.0
1352.3	0.7
1389.9	40.7
1410.1	11.6
1429.0	34.3
1476.6	2.9
1482.9	7.7
1487.5	9.9
1494.8	10.0
1500.1	3.1
1509.5	4.0
1523.5	5.0
3066.1	8.4
3068.5	15.1
3074.5	22.4
3098.1	15.3
3120.0	19.0
3137.2	11.9
3141.9	11.5
3145.8	14.6
3151.3	20.2
3156.4	8.1
3182.4	19.1
3843.0	32.8

Table 48: M06-2X/cc-pVTZ Frequencies and IR Intensities of trans-2,3-epoxy-1,2-dimethylcyclopentan-1-ol

Frequency	IR Intensity
118.5	2.1
183.0	0.4
223.6	4.4
242.5	2.1
264.0	0.2
271.0	28.1
282.6	71.8
353.5	2.0
381.6	7.6
425.5	1.2
481.3	14.5
513.9	1.9
600.0	2.4
654.6	1.2
701.7	1.9
814.6	3.6
855.2	4.5
888.5	8.2
937.2	3.7
952.1	20.9
960.2	13.1
992.7	2.2
1016.7	6.0
1042.3	6.2
1091.6	5.6
1096.4	82.5
1125.4	12.5
1165.5	1.4
1209.5	5.9
1231.1	68.3
1241.5	15.3
1260.0	3.9
1304.4	5.6
1339.6	5.7
1352.3	3.5
1402.3	3.2
1405.7	17.5
1416.9	23.6
1473.0	2.5
1483.4	5.4
1487.5	14.2
1491.4	5.2
1495.6	6.1
1506.2	2.0
1522.0	7.2
3053.3	16.2
3068.9	16.8
3082.8	24.6
3093.6	25.0
3123.0	14.5
3125.9	12.1
3137.7	11.7
3143.5	6.4
3147.8	23.2
3164.0	5.9
3186.1	19.6
3874.1	32.6

Table 49: M06-2X/cc-pVTZ Frequencies and IR Intensities of cis-2,3-epoxy-1-ethyl-2-methylcyclopentan-1-ol (not H-bonded)

Frequency	IR Intensity
77.2	0.5
124.9	1.1
170.6	12.8
196.3	72.3
204.3	4.1
224.6	6.1
274.7	18.9
286.2	3.0
322.3	1.5
356.6	1.3
392.2	3.1
423.9	4.3
484.4	10.2
546.0	12.1
591.4	4.8
662.6	1.8
708.2	2.4
794.0	1.0
804.5	5.3
854.5	7.0
894.8	15.3
945.3	1.3
951.3	16.2
981.9	7.0
1004.2	17.4
1031.0	0.7
1038.2	13.6
1052.8	19.7
1093.1	16.5
1105.5	20.7
1125.2	16.9
1165.7	15.2
1195.9	9.1
1235.0	56.0
1239.2	6.9
1249.3	22.6
1301.7	1.1
1318.9	2.6
1334.4	4.7
1346.3	10.7
1367.0	4.9
1400.3	9.2
1409.8	5.5
1422.9	6.2
1475.6	1.1
1483.5	11.6
1484.5	7.7
1495.6	9.6
1498.5	3.8
1506.6	6.3
1517.0	8.6
1520.2	6.7
3058.7	10.6
3067.2	19.6
3071.7	15.4
3073.9	24.1
3103.3	20.2
3107.0	9.3
3119.7	15.4
3134.9	23.9
3139.3	24.6
3143.4	11.8
3155.5	13.0
3157.6	12.3
3180.1	21.3
3881.1	31.9

Table 50: M06-2X/cc-pVTZ Frequencies and IR Intensities of cis-2,3-epoxy-1-ethyl-2-methylcyclopentan-1-ol (H-bonded)

Frequency	IR Intensity
130.0	1.6
141.6	1.3
199.1	0.3
224.1	0.3
242.5	0.6
257.5	1.7
263.1	1.5
310.7	0.9
373.2	4.8
402.0	4.7
437.1	35.2
458.0	27.0
485.3	47.9
533.2	10.4
616.2	5.1
650.7	0.8
690.9	5.7
790.1	0.4
821.3	0.9
842.8	5.4
895.2	12.1
941.0	3.3
947.2	23.6
976.9	14.0
1006.2	1.1
1025.6	12.2
1054.0	23.1
1075.0	17.4
1087.8	19.0
1122.8	3.8
1130.5	33.3
1171.0	20.4
1184.3	9.8
1203.5	37.4
1246.4	1.2
1271.9	3.6
1294.6	9.5
1326.3	2.7
1336.1	6.9
1345.4	2.8
1383.3	20.5
1409.8	11.9
1411.8	30.0
1420.5	7.2
1475.8	3.3
1482.5	11.0
1493.7	12.4
1497.4	3.6
1501.2	2.1
1507.5	10.7
1517.7	5.7
1522.7	7.2
3062.6	9.8
3066.9	11.2
3072.2	23.1
3080.5	26.1
3096.9	17.0
3105.3	2.4
3119.6	15.0
3130.9	32.6
3135.5	8.7
3140.3	34.2
3143.2	11.7
3155.3	9.6
3178.6	18.6
3840.7	33.4

Table 51: Frequencies and IR Intensities of trans-2,3-epoxy-1-ethyl-2-methylcyclopentan-1-ol.

Frequency	IR Intensity
97.8	0.2
117.7	2.3
169.5	1.1
192.0	1.0
239.3	11.7
246.1	4.5
269.5	64.4
276.5	28.9
303.1	4.2
362.5	2.0
404.1	2.9
437.0	3.6
489.5	11.5
521.8	2.3
588.1	1.7
677.8	0.6
704.6	2.2
789.4	4.1
826.5	2.9
851.3	6.7
895.4	9.4
946.2	5.1
967.7	20.0
983.6	7.4
991.7	23.0
1026.1	26.3
1033.8	2.5
1048.2	2.4
1092.9	4.6
1101.1	38.4
1126.4	17.8
1167.1	1.9
1204.8	2.6
1226.8	66.9
1240.4	4.6
1249.1	12.2
1302.7	5.6
1319.9	5.1
1336.8	7.6
1351.2	2.3
1361.6	3.3
1405.0	15.9
1410.5	3.0
1428.6	17.3
1474.3	2.2
1482.7	8.5
1486.2	5.2
1492.3	3.7
1494.0	7.1
1505.6	8.1
1513.9	5.7
1522.8	8.0
3057.0	13.1
3064.4	16.3
3070.1	21.8
3081.9	26.6
3090.9	20.0
3102.3	2.3
3124.2	8.1
3125.5	31.2
3138.5	6.5
3139.5	29.0
3141.8	19.9
3163.6	6.0
3185.8	20.7
3880.2	29.3

Table 52: M06-2X/cc-pVTZ Frequencies and IR Intensities of cis-2-ethyl-2,3- epoxy-1-methylcyclopentan-1-ol (not H-bonded)

Frequency	IR Intensity
84.6	0.2
116.0	1.0
185.8	0.5
199.4	5.1
221.0	83.7
233.6	8.6
276.4	2.7
303.6	1.6
319.0	1.0
364.0	1.8
406.2	3.1
442.7	3.3
487.5	13.5
556.1	4.1
599.6	15.4
650.7	2.0
697.1	2.0
775.3	2.6
815.9	3.9
870.1	11.0
922.7	5.0
947.6	2.1
957.6	15.3
968.4	8.2
981.7	10.7
1016.4	7.0
1026.4	19.3
1058.6	4.8
1090.7	13.2
1112.9	37.4
1123.3	5.4
1168.0	25.1
1211.5	10.7
1219.0	39.5
1239.6	13.0
1244.8	30.8
1293.9	8.9
1310.5	1.2
1332.5	6.7
1342.1	12.5
1371.8	1.6
1405.4	18.2
1411.3	0.7
1415.3	7.4
1478.4	6.4
1484.2	0.5
1494.3	8.6
1498.2	10.2
1500.3	7.4
1504.2	1.2
1510.0	11.1
1515.8	6.8
3053.4	11.6
3068.6	17.2
3073.7	27.4
3074.1	10.7
3092.6	25.3
3115.7	0.5
3119.0	16.3
3121.3	32.4
3135.8	24.6
3143.1	17.4
3148.3	19.1
3152.5	10.7
3179.4	20.1
3872.4	32.4

Table 53: Frequencies and IR Intensities of cis-2-ethyl-2,3-epoxy-1-methylcyclopentan-1-ol (H-bonded)

Frequency	IR Intensity
97.5	1.4
104.3	1.5
172.2	0.6
201.6	0.2
229.0	2.0
274.2	0.3
300.4	1.7
318.1	0.7
359.5	2.2
398.9	16.1
422.9	62.8
443.9	11.3
486.2	30.6
558.8	5.9
605.2	11.9
654.4	3.9
693.4	2.9
779.3	2.8
815.4	1.3
864.9	7.8
925.8	1.4
949.4	2.3
957.6	19.3
968.5	4.5
980.2	15.3
1018.6	25.8
1027.9	14.5
1059.8	22.9
1091.6	5.9
1113.7	8.0
1126.6	12.1
1173.3	31.3
1182.2	3.3
1226.1	47.0
1241.3	15.3
1268.0	1.4
1300.4	15.2
1316.8	8.6
1336.2	2.2
1351.3	0.2
1373.3	6.2
1393.5	30.1
1415.1	6.1
1428.3	36.8
1480.5	9.1
1481.8	3.1
1491.2	4.0
1498.9	9.5
1500.5	8.4
1506.3	0.8
1511.9	6.6
1517.3	4.9
3064.5	7.6
3067.1	10.4
3071.8	23.3
3073.7	24.2
3099.1	15.2
3111.6	2.5
3119.6	19.8
3137.1	23.2
3141.5	17.7
3144.6	14.8
3147.7	33.6
3151.7	6.6
3178.3	19.1
3843.0	33.6

Table 54: Frequencies and IR Intensities of trans-2-ethyl-2,3-epoxy-1-methylcyclopentan-1-ol

Frequency	IR Intensity
42.0	0.0
120.9	1.7
173.1	1.4
219.7	1.8
229.4	3.8
260.0	8.3
261.8	80.6
275.5	6.0
307.4	1.9
377.2	7.5
396.5	3.4
433.2	1.6
483.1	14.4
554.8	5.3
596.0	2.5
647.0	2.8
705.3	0.2
778.9	3.3
819.4	1.8
872.6	5.7
910.5	1.8
940.9	4.0
958.2	7.2
971.1	29.3
999.2	3.1
1008.6	20.2
1028.9	15.8
1064.6	12.0
1091.8	20.5
1104.8	38.7
1128.8	4.5
1164.2	1.6
1207.4	5.4
1226.7	49.7
1239.8	19.4
1244.4	12.0
1292.5	5.1
1314.3	8.1
1336.1	7.6
1351.8	3.1
1368.6	1.3
1404.5	8.6
1412.9	1.3
1421.7	24.3
1472.6	2.8
1485.7	4.3
1490.9	4.4
1496.9	3.3
1497.3	14.7
1500.9	6.7
1507.2	1.3
1519.4	6.2
3053.9	14.9
3063.2	17.4
3070.6	29.4
3080.8	24.3
3092.5	25.0
3104.9	12.5
3123.6	10.2
3124.7	16.3
3133.1	31.9
3144.2	8.0
3149.5	20.3
3172.0	8.5
3181.8	18.7
3870.4	31.6

Table 55: M06-2X/cc-pVTZ Frequencies and IR Intensities of cis-2,3- aziridine-1,2-dimethylcyclopentan-1-ol (not H-bonded)

Frequency	IR Intensity
133.3	0.6
180.0	89.5
198.2	9.7
217.7	4.0
243.0	1.3
274.1	1.0
324.1	0.6
367.0	2.1
382.7	1.7
426.6	4.9
482.2	8.1
522.7	17.5
587.4	4.1
655.5	1.7
672.1	2.3
813.0	1.9
855.1	0.6
874.1	26.0
931.0	5.5
948.6	7.3
956.7	13.6
976.8	5.8
1005.0	8.4
1018.3	34.4
1054.4	24.4
1091.9	19.1
1094.6	15.6
1128.4	32.2
1154.5	23.3
1166.5	19.3
1211.1	18.7
1241.4	25.8
1246.7	17.1
1297.2	16.4
1317.6	21.6
1339.1	1.1
1354.4	10.3
1403.8	23.0
1412.0	6.4
1419.3	4.4
1470.0	5.7
1482.4	5.7
1489.6	9.4
1492.9	7.1
1500.4	3.9
1504.1	6.7
1518.4	1.0
3054.9	13.1
3060.3	20.9
3070.9	28.3
3090.5	31.0
3115.6	19.9
3122.2	24.7
3129.6	14.2
3133.7	15.5
3146.9	15.9
3154.3	10.7
3186.3	16.6
3544.3	2.3
3873.7	29.3

Table 56: M06-2X/cc-pVTZ Frequencies and IR Intensities of cis-2,3- aziridine-1,2-dimethylcyclopentan-1-ol (H-bonded)

Frequency	IR Intensity
117.6	2.1
214.0	0.0
220.3	0.2
230.2	1.0
272.8	0.8
320.0	0.9
354.9	5.3
377.0	1.8
425.4	7.7
454.8	51.3
484.5	44.2
528.0	16.8
594.9	4.1
660.6	8.3
668.9	1.1
813.2	0.2
847.1	0.3
878.9	15.4
931.6	9.8
946.3	11.5
961.6	8.7
981.3	1.7
1008.0	11.9
1024.1	56.7
1055.0	24.8
1091.3	5.0
1111.0	9.0
1133.8	21.4
1139.1	10.8
1182.0	1.4
1204.1	18.5
1234.2	84.9
1252.6	0.5
1305.7	10.7
1338.7	4.0
1353.0	0.5
1360.2	6.4
1394.5	36.0
1415.1	9.6
1431.7	45.5
1473.6	3.9
1483.3	7.4
1488.5	8.0
1491.0	8.6
1501.7	2.1
1504.1	6.4
1519.8	1.2
3057.2	19.6
3064.4	11.6
3071.3	29.2
3097.9	17.4
3115.3	21.4
3125.9	13.3
3133.4	13.2
3139.9	17.9
3145.2	18.7
3150.5	16.6
3188.6	16.2
3540.2	2.6
3831.2	30.5

Table 57: M06-2X/cc-pVTZ Frequencies and IR Intensities of trans-2,3- aziridine-1,2-dimethylcyclopentan-1-ol

Frequency	IR Intensity
125.1	1.2
200.1	0.3
228.3	7.4
245.1	3.6
255.9	87.0
261.0	0.0
286.9	7.9
352.2	1.3
385.6	8.3
432.7	0.4
476.0	17.0
502.0	1.6
600.7	4.8
649.0	0.1
684.7	0.2
816.3	2.7
854.4	1.8
873.8	15.4
925.7	6.8
946.7	15.4
957.9	10.2
975.2	18.9
1005.1	10.5
1024.8	44.7
1049.4	36.0
1093.7	13.0
1099.4	15.1
1130.9	9.7
1145.3	6.2
1170.7	5.0
1210.3	6.9
1239.4	18.1
1247.6	72.0
1298.5	15.4
1317.4	12.0
1348.2	1.7
1359.1	2.9
1401.4	12.2
1409.7	11.8
1424.0	13.0
1472.8	2.4
1479.3	9.0
1484.8	8.7
1492.5	2.6
1495.1	5.1
1505.1	3.2
1514.2	3.7
3052.6	19.1
3056.7	25.4
3080.0	29.4
3090.1	31.9
3117.8	23.1
3121.5	14.5
3123.3	13.8
3143.8	3.6
3148.3	23.8
3153.3	6.4
3193.3	16.1
3541.9	2.4
3876.5	31.0

Table 58: M06-2X/cc-pVTZ Frequencies and IR Intensities of cis-2,3-aziridine-1-ethyl-2-methylcyclopentan-1-ol (not H-bonded)

Frequency	IR Intensity
69.1	3.0
125.2	23.4
144.9	75.2
186.8	5.1
210.8	1.2
225.5	1.8
273.2	9.3
297.7	1.2
333.5	0.6
362.9	0.8
389.1	1.9
429.1	7.8
485.5	3.6
536.1	16.4
589.2	2.7
651.1	2.2
696.0	1.7
792.5	0.6
803.1	2.8
855.6	1.5
873.7	24.4
931.9	10.6
953.7	3.6
977.8	13.1
982.5	45.4
1012.2	13.1
1026.7	1.9
1051.9	34.9
1054.4	18.8
1096.2	3.3
1099.8	11.1
1127.2	31.8
1153.0	19.4
1163.1	3.3
1194.4	9.1
1235.1	29.4
1257.8	11.5
1282.1	33.7
1305.2	0.2
1323.2	5.9
1344.0	1.0
1357.9	8.5
1373.2	8.6
1403.7	8.6
1414.3	5.3
1424.0	5.0
1472.1	4.2
1481.0	9.7
1484.4	9.2
1494.4	6.9
1500.1	2.1
1508.2	5.4
1513.8	5.4
1515.5	8.4
3057.2	9.2
3060.6	22.6
3067.1	21.4
3071.5	32.6
3102.8	23.2
3106.7	10.4
3117.2	18.8
3128.5	14.6
3133.6	25.8
3137.7	5.4
3141.5	38.7
3155.8	14.7
3187.1	17.3
3544.4	2.2
3883.7	30.5

Table 59: M06-2X/cc-pVTZ Frequencies and IR Intensities of cis-2,3-aziridine-1-ethyl-2-methylcyclopentan-1-ol (H-bonded)

Frequency	IR Intensity
77.6	0.6
117.4	1.1
185.2	0.9
215.8	0.3
222.2	0.4
243.4	1.2
290.4	2.0
326.1	1.0
355.1	1.8
385.3	3.1
429.5	6.3
468.6	36.8
490.0	55.1
540.5	19.2
596.4	3.0
655.1	8.3
694.5	1.3
795.5	0.4
804.7	0.4
847.2	1.2
877.9	13.3
933.3	15.2
953.3	6.6
983.0	57.5
987.6	6.4
1019.1	8.6
1035.2	7.1
1052.8	23.2
1053.7	24.0
1097.8	7.2
1105.3	5.7
1133.5	16.6
1139.9	10.8
1173.8	4.9
1194.6	6.7
1224.1	74.3
1251.9	2.3
1294.6	4.3
1322.4	0.9
1340.3	10.8
1344.7	1.8
1362.5	4.2
1371.0	20.4
1411.2	7.7
1415.1	11.7
1430.4	37.3
1474.0	1.9
1480.4	11.6
1486.1	9.6
1492.0	5.3
1499.2	1.5
1508.4	9.0
1512.5	5.5
1514.3	6.6
3053.1	17.5
3057.4	21.1
3070.8	30.3
3074.7	22.5
3100.5	14.7
3106.7	11.0
3118.1	22.2
3125.5	14.4
3135.8	6.0
3138.9	43.9
3153.2	5.4
3159.5	24.6
3188.5	17.0
3537.7	2.6
3832.1	34.0

Table 60: M06-2X/cc-pVTZ Frequencies and IR Intensities of trans-2,3-aziridine-1-ethyl-2-methylcyclopentan-1-ol

Frequency	IR Intensity
99.6	0.1
123.0	1.3
175.1	1.4
201.8	0.2
242.9	13.8
246.4	1.6
277.6	73.2
285.4	18.4
305.3	7.1
362.0	2.0
409.4	3.3
440.9	2.1
486.0	15.0
509.1	3.1
586.9	3.9
669.5	0.4
692.3	0.3
790.2	3.4
829.4	2.6
850.8	2.0
875.5	16.1
933.3	11.9
963.2	5.7
975.1	15.0
990.4	8.7
1004.5	80.7
1025.3	16.7
1041.3	1.8
1052.9	18.6
1096.0	2.1
1103.0	6.4
1130.2	11.9
1143.3	4.4
1173.4	2.6
1203.5	3.3
1233.4	55.3
1245.8	14.4
1284.3	38.0
1313.0	10.9
1324.2	0.6
1343.8	1.0
1360.0	2.5
1368.2	4.0
1407.1	14.7
1412.0	2.3
1431.6	10.5
1475.3	2.1
1480.8	6.7
1484.4	8.9
1489.9	1.6
1493.6	3.9
1506.2	8.0
1512.4	6.6
1514.6	4.2
3056.4	18.4
3057.1	13.6
3063.6	31.7
3079.7	32.1
3089.4	26.5
3102.3	1.3
3119.0	23.1
3121.9	10.2
3125.5	28.7
3137.7	25.8
3142.3	19.0
3152.3	6.0
3193.0	16.8
3539.9	2.5
3882.6	28.1

Table 61: M06-2X/cc-pVTZ Frequencies and IR Intensities of cis-2-ethyl-2,3-aziridine-1-methylcyclopentan-1-ol (not H-bonded)

Frequency	IR Intensity
88.1	0.2
118.0	0.3
187.9	3.6
207.8	31.0
214.6	64.0
243.1	0.9
279.9	4.0
306.5	0.8
336.7	0.6
370.1	1.5
416.7	4.3
442.0	4.6
486.2	11.8
545.1	5.4
597.2	8.9
647.1	1.8
678.9	3.4
771.2	3.2
816.7	3.7
868.4	3.6
885.0	20.8
944.2	2.9
948.7	39.0
966.8	6.8
979.4	1.9
1008.8	2.4
1020.6	2.2
1023.9	32.5
1076.3	18.6
1092.5	13.4
1109.0	11.9
1119.7	1.8
1150.9	68.1
1161.2	12.5
1211.2	17.6
1239.7	19.3
1243.9	24.5
1262.2	9.2
1312.2	8.9
1324.9	16.2
1337.0	2.0
1352.0	12.1
1372.7	2.4
1405.9	18.8
1413.0	5.5
1424.6	10.2
1466.7	4.8
1480.6	2.8
1489.7	3.0
1494.6	9.6
1498.8	6.8
1504.9	3.4
1509.7	4.9
1515.0	8.1
3053.3	13.5
3061.1	18.4
3068.9	23.7
3069.7	28.5
3089.9	29.3
3095.3	8.5
3114.5	20.7
3120.5	33.7
3132.7	26.4
3143.3	16.6
3153.3	17.5
3154.0	10.7
3188.5	16.2
3546.3	2.2
3872.5	29.2

Table 62: M06-2X/cc-pVTZ Frequencies and IR Intensities of cis-2-ethyl-2,3-aziridine-1-methylcyclopentan-1-ol (H-bonded)

Frequency	IR Intensity
98.4	0.8
108.1	0.9
173.8	0.5
211.3	0.3
242.2	1.0
276.9	0.3
301.0	2.5
338.3	0.4
364.5	2.1
410.1	3.2
440.3	2.3
467.9	24.0
498.5	83.6
547.9	5.9
603.9	6.2
652.3	8.4
676.9	4.0
775.1	3.3
817.1	1.2
863.9	2.0
890.4	11.6
940.9	21.2
948.5	17.3
968.8	11.5
983.9	2.2
1012.2	3.3
1022.2	21.2
1030.6	39.0
1073.9	20.7
1097.1	3.8
1109.6	12.1
1124.5	5.0
1141.4	14.1
1178.2	3.7
1202.1	20.4
1234.0	86.5
1249.6	0.7
1273.2	0.4
1315.0	11.5
1336.2	7.6
1350.3	1.5
1364.9	12.5
1374.3	5.0
1400.9	25.3
1415.4	6.6
1433.4	51.9
1472.3	4.6
1482.3	3.6
1487.5	5.2
1491.0	8.8
1500.1	7.0
1505.3	3.0
1511.5	3.3
1516.3	3.7
3053.6	21.1
3064.6	9.4
3069.6	25.3
3070.9	27.6
3089.7	9.7
3098.9	17.9
3115.2	23.4
3134.0	25.6
3141.6	19.2
3144.5	14.0
3147.8	24.4
3158.9	13.4
3185.7	16.2
3539.0	2.7
3826.3	31.7

Table 63: M06-2X/cc-pVTZ Frequencies and IR Intensities of trans-2-ethyl-2,3-aziridine-1-methylcyclopentan-1-ol

Frequency	IR Intensity
43.5	0.1
123.7	1.0
180.8	0.8
232.4	0.4
236.9	4.0
262.6	0.5
268.4	1.6
297.1	86.5
310.5	4.2
380.9	10.8
402.5	4.7
439.8	0.2
476.8	16.2
541.6	3.7
597.3	4.6
642.3	0.9
690.1	0.8
774.4	3.6
819.2	3.1
870.3	2.8
885.0	13.4
931.1	13.2
947.2	26.4
965.3	28.4
988.9	1.1
1004.8	5.1
1023.9	27.4
1031.8	22.7
1077.4	14.2
1096.4	22.9
1105.6	13.8
1126.3	3.1
1138.9	1.3
1169.4	5.7
1207.9	7.1
1238.7	7.8
1244.7	87.2
1266.2	1.0
1313.9	12.0
1324.3	15.2
1349.1	4.6
1357.6	2.6
1370.6	3.5
1401.4	14.9
1414.8	8.7
1430.3	12.3
1468.5	2.5
1479.6	6.5
1482.3	3.4
1493.3	3.8
1495.7	4.0
1499.3	4.9
1504.5	4.3
1516.6	4.9
3046.2	26.4
3053.2	19.1
3068.9	25.9
3078.5	28.9
3083.3	22.9
3090.1	34.1
3121.5	13.8
3122.2	17.9
3130.1	33.9
3145.8	3.8
3150.3	23.0
3179.1	6.6
3188.4	15.6
3542.3	2.5
3871.3	29.5

Table 64: M06-2X/cc-pVTZ Frequencies and IR Intensities of cis-2,3-phosphirane-1,2-dimethylcyclopentan-1-ol (not H-bonded)

Frequency	IR Intensity
119.3	0.0
208.9	2.2
213.7	1.1
236.8	7.6
260.1	80.6
276.8	1.4
308.7	0.9
324.0	6.8
354.0	0.0
383.1	0.8
429.2	3.9
481.9	10.1
560.0	1.5
596.4	0.2
607.9	0.9
652.2	1.4
715.3	1.7
765.7	1.7
837.9	4.2
894.3	8.0
921.9	9.3
945.2	8.4
957.6	1.0
978.7	0.3
989.4	7.0
1014.9	8.3
1053.2	4.3
1086.7	19.0
1105.2	25.8
1141.3	21.3
1177.2	33.3
1204.2	7.9
1236.0	53.3
1238.4	6.6
1262.6	4.2
1324.2	18.6
1330.3	16.0
1368.3	2.3
1396.7	27.2
1403.6	8.2
1412.9	4.5
1488.2	2.3
1489.8	2.8
1496.4	9.1
1499.4	12.1
1510.0	4.7
1516.3	1.7
2429.9	78.0
3053.4	15.0
3055.3	27.5
3059.3	34.6
3081.8	13.0
3115.2	19.1
3117.7	14.6
3122.8	21.6
3129.4	26.1
3143.6	11.7
3151.9	12.7
3196.7	5.4
3871.0	30.6

Table 65: M06-2X/cc-pVTZ Frequencies and IR Intensities of cis-2,3-phosphirane-1,2-dimethylcyclopentan-1-ol (H-bonded)

Frequency	IR Intensity
126.2	1.8
209.0	0.5
217.1	0.1
236.5	0.2
266.4	1.2
308.1	0.3
316.5	0.5
347.4	8.9
367.7	53.8
387.4	21.4
428.6	6.2
479.2	12.3
560.3	3.3
595.7	0.2
608.4	5.9
642.6	1.6
710.6	2.0
759.6	1.0
833.6	0.5
885.5	8.2
925.9	1.6
948.8	1.6
963.2	4.0
979.5	0.0
990.3	6.4
1016.5	44.7
1051.9	3.7
1089.3	8.5
1101.2	9.3
1142.9	29.4
1168.4	6.8
1210.6	4.5
1238.8	68.7
1245.2	16.6
1264.2	4.6
1324.8	3.0
1340.5	7.8
1379.8	45.8
1384.6	18.7
1414.3	4.1
1418.4	32.9
1482.9	2.1
1490.1	1.7
1494.6	9.1
1497.9	14.2
1509.0	6.2
1517.2	1.0
2441.7	67.1
3055.6	24.7
3062.0	20.9
3069.7	18.9
3078.3	10.5
3119.2	21.2
3119.4	12.3
3135.3	19.0
3142.9	10.2
3144.5	20.9
3157.1	13.2
3195.7	4.6
3837.3	19.6

Table 66: M06-2X/cc-pVTZ Frequencies and IR Intensities of trans-2,3-phosphirane-1,2-dimethylcyclopentan-1-ol

Frequency	IR Intensity
119.7	0.7
196.6	0.1
218.3	3.2
236.3	22.3
243.2	3.9
256.2	66.5
293.3	2.1
318.8	1.1
330.2	4.4
390.3	6.1
410.3	2.4
457.5	2.2
561.9	2.6
608.8	1.1
611.1	3.5
654.0	12.3
724.3	2.2
784.1	3.3
826.6	6.3
893.9	7.8
911.4	4.8
945.6	0.7
955.6	22.8
980.7	12.6
988.3	2.3
1019.8	6.1
1056.1	3.9
1081.5	62.9
1096.2	0.4
1140.4	2.1
1179.4	30.1
1207.1	5.2
1227.5	0.4
1238.9	15.5
1258.4	33.9
1323.6	4.7
1342.5	4.1
1367.5	7.6
1395.7	10.6
1408.9	17.7
1412.5	14.3
1484.2	1.2
1486.0	9.2
1493.4	1.6
1498.9	4.6
1499.8	6.4
1504.6	8.1
2435.5	71.9
3054.6	18.4
3062.9	26.5
3075.8	27.9
3076.6	13.1
3118.9	14.7
3123.8	23.4
3129.4	19.0
3129.5	15.8
3143.0	8.2
3148.2	14.1
3199.9	4.4
3871.0	30.1

Table 67: M06-2X/cc-pVTZ Frequencies and IR Intensities of cis-2,3-phosphirane-1-ethyl-2-methylcyclopentan-1-ol (not H-bonded)

Frequency	IR Intensity
65.8	0.1
120.6	0.0
179.0	0.6
211.2	63.0
219.7	1.0
229.2	13.8
277.5	11.0
290.7	10.4
316.1	2.4
341.7	5.7
352.0	2.7
372.7	0.5
428.3	4.7
507.3	7.4
559.9	1.4
595.1	0.6
635.3	1.5
664.2	1.4
728.0	1.8
744.5	0.8
796.7	1.7
841.4	4.4
902.8	8.1
925.6	11.1
944.2	5.4
961.1	4.9
985.5	6.9
1013.1	14.7
1020.8	2.5
1046.2	17.1
1061.6	8.2
1086.8	8.2
1109.3	15.3
1141.0	18.9
1176.5	14.7
1192.1	9.2
1231.9	44.4
1235.2	10.3
1267.2	3.7
1309.2	11.9
1316.7	21.2
1332.9	0.8
1359.9	9.3
1374.7	0.6
1399.5	20.6
1412.1	4.1
1421.7	6.4
1479.8	2.9
1496.3	9.8
1497.5	6.8
1504.3	5.8
1507.3	5.0
1510.3	3.0
1519.4	12.6
2434.1	79.0
3055.1	24.8
3058.0	18.0
3062.6	18.4
3066.4	30.9
3088.0	10.1
3112.2	11.8
3116.5	16.1
3121.0	12.2
3130.7	15.3
3136.3	29.6
3142.9	15.2
3148.0	29.6
3195.6	5.7
3878.5	29.8

Table 68: M06-2X/cc-pVTZ Frequencies and IR Intensities of cis-2,3-phosphirane-1-ethyl-2-methylcyclopentan-1-ol (H-bonded)

Frequency	IR Intensity
129.9	0.9
154.3	0.2
220.4	0.1
227.6	0.1
233.9	0.4
257.5	0.4
290.3	0.1
315.0	0.8
328.8	2.9
357.9	6.0
373.8	70.7
415.3	4.2
446.8	11.4
476.5	6.2
564.2	2.9
599.3	2.6
602.3	1.8
652.1	2.4
711.6	1.8
747.9	2.5
798.7	2.8
835.9	0.4
891.4	8.5
928.7	4.8
938.3	8.5
961.4	3.4
983.6	10.2
1009.6	7.3
1038.2	17.1
1057.3	6.2
1075.8	28.5
1088.2	10.6
1111.6	14.5
1143.7	20.0
1173.5	8.9
1196.0	23.3
1223.3	24.6
1241.6	7.2
1252.1	6.1
1308.8	3.4
1324.9	0.9
1330.2	2.4
1372.1	41.1
1388.4	12.1
1396.6	31.8
1414.3	12.2
1419.3	2.8
1485.7	0.3
1492.1	9.7
1495.2	14.4
1502.9	2.9
1507.5	15.8
1512.2	2.6
1524.4	3.5
2441.3	65.4
3056.3	22.0
3063.4	12.2
3075.4	15.4
3079.3	14.0
3082.4	32.9
3109.4	1.9
3118.4	19.2
3119.5	14.0
3129.1	23.8
3133.1	37.9
3139.9	19.1
3141.2	12.6
3192.9	4.1
3835.9	20.3

Table 69: M06-2X/cc-pVTZ Frequencies and IR Intensities of trans-2,3-phosphirane-1-ethyl-2-methylcyclopentan-1-ol

Frequency	IR Intensity
86.3	0.0
113.7	0.5
174.0	1.2
202.2	0.1
231.4	6.4
240.2	16.0
273.3	41.0
295.1	14.8
303.2	31.9
319.3	0.3
341.8	3.4
410.5	4.5
417.5	1.8
468.4	1.4
556.2	3.1
603.7	3.8
634.6	2.5
654.8	8.9
723.2	2.5
775.6	4.2
793.9	2.1
840.7	5.3
891.7	6.9
929.0	4.7
949.8	4.4
978.0	9.1
978.7	14.5
998.4	49.4
1017.4	4.8
1037.8	2.0
1060.9	2.7
1088.5	28.2
1101.7	0.3
1143.6	1.7
1181.4	23.4
1198.8	2.9
1227.9	0.6
1238.2	20.9
1252.4	24.3
1314.6	11.9
1317.7	2.0
1343.2	3.7
1350.2	6.0
1374.9	3.9
1400.6	16.6
1411.9	6.7
1420.1	5.9
1483.5	2.1
1486.0	6.6
1488.6	2.9
1499.0	5.6
1505.4	12.4
1505.9	5.2
1515.5	5.5
2434.1	73.9
3058.7	14.1
3062.9	18.7
3067.4	28.2
3074.1	8.8
3075.2	28.4
3103.8	2.0
3118.4	19.2
3127.8	44.1
3128.2	8.6
3130.5	15.0
3138.0	23.5
3142.1	9.3
3199.2	4.7
3877.0	28.0

Table 70: M06-2X/cc-pVTZ Frequencies and IR Intensities of cis-2-ethyl-2,3-phosphirane-1-methylcyclopentan-1-ol (not H-bonded)

Frequency	IR Intensity
84.7	0.1
112.2	0.0
187.4	1.1
209.1	1.2
232.4	2.0
249.1	80.9
287.2	11.5
295.2	1.0
316.9	0.6
352.8	0.9
364.2	2.7
386.5	0.3
446.1	3.3
491.6	7.9
558.6	1.7
599.7	0.4
621.1	1.0
690.8	1.3
723.1	1.7
753.3	4.5
803.9	2.7
841.6	5.3
909.8	12.6
920.6	15.5
950.6	0.5
959.2	0.7
967.1	5.0
994.9	0.9
1016.4	7.6
1023.6	1.8
1065.5	1.9
1084.4	8.1
1109.5	26.4
1141.9	24.1
1175.5	31.4
1204.2	2.3
1210.4	24.1
1235.6	40.8
1257.8	6.3
1314.0	1.4
1320.4	16.6
1329.1	20.3
1359.1	1.4
1374.2	6.5
1394.1	21.3
1407.1	11.1
1412.0	8.1
1486.4	1.1
1492.8	1.6
1497.7	5.6
1506.0	14.8
1508.9	3.5
1511.9	1.4
1514.6	9.4
2424.7	76.6
3054.3	14.7
3058.3	33.4
3069.7	22.4
3073.8	14.9
3081.6	13.4
3112.6	5.7
3114.1	20.1
3122.7	27.2
3129.1	25.6
3131.2	26.0
3153.2	12.6
3157.8	20.1
3200.1	4.8
3871.6	30.3

Table 71: M06-2X/cc-pVTZ Frequencies and IR Intensities of cis-2-ethyl-2,3-phosphirane-1-methylcyclopentan-1-ol (H-bonded)

Frequency	IR Intensity
95.6	0.6
116.6	0.8
193.1	0.2
208.7	0.6
229.4	0.2
279.2	0.4
290.9	0.4
312.8	0.1
343.7	3.8
360.2	0.9
380.2	37.6
394.5	45.5
443.5	3.7
487.8	11.6
559.5	3.2
598.2	0.7
619.5	5.1
682.8	2.0
714.1	2.2
750.0	2.4
801.8	3.3
835.7	0.8
900.3	11.6
925.4	3.8
953.5	0.0
960.3	1.0
972.5	4.1
995.4	2.5
1017.2	34.5
1024.9	11.3
1064.7	2.7
1086.7	3.9
1105.8	11.5
1146.7	20.8
1168.8	9.3
1204.5	1.2
1228.0	34.4
1240.1	44.1
1248.5	14.8
1316.1	3.8
1325.1	12.7
1342.4	2.1
1361.5	2.8
1380.9	13.3
1384.6	48.2
1411.4	8.3
1419.1	34.6
1484.3	2.6
1489.8	4.5
1495.5	5.7
1504.9	14.5
1507.9	4.4
1510.4	0.8
1516.4	4.5
2443.7	65.5
3061.3	23.3
3068.9	10.2
3071.1	21.6
3072.2	20.2
3079.6	9.8
3110.3	5.9
3119.1	22.5
3131.8	23.7
3134.6	19.9
3145.3	24.4
3155.2	14.9
3161.0	14.1
3198.0	4.1
3835.8	20.1

Table 72: M06-2X/cc-pVTZ Frequencies and IR Intensities of trans-2-ethyl-2,3-phosphirane-1-methylcyclopentan-1-ol

Frequency	IR Intensity
78.1	0.2
109.4	0.5
204.2	1.7
214.6	0.3
221.3	1.2
241.6	22.0
256.4	66.5
282.9	1.2
294.0	3.7
314.7	0.6
363.8	5.1
401.0	3.7
416.0	3.5
475.2	3.9
558.7	2.6
610.1	3.5
614.2	0.8
686.3	6.7
719.5	4.9
757.1	2.4
818.4	16.2
828.8	1.7
905.8	6.7
914.4	13.3
951.0	2.7
964.3	0.6
965.5	18.9
1001.0	7.3
1012.3	4.5
1034.9	2.6
1064.2	1.1
1091.6	25.1
1101.0	25.2
1139.7	3.2
1176.2	23.6
1206.0	4.0
1207.3	5.7
1233.6	11.0
1254.7	35.5
1318.0	10.3
1319.9	6.2
1341.1	2.9
1360.1	1.0
1371.1	6.4
1391.8	8.7
1409.4	1.1
1413.2	35.4
1482.6	3.1
1485.4	4.0
1494.4	2.1
1497.3	4.8
1498.1	5.0
1502.0	8.7
1514.5	3.8
2436.9	69.3
3053.9	17.9
3068.7	30.6
3072.2	29.0
3073.1	16.1
3076.4	15.0
3107.4	11.6
3118.8	15.4
3123.0	21.4
3128.3	27.2
3128.6	26.9
3149.8	13.5
3173.1	8.0
3205.1	3.9
3867.6	28.5

Table 73: M06-2X/cc-pVTZ Frequencies and IR Intensities of cis-2,3-thiirane-1,2-dimethylcyclopentan-1-ol (not H-bonded)

Frequency	IR Intensity
123.1	0.2
210.2	33.6
216.2	11.1
223.4	43.9
242.8	9.6
277.1	0.8
328.6	2.1
332.7	0.9
357.9	0.4
383.4	1.0
434.8	6.4
497.8	11.8
573.5	3.2
619.7	1.0
629.6	2.4
679.1	14.4
774.1	0.8
836.3	6.2
914.7	2.7
943.1	4.7
950.0	2.2
985.3	0.9
990.7	8.0
1017.8	7.2
1059.5	1.8
1079.6	18.9
1102.8	34.9
1144.4	17.9
1188.8	37.1
1207.4	9.2
1238.6	2.3
1240.9	58.9
1268.9	2.0
1327.6	4.2
1333.6	23.0
1379.4	0.7
1399.1	32.6
1405.9	1.5
1415.3	6.5
1483.7	2.0
1489.3	12.2
1491.5	5.6
1498.6	11.0
1502.9	2.0
1514.4	1.1
3055.9	10.1
3061.3	25.3
3063.4	23.5
3094.7	22.6
3122.0	20.5
3124.7	17.7
3130.0	11.5
3151.0	9.6
3153.4	11.6
3156.9	8.5
3188.6	7.6
3874.2	34.3

Table 74: M06-2X/cc-pVTZ Frequencies and IR Intensities of cis-2,3-thiirane-1,2-dimethylcyclopentan-1-ol (H-bonded)

Frequency	IR Intensity
122.6	2.6
217.3	0.7
228.2	0.2
233.2	0.2
272.7	0.8
323.8	0.7
326.5	0.6
352.5	1.2
378.9	3.0
415.1	64.3
436.6	28.8
502.1	19.2
572.7	6.7
613.0	0.4
630.4	5.4
673.9	11.0
767.5	1.0
832.6	3.1
914.4	0.2
946.6	3.1
959.1	1.4
986.6	8.5
992.4	3.8
1020.8	32.7
1058.6	9.4
1082.3	13.6
1103.8	6.6
1147.2	29.7
1169.7	8.4
1218.5	6.9
1243.2	38.0
1253.2	41.0
1273.4	1.1
1326.5	1.3
1346.6	7.9
1381.8	56.2
1398.9	15.3
1416.9	8.7
1421.5	20.6
1482.4	4.3
1485.3	3.6
1491.0	13.0
1496.9	8.6
1501.6	4.7
1515.9	0.4
3062.3	20.5
3063.2	12.5
3068.5	17.4
3101.8	11.9
3125.7	16.8
3130.0	9.3
3143.7	13.2
3147.2	15.4
3155.4	11.1
3158.0	11.1
3188.9	6.0
3830.5	23.5

Table 75: M06-2X/cc-pVTZ Frequencies and IR Intensities of trans-2,3-thiirane-1,2-dimethylcyclopentan-1-ol

Frequency	IR Intensity
118.8	1.0
206.9	0.5
222.9	1.9
238.5	10.2
250.4	5.5
260.8	79.9
299.6	4.5
330.3	1.5
340.8	2.6
392.1	4.7
416.0	2.5
468.6	2.3
581.7	8.5
620.4	0.8
632.0	4.0
679.9	27.4
788.7	7.0
832.3	1.1
907.7	4.6
930.9	6.0
955.9	21.0
982.5	5.5
995.8	0.3
1018.7	6.5
1061.6	10.5
1078.9	52.3
1096.9	9.1
1143.4	1.3
1189.8	34.7
1209.7	9.5
1231.9	2.0
1237.9	12.9
1264.8	22.3
1328.2	4.5
1346.1	6.3
1379.5	10.4
1401.6	8.1
1410.2	26.8
1415.4	3.2
1476.7	4.1
1486.3	8.3
1491.0	4.2
1494.6	5.8
1500.1	6.9
1503.0	3.0
3055.7	17.0
3070.5	18.2
3075.3	20.6
3094.2	23.7
3124.8	15.0
3130.2	13.6
3139.3	10.6
3146.5	6.2
3151.0	16.7
3157.3	6.9
3194.7	6.1
3872.8	34.3

Table 76: M06-2X/cc-pVTZ Frequencies and IR Intensities of cis-2,3-thiirane-1-ethyl-2-methylcyclopentan-1-ol ((not H-bonded)

Frequency	IR Intensity
72.8	0.3
123.6	0.7
180.3	4.2
194.4	79.0
223.1	1.3
229.1	2.9
281.5	19.3
287.4	1.2
329.2	1.7
350.2	1.5
352.5	1.5
369.8	0.7
430.3	7.7
520.8	7.2
571.7	2.9
623.7	3.3
651.8	1.3
680.8	16.5
752.9	1.4
798.0	2.1
837.4	5.7
914.7	2.9
941.2	4.7
961.2	6.3
991.1	8.7
1015.8	9.8
1021.5	5.9
1043.1	16.0
1069.7	8.3
1079.0	10.7
1107.6	16.8
1143.7	16.0
1184.3	13.4
1198.9	17.8
1232.7	20.9
1234.9	36.6
1272.4	1.7
1312.0	3.8
1326.1	17.8
1331.5	0.3
1364.0	7.5
1384.2	0.3
1400.9	21.1
1412.9	2.2
1423.3	9.8
1478.8	2.9
1486.0	11.1
1497.2	8.7
1500.1	3.8
1502.2	4.8
1507.3	4.6
1517.3	9.5
3057.2	6.2
3061.0	25.4
3063.8	21.2
3067.9	24.7
3107.9	17.0
3115.0	6.9
3125.3	13.3
3131.6	21.2
3133.9	7.6
3143.3	30.2
3155.1	7.5
3157.2	16.8
3189.0	7.9
3881.8	33.7

Table 77: M06-2X/cc-pVTZ Frequencies and IR Intensities of cis-2,3-thiirane-1-ethyl-2-methylcyclopentan-1-ol ((H-bonded)

Frequency	IR Intensity
130.9	1.7
158.0	0.3
213.2	0.3
235.4	0.2
241.9	1.1
259.8	0.2
281.4	0.1
324.1	1.5
342.8	0.5
357.9	0.4
415.4	8.1
419.1	67.0
457.8	24.6
496.7	13.0
576.8	8.2
614.2	0.4
636.1	3.0
675.3	13.8
757.8	0.4
798.1	3.9
836.7	2.2
915.1	0.8
935.3	17.0
962.9	5.4
988.7	1.7
1008.7	8.3
1037.1	9.7
1064.3	9.5
1074.2	32.9
1085.0	9.0
1114.3	12.5
1149.1	16.9
1173.7	10.9
1202.4	30.4
1230.2	18.6
1242.6	5.8
1258.9	6.4
1313.3	2.4
1327.3	0.3
1333.7	1.2
1374.1	44.0
1397.8	36.2
1401.5	11.0
1417.9	4.0
1420.7	5.5
1483.0	1.1
1487.8	14.3
1494.2	9.5
1499.9	4.8
1506.2	9.5
1508.2	5.2
1521.6	4.1
3062.4	6.0
3064.8	19.1
3076.8	15.4
3080.1	29.3
3098.5	14.4
3110.1	1.8
3125.1	14.2
3128.8	25.8
3134.0	16.4
3140.5	31.7
3144.0	11.8
3153.8	6.9
3188.9	5.7
3827.5	24.1

Table 78: M06-2X/cc-pVTZ Frequencies and IR Intensities of trans-2,3-thiirane-1-ethyl-2-methylcyclopentan-1-ol

Frequency	IR Intensity
90.8	0.1
114.5	0.9
174.2	1.1
211.9	0.2
236.2	3.3
244.4	18.3
279.7	51.7
297.3	14.7
303.1	23.6
330.8	0.7
348.9	2.3
415.8	4.8
423.1	1.2
485.5	1.9
575.4	6.7
620.6	4.1
654.1	2.6
679.1	25.2
779.5	8.7
797.7	1.0
844.0	0.8
914.5	2.5
952.0	15.5
974.9	4.8
984.6	20.4
1002.5	30.9
1019.0	6.9
1036.3	2.2
1065.8	6.3
1082.5	25.6
1104.0	2.1
1147.1	1.2
1190.4	28.6
1202.9	7.8
1229.0	0.8
1237.1	19.0
1258.9	15.9
1317.6	5.7
1324.0	7.4
1344.4	3.8
1354.1	6.3
1388.4	9.1
1402.7	15.0
1414.4	5.1
1424.1	3.5
1478.0	3.7
1484.4	6.3
1488.5	3.2
1492.1	6.1
1503.1	7.4
1505.8	8.2
1515.2	6.1
3058.6	14.5
3067.7	11.5
3071.1	25.3
3075.0	21.5
3092.3	20.3
3106.0	1.2
3128.6	28.7
3129.4	11.5
3139.0	15.0
3140.4	18.7
3143.3	14.6
3156.6	6.3
3196.0	6.3
3878.2	31.3

Table 79: M06-2X/cc-pVTZ Frequencies and IR Intensities of cis-2-ethyl-2,3-thiirane-1-methylcyclopentan-1-ol (not H-bonded)

Frequency	IR Intensity
86.7	0.3
112.0	0.2
187.3	1.8
210.0	2.6
227.1	76.8
235.0	12.1
281.1	4.9
295.1	0.8
335.4	1.4
356.8	1.1
369.2	1.5
387.8	1.0
445.7	5.0
505.7	11.2
568.4	2.6
625.0	3.8
636.1	1.3
715.1	14.1
755.7	1.7
809.6	4.0
840.4	10.8
913.2	7.5
945.4	2.6
951.1	0.9
968.5	5.7
997.0	4.9
1017.1	2.5
1030.7	4.5
1067.9	1.6
1084.9	9.7
1110.2	32.2
1145.4	18.7
1186.2	37.6
1207.4	7.8
1212.7	7.7
1238.7	48.5
1263.9	3.1
1314.0	4.8
1324.8	3.1
1332.7	23.5
1362.3	2.2
1384.4	2.2
1398.8	23.3
1406.5	5.6
1413.4	9.2
1482.9	3.1
1489.0	1.3
1495.9	8.9
1499.1	9.4
1505.9	4.0
1511.1	4.2
1515.3	9.0
3055.7	11.6
3058.1	26.7
3071.5	19.2
3076.9	13.4
3094.5	23.6
3122.1	19.0
3124.2	11.4
3124.8	18.2
3134.5	21.5
3151.6	10.5
3153.5	12.3
3160.0	18.4
3192.5	6.9
3874.3	34.2

Table 80: M06-2X/cc-pVTZ Frequencies and IR Intensities of cis-2-ethyl-2,3-thiirane-1-methylcyclopentan-1-ol (H-bonded)

Frequency	IR Intensity
90.7	0.6
110.1	1.4
181.5	0.6
207.1	0.2
234.9	0.8
274.7	0.1
291.1	0.4
330.3	0.2
350.9	2.4
368.6	0.6
384.1	3.6
425.6	61.0
447.4	31.2
506.8	19.0
569.1	5.8
617.4	3.0
639.1	2.2
707.8	13.0
756.3	0.9
808.9	3.2
836.2	7.0
916.5	2.2
946.1	4.1
957.7	1.1
973.2	4.1
997.6	11.1
1022.2	16.3
1031.9	17.0
1069.0	7.9
1086.8	7.8
1108.1	8.8
1151.5	19.6
1170.3	10.5
1211.4	2.6
1233.7	23.5
1248.6	54.6
1253.5	5.2
1323.4	7.1
1323.7	5.8
1349.2	2.8
1364.0	1.7
1385.0	56.1
1400.0	6.0
1413.2	8.4
1421.1	29.0
1482.7	3.4
1485.7	6.1
1493.3	4.3
1499.5	12.4
1505.3	4.7
1511.2	3.6
1516.1	4.0
3059.1	20.3
3069.0	6.7
3071.6	18.9
3075.7	17.6
3102.8	12.6
3121.8	2.9
3126.7	18.5
3135.0	21.6
3145.4	15.3
3148.0	16.4
3154.3	17.6
3164.7	13.7
3193.1	5.5
3828.4	23.8

Table 81: M06-2X/cc-pVTZ Frequencies and IR Intensities of trans-2-ethyl-2,3-thiirane-1-methylcyclopentan-1-ol

Frequency	IR Intensity
61.8	0.1
112.0	0.8
191.9	0.7
215.7	0.3
228.6	2.5
249.0	2.7
269.5	64.1
274.5	24.4
299.8	4.2
331.4	1.5
371.2	4.0
407.5	6.1
415.3	1.7
483.0	4.5
576.3	7.3
622.9	2.5
635.5	1.6
712.4	23.3
764.3	2.6
816.3	14.1
841.1	5.2
903.7	9.9
934.7	3.4
962.8	16.1
970.2	4.9
997.7	9.9
1014.2	1.8
1040.2	3.9
1067.2	0.3
1096.4	13.3
1099.6	40.3
1143.9	1.8
1186.4	30.9
1206.0	4.3
1214.6	2.1
1234.1	12.8
1259.7	21.7
1316.2	11.6
1326.4	4.8
1344.9	4.0
1361.9	1.0
1387.6	5.0
1398.9	2.1
1409.5	2.1
1412.6	36.0
1476.5	3.1
1484.8	5.1
1490.8	5.4
1495.5	3.4
1499.4	2.1
1501.5	8.9
1516.4	5.1
3055.4	16.6
3069.3	24.4
3071.3	20.8
3074.3	19.3
3094.0	23.2
3116.9	9.7
3124.5	11.9
3129.1	19.3
3130.4	27.8
3146.2	9.0
3153.9	14.3
3177.5	7.0
3197.2	5.4
3869.3	33.3

Table 82: M06-2X optimized geometry of cis-2,3-epoxy-1,2-dimethylcyclopentan-1-ol (not H-bonded) (in Å) with the aTZ basis set

Atom	x	y	z
C	−0.255493	0.784830	−0.022740
C	−1.554546	0.188680	−0.347625
C	−1.430016	−1.314834	−0.423343
C	−0.120823	−1.588670	0.333592
C	0.758241	−0.345139	0.097911
C	1.566518	−0.468303	−1.188670
H	2.240613	−1.324255	−1.121017
H	0.912941	−0.621023	−2.049095
H	2.164080	0.426653	−1.354247
O	1.614951	−0.055560	1.190538
H	2.254187	−0.766851	1.283788
H	−0.317766	−1.658299	1.400779
H	0.373347	−2.503721	0.007701
H	−2.284246	−1.825873	0.019666
H	−1.362849	−1.619889	−1.469479
O	−1.284805	0.689921	0.956300
H	−2.312540	0.726117	−0.904406
C	0.184969	2.169874	−0.380957
H	0.626311	2.199598	−1.376412
H	−0.670008	2.842405	−0.356307
H	0.927404	2.517708	0.337614

Table 83: M06-2X optimized geometry of cis-2,3-epoxy-1,2-dimethylcyclopentan-1-ol (H-bonded) (in Å) with the aTZ basis set

Atom	x	y	z
C	−0.018512	−0.004782	0.019634
C	1.527000	−0.031630	0.023641
C	1.982660	1.434289	0.012010
C	0.854397	2.140833	−0.699030
C	−0.352579	1.316229	−0.667411
C	−1.759093	1.823338	−0.716416
H	−2.417694	1.057311	−1.127601
H	−2.115196	2.081632	0.280146
H	−1.810103	2.707766	−1.348235
O	0.412243	1.452660	−1.869412
H	0.811641	3.221212	−0.762621
H	2.068607	1.837132	1.023115
H	2.942682	1.572738	−0.484315
H	1.913677	−0.592724	0.872138
H	1.863813	−0.527941	−0.884775
O	−0.575786	−1.101447	−0.684308
H	−0.261801	−1.054317	−1.593566
C	−0.593546	−0.053831	1.422227
H	−0.302927	−0.988134	1.900248
H	−0.217954	0.778929	2.018825
H	−1.681048	−0.006217	1.392921

Table 84: M06-2X optimized geometry of trans-2,3-epoxy-1,2-dimethylcyclopentan-1-ol (in Å) with the aTZ basis set

Atom	x	y	z
C	0.726363	−0.410665	0.005332
C	−0.226451	−1.588396	0.276265
C	−1.571997	−1.183458	−0.345589
C	−1.565459	0.318867	−0.218861
C	−0.198135	0.800920	−0.018248
O	−1.122699	0.766564	1.063869
H	−2.327766	0.933381	−0.681078
H	−1.616498	−1.446942	−1.402938
H	−2.421082	−1.643657	0.158516
H	0.176260	−2.510163	−0.143221
H	−0.336807	−1.724767	1.350146
C	0.324877	2.142733	−0.420160
H	−0.492683	2.858486	−0.480742
H	1.042718	2.505511	0.317063
H	0.822795	2.076562	−1.385462
C	1.843321	−0.293307	1.025070
H	2.415978	−1.222205	1.073949
H	2.521261	0.515660	0.753916
H	1.429459	−0.095624	2.013674
O	1.249021	−0.480778	−1.321148
H	1.868244	−1.214063	−1.367232

Table 85: M06-2X optimized geometry of cis-2,3-epoxy-1-ethyl-2-methylcyclopentan-1-ol (not H-bonded) (in Å) with the aTZ basis set

Atom	x	y	z
C	−0.920003	0.588809	−0.105052
C	−1.713443	−0.548525	−0.579605
C	−0.908341	−1.820775	−0.466967
C	0.195511	−1.439751	0.531935
C	0.449859	0.065441	0.312889
C	1.466934	0.330035	−0.804094
C	2.888663	−0.103876	−0.467425
H	2.957990	−1.171669	−0.261224
H	3.559648	0.111783	−1.297538
H	3.268139	0.438465	0.399289
H	1.125543	−0.173476	−1.712837
H	1.469665	1.400619	−1.012445
O	0.830464	0.742225	1.500375
H	1.591323	0.299548	1.884376
H	−0.169639	−1.567724	1.548443
H	1.095383	−2.039691	0.410150
H	−1.507459	−2.663932	−0.124268
H	−0.496604	−2.077877	−1.444859
O	−1.967528	0.040856	0.689637
H	−2.503446	−0.436268	−1.312230
C	−1.104142	2.013829	−0.527731
H	−0.574357	2.227873	−1.454697
H	−2.163646	2.211043	−0.677699
H	−0.728984	2.680934	0.248807

Table 86: M06-2X optimized geometry of cis-2,3-epoxy-1-ethyl-2-methylcyclopentan-1-ol (H-bonded) (in Å) with the aTZ basis set

Atom	x	y	z
C	0.425930	0.099496	0.664434
C	−0.652805	0.604099	−0.289708
C	−1.372871	−0.541382	−0.844691
C	−0.758014	−1.830641	−0.361927
C	0.080680	−1.399546	0.852546
H	0.979063	−2.001872	0.980157
H	−0.507985	−1.496996	1.762632
H	−0.138755	−2.245736	−1.158211
H	−1.512836	−2.573476	−0.104717
O	−1.942434	0.232085	0.210905
H	−1.903213	−0.485165	−1.787710
C	−0.554299	1.961222	−0.910969
H	−1.469700	2.187000	−1.454237
H	−0.409315	2.714575	−0.135572
H	0.287987	2.015054	−1.600490
O	0.367331	0.815278	1.887980
H	−0.538284	0.759625	2.211775
C	1.829721	0.330861	0.121493
C	2.117593	−0.381171	−1.194635
H	1.398023	−0.107083	−1.968211
H	3.110651	−0.123897	−1.559258
H	2.082547	−1.464510	−1.077348
H	1.975055	1.407154	0.012979
H	2.529728	−0.000923	0.890293

Table 87: M06-2X optimized geometry of trans-2,3-epoxy-1-ethyl-2-methylcyclopentan-1-ol (in Å) with the aTZ basis set

Atom	x	y	z
C	−0.467271	−0.009888	0.212898
C	0.887398	0.636627	−0.054880
C	1.903153	−0.416460	−0.033628
C	1.261073	−1.748027	0.258162
C	−0.218895	−1.502530	−0.075612
H	−0.891145	−2.130237	0.508838
H	−0.402922	−1.702754	−1.129855
H	1.395961	−1.979203	1.315445
H	1.691280	−2.559864	−0.327333
O	1.461502	0.155584	−1.266165
H	2.935120	−0.212415	0.223130
C	1.117377	2.076531	0.277155
H	2.166482	2.328569	0.134582
H	0.520876	2.716332	−0.374668
H	0.833484	2.271978	1.309389
C	−1.583017	0.601249	−0.625246
C	−2.928144	−0.094378	−0.449805
H	−2.873915	−1.144144	−0.739000
H	−3.275562	−0.044508	0.584259
H	−3.693140	0.379542	−1.062751
H	−1.674480	1.654590	−0.352446
H	−1.274462	0.559559	−1.671959
O	−0.687571	0.203240	1.606953
H	−1.504788	−0.233887	1.859301

Table 88: M06-2X optimized geometry of cis-2-ethyl-2,3- epoxy-1-methylcyclopentan-1-ol (not H-bonded) (in Å) with the aTZ basis set

Atom	x	y	z
C	−0.398510	−0.289825	0.375019
C	0.078513	−1.563641	−0.174546
C	1.415778	−1.369109	−0.849866
C	1.915260	−0.048195	−0.244164
C	0.646854	0.773380	0.060196
C	0.222785	1.611278	−1.139727
H	1.002740	2.339483	−1.371099
H	0.070303	0.988449	−2.022532
H	−0.697884	2.152350	−0.924556
O	0.784629	1.600768	1.204489
H	1.436804	2.282027	1.020500
H	2.419210	−0.241281	0.699848
H	2.598078	0.487993	−0.902957
H	2.099083	−2.197851	−0.666753
H	1.267766	−1.288831	−1.928600
O	0.132658	−1.306787	1.222071
H	−0.599118	−2.353391	−0.475507
C	−1.843300	0.083049	0.592495
C	−2.710748	−0.009327	−0.661081
H	−2.717950	−1.026955	−1.051724
H	−3.739775	0.269351	−0.436867
H	−2.353442	0.646924	−1.453135
H	−2.234666	−0.586918	1.357520
H	−1.878477	1.091676	1.009336

Table 89: M06-2X optimized geometry of cis-2-ethyl-2,3- epoxy-1-methylcyclopentan-1-ol (H-bonded) (in Å) with the aTZ basis set

Atom	x	y	z
C	−1.846771	−0.013084	0.594264
C	−0.391258	−0.310166	0.351055
C	0.135393	−1.570901	−0.171976
C	1.429847	−1.325501	−0.908181
C	1.895318	0.028790	−0.355578
C	0.615970	0.798198	0.050099
C	0.137015	1.723543	−1.051327
H	−0.788374	2.220329	−0.762262
H	0.894052	2.486144	−1.229200
H	−0.029076	1.169320	−1.976064
O	0.817829	1.612895	1.192674
H	1.064212	1.033859	1.921965
H	2.499031	−0.126124	0.536648
H	2.492388	0.596828	−1.066378
H	2.160043	−2.118337	−0.748713
H	1.221192	−1.273773	−1.978630
O	0.218267	−1.274156	1.220882
H	−0.509223	−2.404992	−0.421767
H	−2.211780	−0.753793	1.305915
H	−1.924704	0.961669	1.081390
C	−2.705631	−0.050779	−0.668019
H	−2.383399	0.685474	−1.402034
H	−2.656292	−1.033407	−1.137674
H	−3.748232	0.153267	−0.427644

Table 90: M06-2X optimized geometry of trans-2-ethyl-2,3- epoxy-1-methylcyclopentan-1-ol (in Å) with the aTZ basis set

Atom	x	y	z
C	0.559364	0.805161	−0.011993
C	1.886259	0.085727	−0.321334
C	1.498462	−1.317803	−0.810662
C	0.212278	−1.588159	−0.071840
C	−0.382192	−0.329158	0.382047
C	−1.857233	−0.113777	0.587548
H	−2.010168	0.804892	1.158805
C	−2.666547	−0.067448	−0.706132
H	−2.522529	−0.984108	−1.279064
H	−3.729170	0.027366	−0.485146
H	−2.362841	0.768484	−1.329995
H	−2.205682	−0.934789	1.215591
O	0.269169	−1.205024	1.301802
H	−0.391042	−2.463636	−0.279181
H	1.296889	−1.324632	−1.882567
H	2.267734	−2.060367	−0.601594
H	2.460601	0.648637	−1.056960
H	2.479384	0.012924	0.587833
C	0.697579	1.864147	1.066216
H	0.991944	1.403589	2.008770
H	1.462782	2.592308	0.787515
H	−0.244890	2.392789	1.205458
O	0.000044	1.361642	−1.201231
H	0.550053	2.099490	−1.478365

Table 91: M06-2X optimized geometry of cis-2,3- aziridine-1,2-dimethylcyclopentan-1-ol (not H-bonded) (in Å) with the aTZ basis set

Atom	x	y	z
C	0.972450	0.008630	0.224321
C	−0.329106	0.609555	−0.291182
C	−1.106261	−0.478317	−0.921030
C	−0.344928	−1.780816	−0.804913
C	0.658116	−1.496903	0.324716
C	−0.363318	2.042186	−0.735785
N	−1.493204	0.043295	0.396142
C	2.108684	0.268478	−0.759283
O	1.273542	0.598460	1.479792
H	3.012436	−0.243408	−0.423375
H	1.858753	−0.105516	−1.753595
H	2.324391	1.333544	−0.827163
H	2.079281	0.203798	1.823584
H	0.188396	−1.672875	1.289254
H	1.559526	−2.105867	0.253439
H	−0.994449	−2.625844	−0.577639
H	0.166291	−1.991706	−1.746133
H	−1.750487	−0.284296	−1.768474
H	0.327682	2.223522	−1.558310
H	−1.364250	2.310254	−1.075626
H	−0.091059	2.698732	0.091700
H	−2.274401	0.681869	0.299125

Table 92: M06-2X optimized geometry of cis-2,3- aziridine-1,2-dimethylcyclopentan-1-ol (H-bonded) (in Å) with the aTZ basis set

Atom	x	y	z
C	0.300664	−0.755435	−0.053001
C	−0.788877	0.302634	0.119994
C	0.000260	1.628316	0.235479
C	1.319667	1.413597	−0.519019
C	1.563102	−0.071722	−0.382757
C	−0.060563	−2.141894	−0.494328
C	−1.745618	0.316398	−1.056702
N	1.323999	−0.565515	0.985575
O	−1.568838	0.042166	1.274198
H	−0.790116	−2.578731	0.189704
H	−0.492882	−2.138409	−1.494398
H	0.823343	−2.780470	−0.512606
H	2.324021	−0.564134	−0.973884
H	1.216459	1.661360	−1.577201
H	2.133751	2.013171	−0.112270
H	−0.576578	2.469718	−0.144336
H	0.205249	1.811247	1.288336
H	−0.952548	−0.041409	2.010710
H	−2.473211	1.115600	−0.923220
H	−1.202464	0.486854	−1.987696
H	−2.283007	−0.628100	−1.127910
H	1.826616	−1.435187	1.123291

Table 93: M06-2X optimized geometry of trans-2,3- aziridine-1,2-dimethylcyclopentan-1-ol (in Å) with the aTZ basis set

Atom	x	y	z
C	0.300664	−0.755435	−0.053001
C	−0.788877	0.302634	0.119994
C	0.000260	1.628316	0.235479
C	1.319667	1.413597	−0.519019
C	1.563102	−0.071722	−0.382757
C	−0.060563	−2.141894	−0.494328
C	−1.745618	0.316398	−1.056702
N	1.323999	−0.565515	0.985575
O	−1.568838	0.042166	1.274198
H	−0.790116	−2.578731	0.189704
H	−0.492882	−2.138409	−1.494398
H	0.823343	−2.780470	−0.512606
H	2.324021	−0.564134	−0.973884
H	1.216459	1.661360	−1.577201
H	2.133751	2.013171	−0.112270
H	−0.576578	2.469718	−0.144336
H	0.205249	1.811247	1.288336
H	−0.952548	−0.041409	2.010710
H	−2.473211	1.115600	−0.923220
H	−1.202464	0.486854	−1.987696
H	−2.283007	−0.628100	−1.127910
H	1.826616	−1.435187	1.123291

Table 94: M06-2X optimized geometry of cis-2,3- aziridine-1-ethyl-2-methylcyclopentan-1-ol (not H-bonded) (in Å) with the aTZ basis set

Atom	x	y	z
C	−0.958535	0.570540	−0.083280
C	−1.782226	−0.570033	−0.551000
C	−0.962012	−1.839976	−0.498849
C	0.190220	−1.498013	0.455663
C	0.411305	0.026108	0.318771
C	1.455039	0.357987	−0.753059
C	2.878815	−0.057087	−0.398383
H	2.963180	−1.123684	−0.190518
H	3.556103	0.168516	−1.220675
H	3.245992	0.490969	0.470569
H	1.146455	−0.119056	−1.687570
H	1.439150	1.434593	−0.923081
O	0.760393	0.630154	1.559615
H	1.606972	0.281820	1.850996
H	−0.079836	−1.717176	1.488406
H	1.089816	−2.070021	0.236984
H	−1.537710	−2.703898	−0.164201
H	−0.586146	−2.066108	−1.498494
H	−2.535535	−0.449975	−1.318816
C	−1.083275	1.975734	−0.600593
H	−0.603411	2.096643	−1.571451
H	−2.137766	2.223673	−0.700779
H	−0.630118	2.677398	0.101434
N	−2.080519	0.088962	0.714796
H	−1.771060	−0.451086	1.514906

Table 95: M06-2X optimized geometry of cis-2,3- aziridine-1-ethyl-2-methylcyclopentan-1-ol (H-bonded) (in Å) with the aTZ basis set

Atom	x	y	z
N	−1.980638	0.237658	0.215452
C	−0.660194	0.607651	−0.319759
C	0.413858	0.111857	0.649192
C	0.069044	−1.386288	0.850951
C	−0.750118	−1.834808	−0.370286
C	−1.378700	−0.555460	−0.869702
O	0.345305	0.836811	1.866179
C	1.825234	0.331295	0.119761
C	2.131674	−0.408177	−1.176885
C	−0.495521	1.924543	−1.017202
H	0.966482	−1.985291	1.001326
H	−0.535366	−1.469990	1.751640
H	−0.111913	−2.247583	−1.152629
H	−1.495105	−2.587461	−0.112395
H	−1.841704	−0.512678	−1.847044
H	−1.373251	2.148887	−1.624484
H	−0.366581	2.723829	−0.285294
H	0.375475	1.923254	−1.672221
H	−0.572749	0.793569	2.157988
H	1.414061	−0.162967	−1.961744
H	3.124767	−0.148614	−1.540358
H	2.107366	−1.488460	−1.033024
H	1.976329	1.404825	−0.006956
H	2.514236	0.012991	0.904105
H	−2.682989	0.860027	−0.168576

Table 96: M06-2X optimized geometry of trans-2,3- aziridine-1-ethyl-2-methylcyclopentan-1-ol (in Å) with the aTZ basis set

Atom	x	y	z
C	−0.444768	−0.014081	0.174599
C	0.908805	0.633835	−0.105878
C	1.937321	−0.430257	−0.045300
C	1.273636	−1.750203	0.277537
C	−0.206084	−1.517622	−0.065015
H	−0.875718	−2.119149	0.548792
H	−0.418598	−1.776822	−1.103644
H	1.391859	−1.953967	1.342034
H	1.702043	−2.588214	−0.273553
H	2.941249	−0.232528	0.306067
C	1.130949	2.058614	0.309566
H	2.168547	2.331448	0.126886
H	0.500660	2.732988	−0.272621
H	0.900370	2.191610	1.364535
C	−1.569754	0.575484	−0.670795
C	−2.918791	−0.102446	−0.458524
H	−2.875682	−1.164072	−0.703943
H	−3.259006	−0.006454	0.574399
H	−3.684433	0.351789	−1.085433
H	−1.648558	1.637812	−0.433033
H	−1.282835	0.503115	−1.722883
O	−0.675959	0.223935	1.563410
H	−1.499367	−0.201559	1.815717
N	1.592152	0.197751	−1.319940
H	1.020443	−0.411712	−1.894061

Table 97: M06-2X optimized geometry of cis-2,3- aziridine-1-ethyl-2-methylcyclopentan-1-ol (not H-bonded) (in Å) with the aTZ basis set

Atom	x	y	z
C	−0.391981	−0.324865	0.423473
C	0.081197	−1.620633	−0.124612
C	1.382888	−1.411789	−0.869418
C	1.908666	−0.077489	−0.323570
C	0.655727	0.732083	0.079528
C	0.202892	1.632492	−1.061446
H	0.981508	2.366714	−1.280345
H	0.024633	1.052240	−1.967754
H	−0.709000	2.165676	−0.796956
O	0.874862	1.510596	1.251658
H	1.456587	2.244544	1.036268
H	2.523768	−0.231467	0.562497
H	2.519550	0.460246	−1.048067
H	2.089383	−2.230054	−0.724931
H	1.176778	−1.342753	−1.939214
H	−0.609010	−2.383532	−0.460630
C	−1.845437	0.067128	0.567380
C	−2.660394	−0.013725	−0.721512
H	−2.633204	−1.024493	−1.129585
H	−3.703123	0.239743	−0.532023
H	−2.290120	0.663013	−1.489704
H	−2.281619	−0.600043	1.309639
H	−1.893782	1.074553	0.988237
N	0.081969	−1.382296	1.312456
H	1.008962	−1.186325	1.673073

Table 98: M06-2X optimized geometry of cis-2,3- aziridine-1-ethyl-2-methylcyclopentan-1-ol (H-bonded) (in Å) with the aTZ basis set

Atom	x	y	z
N	0.249763	−1.323545	1.234733
C	−0.406334	−0.337407	0.357716
C	0.611646	0.761584	0.039410
C	1.863081	−0.019219	−0.431378
C	1.357489	−1.356670	−0.989271
C	0.092185	−1.606862	−0.201621
C	−1.865949	−0.008759	0.556900
C	−2.696552	−0.010946	−0.724945
C	0.124310	1.726033	−1.024385
O	0.872814	1.542901	1.192842
H	−0.784024	2.234368	−0.702776
H	0.893050	2.477774	−1.198481
H	−0.071459	1.200898	−1.960096
H	1.108765	0.924979	1.894389
H	2.497230	−0.198468	0.434214
H	2.439555	0.553426	−1.155820
H	2.089223	−2.156601	−0.877808
H	1.106251	−1.275940	−2.048707
H	−0.591479	−2.394520	−0.490748
H	−2.294717	−0.736787	1.249590
H	−1.934802	0.961741	1.054488
H	−2.347297	0.733991	−1.436402
H	−2.648240	−0.985078	−1.212196
H	−3.742121	0.202436	−0.504599
H	−0.457350	−1.829327	1.755702

Table 99: M06-2X optimized geometry of trans-2,3- aziridine-1-ethyl-2-methylcyclopentan-1-ol (in Å) with the aTZ basis set

Atom	x	y	z
N	0.233235	−1.183307	1.368451
C	0.051907	−1.592464	−0.033333
C	1.313700	−1.437006	−0.848715
C	1.844360	−0.064364	−0.407657
C	0.599247	0.771347	−0.053920
C	−0.414225	−0.266593	0.420080
C	0.883316	1.847063	0.978171
O	0.034760	1.346169	−1.234457
C	−1.871467	0.091803	0.584714
C	−2.675850	0.093230	−0.713188
H	−1.945871	1.068717	1.068593
H	−2.617653	−0.881243	−1.199124
H	−3.725395	0.305988	−0.510163
H	−2.294227	0.837256	−1.406016
H	−2.317819	−0.626019	1.278039
H	−0.655562	−2.381934	−0.250535
H	1.055184	−1.434775	−1.908258
H	2.034007	−2.234958	−0.670333
H	2.430230	0.434008	−1.180401
H	2.468062	−0.173054	0.476663
H	1.201719	1.389295	1.913843
H	1.680227	2.508232	0.629656
H	−0.006718	2.450249	1.156877
H	0.646255	2.008604	−1.567141
H	−0.478550	−1.619240	1.943267

Table 100: M06-2X optimized geometry of cis-2,3- phosphirane-1,2-dimethylcyclopentan-1-ol (not H-bonded) (in Å) with the aTZ basis set

Atom	x	y	z
C	0.980799	−0.012171	0.250281
C	−0.342497	0.592476	−0.228646
C	−1.116033	−0.506080	−0.882842
C	−0.291959	−1.783654	−0.835632
C	0.702068	−1.526441	0.302187
C	−0.321612	1.995483	−0.776954
P	−1.814510	0.014620	0.772125
C	2.104558	0.294218	−0.736960
O	1.302253	0.531572	1.522242
H	3.015319	−0.223877	−0.429279
H	1.848092	−0.043781	−1.741998
H	2.309454	1.362721	−0.767936
H	2.185779	0.244809	1.768490
H	0.247278	−1.754701	1.266634
H	1.615298	−2.114611	0.213264
H	−0.894426	−2.676049	−0.671805
H	0.235572	−1.909166	−1.784264
H	−1.708381	−0.293968	−1.762440
H	0.303164	2.057337	−1.670589
H	−1.324134	2.320108	−1.049596
H	0.072090	2.690013	−0.034291
H	−2.690089	0.977921	0.217574

Table 101: M06-2X optimized geometry of cis-2,3- phosphirane-1,2-dimethylcyclopentan-1-ol (H-bonded) (in Å) with the aTZ basis set

Atom	x	y	z
C	0.970213	−0.000542	0.241997
C	−0.341213	0.600643	−0.293113
C	−1.106732	−0.500377	−0.936697
C	−0.288661	−1.780724	−0.857954
C	0.687456	−1.515967	0.292376
C	−0.307873	1.996827	−0.856023
P	−1.838880	0.038797	0.699716
C	2.129198	0.300835	−0.696988
O	1.351383	0.531907	1.497012
H	3.024120	−0.198784	−0.328703
H	1.914472	−0.055200	−1.705248
H	2.324932	1.370625	−0.733410
H	0.592661	0.475395	2.087749
H	0.218636	−1.754055	1.248980
H	1.604506	−2.098054	0.224826
H	−0.898394	−2.668809	−0.698826
H	0.253762	−1.913845	−1.796838
H	−1.696719	−0.302265	−1.821368
H	0.335616	2.041310	−1.736940
H	−1.303166	2.323515	−1.152092
H	0.078464	2.699706	−0.117216
H	−2.699758	1.010236	0.140930

Table 102: M06-2X optimized geometry of trans-2,3- phosphirane-1,2-dimethylcyclopentan-1-ol (in Å) with the aTZ basis set

Atom	x	y	z
C	−0.300143	0.610637	−0.274278
C	1.029986	−0.008206	0.168457
C	0.703625	−1.497843	0.354353
C	−0.347644	−1.796843	−0.721025
C	−1.136422	−0.501896	−0.814353
C	1.655217	0.641901	1.388486
O	1.891122	0.148323	−0.965505
P	−1.741951	0.154827	0.830193
C	−0.251039	1.967745	−0.925639
H	−1.767271	−0.328134	−1.675076
H	0.140533	−1.980752	−1.679349
H	−0.964121	−2.660911	−0.478227
H	1.601710	−2.108648	0.261003
H	0.292558	−1.665307	1.351310
H	−1.234849	2.246761	−1.300416
H	0.067686	2.734785	−0.218761
H	0.451934	1.956011	−1.758065
H	2.594922	0.146254	1.642962
H	1.866529	1.692241	1.189146
H	0.988408	0.571391	2.246893
H	2.749120	−0.225013	−0.746017
H	−2.605618	1.119228	0.260670

Table 103: M06-2X optimized geometry of cis-2,3- phosphirane-1-ethyl-2-methylcyclopentan-1-ol (not H-bonded) (in Å) with the aTZ basis set

Atom	x	y	z
C	0.406452	−1.473601	0.338795
C	0.637332	0.050294	0.296603
C	−0.680974	0.599965	−0.265632
C	−1.371316	−0.528284	−0.960747
C	−0.502008	−1.770879	−0.858755
C	1.801195	0.427588	−0.633229
C	3.166977	−0.082735	−0.186172
O	0.848444	0.607818	1.585545
C	−0.698697	2.002662	−0.817680
P	−2.185452	−0.037113	0.649365
H	3.212756	−1.169972	−0.140518
H	3.937567	0.246096	−0.882129
H	3.441197	0.312388	0.793456
H	1.578433	0.059640	−1.638423
H	1.840821	1.515120	−0.693014
H	1.660028	0.247516	1.951880
H	−0.106486	−1.708317	1.272143
H	1.332428	−2.044800	0.320090
H	−1.076356	−2.687306	−0.729687
H	0.089439	−1.875811	−1.771615
H	−1.917287	−0.340372	−1.875233
H	−0.048888	2.091552	−1.690484
H	−1.704554	2.282447	−1.125183
H	−0.362259	2.715235	−0.063984
H	−3.066020	0.890274	0.044212

Table 104: M06-2X optimized geometry of cis-2,3- phosphirane-1-ethyl-2-methylcyclopentan-1-ol (H-bonded) (in Å) with the aTZ basis set

Atom	x	y	z
C	−0.365735	1.473612	0.738590
C	−0.569293	−0.057603	0.674323
C	0.480619	−0.527860	−0.348108
C	0.961208	0.660333	−1.101486
C	0.215514	1.895153	−0.618324
O	−0.405743	−0.678596	1.938827
C	−1.991202	−0.439178	0.257356
C	−2.486869	0.185989	−1.040205
C	0.306273	−1.881389	−0.985646
P	2.219276	0.028467	0.128443
H	−1.291354	1.991314	0.987228
H	0.346601	1.688198	1.536734
H	−0.577093	2.131737	−1.328455
H	0.859663	2.769867	−0.538922
H	1.213945	0.571453	−2.149732
H	1.134918	−2.107338	−1.654619
H	0.259724	−2.659777	−0.223368
H	−0.616383	−1.923569	−1.567902
H	0.523365	−0.614946	2.184709
H	−1.800533	0.006075	−1.869426
H	−3.455206	−0.230535	−1.313671
H	−2.612497	1.263864	−0.940404
H	−2.041596	−1.527116	0.201627
H	−2.642270	−0.144347	1.082235
H	2.859034	−0.856811	−0.768171

Table 105: M06-2X optimized geometry of trans-2,3- phosphirane-1-ethyl-2-methylcyclopentan-1-ol (in Å) with the aTZ basis set

Atom	x	y	z
P	1.642051	0.078177	−1.763408
C	0.886774	0.630761	−0.141033
C	−0.473506	−0.011572	0.154774
C	−0.225620	−1.516076	−0.046920
C	1.224086	−1.730629	0.405710
C	1.917096	−0.437962	0.013480
C	−1.616075	0.554396	−0.680958
C	−2.969916	−0.077665	−0.378028
O	−0.692340	0.247664	1.545986
C	1.113391	2.035699	0.353299
H	−0.938333	−2.116627	0.517964
H	−0.343304	−1.772254	−1.101779
H	1.263132	−1.834509	1.491220
H	1.675916	−2.615768	−0.039426
H	2.875291	−0.197781	0.453120
H	2.150853	2.330016	0.200190
H	0.481545	2.750204	−0.175369
H	0.884115	2.097042	1.416799
H	−2.955381	−1.153911	−0.553990
H	−3.275299	0.096277	0.655436
H	−3.745765	0.349221	−1.011709
H	−1.664241	1.630771	−0.503282
H	−1.369308	0.411074	−1.734283
H	−1.487814	−0.216771	1.819084
H	2.637663	1.082772	−1.751906

Table 106: M06-2X optimized geometry of cis-2-ethyl-2,3-phosphirane-methylcyclopentan-1-ol (not H-bonded) (in Å) with the aTZ basis set

Atom	x	y	z
C	−0.319077	1.019309	−0.313250
C	0.328837	−0.367173	−0.200332
C	−0.288421	−1.042676	0.983841
C	−1.275853	−0.086583	1.634995
C	−1.616424	0.895775	0.509225
C	1.798558	−0.507705	−0.563202
C	2.765088	−0.308998	0.604618
P	−0.871730	−1.737393	−0.647896
C	0.569516	2.114903	0.271031
O	−0.559125	1.293407	−1.686474
H	1.518539	2.175994	−0.259006
H	0.060924	3.077262	0.177625
H	0.763990	1.942988	1.329683
H	−0.848511	2.205394	−1.775511
H	−2.392219	0.483631	−0.136616
H	−1.961891	1.862494	0.875283
H	−2.156649	−0.588424	2.033366
H	−0.785832	0.432390	2.462543
H	0.309503	−1.685836	1.614133
H	1.980040	−1.493293	−0.988779
H	2.012770	0.204429	−1.362995
H	2.669304	0.674441	1.059935
H	2.589735	−1.052786	1.382119
H	3.795359	−0.421699	0.267026
H	0.134676	−2.730763	−0.701253

Table 107: M06-2X optimized geometry of cis-2-ethyl-2,3-phosphirane-methylcyclopentan-1-ol (H-bonded) (in Å) with the aTZ basis set

Atom	x	y	z
C	−0.366889	1.000142	−0.268742
C	0.302598	−0.383485	−0.144692
C	−0.290191	−1.058134	1.042858
C	−1.269076	−0.104967	1.712195
C	−1.639229	0.874830	0.594892
C	1.763402	−0.523471	−0.537439
C	2.748124	−0.282747	0.607031
P	−0.910576	−1.766211	−0.571919
C	0.519428	2.118190	0.261331
O	−0.641153	1.352820	−1.612489
H	1.456473	2.170496	−0.290117
H	−0.005737	3.063790	0.132000
H	0.734229	1.977211	1.320349
H	−1.088331	0.608999	−2.030455
H	−2.443666	0.464826	−0.018139
H	−1.970677	1.844345	0.961922
H	−2.135872	−0.611005	2.134686
H	−0.757907	0.415938	2.525354
H	0.314044	−1.710210	1.658301
H	1.945910	−1.519219	−0.938790
H	1.955185	0.171067	−1.358375
H	2.648608	0.712577	1.034079
H	2.593862	−1.006027	1.408033
H	3.773044	−0.394954	0.253820
H	0.094425	−2.754288	−0.669719

Table 108: M06-2X optimized geometry of trans-2-ethyl-2,3-phosphirane-methylcyclopentan-1-ol (in Å) with the aTZ basis set

Atom	x	y	z
C	−0.369613	−0.292152	0.247267
C	0.461477	0.998149	0.195602
C	1.683650	0.626364	−0.659322
C	1.154772	−0.397879	−1.668459
C	0.097500	−1.153207	−0.882246
C	0.825095	1.570601	1.553920
O	−0.353642	1.935002	−0.518739
P	0.652170	−1.767410	0.792516
C	−1.836069	−0.169219	0.629659
C	−2.765092	0.071404	−0.559193
H	−1.933857	0.653980	1.339827
H	−2.744645	−0.775276	−1.245871
H	−3.793177	0.193344	−0.217653
H	−2.470476	0.963005	−1.106264
H	−2.158453	−1.067272	1.155763
H	−0.611715	−1.769278	−1.416555
H	0.671671	0.113553	−2.503052
H	1.938610	−1.040697	−2.066183
H	2.113400	1.511976	−1.127265
H	2.453655	0.178633	−0.028817
H	1.421536	0.861121	2.125460
H	1.407052	2.487489	1.432909
H	−0.075299	1.810781	2.118914
H	0.106393	2.778833	−0.538775
H	−0.471776	−2.603858	0.983731

Table 109: M06-2X optimized geometry of cis-2,3-thiirane- 1,2-dimethylcyclopentan-1-ol (not H-bonded) (in Å) with the aTZ basis set

Atom	x	y	z
C	−0.264769	0.792344	0.051282
C	−1.573866	0.195527	−0.304475
C	−1.415122	−1.307067	−0.447696
C	−0.117036	−1.613830	0.309694
C	0.749479	−0.350913	0.142924
C	1.569732	−0.425242	−1.143833
H	2.227444	−1.295763	−1.107748
H	0.924486	−0.527970	−2.017912
H	2.183552	0.465680	−1.261609
O	1.606038	−0.109521	1.245402
H	2.289896	−0.784318	1.262452
H	−0.317521	−1.751532	1.369342
H	0.387623	−2.502901	−0.067693
H	−2.270350	−1.860387	−0.063906
H	−1.312809	−1.546418	−1.509277
S	−1.488644	0.818386	1.399279
H	−2.252572	0.729639	−0.956327
C	0.189843	2.128738	−0.469831
H	0.525429	2.048278	−1.504954
H	−0.626592	2.845846	−0.424646
H	1.016230	2.504795	0.133122

Table 110: M06-2X optimized geometry of cis-2,3-thiirane- 1,2-dimethylcyclopentan-1-ol (H-bonded) (in Å) with the aTZ basis set

Atom	x	y	z
C	−0.010101	−0.027189	−0.018760
C	1.532801	−0.040359	0.038916
C	1.957035	1.431479	0.045301
C	0.858124	2.135151	−0.726547
C	−0.351105	1.289220	−0.739667
C	−1.743867	1.845534	−0.636744
H	−2.471154	1.103686	−0.966849
H	−1.972789	2.114254	0.395573
H	−1.844365	2.732306	−1.258557
S	0.473835	1.437439	−2.368394
H	0.766544	3.212066	−0.674097
H	1.965010	1.829959	1.063070
H	2.945054	1.593386	−0.381664
H	1.891199	−0.588250	0.908134
H	1.913973	−0.538510	−0.850319
O	−0.546164	−1.156295	−0.676095
H	−0.222153	−1.144187	−1.583875
C	−0.609170	−0.033889	1.379673
H	−0.301001	−0.943344	1.892911
H	−0.264948	0.828280	1.952846
H	−1.696277	−0.017696	1.332357

Table 111: M06-2X optimized geometry of trans-2,3-thiirane- 1,2-dimethylcyclopentan-1-ol (in Å) with the aTZ basis set

Atom	x	y	z
C	0.723434	−0.397437	0.058157
C	−0.217776	−1.593633	0.272143
C	−1.541222	−1.174146	−0.379920
C	−1.576358	0.328869	−0.194862
C	−0.205046	0.823197	0.061451
S	−1.306939	0.918963	1.508815
H	−2.266473	0.928696	−0.772672
H	−1.521586	−1.381581	−1.451542
H	−2.405420	−1.676721	0.050192
H	0.204314	−2.494997	−0.172330
H	−0.354289	−1.773293	1.336185
C	0.314593	2.118089	−0.498511
H	−0.476794	2.864897	−0.506500
H	1.135182	2.500276	0.108870
H	0.680184	1.961919	−1.512322
C	1.858880	−0.321683	1.059715
H	2.463670	−1.230057	1.014354
H	2.502702	0.529310	0.838788
H	1.467096	−0.221684	2.071013
O	1.237539	−0.424139	−1.277892
H	1.825028	−1.180186	−1.360013

Table 112: M06-2X optimized geometry of cis-2,3-thiirane- 1-ethyl-2-methylcyclopentan-1-ol (not H-bonded) (in Å) with the aTZ basis set

Atom	x	y	z
C	−0.943863	0.593268	−0.036660
C	−1.738503	−0.551637	−0.540220
C	−0.891442	−1.808212	−0.480185
C	0.214714	−1.458408	0.522735
C	0.437948	0.058740	0.362110
C	1.446518	0.373231	−0.754939
C	2.868453	−0.097821	−0.470634
H	2.934295	−1.177866	−0.346056
H	3.527623	0.175797	−1.293268
H	3.269523	0.376186	0.426310
H	1.086276	−0.068594	−1.688383
H	1.463538	1.453636	−0.896874
O	0.833626	0.695500	1.564641
H	1.644815	0.290105	1.880865
H	−0.125022	−1.643117	1.539027
H	1.122184	−2.034917	0.355418
H	−1.460862	−2.689412	−0.189938
H	−0.472595	−1.993998	−1.472611
S	−2.293973	0.073973	1.071286
H	−2.435329	−0.409917	−1.355890
C	−1.062397	1.975788	−0.621223
H	−0.550210	2.039739	−1.582145
H	−2.110016	2.226357	−0.771191
H	−0.623771	2.709475	0.055055

Table 113: M06-2X optimized geometry of cis-2,3-thiirane- 1-ethyl-2-methylcyclopentan-1-ol (H-bonded) (in Å) with the aTZ basis set

Atom	x	y	z
C	0.405520	0.098824	0.699368
C	−0.695700	0.623628	−0.239987
C	−1.379902	−0.536067	−0.843633
C	−0.719292	−1.819323	−0.381465
C	0.086854	−1.406853	0.857743
H	0.996718	−1.992621	0.981140
H	−0.511184	−1.538330	1.757152
H	−0.066153	−2.175071	−1.180035
H	−1.442596	−2.604971	−0.170235
S	−2.379008	0.316489	0.422619
H	−1.785092	−0.467914	−1.844959
C	−0.499538	1.933806	−0.951251
H	−1.390492	2.192800	−1.519100
H	−0.306099	2.726031	−0.227907
H	0.349219	1.882004	−1.634762
O	0.411467	0.784371	1.936073
H	−0.477839	0.725140	2.303449
C	1.798163	0.341424	0.116359
C	2.084725	−0.372926	−1.198626
H	1.334539	−0.149435	−1.959315
H	3.053627	−0.068437	−1.591161
H	2.111497	−1.454409	−1.066790
H	1.933660	1.417981	0.005961
H	2.510707	0.017906	0.876873

Table 114: M06-2X optimized geometry of trans-2,3-thiirane- 1-ethyl-2-methylcyclopentan-1-ol (in Å) with the aTZ basis set

Atom	x	y	z
C	−0.466940	−0.006116	0.141286
C	0.891394	0.651048	−0.135402
C	1.918577	−0.410656	−0.050091
C	1.251171	−1.725747	0.292221
C	−0.218314	−1.506811	−0.090099
H	−0.898322	−2.111063	0.510019
H	−0.384338	−1.753384	−1.137113
H	1.345365	−1.881365	1.368697
H	1.699544	−2.577160	−0.216296
S	1.627673	0.203596	−1.741073
H	2.898121	−0.182192	0.347785
C	1.124778	2.050027	0.364243
H	2.157286	2.342140	0.183098
H	0.476879	2.759361	−0.150538
H	0.912427	2.100683	1.431166
C	−1.606258	0.575197	−0.685459
C	−2.953261	−0.083581	−0.410859
H	−2.924052	−1.152526	−0.624598
H	−3.264088	0.049568	0.627174
H	−3.731987	0.356181	−1.031875
H	−1.669206	1.644681	−0.474131
H	−1.346588	0.463826	−1.739407
O	−0.675332	0.235567	1.536764
H	−1.466137	−0.235771	1.811248

Table 115: M06-2X optimized geometry of cis-2-ethyl-2,3- thiirane-1-methylcyclopentan-1-ol (not H-bonded) (in Å) with the aTZ basis set

Atom	x	y	z
C	−0.470879	−0.221282	0.059249
C	0.004565	−1.391045	−0.718593
C	1.450433	−1.170126	−1.123579
C	1.946858	−0.099935	−0.144357
C	0.705845	0.757468	0.168242
C	0.560945	1.879938	−0.858796
H	1.447909	2.515642	−0.828180
H	0.469742	1.480490	−1.870197
H	−0.310328	2.494712	−0.642409
O	0.718232	1.303913	1.475769
H	1.407643	1.971225	1.527502
H	2.287727	−0.558076	0.780674
H	2.758496	0.499853	−0.555603
H	2.041661	−2.083546	−1.088853
H	1.471715	−0.798471	−2.151326
S	−0.253784	−1.738508	1.043838
H	−0.666033	−1.908404	−1.392102
C	−1.859740	0.367204	−0.070074
C	−3.007844	−0.630109	−0.025312
H	−3.082041	−1.101042	0.951821
H	−3.948433	−0.124231	−0.239590
H	−2.877864	−1.422122	−0.763753
H	−1.980348	1.110109	0.721640
H	−1.897886	0.910959	−1.017651

Table 116: M06-2X optimized geometry of cis-2-ethyl-2,3- thiirane-1-methylcyclopentan-1-ol (H-bonded) (in Å) with the aTZ basis set

Atom	x	y	z
C	−1.856495	0.007932	0.573463
C	−0.381334	−0.315319	0.442166
C	0.121453	−1.575717	−0.140197
C	1.375276	−1.292975	−0.944579
C	1.886375	0.036057	−0.380900
C	0.628462	0.797616	0.093189
C	0.104783	1.704242	−1.010087
H	−0.816466	2.198123	−0.706036
H	0.853524	2.469485	−1.209579
H	−0.074188	1.140970	−1.926153
O	0.880650	1.648033	1.192615
H	1.161436	1.093077	1.929213
H	2.534838	−0.141519	0.474393
H	2.446983	0.617115	−1.110588
H	2.108955	−2.094474	−0.880827
H	1.087445	−1.186598	−1.993894
S	0.314702	−1.491825	1.670759
H	−0.569776	−2.338130	−0.473853
H	−2.298571	−0.688046	1.284045
H	−1.946798	1.003097	1.014256
C	−2.637017	−0.073437	−0.738822
H	−2.284663	0.640414	−1.479394
H	−2.561451	−1.071275	−1.171564
H	−3.692309	0.128460	−0.558180

Table 117: M06-2X optimized geometry of trans-2-ethyl-2,3- thiirane-1-methylcyclopentan-1-ol (in Å) with the aTZ basis set

Atom	x	y	z
C	−1.856495	0.007932	0.573463
C	−0.381334	−0.315319	0.442166
C	0.121453	−1.575717	−0.140197
C	1.375276	−1.292975	−0.944579
C	1.886375	0.036057	−0.380900
C	0.628462	0.797616	0.093189
C	0.104783	1.704242	−1.010087
H	−0.816466	2.198123	−0.706036
H	0.853524	2.469485	−1.209579
H	−0.074188	1.140970	−1.926153
O	0.880650	1.648033	1.192615
H	1.161436	1.093077	1.929213
H	2.534838	−0.141519	0.474393
H	2.446983	0.617115	−1.110588
H	2.108955	−2.094474	−0.880827
H	1.087445	−1.186598	−1.993894
S	0.314702	−1.491825	1.670759
H	−0.569776	−2.338130	−0.473853
H	−2.298571	−0.688046	1.284045
H	−1.946798	1.003097	1.014256
C	−2.637017	−0.073437	−0.738822
H	−2.284663	0.640414	−1.479394
H	−2.561451	−1.071275	−1.171564
H	−3.692309	0.128460	−0.558180

Table 118: M06-2X/aug-cc-pVTZ Frequencies and IR Intensities of cis-2,3-epoxy-1,2-dimethylcyclopentan-1-ol (not H-bonded)

Frequency	IR Intensity
128.9	0.8
186.1	2.7
210.8	95.1
213.6	1.1
240.6	5.0
267.9	0.4
312.3	0.7
363.2	1.8
380.4	2.5
422.6	1.6
481.6	12.6
534.8	13.2
587.4	6.0
661.6	1.6
691.2	4.0
812.5	3.5
853.1	7.1
893.5	17.2
944.2	6.4
949.6	2.8
955.4	9.4
994.2	1.9
1011.3	6.2
1045.3	7.8
1087.0	15.1
1101.5	61.3
1127.3	12.2
1168.3	18.2
1210.0	12.1
1226.3	37.4
1241.5	28.4
1258.7	24.0
1304.6	6.7
1333.0	3.9
1343.0	11.0
1400.3	10.8
1404.7	11.6
1412.5	8.7
1474.1	4.8
1484.5	7.0
1490.1	7.0
1495.4	10.6
1499.9	3.4
1507.8	8.2
1520.8	5.3
3054.7	11.5
3070.2	15.4
3071.8	20.3
3090.9	26.0
3117.5	16.1
3122.0	22.1
3138.4	11.8
3147.0	10.7
3150.7	10.8
3155.1	10.5
3176.0	19.4
3867.8	34.6

Table 119: M06-2X/aug-cc-pVTZ Frequencies and IR Intensities of cis-2,3-epoxy-1,2-dimethylcyclopentan-1-ol (H-bonded)

Frequency	IR Intensity
112.9	3.0
196.1	0.1
213.9	1.3
232.4	1.3
266.9	0.7
308.8	0.7
354.9	4.5
373.1	3.4
413.6	49.1
434.3	32.9
482.3	26.6
539.5	14.2
594.3	5.3
665.0	2.5
687.2	4.4
809.9	0.6
846.1	5.8
895.7	14.9
943.8	8.3
951.8	4.3
962.7	5.6
994.8	14.9
1014.6	8.4
1046.2	28.1
1083.4	13.5
1116.7	5.9
1126.0	24.4
1169.7	38.0
1182.6	2.5
1227.1	51.2
1246.6	9.0
1294.5	18.2
1313.8	1.9
1335.9	2.9
1351.7	0.8
1387.7	38.3
1411.0	11.4
1426.7	30.3
1476.4	3.6
1483.3	7.5
1487.3	9.4
1494.2	10.3
1499.7	3.2
1509.3	4.7
1522.1	4.6
3065.5	8.2
3068.2	14.8
3073.4	21.9
3097.0	15.1
3118.3	18.4
3136.5	12.4
3140.7	9.9
3144.9	14.2
3150.5	19.9
3155.2	7.6
3180.0	18.4
3840.1	34.8

Table 120: M06-2X/aug-cc-pVTZ Frequencies and IR Intensities of trans-2,3-epoxy-1,2-dimethylcyclopentan-1-ol

Frequency	IR Intensity
118.3	2.3
184.2	0.6
223.9	4.8
242.5	1.8
264.3	0.3
270.0	38.8
281.4	61.4
354.0	1.6
381.7	8.1
425.1	1.2
481.7	14.7
513.7	1.8
600.2	2.3
654.7	1.3
702.1	2.1
813.9	3.9
854.2	4.4
887.5	8.7
937.0	3.7
951.5	22.1
959.1	13.6
992.7	2.1
1016.7	6.0
1041.9	6.6
1090.7	5.6
1094.8	84.9
1125.3	12.0
1164.4	1.4
1209.5	5.1
1228.6	69.3
1240.3	12.3
1257.6	3.7
1302.8	5.4
1339.0	5.3
1351.2	3.2
1401.5	2.6
1405.5	18.8
1415.3	20.3
1474.2	2.5
1483.3	5.9
1487.2	14.0
1492.0	4.8
1495.4	6.5
1506.3	2.0
1520.2	7.3
3053.0	15.9
3068.3	16.4
3081.9	24.7
3092.2	25.5
3122.4	13.1
3124.3	12.1
3136.9	11.3
3142.3	6.7
3146.7	21.8
3162.8	5.4
3183.9	18.4
3869.3	34.5

Table 121: M06-2X/aug-cc-pVTZ Frequencies and IR Intensities of cis-2,3-epoxy-1-ethyl-2-methylcyclopentan-1-ol (not H-bonded)

Frequency	IR Intensity
72.9	0.4
122.6	0.9
171.5	8.5
198.3	56.3
205.0	19.6
224.0	9.4
270.4	19.7
285.5	1.5
321.9	1.2
356.1	1.3
392.3	2.9
423.4	4.3
484.1	10.0
545.8	12.1
590.8	5.0
662.5	1.8
707.4	2.5
791.8	1.0
803.6	5.4
853.4	7.3
893.8	16.7
944.8	1.0
950.6	16.0
980.9	6.8
1003.4	17.6
1030.7	1.9
1036.9	14.5
1051.6	19.5
1091.8	16.0
1104.7	19.7
1125.0	17.1
1165.1	15.6
1194.7	9.2
1233.2	57.2
1237.4	5.1
1248.9	20.5
1300.7	1.3
1318.5	2.6
1333.5	5.0
1344.5	9.5
1365.8	5.1
1399.3	8.0
1409.7	5.9
1421.5	5.7
1474.9	1.4
1483.7	11.0
1484.0	8.3
1495.2	9.3
1498.1	4.4
1505.8	5.9
1516.1	8.9
1518.6	6.5
3056.8	11.3
3066.4	19.0
3070.9	16.1
3072.7	23.0
3102.2	18.3
3105.6	11.0
3118.2	15.1
3132.2	22.4
3138.3	24.7
3142.4	11.5
3154.8	11.6
3155.5	12.8
3177.5	20.5
3876.1	33.1

Table 122: M06-2X/aug-cc-pVTZ Frequencies and IR Intensities of cis-2,3-epoxy-1-ethyl-2-methylcyclopentan-1-ol (H-bonded)

Frequency	IR Intensity
132.2	1.7
143.4	1.2
197.7	0.3
224.0	0.4
244.3	0.7
257.6	1.8
264.5	1.3
313.0	1.0
373.7	5.1
402.3	5.0
432.6	49.9
453.8	20.4
483.9	37.2
533.1	9.5
615.9	5.2
651.0	0.7
691.0	5.6
789.4	0.4
821.1	0.8
842.2	5.3
894.6	13.2
941.4	3.0
946.8	23.5
977.0	13.4
1006.1	1.2
1025.6	13.4
1053.5	23.5
1074.5	18.3
1087.1	18.6
1122.1	3.6
1130.5	32.8
1169.2	22.1
1182.4	8.6
1202.7	35.6
1246.3	1.3
1271.5	3.9
1293.7	9.2
1326.0	2.9
1335.7	5.7
1344.8	2.6
1382.1	21.2
1408.6	25.3
1410.4	13.5
1418.9	4.0
1475.6	3.9
1482.9	10.7
1493.2	12.7
1497.2	3.4
1500.4	2.1
1507.5	10.7
1517.1	5.5
1521.6	7.7
3062.4	9.4
3066.7	10.6
3070.5	23.2
3079.3	25.8
3095.6	16.9
3104.0	2.5
3118.4	14.6
3129.6	31.8
3134.9	8.0
3139.2	33.0
3142.3	11.8
3154.0	9.2
3176.4	18.0
3837.8	35.4

Table 123: Frequencies and IR Intensities of trans-2,3-epoxy-1-ethyl-2-methylcyclopentan-1-ol.

Frequency	IR Intensity
99.5	0.2
117.2	2.2
169.7	1.2
192.8	1.1
237.3	8.0
246.4	3.9
272.4	49.2
275.3	48.1
302.2	3.3
362.0	1.8
404.6	3.3
436.8	3.7
489.8	11.7
521.3	2.3
588.2	1.6
677.7	0.6
705.2	2.4
786.9	4.2
825.7	3.1
850.7	6.6
894.1	10.2
945.5	4.9
966.7	20.6
982.5	7.2
991.1	24.8
1024.9	27.7
1033.4	1.5
1047.8	2.5
1092.1	5.5
1100.0	38.0
1126.1	17.4
1166.4	1.8
1204.6	2.4
1224.2	66.2
1239.3	5.3
1248.2	9.2
1301.3	5.3
1319.0	5.5
1335.9	7.1
1349.8	1.8
1360.9	3.3
1404.2	14.1
1410.6	3.8
1426.8	15.6
1475.5	2.2
1482.6	8.2
1485.4	5.7
1492.9	3.7
1493.7	7.8
1504.8	7.8
1513.2	5.1
1521.0	8.2
3056.4	12.7
3062.9	17.4
3069.6	20.6
3080.7	26.8
3089.7	20.1
3100.8	2.4
3122.4	7.9
3123.9	29.6
3137.4	8.1
3138.3	26.7
3140.5	18.9
3162.4	5.5
3183.8	19.6
3875.3	31.0

Table 124: M06-2X/aug-cc-pVTZ Frequencies and IR Intensities of cis-2-ethyl-2,3- epoxy-1-methylcyclopentan-1-ol (not H-bonded)

Frequency	IR Intensity
85.6	0.3
116.6	1.1
184.8	6.5
192.6	66.9
201.7	25.5
233.5	1.1
276.6	2.3
303.8	1.3
320.7	0.9
364.3	1.7
405.2	3.0
442.8	2.7
486.6	13.1
556.0	4.0
599.9	15.8
650.7	1.9
697.3	2.0
773.6	2.5
815.2	3.9
869.4	11.5
922.3	5.3
947.8	2.5
957.7	16.3
968.0	7.2
981.4	10.9
1016.2	8.7
1025.4	18.5
1058.3	4.7
1090.0	14.8
1112.1	36.6
1122.6	5.5
1167.0	26.2
1209.6	7.9
1217.0	42.3
1239.5	14.4
1243.1	27.5
1293.2	8.2
1308.9	1.1
1332.0	6.6
1341.5	11.1
1370.6	1.3
1404.7	17.4
1409.2	0.7
1413.2	6.7
1478.5	7.2
1483.7	0.6
1494.1	7.3
1498.1	9.9
1499.8	8.8
1503.8	0.8
1509.4	10.3
1514.9	7.3
3052.8	11.0
3068.4	17.6
3072.7	25.8
3073.4	11.0
3090.7	25.5
3114.8	0.5
3117.6	15.6
3120.9	31.4
3135.2	24.4
3141.9	17.7
3146.3	17.8
3150.5	10.4
3177.5	19.1
3868.6	34.6

Table 125: Frequencies and IR Intensities of cis-2-ethyl-2,3-epoxy-1-methylcyclopentan-1-ol (H-bonded)

Frequency	IR Intensity
95.8	1.0
105.0	1.9
173.5	0.4
201.8	0.2
228.7	2.3
273.6	0.3
300.7	1.7
318.7	1.0
359.5	2.7
396.4	27.7
415.1	58.2
442.8	4.9
485.0	26.6
558.7	5.9
605.4	12.1
654.5	3.7
693.8	2.8
776.2	2.8
814.9	1.2
864.2	7.9
925.6	1.3
949.9	2.0
956.8	19.9
967.9	5.8
980.2	14.6
1017.9	28.3
1027.2	13.1
1059.3	22.3
1091.1	5.9
1112.1	7.7
1126.2	11.9
1171.6	32.6
1181.0	2.1
1225.2	50.0
1240.9	12.6
1267.3	1.3
1299.5	14.5
1316.0	8.2
1335.8	2.1
1350.5	0.2
1372.2	6.7
1391.4	27.7
1413.2	5.9
1425.8	32.1
1480.1	9.4
1481.6	2.4
1490.3	4.9
1498.4	15.0
1499.1	3.0
1505.9	0.6
1511.5	7.0
1516.4	5.0
3063.5	7.1
3066.3	10.2
3070.9	23.1
3072.7	23.9
3097.9	15.1
3110.2	2.4
3118.2	19.1
3135.9	22.9
3140.1	16.6
3143.6	16.2
3146.6	32.5
3150.2	5.0
3176.0	18.3
3840.6	35.6

Table 126: Frequencies and IR Intensities of trans-2-ethyl-2,3-epoxy-1-methylcyclopentan-1-ol

Frequency	IR Intensity
40.6	0.0
119.0	1.9
175.0	1.3
214.5	1.4
226.8	3.9
259.4	0.9
272.0	47.7
276.4	46.7
306.3	1.5
377.4	8.3
396.0	3.9
432.9	1.8
482.8	14.4
554.3	5.4
596.7	2.6
647.3	2.8
705.5	0.2
776.5	3.2
818.2	1.9
871.0	5.7
910.0	2.0
940.4	4.2
957.9	6.4
970.9	31.4
998.6	3.8
1008.6	21.2
1028.2	15.4
1063.7	11.7
1091.2	22.5
1103.5	37.4
1127.5	4.5
1163.9	1.8
1207.3	5.5
1224.4	49.6
1238.6	19.6
1242.9	10.0
1292.3	5.2
1314.0	7.4
1336.0	6.8
1351.1	3.1
1368.1	1.4
1403.5	8.4
1410.6	1.2
1419.4	22.3
1473.6	3.1
1485.0	4.5
1491.6	4.3
1496.5	7.0
1497.4	12.5
1500.2	5.2
1506.2	1.5
1518.9	6.3
3053.5	14.4
3062.6	16.9
3069.2	29.1
3079.6	24.5
3090.9	25.4
3104.1	11.9
3122.3	7.0
3123.3	18
3131.5	31.4
3142.8	8.5
3148.7	19.0
3168.3	8.0
3178.5	17.7
3865.4	32.8

Table 127: M06-2X/aug-cc-pVTZ Frequencies and IR Intensities of cis-2,3- aziridine-1,2-dimethylcyclopentan-1-ol (not H-bonded)

Frequency	IR Intensity
132.6	1.3
167.5	97.5
194.9	2.4
218.0	4.1
243.1	0.8
275.7	1.0
327.9	0.7
367.4	1.9
382.5	1.4
426.2	5.1
481.4	7.9
522.6	17.3
587.0	4.2
655.7	1.6
671.8	2.4
812.4	2.0
854.4	0.6
873.7	26.0
930.5	5.6
948.8	7.5
956.8	13.6
976.5	5.5
1005.1	9.1
1017.3	34.6
1053.2	24.1
1091.1	21.9
1093.9	13.3
1128.2	31.0
1152.9	25.7
1165.0	14.7
1209.4	19.4
1240.1	23.9
1246.1	18.4
1294.1	16.5
1315.7	18.3
1338.3	1.5
1353.5	9.6
1403.1	23.1
1410.5	4.6
1417.4	4.4
1469.5	6.5
1482.7	5.0
1489.2	9.3
1492.9	7.4
1500.3	4.0
1504.1	6.5
1518.2	0.7
3054.6	12.6
3059.9	20.7
3069.8	27.9
3088.8	30.9
3114.0	19.4
3121.8	23.8
3128.6	13.7
3133.0	14.8
3146.4	15.9
3152.3	10.1
3183.6	16.1
3544.4	3.5
3869.9	31.8

Table 128: M06-2X/aug-cc-pVTZ Frequencies and IR Intensities of cis-2,3- aziridine-1,2-dimethylcyclopentan-1-ol (H-bonded)

Frequency	IR Intensity
118.3	2.2
210.7	0.0
219.3	0.3
230.0	1.0
272.7	0.7
320.4	1.0
355.5	5.7
377.2	2.0
424.3	13.7
446.9	55.7
482.4	31.3
527.9	16.3
594.3	4.1
660.5	8.5
668.8	1.0
813.1	0.2
846.7	0.4
877.8	15.2
930.9	10.0
947.2	11.1
961.2	9.3
980.7	1.8
1007.9	10.8
1023.4	57.0
1054.7	24.9
1090.4	4.9
1110.4	8.4
1132.7	21.5
1136.9	10.5
1180.9	2.1
1202.3	18.2
1232.1	82.7
1252.0	0.5
1304.4	9.9
1337.8	3.8
1352.0	0.6
1358.7	4.9
1392.7	34.9
1414.6	9.6
1428.8	39.4
1472.8	4.7
1483.9	7.2
1488.2	7.3
1490.4	8.4
1501.5	2.2
1504.1	7.0
1519.7	1.0
3056.8	19.4
3063.9	11.3
3070.3	28.8
3096.7	17.1
3114.3	20.8
3124.9	12.9
3132.4	12.6
3138.6	17.3
3144.4	17.9
3149.7	16.1
3186.0	15.6
3540.5	3.8
3828.6	32.0

Table 129: M06-2X/aug-cc-pVTZ Frequencies and IR Intensities of trans-2,3- aziridine-1,2-dimethylcyclopentan-1-ol

Frequency	IR Intensity
124.9	1.4
199.6	0.3
227.7	6.5
245.1	1.9
256.4	85.8
259.0	3.2
286.4	9.2
352.4	1.1
385.7	8.9
432.2	0.4
476.6	17.4
501.8	1.7
600.5	4.5
649.0	0.1
685.0	0.2
815.4	3.0
853.6	1.7
873.2	15.3
925.1	6.8
946.1	15.5
956.5	11.3
975.3	18.8
1005.1	10.5
1024.1	44.3
1049.3	36.2
1092.7	13.5
1098.7	15.3
1130.0	9.4
1143.3	6.2
1169.3	5.8
1209.8	6.5
1237.7	24.5
1246.3	62.6
1295.5	13.9
1315.4	9.7
1347.4	1.6
1358.1	2.9
1401.0	11.7
1409.1	11.6
1421.8	11.6
1473.2	3.4
1478.9	8.8
1484.7	8.0
1493.3	1.0
1495.0	6.8
1504.5	3.2
1513.6	3.7
3052.1	18.9
3056.2	25.0
3078.8	29.5
3088.5	32.2
3117.0	21.9
3120.8	12.1
3121.7	15.2
3142.7	3.7
3147.3	22.6
3152.0	5.9
3191.1	15.2
3541.8	3.5
3872.1	32.8

Table 130: M06-2X/aug-cc-pVTZ Frequencies and IR Intensities of cis-2,3-aziridine-1-ethyl-2-methylcyclopentan-1-ol (not H-bonded)

Frequency	IR Intensity
83.7	0.2
117.0	0.1
190.8	0.1
218.8	4.6
227.3	0.6
235.6	38.3
279.6	28.8
296.8	26.6
332.2	1.2
365.5	4.2
391.5	8.3
427.6	0.6
489.5	12.1
531.7	4.5
595.1	3.9
654.0	4.0
698.7	0.8
793.0	1.8
808.9	6.7
843.0	17.1
878.3	16.1
919.7	24.1
952.3	4.0
980.9	8.6
1007.3	11.2
1022.6	37.5
1028.2	5.5
1047.3	12.4
1082.8	14.7
1104.0	8.0
1115.1	40.8
1125.5	5.7
1131.9	13.3
1186.9	5.6
1203.5	2.0
1229.8	49.9
1251.7	3.4
1280.9	51.3
1306.2	2.5
1323.6	3.3
1334.6	13.5
1341.5	0.4
1365.1	4.2
1400.4	12.5
1409.5	5.2
1420.9	3.0
1469.8	5.2
1481.7	10.2
1495.0	6.1
1500.0	9.0
1504.0	4.4
1507.4	4.6
1512.3	0.2
1520.5	10.7
3057.3	7.5
3063.7	16.6
3064.3	26.5
3067.2	32.9
3088.6	10.6
3105.8	24.7
3110.4	1.9
3128.1	25.0
3132.1	1.4
3134.6	37.3
3143.2	23.0
3153.1	11.5
3179.0	20.6
3543.3	3.3
3872.9	31.3

Table 131: M06-2X/aug-cc-pVTZ Frequencies and IR Intensities of cis-2,3-aziridine-1-ethyl-2-methylcyclopentan-1-ol (H-bonded)

Frequency	IR Intensity
139.3	1.3
152.1	0.3
201.7	0.4
221.8	0.3
252.1	0.7
266.6	1.6
269.2	0.3
328.5	0.6
377.4	3.8
406.7	7.1
443.3	10.3
464.3	18.9
495.4	70.6
522.2	10.9
616.2	4.5
647.9	3.3
671.9	4.3
791.7	0.5
824.2	1.4
843.0	1.2
877.2	13.6
930.8	14.0
948.1	8.1
971.5	32.9
984.5	2.9
1020.0	16.2
1036.1	27.0
1053.5	30.3
1076.0	19.8
1094.3	5.0
1119.2	5.7
1130.4	29.0
1142.4	13.0
1174.6	3.3
1198.8	6.2
1209.4	56.1
1251.8	4.8
1288.2	1.6
1305.0	5.5
1333.3	1.2
1343.6	8.9
1358.6	5.3
1387.8	15.2
1412.1	13.2
1415.5	26.6
1421.0	12.9
1473.0	7.0
1484.1	7.4
1488.5	8.7
1497.0	6.1
1500.5	3.9
1506.4	10.7
1512.9	5.1
1522.1	3.3
3056.4	18.9
3062.2	10.2
3069.8	21.0
3076.2	33.2
3094.4	19.3
3103.0	3.4
3115.5	18.7
3124.0	10.7
3128.5	41.0
3133.9	7.8
3137.4	32.9
3142.8	15.4
3184.4	14.8
3538.5	3.9
3822.8	33.8

Table 132: M06-2X/aug-cc-pVTZ Frequencies and IR Intensities of trans-2,3-aziridine-1-ethyl-2-methylcyclopentan-1-ol

Frequency	IR Intensity
97.7	0.2
115.1	1.0
172.6	0.5
193.2	0.7
237.7	10.6
242.2	0.9
283.2	80.2
285.6	4.5
307.2	10.7
362.7	4.0
416.1	3.9
442.8	6.7
489.6	3.0
499.2	1.9
588.0	1.2
667.6	5.9
696.8	5.1
784.5	2.8
830.8	5.9
833.4	19.9
883.1	10.1
920.0	23.1
961.7	17.6
984.6	2.9
989.3	28.5
1016.7	11.1
1028.3	20.7
1040.7	7.9
1085.4	27.5
1091.3	14.3
1111.5	19.3
1118.7	1.2
1132.4	1.8
1190.4	4.6
1213.3	44.4
1222.0	25.6
1241.7	3.5
1271.2	12.9
1316.1	11.0
1318.1	3.2
1335.3	8.0
1342.6	4.6
1361.2	0.9
1403.5	15.4
1408.5	4.4
1421.5	12.4
1471.9	3.5
1481.8	5.0
1488.1	6.7
1492.3	2.1
1502.2	9.0
1504.3	6.2
1506.8	4.6
1514.4	5.1
3056.5	2.9
3060.6	28.2
3066.5	24.5
3071.8	26.4
3077.5	24.2
3099.1	4.9
3112.2	7.1
3123.8	31.8
3125.1	28.2
3134.2	11.4
3137.7	25.8
3161.6	6.7
3186.6	18.9
3532.6	0.9
3874.3	27.6

Table 133: M06-2X/aug-cc-pVTZ Frequencies and IR Intensities of cis-2-ethyl-2,3-aziridine-1-methylcyclopentan-1-ol (not H-bonded)

Frequency	IR Intensity
91.4	0.5
109.5	0.3
180.9	0.7
207.5	2.0
244.1	4.6
251.9	78.7
281.3	6.9
299.9	5.9
347.7	0.4
372.3	3.1
419.7	1.2
441.8	4.3
485.5	11.3
546.8	2.3
598.3	6.2
645.6	2.6
685.9	0.9
773.3	6.8
820.0	4.4
858.5	20.1
898.6	1.8
928.7	35.3
947.7	4.8
966.1	14.9
987.2	7.3
1000.4	22.6
1026.3	3.3
1056.9	2.0
1088.2	16.0
1103.2	7.8
1117.8	50.3
1120.9	22.1
1136.3	9.6
1192.8	6.9
1209.1	2.8
1227.9	71.4
1245.4	4.6
1261.3	9.5
1306.2	21.4
1321.5	4.8
1326.9	12.4
1339.3	3.0
1370.3	6.3
1405.6	4.8
1407.4	15.7
1411.6	8.5
1463.7	5.6
1486.0	2.8
1494.2	2.1
1497.3	3.6
1501.8	9.5
1510.1	5.3
1514.0	2.8
1517.2	10.5
3051.0	13.4
3063.5	14.5
3067.1	25.5
3068.1	28.5
3084.2	16.2
3105.1	18.1
3113.6	0.6
3118.5	31.5
3127.6	22.3
3130.9	25.7
3145.4	25.4
3147.8	11.5
3180.5	18.1
3540.5	3.3
3866.5	33.2

Table 134: M06-2X/aug-cc-pVTZ Frequencies and IR Intensities of cis-2-ethyl-2,3-aziridine-1-methylcyclopentan-1-ol (H-bonded)

Frequency	IR Intensity
96.8	0.6
108.6	1.1
174.7	0.4
210.3	0.3
241.3	1.0
276.5	0.3
301.1	2.4
337.2	0.5
364.5	2.3
409.0	3.4
440.3	2.8
464.5	33.2
493.4	71.7
547.7	5.9
603.6	6.2
652.4	8.4
676.5	3.9
772.7	3.4
816.5	1.1
863.3	1.9
889.8	11.4
941.3	22.7
948.2	15.0
967.8	12.7
984.0	2.2
1011.3	3.6
1021.8	21.1
1030.0	38.2
1073.6	20.3
1096.8	3.8
1108.7	11.3
1123.7	5.2
1139.9	13.4
1177.0	5.5
1200.7	19.4
1232.0	84.7
1249.2	0.8
1272.7	0.3
1314.1	10.9
1335.8	7.3
1349.5	1.5
1363.3	10.7
1373.5	5.0
1399.5	24.6
1413.6	6.5
1430.5	45.8
1471.1	4.8
1481.9	3.5
1487.1	4.8
1490.0	9.0
1499.8	7.1
1504.8	2.5
1511.0	3.5
1515.5	3.9
3052.6	20.8
3063.9	9.3
3068.6	26.0
3070.4	26.3
3089.0	9.2
3097.7	17.5
3114.0	22.9
3133.1	25.3
3140.2	18.1
3143.5	14.6
3146.8	23.3
3156.8	12.5
3183.6	15.3
3538.8	3.8
3823.9	33.1

Table 135: M06-2X/aug-cc-pVTZ Frequencies and IR Intensities of trans-2-ethyl-2,3-aziridine-1-methylcyclopentan-1-ol

Frequency	IR Intensity
45.7	0.1
122.0	1.0
182.8	0.7
228.0	0.3
234.8	4.5
262.7	0.6
268.5	0.3
305.7	70.0
311.0	19.3
382.1	13.0
402.7	5.5
438.8	0.3
476.9	16.6
541.1	4.0
598.1	4.5
642.7	0.9
690.5	0.8
771.2	3.6
818.3	3.4
868.7	2.7
885.4	13.4
930.9	13.1
947.6	25.5
964.9	30.4
988.9	1.1
1004.9	6.1
1023.4	25.2
1031.7	22.5
1077.5	15.5
1096.7	23.5
1105.5	12.3
1124.5	3.0
1138.0	1.6
1168.5	7.0
1208.0	7.0
1237.4	15.9
1243.8	76.6
1265.2	0.6
1312.3	11.7
1323.3	12.6
1348.8	4.3
1357.1	2.6
1369.8	3.4
1400.7	13.7
1412.8	7.9
1428.7	11.9
1468.1	3.0
1479.4	7.4
1481.0	2.7
1494.0	2.8
1495.5	4.9
1499.3	4.2
1504.2	4.8
1516.8	4.8
3047.3	25.4
3052.6	19.2
3068.1	27.1
3077.4	29.2
3084.9	18.8
3088.6	35.0
3120.5	8.7
3121.0	21.7
3128.6	33.2
3144.6	4.7
3149.2	21.4
3175.3	6.2
3187.0	14.8
3544.3	3.5
3866.5	30.8

Table 136: M06-2X/aug-cc-pVTZ Frequencies and IR Intensities of cis-2,3-phosphirane-1,2-dimethylcyclopentan-1-ol (not H-bonded)

Frequency	IR Intensity
119.3	0.1
206.6	1.9
211.9	1.7
236.2	12.5
253.7	77.6
278.2	1.2
308.8	0.8
324.0	5.8
355.0	0.0
383.2	0.9
428.9	3.5
481.7	10.3
559.9	1.7
595.7	0.2
607.7	0.9
651.1	1.5
715.5	1.7
765.8	1.5
837.1	4.2
893.3	8.1
921.4	9.4
945.2	8.4
957.5	1.2
977.6	0.2
989.2	7.2
1013.7	8.3
1052.4	3.9
1085.7	19.3
1104.3	27.0
1140.4	22.5
1175.6	32.1
1203.4	8.5
1233.9	49.7
1236.8	8.4
1261.4	4.3
1322.8	18.3
1329.9	13.6
1366.1	2.2
1393.9	24.1
1403.0	9.6
1412.0	4.2
1487.9	2.2
1489.7	2.7
1496.1	8.8
1499.2	12.4
1509.7	5.0
1516.3	1.5
2432.2	75.1
3052.8	14.2
3054.8	27.4
3058.4	34.2
3080.6	12.9
3113.6	19.6
3116.5	13.7
3122.0	20.8
3128.5	25.3
3142.5	11.3
3150.6	12.4
3194.0	5.1
3867.3	32.8

Table 137: M06-2X/aug-cc-pVTZ Frequencies and IR Intensities of cis-2,3-phosphirane-1,2-dimethylcyclopentan-1-ol (H-bonded)

Frequency	IR Intensity
119.3	0.1
206.6	1.9
211.9	1.7
236.2	12.5
253.7	77.6
278.2	1.2
308.8	0.8
324.0	5.8
355.0	0.0
383.2	0.9
428.9	3.5
481.7	10.3
559.9	1.7
595.7	0.2
607.7	0.9
651.1	1.5
715.5	1.7
765.8	1.5
837.1	4.2
893.3	8.1
921.4	9.4
945.2	8.4
957.5	1.2
977.6	0.2
989.2	7.2
1013.7	8.3
1052.4	3.9
1085.7	19.3
1104.3	27.0
1140.4	22.5
1175.6	32.1
1203.4	8.5
1233.9	49.7
1236.8	8.4
1261.4	4.3
1322.8	18.3
1329.9	13.6
1366.1	2.2
1393.9	24.1
1403.0	9.6
1412.0	4.2
1487.9	2.2
1489.7	2.7
1496.1	8.8
1499.2	12.4
1509.7	5.0
1516.3	1.5
2432.2	75.1
3052.8	14.2
3054.8	27.4
3058.4	34.2
3080.6	12.9
3113.6	19.6
3116.5	13.7
3122.0	20.8
3128.5	25.3
3142.5	11.3
3150.6	12.4
3194.0	5.1
3867.3	32.8

Table 138: M06-2X/aug-cc-pVTZ Frequencies and IR Intensities of trans-2,3-phosphirane-1,2-dimethylcyclopentan-1-ol

Frequency	IR Intensity
119.0	0.7
194.7	0.2
217.5	3.5
236.9	21.5
243.6	9.7
254.2	61.8
293.7	2.2
319.2	1.0
330.4	4.1
390	6.3
410.5	2.5
457.5	2.1
561.5	2.5
608.3	1.5
611.0	3.2
653.2	12.4
723.8	2.2
783.5	3.5
825.6	6.7
893.2	7.8
910.6	5.0
945.2	0.8
954.2	23.7
979.1	12.9
988.4	2.3
1018.7	6.0
1055.1	3.6
1081.2	64.4
1095.6	0.5
1138.9	2.3
1177.4	29.6
1206.6	5.0
1225.5	0.2
1237.8	15.8
1257.3	31.8
1322.2	4.5
1341.5	3.5
1365.6	7.3
1393.5	11.0
1408.5	17.5
1412.4	11.9
1484.4	1.4
1486.4	8.6
1493.5	2.0
1498.8	3.1
1500.0	7.4
1504.0	8.5
2437.7	69.3
3054.4	18.3
3062.3	26.2
3074.7	26.9
3075.6	14.3
3116.9	15.7
3123.1	19.8
3127.6	21.2
3128.4	14.1
3141.5	8.2
3147.4	13.2
3197.4	4.1
3866.5	31.9

Table 139: M06-2X/aug-cc-pVTZ Frequencies and IR Intensities of cis-2,3-phosphirane-1-ethyl-2-methylcyclopentan-1-ol (not H-bonded)

Frequency	IR Intensity
58.0	0.1
119.8	0.0
178.1	0.6
213.0	52.3
217.4	2.1
226.8	23.2
273.8	15.0
289.9	5.7
316.1	2.2
340.9	4.9
351.0	1.9
372.8	0.4
428.6	4.3
507.1	7.9
559.6	1.7
594.3	0.7
634.7	1.7
662.6	1.4
727.9	1.7
744.1	0.8
793.8	1.8
840.8	4.5
903.1	8.3
924.3	11.1
943.8	5.2
960.7	4.8
984.4	6.4
1011.6	16.2
1020.3	2.2
1044.2	17.5
1060.4	8.2
1085.8	7.9
1108.6	15.3
1140.2	18.9
1174.4	15.6
1191.4	9.3
1230.5	43.9
1233.6	9.2
1265.6	3.5
1308.2	16.4
1314.2	15.4
1331.6	0.7
1359.1	9.3
1373.2	0.5
1397.7	19.4
1411.2	4.1
1420.6	5.1
1478.4	2.8
1496.0	10.0
1497.1	6.0
1502.9	6.8
1506.7	4.3
1509.1	3.6
1519.1	12.9
2436.3	76.1
3054.5	23.7
3056.7	19.0
3061.1	17.1
3064.8	32.3
3087.6	9.2
3109.6	11.1
3114.7	16.1
3119.2	14.1
3127.4	15.7
3135.6	27.4
3141.9	14.7
3147.4	28.3
3192.7	5.3
3874.8	31.2

Table 140: M06-2X/aug-cc-pVTZ Frequencies and IR Intensities of cis-2,3-phosphirane-1-ethyl-2-methylcyclopentan-1-ol (H-bonded)

Frequency	IR Intensity
130.0	0.9
155.4	0.2
217.4	0.1
226.4	0.1
233.1	0.4
257.1	0.3
290.0	0.1
314.6	0.8
328.6	3.4
357.2	7.9
370.9	67.2
415.0	4.5
446.2	11.1
475.9	5.6
563.7	2.9
599.0	2.4
601.8	2.2
651.3	2.4
712.2	1.9
747.7	2.3
797.5	2.8
834.8	0.4
890.6	8.6
928.4	4.3
938.4	8.7
961.4	3.3
982.7	10.1
1009.1	7.6
1037.4	17.4
1056.5	6.7
1074.8	29.2
1087.2	10.5
1110.9	14.5
1142.2	20.7
1172.0	7.4
1194.5	24.6
1222.4	23.2
1241.0	6.8
1250.9	5.9
1308.4	3.1
1324.6	1.3
1329.4	2.2
1370.7	41.3
1386.5	12.9
1393.8	23.3
1413.4	11.7
1418.1	2.9
1485.1	0.2
1491.7	9.9
1494.7	14.0
1502.3	2.9
1506.8	15.5
1511.6	2.8
1524.1	3.4
2443.4	62.7
3055.8	21.3
3063.3	12.1
3073.4	15.8
3078.3	14.3
3080.9	31.4
3108.0	2.1
3117.1	18.5
3118.6	14.6
3128.4	22.7
3132.1	37.1
3138.6	19.1
3140.0	11.7
3189.8	3.9
3831.1	21.8

Table 141: M06-2X/aug-cc-pVTZ Frequencies and IR Intensities of trans-2,3-phosphirane-1-ethyl-2-methylcyclopentan-1-ol

Frequency	IR Intensity
130.0	0.9
155.4	0.2
217.4	0.1
226.4	0.1
233.1	0.4
257.1	0.3
290.0	0.1
314.6	0.8
328.6	3.4
357.2	7.9
370.9	67.2
415.0	4.5
446.2	11.1
475.9	5.6
563.7	2.9
599.0	2.4
601.8	2.2
651.3	2.4
712.2	1.9
747.7	2.3
797.5	2.8
834.8	0.4
890.6	8.6
928.4	4.3
938.4	8.7
961.4	3.3
982.7	10.1
1009.1	7.6
1037.4	17.4
1056.5	6.7
1074.8	29.2
1087.2	10.5
1110.9	14.5
1142.2	20.7
1172.0	7.4
1194.5	24.6
1222.4	23.2
1241.0	6.8
1250.9	5.9
1308.4	3.1
1324.6	1.3
1329.4	2.2
1370.7	41.3
1386.5	12.9
1393.8	23.3
1413.4	11.7
1418.1	2.9
1485.1	0.2
1491.7	9.9
1494.7	14.0
1502.3	2.9
1506.8	15.5
1511.6	2.8
1524.1	3.4
2443.4	62.7
3055.8	21.3
3063.3	12.1
3073.4	15.8
3078.3	14.3
3080.9	31.4
3108.0	2.1
3117.1	18.5
3118.6	14.6
3128.4	22.7
3132.1	37.1
3138.6	19.1
3140.0	11.7
3189.8	3.9
3831.1	21.8

Table 142: M06-2X/aug-cc-pVTZ Frequencies and IR Intensities of cis-2-ethyl-2,3-phosphirane-1-methylcyclopentan-1-ol (not H-bonded)

Frequency	IR Intensity
82.4	0.1
112.6	0.1
186.9	1.0
208.5	1.5
232.1	2.3
246.5	83.6
287.2	9.4
296.0	0.9
316.4	0.5
354.7	0.8
364.0	2.7
386.7	0.4
446.2	2.9
492.1	8.4
558.5	1.8
599.4	0.4
621.3	1.0
691.0	1.6
726.3	1.4
752.4	4.3
803.4	2.8
840.7	5.1
906.4	13.0
920.1	15.5
951.0	0.5
958.7	0.8
966.5	5.3
993.7	0.9
1014.9	7.6
1023.4	1.6
1065.2	1.7
1083.7	8.5
1108.8	26.6
1140.9	25.8
1173.9	30.5
1203.3	2.2
1209.3	24.9
1233.9	39.3
1256.5	6.0
1314.1	1.6
1318.4	15.8
1328.3	18.0
1358.7	1.5
1372.9	6.5
1391.8	19.4
1406.2	11.2
1410.6	8.1
1486.0	1.2
1492.5	1.9
1497.6	4.8
1506.1	14.6
1507.8	3.4
1511.9	1.3
1514.7	9.8
2426.5	74.2
3053.5	14.1
3057.4	33.1
3069.2	23.3
3072.7	14.0
3080.0	13.4
3111.2	5.8
3112.3	19.5
3122.2	26.7
3127.9	24.8
3130.0	25.6
3152.1	11.9
3155.3	19.7
3199.3	4.3
3867.9	32.5

Table 143: M06-2X/aug-cc-pVTZ Frequencies and IR Intensities of cis-2-ethyl-2,3-phosphirane-1-methylcyclopentan-1-ol (H-bonded)

Frequency	IR Intensity
95.7	0.6
116.9	0.9
194.5	0.1
208.7	0.6
229.5	0.2
280.0	0.5
291.8	0.5
313.2	0.2
344.0	5.9
359.8	1.5
376.9	51.8
390.7	27.5
443.7	3.5
487.4	10.9
559.3	3.2
597.7	0.7
619.9	5.2
682.7	2.3
716.0	2.1
749.0	2.3
801.5	3.2
835.3	0.9
903.0	11.8
925.1	3.8
953.5	0.1
960.5	0.9
971.9	4.7
994.6	2.8
1017.0	35.8
1024.3	9.5
1064.6	2.5
1086.3	3.7
1104.8	11.6
1145.9	22.2
1167.3	7.8
1203.5	1.3
1227.0	39.0
1238.9	43.7
1246.5	10.9
1315.7	3.7
1324.4	13.2
1341.4	1.6
1360.5	2.8
1379.3	20.7
1382.3	37.0
1410.0	8.5
1417.2	29.4
1484.0	2.5
1489.2	4.4
1495.0	5.7
1504.6	14.5
1507.7	4.2
1509.9	0.9
1515.8	4.5
2447.7	62.8
3060.3	23.4
3068.4	10.3
3070.6	16.3
3071.3	24.7
3078.7	9.0
3109.4	5.7
3117.2	22.2
3130.7	23.5
3133.9	19.4
3144.5	24.0
3153.8	14.5
3159.4	13.3
3195.2	3.7
3831.2	21.6

Table 144: M06-2X/aug-cc-pVTZ Frequencies and IR Intensities of trans-2-ethyl-2,3-phosphirane-1-methylcyclopentan-1-ol

Frequency	IR Intensity
76.4	0.2
109.7	0.5
203.1	0.4
212.9	0.4
219.4	1.4
246.9	3.6
274.2	77.9
283.2	4.9
292.8	5.9
313.8	1.2
362.8	5.7
401.9	4.8
416.3	3.7
475.5	4.0
557.9	2.5
609.7	3.6
613.5	0.9
685.4	6.6
718.4	4.8
753.1	2.3
816.6	16.5
826.2	2.6
904.4	6.3
909.0	14.5
950.2	2.8
964.0	5.6
964.9	14.5
999.7	7.1
1011.5	4.4
1034.4	2.6
1063.5	1.1
1090.5	26.3
1099.4	23.4
1138.5	3.6
1174.4	23.8
1205.0	5.6
1206.0	3.2
1232.1	11.8
1253.7	34.1
1317.1	12.4
1318.1	3.5
1340.0	2.6
1359.5	0.9
1369.1	6.1
1390.4	9.1
1407.7	2.5
1412.6	31.8
1482.0	3.6
1484.2	3.9
1494.0	2.0
1497.1	4.7
1498.2	5.3
1501.8	8.5
1511.8	3.6
2439.5	67.2
3052.7	17.7
3068.1	31.0
3070.5	27.2
3072.3	17.5
3075.4	14.4
3106.7	11.1
3116.8	16.1
3121.6	17.7
3126.3	23.8
3127.3	30.7
3148.5	13.1
3171.0	8.1
3202.0	3.4
3862.4	29.7

Table 145: M06-2X/aug-cc-pVTZ Frequencies and IR Intensities of cis-2,3-thiirane-1,2-dimethylcyclopentan-1-ol (not H-bonded)

Frequency	IR Intensity
123.1	0.3
205.3	53.8
214.0	1.3
218.1	38.1
242.3	5.4
278.3	0.8
328.4	2.1
333.7	0.8
358.6	0.3
383.5	0.9
434.6	6.0
497.5	12.0
573.1	3.4
618.9	1.1
629.3	2.6
678.3	15.0
774.0	1.0
835.5	6.2
914.2	2.6
943.0	4.7
950.3	2.2
984.5	0.9
990.5	8.4
1017.0	7.2
1058.9	1.8
1078.4	18.9
1102.2	35.9
1143.4	18.9
1187.2	34.7
1206.6	10.1
1236.7	1.6
1238.9	58.1
1267.8	1.8
1326.7	5.1
1333.1	19.5
1376.6	1.0
1397.6	30.6
1405.1	2.0
1414.6	6.1
1483.5	1.8
1489.2	11.7
1491.3	6.5
1497.9	10.9
1502.5	2.3
1514.5	0.9
3055.4	9.5
3060.5	25.2
3063.0	22.9
3093.2	22.7
3121.0	19.3
3123.1	18.0
3129.0	11.1
3150.1	9.3
3152.4	11.0
3155.4	8.4
3186.4	7.2
3869.9	36.6

Table 146: M06-2X/aug-cc-pVTZ Frequencies and IR Intensities of cis-2,3-thiirane-1,2-dimethylcyclopentan-1-ol (H-bonded)

Frequency	IR Intensity
122.5	2.6
216.7	0.5
223.1	0.3
232.7	0.1
273.3	0.8
323.6	0.7
326.7	0.6
352.5	1.1
378.9	2.6
416.5	61.2
436.9	29.0
502.0	18.6
572.2	6.9
612.1	0.5
630.1	5.7
673.4	11.4
767.4	1.2
831.8	3.1
913.9	0.2
946.7	2.8
958.8	1.6
985.6	8.9
992.1	4.0
1020.0	32.3
1057.9	9.3
1081.1	13.6
1103.2	6.4
1146.3	31.0
1168.5	6.6
1218.1	7.3
1241.6	44.8
1252.0	34.3
1271.9	1.0
1325.8	1.3
1346.1	7.6
1379.1	52.6
1397.6	15.0
1415.9	9.5
1419.9	16.5
1482.3	4.2
1485.0	3.7
1490.7	12.7
1496.2	8.3
1501.0	5.3
1515.7	0.4
3061.2	19.8
3062.8	12.5
3067.9	17.2
3100.7	11.5
3123.9	16.6
3129.1	9.0
3142.2	12.9
3146.7	14.2
3154.0	10.8
3156.6	11.0
3186.5	5.7
3825.4	25.2

Table 147: M06-2X/aug-cc-pVTZ Frequencies and IR Intensities of trans-2,3-thiirane-1,2-dimethylcyclopentan-1-ol

Frequency	IR Intensity
118.7	1.1
204.8	0.7
222.0	2.0
239.5	2.7
251.7	1.6
272.2	88.3
299.5	6.6
330.5	1.3
340.7	2.9
392.0	5.3
416.2	2.9
468.5	2.2
581.4	8.7
619.6	0.9
632.1	3.9
679.0	28.2
787.5	7.3
831.1	1.0
907.4	5.1
930.3	6.3
954.7	21.9
981.8	5.0
995.8	0.5
1017.9	6.6
1060.3	10.8
1078.4	52.2
1096.3	10.0
1142.2	1.5
1188.3	33.7
1209.5	9.5
1229.8	1.9
1237.0	12.9
1263.8	20.8
1327.5	4.2
1345.1	5.7
1377.8	10.0
1400.2	9.0
1409.5	24.3
1415.2	3.3
1477.3	4.2
1486.2	7.8
1491.3	4.3
1494.6	6.3
1500.0	7.0
1502.4	2.9
3055.4	17.0
3070.0	17.7
3074.2	20.7
3092.5	24.0
3124.4	14.1
3127.9	13.5
3138.3	10.2
3145.4	6.6
3150.1	15.3
3155.9	6.6
3193.0	5.6
3868.2	36.3

Table 148: M06-2X/aug-cc-pVTZ Frequencies and IR Intensities of cis-2,3-thiirane-1-ethyl-2-methylcyclopentan-1-ol ((not H-bonded)

Frequency	IR Intensity
67.5	0.3
122.9	0.3
180.0	1.1
208.5	66.7
221.1	1.8
227.6	11.2
278.2	23.7
288.2	1.1
328.9	1.9
349.5	1.4
352.2	1.4
369.6	0.7
430.8	7.7
520.6	7.5
571.2	3.3
622.5	3.5
651.0	1.2
679.5	17.1
752.0	1.6
796.3	2.3
836.3	5.9
913.9	2.8
940.0	4.4
960.7	6.1
990.3	8.2
1014.7	11.0
1021.3	5.9
1041.6	16.7
1068.5	8.0
1077.9	10.5
1107.0	16.5
1143.0	16.0
1183.1	14.2
1197.6	16.7
1231.4	20.5
1233.8	35.5
1271.0	1.7
1311.7	4.5
1324.1	15.6
1330.6	0.2
1363.0	7.7
1383.4	0.2
1399.6	20.6
1412.3	2.2
1422.4	8.4
1478.1	2.8
1486.0	11.1
1496.7	8.3
1499.6	3.7
1502.0	5.2
1506.0	4.4
1516.5	9.9
3055.9	6.4
3059.9	25.8
3063.4	20.1
3066.8	24.7
3107.1	15.8
3112.8	8.0
3123.6	13.2
3129.8	28.5
3131.6	0.4
3142.3	28.8
3154.1	7.4
3157.0	15.6
3186.7	7.3
3876.0	35.1

Table 149: M06-2X/aug-cc-pVTZ Frequencies and IR Intensities of cis-2,3-thiirane-1-ethyl-2-methylcyclopentan-1-ol ((H-bonded)

Frequency	IR Intensity
131.4	1.7
159.7	0.3
213.5	0.3
232.9	0.1
241.4	1.2
260.4	0.2
282.3	0.1
323.8	1.5
342.8	0.5
357.4	0.5
414.9	25.1
415.6	51.0
456.8	21.2
496.2	11.6
576.2	8.3
613.5	0.4
635.7	3.2
674.5	14.2
757.5	0.4
797.0	4.0
835.8	2.1
914.7	0.7
935.4	17.0
962.8	5.0
988.4	1.5
1008.4	8.6
1036.5	9.6
1063.5	10.5
1073.3	33.2
1084.1	8.9
1113.6	12.7
1147.8	17.4
1172.0	9.2
1201.1	31.9
1229.0	18.0
1241.9	5.2
1257.8	6.1
1313.0	2.2
1327.1	0.4
1333.2	1.1
1372.7	44.8
1394.7	28.3
1400.2	11.3
1416.8	3.5
1420.0	6.0
1482.6	0.8
1487.6	14.6
1493.7	9.4
1499.4	4.9
1506.2	8.2
1507.7	6.3
1521.6	4.2
3062.1	5.6
3064.7	18.6
3074.8	16.0
3078.9	28.4
3097.2	14.0
3108.8	1.9
3123.5	14.4
3128.0	26.6
3133.2	14.2
3139.3	30.3
3143.3	12.1
3152.5	6.7
3186.2	5.4
3822.8	25.9

Table 150: M06-2X/aug-cc-pVTZ Frequencies and IR Intensities of trans-2,3-thiirane-1-ethyl-2-methylcyclopentan-1-ol

Frequency	IR Intensity
92.5	0.0
114.5	0.9
173.8	1.1
210.9	0.3
235.8	1.9
243.5	13.2
282.2	52.5
294.5	16.6
302.6	24.8
330.5	0.6
348.6	2.7
415.8	5.0
422.5	1.1
484.9	1.9
575.2	6.6
619.9	4.2
653.3	2.8
678.0	25.8
778.1	8.7
795.9	1.1
842.4	0.7
914.2	2.5
950.9	16.5
973.4	4.7
983.5	21.1
1000.4	30.7
1018.5	7.2
1035.5	2.2
1064.8	6.4
1081.5	25.5
1103.2	2.1
1146.2	1.3
1189.1	29.4
1202.2	6.1
1227.5	0.8
1236.3	18.7
1257.5	14.4
1317.0	6.3
1322.7	6.6
1342.8	3.1
1353.2	6.0
1387.1	8.6
1401.6	15.0
1413.6	4.3
1422.9	2.9
1478.3	3.7
1483.5	6.2
1488.1	3.0
1492.4	6.7
1502.6	7.6
1505.1	7.7
1514.7	5.9
3057.8	14.7
3066.2	13.1
3070.6	22.9
3074.5	21.8
3090.9	20.1
3104.6	1.4
3126.8	27.1
3127.3	11.4
3137.4	17.4
3139.4	15.6
3141.9	14.0
3155.3	6.0
3193.9	5.8
3873.4	33.0

Table 151: M06-2X/aug-cc-pVTZ Frequencies and IR Intensities of cis-2-ethyl-2,3-thiirane-1-methylcyclopentan-1-ol (not H-bonded)

Frequency	IR Intensity
83.1	0.3
112.4	0.3
186.2	1.6
208.6	1.6
228.0	78.7
234.4	11.7
281.5	4.8
295.5	0.8
335.0	1.4
357.4	1.0
368.8	1.5
387.7	1.1
445.8	4.5
505.8	11.6
567.9	2.9
624.1	4.0
635.9	1.2
714.4	14.7
754.1	1.7
808.9	4.3
840.0	10.9
912.5	7.2
945.0	2.5
951.6	0.9
968.1	5.7
996.0	5.2
1016.5	2.3
1030.9	4.3
1067.9	1.7
1084.2	9.9
1109.4	32.7
1144.4	20.0
1184.6	35.9
1206.8	7.7
1211.4	8.3
1236.8	48.0
1263.1	2.7
1312.7	4.8
1323.8	3.0
1332.5	20.4
1362.1	2.4
1382.1	2.5
1396.9	21.3
1405.9	6.1
1411.8	8.9
1482.6	2.8
1488.4	1.7
1495.5	7.5
1498.8	10.8
1505.8	3.9
1509.7	3.6
1514.7	9.3
3055.3	10.9
3057.2	26.5
3071.3	19.2
3075.8	13.1
3092.5	23.8
3121.2	17.6
3122.7	12.2
3124.0	17.8
3133.6	21.1
3150.4	9.9
3152.3	11.9
3158.1	17.7
3191.1	6.1
3870.3	36.5

Table 152: M06-2X/aug-cc-pVTZ Frequencies and IR Intensities of cis-2-ethyl-2,3-thiirane-1-methylcyclopentan-1-ol (H-bonded)

Frequency	IR Intensity
89.4	0.6
110.6	1.5
182.7	0.5
206.5	0.2
235.0	0.8
275.4	0.1
291.6	0.4
330.1	0.2
351.9	2.8
368.3	0.7
384.2	3.9
422.3	65.3
446.7	23.6
506.4	17.9
568.6	6.0
616.8	3.2
639.1	2.2
707.1	13.6
754.8	1.0
808.1	3.3
835.4	7.2
916.1	2.0
946.2	3.8
958.1	1.0
972.3	4.5
996.6	11.6
1021.4	16.3
1032.0	16.6
1068.8	7.9
1086.3	7.6
1107.4	8.6
1150.8	20.7
1169.0	8.5
1210.5	2.9
1233.0	27.0
1246.7	55.4
1252.4	1.6
1322.9	10.2
1323.0	2.2
1348.7	2.7
1363.2	1.7
1382.1	53.0
1398.9	6.1
1411.8	8.6
1419.4	24.5
1482.7	3.0
1485.5	5.9
1492.6	4.3
1499.3	13.1
1505.3	4.3
1510.5	3.4
1515.5	4.3
3058.0	20.1
3068.4	6.8
3071.3	18.3
3074.8	17.3
3101.5	12.3
3120.5	2.8
3125.0	18.2
3133.9	21.4
3144.3	15.8
3147.4	14.5
3153.0	17.5
3163.1	13.0
3191.5	4.9
3823.6	25.6

Table 153: M06-2X/aug-cc-pVTZ Frequencies and IR Intensities of trans-2-ethyl-2,3-thiirane-1-methylcyclopentan-1-ol

Frequency	IR Intensity
49.7	0.1
115.5	1.2
200.7	0.7
209.8	5.6
228.3	1.0
242.8	86.1
253.7	4.9
275.4	3.0
310.9	1.2
330.3	1.6
351.8	3.5
403.2	4.3
426.9	0.9
515.5	6.7
571.7	5.3
611.1	2.0
633.4	2.5
673.1	23.9
775.8	2.6
816.1	14.5
841.9	7.6
905.0	11.2
938.8	4.9
958.7	16.2
972.7	1.9
1000.6	4.5
1013.4	2.3
1049.3	6.7
1067.8	0.3
1094.1	33.1
1118.5	5.6
1142.4	2.6
1164.0	50.9
1200.2	1.7
1214.4	5.1
1233.9	13.0
1259.3	21.5
1305.9	4.3
1326.9	4.2
1344.1	7.6
1368.0	8.9
1384.5	7.2
1400.0	1.9
1409.2	16.8
1413.4	15.5
1477.5	3.1
1490.9	5.3
1491.1	2.6
1494.5	3.8
1499.8	4.3
1502.9	9.3
1513.6	5.3
3055.3	15.6
3062.6	15.2
3069.4	23.5
3074.7	21.1
3091.7	26.6
3108.3	2.2
3124.3	13.8
3128.7	14.0
3135.5	23.5
3145.1	4.3
3146.7	12.8
3149.3	17.8
3203.1	5.1
3869.8	36.7

Table 154: M06-2X/cc-pV(T+d)Z optimized geometry of cis-2,3- phosphirane-1,2-dimethylcyclopentan-1-ol (not H-bonded) (in Å)

Atom	x	y	z
C	0.979775	−0.011587	0.249791
C	−0.344244	0.592129	−0.228742
C	−1.118624	−0.507834	−0.882176
C	−0.292458	−1.784654	−0.835357
C	0.701741	−1.525972	0.302205
C	−0.320215	1.995624	−0.776308
P	−1.811370	0.014250	0.769493
C	2.104027	0.295014	−0.737060
O	1.298672	0.533988	1.520595
H	3.014538	−0.223817	−0.429446
H	1.849495	−0.041254	−1.743275
H	2.310035	1.363402	−0.766134
H	2.182489	0.246372	1.765361
H	0.247391	−1.752892	1.267350
H	1.614652	−2.114888	0.214124
H	−0.893964	−2.677709	−0.671078
H	0.234782	−1.910201	−1.784223
H	−1.710293	−0.297082	−1.762614
H	0.302448	2.057491	−1.671506
H	−1.322167	2.325449	−1.045174
H	0.078597	2.686339	−0.032604
H	−2.687220	0.974611	0.216344

Table 155: M06-2X/cc-pV(T+d)Z optimized geometry of cis-2,3- phosphirane-1,2-dimethylcyclopentan-1-ol (H-bonded) (in Å)

Atom	x	y	z
C	0.969279	0.000150	0.241770
C	−0.343062	0.600348	−0.292568
C	−1.108438	−0.501780	−0.936837
C	−0.288338	−1.781503	−0.857733
C	0.687094	−1.515378	0.293109
C	−0.306929	1.997515	−0.853558
P	−1.836875	0.037180	0.695807
C	2.127934	0.300572	−0.698224
O	1.350736	0.535222	1.494636
H	3.022697	−0.198532	−0.328603
H	1.914570	−0.056095	−1.706621
H	2.323810	1.370369	−0.734528
H	0.589608	0.479398	2.082418
H	0.217938	−1.752792	1.249921
H	1.603982	−2.098010	0.226958
H	−0.897458	−2.670072	−0.698464
H	0.254410	−1.914937	−1.796445
H	−1.696734	−0.304872	−1.822942
H	0.332603	2.042303	−1.737439
H	−1.301970	2.330783	−1.143368
H	0.086925	2.695314	−0.113670
H	−2.697761	1.005992	0.138552

Table 156: M06-2X/cc-pV(T+d)Z optimized geometry of trans-2,3- phosphirane-1,2-dimethylcyclopentan-1-ol (in Å)

Atom	x	y	z
C	−0.302990	0.610814	−0.270024
C	1.028066	−0.009118	0.168894
C	0.700641	−1.498127	0.357235
C	−0.346615	−1.796074	−0.722295
C	−1.139298	−0.502731	−0.811949
C	1.658029	0.642343	1.385942
O	1.883820	0.143976	−0.968272
P	−1.741764	0.152677	0.828464
C	−0.250853	1.967132	−0.923677
H	−1.767481	−0.328359	−1.674559
H	0.146898	−1.971635	−1.679477
H	−0.960641	−2.663943	−0.486766
H	1.598680	−2.109434	0.267031
H	0.286089	−1.665387	1.352992
H	−1.232716	2.246220	−1.303869
H	0.064339	2.735597	−0.216531
H	0.456768	1.953149	−1.752104
H	2.595617	0.143142	1.641894
H	1.874819	1.690741	1.181901
H	0.992519	0.579695	2.246163
H	2.745234	−0.219119	−0.744695
H	−2.604866	1.114993	0.260464

Table 157: M06-2X/cc-pV(T+d)Z optimized geometry of cis-2,3- phosphirane-1-ethyl-2-methylcyclopentan-1-ol (not H-bonded) (in Å)

Atom	x	y	z
C	0.406096	−1.476311	0.330879
C	0.638708	0.047548	0.290858
C	−0.681073	0.600001	−0.266006
C	−1.378307	−0.527573	−0.957987
C	−0.509277	−1.771234	−0.862229
C	1.802652	0.422561	−0.639884
C	3.169609	−0.069087	−0.175407
O	0.853189	0.603430	1.579271
C	−0.695626	2.003213	−0.817623
P	−2.177546	−0.034174	0.653371
H	3.219904	−1.154529	−0.096958
H	3.941708	0.241834	−0.877933
H	3.436461	0.357083	0.793026
H	1.588897	0.038711	−1.641230
H	1.835251	1.509281	−0.714772
H	1.649951	0.217614	1.952476
H	−0.101680	−1.712428	1.266949
H	1.331198	−2.048991	0.305374
H	−1.083851	−2.687300	−0.730972
H	0.077092	−1.876188	−1.778410
H	−1.927275	−0.338535	−1.870500
H	−0.053226	2.089927	−1.696212
H	−1.702517	2.290121	−1.115415
H	−0.347882	2.712290	−0.065550
H	−3.059686	0.892142	0.054489

Table 158: M06-2X/cc-pV(T+d)Z optimized geometry of cis-2,3- phosphirane-1-ethyl-2-methylcyclopentan-1-ol (H-bonded) (in Å)

Atom	x	y	z
C	−0.365120	1.473067	0.739010
C	−0.568626	−0.058120	0.673671
C	0.482370	−0.527642	−0.348313
C	0.963024	0.661785	−1.101848
C	0.215769	1.896018	−0.617819
O	−0.406049	−0.681235	1.936312
C	−1.990712	−0.439202	0.256430
C	−2.487338	0.186013	−1.040727
C	0.306332	−1.882234	−0.983744
P	2.215677	0.029768	0.126197
H	−1.290048	1.991496	0.989061
H	0.347371	1.686474	1.537546
H	−0.577023	2.133002	−1.327713
H	0.859463	2.771132	−0.537970
H	1.214183	0.574313	−2.150606
H	1.136541	−2.113606	−1.649061
H	0.255202	−2.657110	−0.217979
H	−0.614666	−1.924616	−1.568792
H	0.523835	−0.617243	2.179294
H	−1.802130	0.005265	−1.870885
H	−3.456430	−0.229531	−1.313415
H	−2.611959	1.264156	−0.941382
H	−2.040605	−1.527233	0.200865
H	−2.640999	−0.144962	1.082227
H	2.856304	−0.852755	−0.768727

Table 159: M06-2X/cc-pV(T+d)Z optimized geometry of trans-2,3- phosphirane-1-ethyl-2-methylcyclopentan-1-ol (in Å)

Atom	x	y	z
P	1.645617	0.077802	−1.760429
C	0.886522	0.630762	−0.146117
C	−0.472890	−0.013671	0.149818
C	−0.222878	−1.517543	−0.053412
C	1.224657	−1.729261	0.407529
C	1.918560	−0.437751	0.011683
C	−1.616070	0.552739	−0.685099
C	−2.971428	−0.070902	−0.371385
O	−0.690867	0.243356	1.540668
C	1.109225	2.034919	0.353339
H	−0.937307	−2.120627	0.506699
H	−0.334081	−1.773548	−1.109158
H	1.256714	−1.825343	1.494028
H	1.679513	−2.617042	−0.029285
H	2.874230	−0.195769	0.455913
H	2.147329	2.331050	0.207269
H	0.480101	2.750611	−0.177244
H	0.873009	2.092550	1.415536
H	−2.963247	−1.148595	−0.539702
H	−3.268681	0.112357	0.662812
H	−3.749304	0.354924	−1.003363
H	−1.659116	1.630174	−0.512600
H	−1.374880	0.403644	−1.739099
H	−1.479150	−0.233102	1.814110
H	2.639434	1.080812	−1.747539

Table 160: M06-2X/cc-pV(T+d)Z optimized geometry of cis-2-ethyl-2,3-phosphirane-methylcyclopentan-1-ol (not H-bonded) (in Å)

Atom	x	y	z
C	−0.318030	1.018488	−0.312413
C	0.328388	−0.368976	−0.199994
C	−0.291355	−1.046060	0.983390
C	−1.277522	−0.088153	1.634898
C	−1.616013	0.895312	0.509219
C	1.798304	−0.508368	−0.563202
C	2.766529	−0.305360	0.602605
P	−0.870876	−1.733135	−0.647708
C	0.570152	2.114314	0.272367
O	−0.555663	1.290926	−1.685435
H	1.518573	2.177121	−0.258641
H	0.060793	3.076466	0.179472
H	0.766195	1.943662	1.331042
H	−0.845861	2.203049	−1.772006
H	−2.391133	0.484345	−0.138436
H	−1.962345	1.861584	0.875841
H	−2.159358	−0.588911	2.032527
H	−0.787439	0.429582	2.463291
H	0.305520	−1.689513	1.614543
H	1.980802	−1.495213	−0.985767
H	2.009414	0.201444	−1.366115
H	2.674263	0.680770	1.052901
H	2.589963	−1.044436	1.384516
H	3.796356	−0.422641	0.264866
H	0.131450	−2.727211	−0.701648

Table 161: M06-2X/cc-pV(T+d)Z optimized geometry of cis-2-ethyl-2,3-phosphirane-methylcyclopentan-1-ol (H-bonded) (in Å)

Atom	x	y	z
C	−0.366063	0.999488	−0.268261
C	0.301749	−0.385210	−0.144589
C	−0.292334	−1.059971	1.043612
C	−1.271009	−0.105054	1.712030
C	−1.639666	0.874327	0.593641
C	1.762591	−0.523711	−0.538640
C	2.749451	−0.281200	0.603687
P	−0.909272	−1.763661	−0.568915
C	0.519530	2.117077	0.264463
O	−0.635866	1.353092	−1.611771
H	1.456857	2.170097	−0.286492
H	−0.005985	3.062399	0.133796
H	0.733305	1.976984	1.323886
H	−1.080673	0.607134	−2.028563
H	−2.443273	0.464089	−0.020648
H	−1.972730	1.843615	0.960019
H	−2.138530	−0.610456	2.133968
H	−0.760591	0.415885	2.525724
H	0.311544	−1.710833	1.660817
H	1.946153	−1.519557	−0.939634
H	1.950749	0.170392	−1.361023
H	2.651012	0.714631	1.029961
H	2.596657	−1.003516	1.406018
H	3.773934	−0.393820	0.249004
H	0.092488	−2.751708	−0.666113

Table 162: M06-2X/cc-pV(T+d)Z optimized geometry of trans-2-ethyl-2,3-phosphirane-methylcyclopentan-1-ol (in Å)

Atom	x	y	z
C	0.558946	0.793338	0.086529
C	1.843881	0.084814	−0.379904
C	1.387241	−1.286612	−0.894236
C	0.143629	−1.609023	−0.082198
C	−0.392582	−0.336691	0.497570
C	−1.887540	−0.045226	0.516528
H	−2.031048	0.974230	0.881222
C	−2.584124	−0.214086	−0.832642
H	−2.495630	−1.240889	−1.189312
H	−3.646991	0.009478	−0.737950
H	−2.153326	0.445141	−1.580553
H	−2.370018	−0.698831	1.245341
H	−0.546373	−2.340389	−0.483064
H	1.095395	−1.209602	−1.943455
H	2.165388	−2.044823	−0.817370
H	2.332862	0.675291	−1.154694
H	2.550970	−0.013247	0.441594
C	0.798363	1.834955	1.166133
H	1.249535	1.385032	2.049426
H	1.472407	2.613001	0.799025
H	−0.142135	2.305187	1.452592
O	−0.072564	1.414169	−1.039453
H	0.458105	2.173048	−1.297532
P	0.202275	−1.557788	1.773182
H	1.535787	−1.112509	1.892110

Table 163: M06-2X/cc-pV(T+d)Z optimized geometry of cis-2,3-thiirane- 1,2-dimethylcyclopentan-1-ol (not H-bonded) (in Å)

Atom	x	y	z
C	−0.266722	0.792094	0.052481
C	−1.576906	0.194750	−0.303485
C	−1.415914	−1.308106	−0.448125
C	−0.117901	−1.613728	0.310113
C	0.748285	−0.350621	0.143420
C	1.569606	−0.424741	−1.142774
H	2.227068	−1.295623	−1.106691
H	0.926747	−0.526808	−2.018802
H	2.184483	0.465740	−1.258990
O	1.602693	−0.106890	1.246033
H	2.292285	−0.776133	1.255505
H	−0.318249	−1.749592	1.370108
H	0.386495	−2.503819	−0.065445
H	−2.270700	−1.862759	−0.065053
H	−1.312906	−1.547161	−1.509781
S	−1.488428	0.816874	1.394809
H	−2.254385	0.727536	−0.957884
C	0.191206	2.127552	−0.469192
H	0.521936	2.048595	−1.506100
H	−0.620811	2.849379	−0.417937
H	1.022586	2.496834	0.131381

Table 164: M06-2X/cc-pV(T+d)Z optimized geometry of cis-2,3-thiirane- 1,2-dimethylcyclopentan-1-ol (H-bonded) (in Å)

Atom	x	y	z
C	−0.010320	−0.026518	−0.019412
C	1.532588	−0.040631	0.038134
C	1.957654	1.431071	0.046348
C	0.860075	2.135989	−0.727500
C	−0.350163	1.289575	−0.741527
C	−1.744167	1.843587	−0.637607
H	−2.468885	1.096113	−0.961192
H	−1.972304	2.118053	0.393469
H	−1.850099	2.725682	−1.265289
S	0.474887	1.439686	−2.363863
H	0.768675	3.212839	−0.671707
H	1.965150	1.828876	1.064448
H	2.946349	1.592546	−0.379397
H	1.891709	−0.590202	0.906115
H	1.913176	−0.537822	−0.852033
O	−0.548701	−1.154328	−0.675549
H	−0.225976	−1.137896	−1.583763
C	−0.608543	−0.032603	1.379574
H	−0.301565	−0.943438	1.891245
H	−0.263385	0.828127	1.954458
H	−1.695672	−0.015663	1.332264

Table 165: M06-2X/cc-pV(T+d)Z optimized geometry of trans-2,3-thiirane- 1,2-dimethylcyclopentan-1-ol (in Å)

Atom	x	y	z
C	0.721481	−0.397567	0.059524
C	−0.219572	−1.593154	0.276474
C	−1.539854	−1.174283	−0.382035
C	−1.578596	0.328534	−0.191679
C	−0.206428	0.823622	0.067039
S	−1.309381	0.917581	1.506273
H	−2.266058	0.927721	−0.773513
H	−1.510144	−1.376712	−1.454421
H	−2.406168	−1.680806	0.039212
H	0.203703	−2.495979	−0.163922
H	−0.360083	−1.769642	1.340655
C	0.314090	2.117038	−0.496804
H	−0.476337	2.865028	−0.506494
H	1.135289	2.500420	0.109292
H	0.679526	1.957170	−1.510113
C	1.861024	−0.322387	1.056648
H	2.462653	−1.233107	1.013108
H	2.507365	0.525189	0.829642
H	1.474812	−0.216706	2.069671
O	1.228187	−0.424163	−1.278297
H	1.825211	−1.173139	−1.357141

Table 166: M06-2X/cc-pV(T+d)Z optimized geometry of cis-2,3-thiirane- 1-ethyl-2-methylcyclopentan-1-ol (not H-bonded) (in Å)

Atom	x	y	z
C	−0.943909	0.593286	−0.035503
C	−1.743757	−0.550953	−0.536162
C	−0.896748	−1.808427	−0.481325
C	0.213880	−1.460475	0.517526
C	0.438815	0.056553	0.357782
C	1.446753	0.368787	−0.760544
C	2.871156	−0.087389	−0.464006
H	2.943890	−1.164616	−0.318427
H	3.532166	0.174796	−1.288912
H	3.263446	0.406831	0.425777
H	1.093882	−0.084981	−1.691205
H	1.456973	1.447763	−0.913152
O	0.838004	0.692447	1.558982
H	1.636936	0.268631	1.882494
H	−0.122142	−1.645463	1.535182
H	1.119751	−2.038605	0.346041
H	−1.465460	−2.689611	−0.189356
H	−0.482160	−1.994122	−1.475613
S	−2.286600	0.076293	1.073067
H	−2.441480	−0.408167	−1.351034
C	−1.059486	1.976398	−0.620391
H	−0.556373	2.037828	−1.586378
H	−2.106877	2.234390	−0.759704
H	−0.609129	2.706701	0.052094

Table 167: M06-2X/cc-pV(T+d)Z optimized geometry of cis-2,3-thiirane- 1-ethyl-2-methylcyclopentan-1-ol (H-bonded) (in Å)

Atom	x	y	z
C	0.404849	0.099056	0.698807
C	−0.697590	0.623333	−0.239583
C	−1.382185	−0.537520	−0.843332
C	−0.719230	−1.820304	−0.381311
C	0.086529	−1.406644	0.857905
H	0.995944	−1.992957	0.982921
H	−0.511737	−1.537074	1.757437
H	−0.065757	−2.175936	−1.179769
H	−1.441639	−2.606802	−0.169772
S	−2.375893	0.314813	0.419720
H	−1.785237	−0.470606	−1.845683
C	−0.499467	1.934257	−0.949723
H	−1.391974	2.198916	−1.512688
H	−0.300107	2.723005	−0.223946
H	0.346336	1.882214	−1.637045
O	0.411793	0.786051	1.933889
H	−0.478918	0.727459	2.298017
C	1.797618	0.341527	0.115801
C	2.085539	−0.372995	−1.198763
H	1.336618	−0.148845	−1.960694
H	3.055270	−0.069573	−1.590342
H	2.111161	−1.454643	−1.067184
H	1.932324	1.418259	0.005431
H	2.509554	0.019057	0.877419

Table 168: M06-2X/cc-pV(T+d)Z optimized geometry of trans-2,3-thiirane- 1-ethyl-2-methylcyclopentan-1-ol (in Å)

Atom	x	y	z
C	−0.466326	−0.007663	0.136508
C	0.891221	0.651069	−0.140731
C	1.919980	−0.410766	−0.051723
C	1.251230	−1.724615	0.294816
C	−0.216163	−1.507758	−0.096367
H	−0.898625	−2.114177	0.498912
H	−0.375529	−1.753958	−1.144598
H	1.338072	−1.871987	1.373119
H	1.702555	−2.579687	−0.204968
S	1.632257	0.203120	−1.737294
H	2.896764	−0.180781	0.352316
C	1.121894	2.049010	0.364484
H	2.154536	2.343412	0.187053
H	0.474695	2.759722	−0.149596
H	0.906161	2.095384	1.430963
C	−1.606395	0.574038	−0.689188
C	−2.954633	−0.078412	−0.405304
H	−2.930841	−1.148619	−0.614060
H	−3.257949	0.061254	0.634092
H	−3.735610	0.361108	−1.023739
H	−1.665385	1.644503	−0.481661
H	−1.351760	0.458318	−1.744061
O	−0.673412	0.232079	1.531690
H	−1.458343	−0.249088	1.806096

Table 169: M06-2X/cc-pV(T+d)Z optimized geometry of cis-2-ethyl-2,3- thiirane-1-methylcyclopentan-1-ol (not H-bonded) (in Å)

Atom	x	y	z
C	−0.384873	−0.296569	0.465760
C	0.072564	−1.584686	−0.114153
C	1.365202	−1.354742	−0.877163
C	1.908147	−0.044504	−0.293506
C	0.657108	0.767029	0.096763
C	0.187804	1.615156	−1.083475
H	0.962973	2.344248	−1.329197
H	0.014277	1.004177	−1.970038
H	−0.724972	2.155884	−0.839021
O	0.855785	1.598776	1.226172
H	1.436804	2.322546	0.977117
H	2.483719	−0.237968	0.608207
H	2.537934	0.497721	−0.998615
H	2.064729	−2.183230	−0.780176
H	1.127803	−1.240890	−1.938293
S	0.224949	−1.501414	1.686159
H	−0.646002	−2.302091	−0.487151
C	−1.847308	0.100158	0.566882
C	−2.633448	−0.024014	−0.738502
H	−2.625523	−1.053240	−1.097869
H	−3.673281	0.260003	−0.578143
H	−2.235644	0.608018	−1.529508
H	−2.322029	−0.528303	1.317669
H	−1.890128	1.122554	0.948441

Table 170: M06-2X/cc-pV(T+d)Z optimized geometry of cis-2-ethyl-2,3- thiirane-1-methylcyclopentan-1-ol (H-bonded) (in Å)

Atom	x	y	z
C	−1.855796	0.006654	0.574593
C	−0.380385	−0.316943	0.443272
C	0.124071	−1.578037	−0.138974
C	1.377409	−1.292822	−0.944365
C	1.886675	0.036736	−0.380033
C	0.627554	0.797327	0.092577
C	0.104088	1.702095	−1.012556
H	−0.817040	2.196386	−0.708714
H	0.852829	2.467703	−1.211068
H	−0.074172	1.139220	−1.929085
O	0.876244	1.649817	1.190247
H	1.154048	1.093352	1.926873
H	2.534095	−0.140282	0.476320
H	2.448728	0.617823	−1.108731
H	2.112422	−2.093208	−0.880746
H	1.089760	−1.186864	−1.993840
S	0.314938	−1.490450	1.666819
H	−0.566480	−2.340269	−0.474828
H	−2.298069	−0.690771	1.283854
H	−1.944231	1.000722	1.018704
C	−2.637923	−0.070826	−0.737015
H	−2.287960	0.646169	−1.475763
H	−2.561573	−1.067031	−1.173683
H	−3.693418	0.128800	−0.554412

Table 171: M06-2X/cc-pV(T+d)Z optimized geometry of trans-2-ethyl-2,3- thiirane-1-methylcyclopentan-1-ol (in Å)

Atom	x	y	z
C	0.564779	0.814131	0.063999
C	1.865592	0.103439	−0.348497
C	1.419388	−1.264268	−0.877739
C	0.183499	−1.582779	−0.060675
C	−0.378363	−0.323433	0.484089
C	−1.871183	−0.061727	0.555926
H	−2.028278	0.938902	0.963731
C	−2.593698	−0.195088	−0.784286
H	−2.467466	−1.198403	−1.192690
H	−3.662191	−0.026387	−0.650402
H	−2.214622	0.518971	−1.509322
H	−2.303420	−0.762216	1.269282
S	0.356500	−1.419239	1.738629
H	−0.478885	−2.376395	−0.379010
H	1.118369	−1.187960	−1.924753
H	2.193199	−2.025476	−0.797602
H	2.396723	0.691776	−1.096602
H	2.516766	−0.014449	0.514819
C	0.766975	1.879607	1.124765
H	1.181942	1.443006	2.031797
H	1.461836	2.641272	0.763068
H	−0.178839	2.366332	1.361885
O	−0.059034	1.384377	−1.090063
H	0.470661	2.134265	−1.374830

Table 172: M06-2X/cc-pV(T+d)Z Frequencies and IR Intensities of cis-2,3-phosphirane-1,2-dimethylcyclopentan-1-ol (not H-bonded)

Frequency	IR Intensity
119.2	0.0
209.0	2.2
213.8	1.1
236.7	7.2
260.5	80.9
277.1	1.4
309.0	0.9
324.4	7.0
354.2	0.0
383.1	0.8
429.4	3.8
482.1	10.0
560.3	1.4
596.8	0.2
608.6	0.8
653.2	1.3
717.2	1.6
766.6	1.8
838.1	4.2
895.6	8.1
921.9	9.3
945.4	8.7
957.6	1.0
979.0	0.3
989.4	7.0
1015.0	8.2
1053.4	4.3
1087.0	19.3
1105.5	25.1
1141.4	21.8
1177.0	32.9
1204.1	7.9
1235.4	49.6
1238.1	10.2
1262.7	4.3
1323.8	18.6
1330.3	15.7
1367.5	2.2
1396.1	26.4
1403.5	9.3
1412.8	4.5
1488.3	2.4
1489.8	2.8
1496.6	9.0
1499.5	12.1
1510.1	4.7
1516.4	1.6
2430.1	75.8
3053.5	14.5
3055.3	28.4
3059.6	34.4
3081.4	12.9
3114.9	19.5
3117.7	14.4
3122.7	21.6
3129.1	26.5
3143.4	11.9
3151.9	12.7
3196.1	5.4
3870.9	30.5

Table 173: M06-2X/cc-pV(T+d)Z Frequencies and IR Intensities of cis-2,3-phosphirane-1,2-dimethylcyclopentan-1-ol (H-bonded)

Frequency	IR Intensity
126.0	1.7
209.0	0.5
217.2	0.2
236.4	0.2
266.4	1.2
308.4	0.3
316.8	0.6
347.3	11.1
365.9	55.7
386.7	17.8
428.7	5.9
479.3	12.0
560.7	3.1
596.2	0.2
609.1	5.9
643.6	1.5
712.3	1.9
760.6	1.2
833.8	0.4
886.7	8.2
925.8	1.6
949.0	1.8
963.3	4.1
979.6	0.0
990.2	6.4
1016.6	44.6
1052.1	3.7
1090.0	7.8
1101.1	9.8
1142.9	29.6
1168.2	6.4
1210.4	4.4
1238.5	68.7
1245.1	16.7
1263.5	4.8
1324.6	2.9
1340.4	7.9
1379.5	41.7
1383.3	22.4
1414.2	4.3
1418.5	32.9
1482.8	2.2
1490.1	1.7
1494.7	9.0
1498.1	14.3
1509.1	6.1
1517.3	1.0
2442.6	65.3
3055.7	24.8
3062.3	20.7
3069.8	19.1
3077.7	10.7
3118.9	22.3
3119.4	11.4
3135.1	19.1
3142.7	10.8
3144.5	20.5
3157.1	13.2
3195.1	4.6
3837.9	19.5

Table 174: M06-2X/cc-pV(T+d)Z Frequencies and IR Intensities of trans-2,3-phosphirane-1,2-dimethylcyclopentan-1-ol

Frequency	IR Intensity
119.6	0.7
196.2	0.1
218.4	3.1
236.4	21.4
243.2	4.1
256.4	67.3
293.4	2.1
319.4	1.2
330.4	4.4
390.2	6.1
410.9	2.5
457.4	2.2
562.3	2.6
609.9	0.5
611.2	4.1
654.6	12.0
726.0	2.0
785.2	3.3
826.8	6.5
895.0	7.8
911.6	4.9
945.7	0.7
955.7	22.8
981.0	12.6
988.2	2.2
1019.9	6.1
1056.3	3.8
1081.6	62.6
1096.7	0.3
1140.4	2.1
1179.2	30.4
1207.0	5.2
1227.1	0.5
1238.7	15.2
1258.6	34.0
1323.1	4.7
1342.5	4.1
1366.7	7.7
1395.0	10.7
1408.8	17.8
1412.5	14.3
1484.3	1.2
1486.1	9.2
1493.4	1.7
1498.9	4.2
1499.8	6.6
1504.7	8.1
2433.9	69.9
3054.6	18.4
3062.8	26.8
3075.9	24.8
3076.3	16.4
3118.6	15.0
3123.6	23.2
3129.2	19.2
3129.3	16.0
3143.2	8.1
3148.0	14.2
3199.4	4.4
3871.1	30.1

Table 175: M06-2X/cc-pV(T+d)Z Frequencies and IR Intensities of cis-2,3-phosphirane-1-ethyl-2-methylcyclopentan-1-ol (not H-bonded)

Frequency	IR Intensity
65.7	0.1
120.6	0.0
179.1	0.6
211.6	62.4
219.6	1.0
229.2	14.1
277.7	11.1
290.8	10.4
316.4	2.4
342.1	5.8
352.1	2.8
372.7	0.5
428.6	4.6
507.2	7.3
560.2	1.4
595.6	0.6
636.5	1.5
665.0	1.4
730.0	1.6
745.4	1.0
796.7	1.7
841.7	4.4
904.2	8.2
926.0	11.2
944.4	5.5
961.2	4.8
986.0	7.0
1013.1	14.8
1020.7	2.5
1046.3	17.2
1061.7	8.1
1087.1	8.0
1109.6	15.2
1141.0	19.2
1176.5	14.4
1191.9	9.1
1231.7	46.4
1234.7	8.3
1267.4	3.8
1309.1	12.5
1316.3	20.6
1332.8	0.8
1359.8	9.3
1374.0	0.6
1399.2	20.7
1412.0	4.2
1421.6	6.3
1479.8	2.9
1496.4	9.8
1497.6	6.8
1504.3	5.8
1507.4	5.0
1510.4	3.0
1519.5	12.6
2430.7	76.7
3055.2	24.9
3058.1	17.7
3062.7	18.6
3066.4	31.1
3087.6	10.1
3112.1	12.3
3116.2	15.7
3120.9	12.3
3130.6	15.2
3136.1	29.7
3142.7	15.5
3147.9	29.7
3195.1	5.7
3878.4	29.7

Table 176: M06-2X/cc-pV(T+d)Z Frequencies and IR Intensities of cis-2,3-phosphirane-1-ethyl-2-methylcyclopentan-1-ol (H-bonded)

Frequency	IR Intensity
129.8	0.9
154.5	0.2
220.4	0.1
227.5	0.1
234.1	0.4
257.4	0.4
290.5	0.1
315.2	0.7
329.2	3.2
358.1	6.8
372.9	69.9
415.5	4.2
446.6	11.3
476.7	6.1
564.8	2.7
599.7	2.5
603.4	1.8
652.9	2.2
713.4	1.7
748.9	2.6
798.9	2.7
836.0	0.5
892.6	8.6
929.0	4.7
938.4	8.6
961.5	3.3
984.0	10.1
1009.6	7.3
1038.4	17.2
1057.5	6.3
1075.9	28.7
1088.8	10.0
1111.5	14.8
1143.6	20.2
1173.3	8.5
1195.8	23.0
1223.2	24.7
1241.3	7.2
1251.6	6.1
1308.5	3.3
1324.9	1.0
1330.0	2.3
1371.9	41.2
1387.2	12.2
1396.6	31.4
1414.2	12.3
1419.2	2.8
1485.8	0.3
1492.2	9.4
1495.3	14.6
1503.0	2.9
1507.5	15.9
1512.3	2.5
1524.6	3.5
2442.3	63.6
3056.4	22.1
3063.5	12.3
3075.3	14.9
3078.9	14.6
3082.5	33.2
3109.4	1.9
3118.3	18.9
3119.6	14.2
3128.9	24.5
3133.2	37.6
3139.8	18.6
3141.0	13.2
3192.3	4.1
3836.4	20.1

Table 177: M06-2X/cc-pV(T+d)Z Frequencies and IR Intensities of trans-2,3-phosphirane-1-ethyl-2-methylcyclopentan-1-ol

Frequency	IR Intensity
86.3	0.0
113.7	0.5
174.0	1.2
202.0	0.1
231.4	6.2
240.4	15.9
273.5	41.0
295.3	14.6
303.3	32.4
320.1	0.3
342.0	3.5
410.7	4.5
417.9	1.9
468.3	1.3
556.6	3.0
604.5	3.8
635.7	2.5
655.2	8.6
725.2	2.3
776.3	4.1
794.4	2.2
841.2	5.4
893.4	7.0
929.3	4.8
949.9	4.4
978.2	5.2
978.7	18.8
998.7	49.1
1017.4	4.7
1037.9	2.0
1061.0	2.8
1089.0	27.8
1101.9	0.3
1143.6	1.7
1181.3	23.6
1198.6	2.9
1227.6	0.7
1238.1	20.4
1252.4	24.3
1314.2	12.1
1317.6	2.1
1343.3	3.8
1350.0	6.0
1374.0	4.0
1400.4	16.6
1411.8	6.8
1419.9	5.9
1483.5	2.2
1486.1	6.5
1488.7	3.0
1499.1	5.5
1505.5	12.2
1505.9	5.3
1515.5	5.5
2434.4	71.8
3058.6	13.9
3062.9	18.8
3067.2	28.5
3073.7	9.1
3075.3	28.4
3103.7	2.1
3118.1	19.2
3127.7	41.6
3128.0	11.2
3130.2	15.3
3137.9	23.5
3142.3	9.2
3198.6	4.7
3877.0	27.9

Table 178: M06-2X/cc-pV(T+d)Z Frequencies and IR Intensities of cis-2-ethyl-2,3-phosphirane-1-methylcyclopentan-1-ol (not H-bonded)

Frequency	IR Intensity
83.9	0.1
112.1	0.0
186.9	1.0
209.0	1.3
232.4	1.9
250.9	80.5
287.5	12.0
295.7	1.0
317.3	0.5
353.7	0.9
364.9	2.8
386.7	0.3
446.2	3.3
492.1	7.8
558.8	1.6
600.2	0.4
622.0	0.9
692.2	1.4
725.8	1.5
754.0	4.8
804.4	2.6
841.8	5.2
910.1	12.6
920.7	15.8
950.8	0.6
959.4	0.8
967.0	5.1
995.1	0.9
1016.4	7.5
1023.8	1.7
1065.6	1.9
1084.5	8.1
1109.7	25.9
1141.9	24.7
1175.3	30.8
1204.0	2.0
1210.1	25.1
1235.5	40.4
1257.9	6.3
1314.0	1.3
1320.0	16.5
1329.1	20.3
1359.0	1.4
1373.9	6.3
1393.7	21.5
1407.1	11.5
1412.1	8.2
1486.5	1.2
1492.9	1.6
1497.8	5.5
1506.3	14.7
1508.6	3.5
1512.1	1.3
1514.8	9.6
2426.0	74.6
3054.3	14.9
3058.6	33.5
3069.7	22.5
3073.7	14.9
3081.0	13.3
3112.2	5.7
3113.7	20.3
3122.7	27.1
3128.6	26.2
3131.1	25.9
3153.4	12.6
3157.5	20.2
3200.2	4.8
3871.5	30.3

Table 179: M06-2X/cc-pV(T+d)Z Frequencies and IR Intensities of cis-2-ethyl-2,3-phosphirane-1-methylcyclopentan-1-ol (H-bonded)

Frequency	IR Intensity
95.8	0.6
116.7	0.8
194.0	0.2
209.0	0.6
229.7	0.2
279.5	0.5
291.5	0.4
313.7	0.2
344.0	4.5
360.9	1.2
378.7	46.2
392.4	36.1
443.5	3.6
488.2	11.2
559.9	3.0
598.7	0.7
620.7	5.1
684.0	2.1
717.0	1.9
750.8	2.6
802.6	3.1
836.3	0.8
904.7	11.6
925.7	3.9
953.5	0.0
960.7	1.0
972.6	4.3
995.8	2.8
1017.9	33.6
1024.9	11.8
1064.9	2.6
1087.0	3.7
1105.7	11.8
1146.8	21.0
1168.4	9.0
1204.2	1.1
1227.7	34.9
1240.0	43.1
1248.3	15.7
1315.6	3.7
1324.9	12.6
1342.0	2.2
1361.2	2.8
1379.8	11.8
1384.4	50.0
1411.6	8.4
1419.2	34.4
1484.4	2.6
1489.9	4.5
1495.5	6.0
1505.0	14.2
1508.1	4.4
1510.4	0.9
1516.4	4.3
2444.2	63.4
3061.6	23.4
3068.8	10.8
3071.2	19.8
3072.1	21.8
3079.1	9.9
3110.2	6.0
3118.8	22.6
3131.8	23.8
3134.5	20.1
3145.4	24.4
3155.3	14.8
3160.9	14.1
3197.1	4.1
3836.3	20.0

Table 180: M06-2X/cc-pV(T+d)Z Frequencies and IR Intensities of trans-2-ethyl-2,3-phosphirane-1-methylcyclopentan-1-ol

Frequency	IR Intensity
79.2	0.1
105.9	0.3
189.7	0.5
207.4	0.1
227.8	1.2
247.3	5.9
269.0	72.3
277.2	9.7
291.8	5.0
324.6	0.8
366.6	9.1
406.5	2.1
415.8	4.9
475.6	3.3
565.0	3.3
591.3	1.5
622.5	3.2
678.7	9.0
728.0	2.3
762.6	2.3
815.8	14.2
843.5	3.5
909.0	4.0
915.2	11.1
948.9	3.4
963.9	14.3
970.5	5.3
1005.3	5.9
1018.8	2.1
1045.7	4.1
1070.6	1.7
1095.8	30.4
1101.7	20.9
1137.8	3.4
1178.2	26.6
1206.1	5.1
1212.9	11.9
1234.7	10.5
1251.9	28.2
1316.7	6.3
1321.8	12.2
1338.4	4.0
1362.4	2.0
1374.3	3.8
1390.4	9.9
1409.1	1.8
1413.5	34.9
1483.9	2.8
1487.2	3.0
1494.4	2.7
1496.7	1.5
1500.5	6.4
1501.8	10.4
1514.6	3.8
2455.5	58.3
3053.0	18.1
3062.7	18.9
3069.1	33.6
3069.5	23.6
3092.4	20.2
3097.0	15.5
3116.6	15.2
3122.8	15.6
3127.6	32.1
3136.0	17.1
3149.4	17.1
3178.8	6.1
3182.6	7.2
3867.7	28.2

Table 181: M06-2X/cc-pV(T+d)Z Frequencies and IR Intensities of cis-2,3-thiirane-1,2-dimethylcyclopentan-1-ol (not H-bonded)

Frequency	IR Intensity
123.1	0.2
210.1	35.4
216.5	11.5
223.4	41.9
242.6	9.4
277.4	0.8
329.1	2.0
333.3	1.0
358.5	0.3
383.6	0.9
434.9	6.4
498.1	11.7
573.5	3.1
620.9	0.9
629.9	2.3
679.4	14.2
775.6	0.8
836.4	6.0
914.5	2.6
943.4	4.7
950.0	2.4
985.3	0.9
990.6	8.1
1017.9	7.1
1060.0	1.7
1080.1	19.0
1103.0	34.4
1144.4	18.1
1188.8	36.9
1207.3	9.4
1238.0	1.7
1240.6	59.1
1269.0	2.1
1327.3	4.7
1333.6	22.4
1378.7	0.5
1398.7	33.1
1405.6	1.5
1414.8	6.5
1483.7	2.0
1489.3	11.8
1491.7	5.9
1498.7	11.0
1503.1	2.0
1514.5	1.1
3055.8	10.3
3061.6	25.3
3063.5	23.5
3094.6	22.6
3121.8	20.2
3124.4	18.2
3130.0	11.7
3150.8	9.7
3153.2	11.7
3156.5	8.7
3187.4	7.8
3874.2	34.1

Table 182: M06-2X/cc-pV(T+d)Z Frequencies and IR Intensities of cis-2,3-thiirane-1,2-dimethylcyclopentan-1-ol (H-bonded)

Frequency	IR Intensity
122.4	2.6
217.5	0.6
228.3	0.2
233.2	0.2
272.8	0.8
324.3	0.6
327.1	0.6
353.1	1.2
379.1	2.8
416.3	62.3
437.1	30.9
502.3	19.0
572.8	6.6
614.4	0.4
630.6	5.4
674.0	10.9
769.0	1.0
832.6	3.0
914.0	0.2
946.8	3.1
959.3	1.5
986.7	8.4
992.2	4.0
1020.8	32.5
1059.1	9.3
1083.0	13.3
1103.6	6.7
1147.3	29.8
1169.6	8.1
1218.3	6.8
1243.0	38.4
1253.2	40.8
1272.7	1.2
1326.3	1.3
1346.5	8.1
1381.7	56.1
1397.8	15.1
1416.5	7.6
1421.4	22.1
1482.3	4.2
1485.4	3.5
1491.1	13.0
1497.0	8.5
1501.7	4.6
1516.0	0.4
3062.6	21.1
3063.2	12.2
3068.5	17.6
3101.6	11.8
3125.3	17.0
3130.0	9.4
3143.7	13.0
3146.9	15.7
3155.1	11.1
3158.0	11.3
3187.7	6.1
3830.8	23.2

Table 183: M06-2X/cc-pV(T+d)Z Frequencies and IR Intensities of trans-2,3-thiirane-1,2-dimethylcyclopentan-1-ol

Frequency	IR Intensity
118.7	1.0
206.8	0.5
223.0	1.8
238.8	8.1
250.7	4.0
262.9	83.4
300.0	4.6
330.9	1.6
341.6	2.4
392.1	4.8
416.8	2.5
468.6	2.3
581.9	8.3
621.9	0.8
632.0	3.9
679.7	27.0
789.9	6.9
832.5	1.1
907.7	4.6
931.0	6.0
956.0	21.0
982.4	5.6
995.9	0.3
1018.7	6.5
1062.0	9.6
1079.2	53.2
1097.2	8.5
1143.4	1.3
1189.9	35.0
1209.6	9.5
1231.6	1.4
1237.5	13.4
1265.0	22.2
1327.8	4.7
1346.1	6.3
1378.8	10.8
1400.9	7.6
1410.0	26.9
1414.9	3.5
1476.9	4.1
1486.4	8.3
1491.1	4.1
1494.6	5.8
1500.1	6.7
1503.1	3.2
3055.6	17.0
3070.3	18.4
3075.5	20.9
3094.1	23.7
3124.7	15.0
3129.9	13.7
3139.1	10.8
3146.3	6.3
3150.7	17.0
3157.3	6.8
3193.4	6.2
3872.8	34.2

Table 184: M06-2X/cc-pV(T+d)Z Frequencies and IR Intensities of cis-2,3-thiirane-1-ethyl-2-methylcyclopentan-1-ol ((not H-bonded)

Frequency	IR Intensity
72.9	0.3
123.7	0.6
180.7	3.6
195.5	79.1
223.0	1.4
229.2	3.0
281.7	19.7
287.6	1.1
329.6	1.6
350.7	1.7
353.1	1.4
369.9	0.7
430.6	7.7
520.8	7.1
571.9	2.8
624.7	3.1
652.7	1.4
681.3	16.1
754.1	1.4
798.0	2.1
837.5	5.5
914.5	2.9
941.6	4.8
961.2	6.2
991.0	8.8
1015.9	9.9
1021.5	5.9
1043.3	16.1
1069.8	8.2
1079.5	10.2
1107.8	16.8
1143.7	16.1
1184.3	13.1
1198.8	17.9
1232.5	14.2
1234.3	43.1
1272.6	1.7
1311.9	3.9
1325.8	17.7
1331.3	0.3
1363.8	7.6
1383.6	0.2
1400.5	21.6
1412.7	2.4
1422.9	9.6
1478.9	3.0
1486.2	11.0
1497.3	8.7
1500.2	3.7
1502.3	4.8
1507.3	4.6
1517.3	9.5
3057.3	6.0
3061.2	25.7
3063.8	21.3
3067.9	24.8
3107.8	16.9
3115.0	7.0
3125.0	13.5
3131.6	21.6
3133.8	7.3
3143.2	30.2
3154.7	7.7
3157.0	16.9
3187.7	8.0
3881.6	33.6

Table 185: M06-2X/cc-pV(T+d)Z Frequencies and IR Intensities of cis-2,3-thiirane-1-ethyl-2-methylcyclopentan-1-ol ((H-bonded)

Frequency	IR Intensity
130.8	1.7
158.4	0.3
213.4	0.3
235.3	0.2
242.1	1.1
259.8	0.2
281.6	0.1
324.4	1.5
343.7	0.5
358.2	0.4
415.6	7.2
418.2	68.9
457.5	24.0
496.9	12.5
577.0	8.1
615.0	0.4
636.2	3.0
675.3	13.6
759.1	0.3
798.3	3.8
836.7	2.1
914.8	0.8
935.6	17.0
962.8	5.4
989.0	1.7
1008.7	8.3
1037.2	9.6
1064.7	9.6
1074.3	33.0
1085.5	8.7
1114.1	12.7
1149.1	17.0
1173.5	10.7
1202.2	30.3
1230.0	18.4
1242.3	6.0
1258.5	6.6
1313.1	2.3
1327.2	0.3
1333.5	1.2
1373.9	44.4
1397.6	36.1
1400.6	10.3
1417.4	5.0
1420.5	5.1
1483.0	1.0
1487.9	14.3
1494.2	9.6
1499.9	4.7
1506.3	9.6
1508.2	5.1
1521.7	4.1
3062.5	6.1
3064.9	19.1
3076.7	15.7
3080.3	29.2
3098.3	14.4
3110.1	1.8
3124.8	14.3
3128.9	25.6
3134.1	16.6
3140.3	32.1
3143.8	11.8
3153.5	7.0
3187.6	5.8
3828.0	23.8

Table 186: M06-2X/cc-pV(T+d)Z Frequencies and IR Intensities of trans-2,3-thiirane-1-ethyl-2-methylcyclopentan-1-ol

Frequency	IR Intensity
90.8	0.1
114.5	0.9
174.2	1.1
211.6	0.2
236.2	3.3
244.6	17.9
280.1	51.3
297.7	14.4
303.4	24.6
331.6	0.7
349.4	2.3
416.0	4.6
423.7	1.4
485.4	1.8
575.5	6.5
621.7	4.0
654.7	2.3
678.8	24.9
780.2	8.4
798.2	1.2
844.1	0.8
914.3	2.6
952.3	15.3
974.8	4.7
984.7	20.8
1002.6	30.5
1019.1	6.8
1036.3	2.1
1066.1	5.8
1083.0	25.6
1104.2	2.0
1147.0	1.2
1190.4	28.8
1202.8	7.9
1228.5	0.7
1236.9	18.9
1259.0	15.7
1317.6	5.8
1323.6	7.7
1344.3	3.8
1353.9	6.3
1387.4	9.1
1402.5	14.9
1414.2	5.2
1423.6	3.6
1478.2	3.7
1484.3	6.3
1488.6	3.2
1492.2	6.1
1503.2	7.4
1505.8	8.1
1515.2	6.1
3058.6	14.4
3067.6	11.7
3071.0	25.4
3075.3	21.9
3092.2	20.2
3105.8	1.3
3128.5	27.8
3129.1	12.6
3138.9	14.1
3140.2	19.9
3143.1	14.8
3156.7	6.2
3194.7	6.4
3878.3	31.2

Table 187: M06-2X/cc-pV(T+d)Z Frequencies and IR Intensities of cis-2-ethyl-2,3-thiirane-1-methylcyclopentan-1-ol (not H-bonded)

Frequency	IR Intensity
85.9	0.3
112.0	0.2
186.9	1.5
209.7	2.0
229.3	66.9
235.6	22.5
281.2	5.2
295.2	0.9
336.2	1.3
357.1	1.0
369.8	1.6
388.2	1.0
445.8	5.0
506.3	11.0
568.3	2.6
626.1	3.5
636.1	1.4
715.6	13.9
756.5	1.8
810.2	3.9
841.0	10.5
913.0	7.4
945.5	2.4
951.5	1.1
968.3	5.7
997.1	5.0
1017.2	2.4
1030.9	4.4
1068.1	1.6
1085.1	9.8
1110.3	31.9
1145.3	19.1
1186.3	37.4
1207.4	7.8
1212.1	8.0
1238.5	48.2
1264.0	3.1
1313.9	4.8
1324.3	3.2
1332.8	23.2
1362.2	2.3
1383.8	1.7
1397.9	23.8
1406.4	6.2
1413.4	9.1
1482.9	3.1
1489.0	1.4
1495.9	8.5
1499.1	9.6
1505.9	4.0
1511.0	4.2
1515.2	9.0
3055.7	11.7
3058.3	26.7
3071.5	19.0
3076.6	13.8
3094.2	23.7
3122.0	18.3
3123.8	11.6
3124.6	19.0
3134.4	21.6
3151.4	10.1
3153.4	12.9
3159.9	18.5
3191.3	7.1
3874.3	34.1

Table 188: M06-2X/cc-pV(T+d)Z Frequencies and IR Intensities of cis-2-ethyl-2,3-thiirane-1-methylcyclopentan-1-ol (H-bonded)

Frequency	IR Intensity
89.7	0.6
110.0	1.4
181.4	0.6
207.0	0.2
235.2	0.8
275.0	0.1
291.5	0.4
331.1	0.2
351.3	2.5
369.2	0.6
384.6	3.7
423.9	64.0
447.0	28.3
507.2	18.4
569.2	5.7
618.8	2.9
638.9	2.2
707.8	12.9
757.1	1.0
809.5	3.0
836.6	6.8
916.1	2.2
946.5	3.9
958.0	1.1
973.2	4.1
997.7	11.3
1022.3	15.9
1031.9	17.3
1069.3	7.9
1087.1	7.7
1108.0	9.0
1151.6	19.7
1170.0	10.2
1211.0	2.5
1233.5	23.8
1248.5	54.3
1253.3	5.6
1323.3	2.9
1323.6	9.9
1349.1	3.0
1363.9	1.9
1384.7	55.8
1398.7	6.2
1413.1	8.4
1421.2	29.0
1482.9	3.3
1485.9	6.1
1493.3	4.3
1499.7	12.6
1505.5	4.5
1511.0	3.5
1516.1	4.0
3059.5	20.4
3069.1	6.7
3071.6	18.6
3075.5	18.1
3102.6	12.6
3121.2	2.9
3126.4	18.7
3134.9	21.8
3145.3	15.0
3147.8	17.1
3154.2	17.4
3164.7	13.7
3192.2	5.6
3828.9	23.6

Table 189: M06-2X/cc-pV(T+d)Z Frequencies and IR Intensities of trans-2-ethyl-2,3-thiirane-1-methylcyclopentan-1-ol

Frequency	IR Intensity
61.1	0.1
111.8	0.8
191.8	0.8
215.2	0.3
228.6	2.6
248.5	4.3
265.7	79.5
273.5	8.1
299.9	3.8
332.3	1.3
371.5	3.9
407.6	5.9
415.8	1.7
483.2	4.4
576.4	7.1
624.0	2.4
635.2	1.5
712.2	23.0
764.1	2.5
816.4	13.6
842.2	5.3
903.7	9.9
934.7	3.3
962.8	16.2
970.3	4.6
997.9	9.9
1014.3	1.8
1040.3	3.9
1067.4	0.3
1096.6	14.5
1099.8	38.8
1143.8	1.8
1186.5	31.1
1205.7	4.2
1214.1	2.2
1234.1	12.5
1259.7	21.8
1316.1	11.8
1325.9	4.8
1345.0	4.0
1362.0	1.0
1386.7	5.1
1397.8	2.2
1409.4	2.1
1412.6	36.0
1476.4	3.2
1484.5	5.0
1490.8	5.3
1495.4	3.5
1499.2	2.1
1501.3	8.9
1516.3	5.0
3055.3	16.5
3069.4	23.6
3071.5	21.3
3074.0	20.1
3094.0	23.2
3116.9	10.0
3124.4	11.9
3128.6	18.4
3130.4	28.7
3146.0	9.0
3153.6	14.6
3178.3	7.0
3195.9	5.5
3869.5	33.2

Table 190: MP2 optimized geometry of cis-2,3-epoxy-1,2-dimethylcyclopentan-1-ol (not H-bonded) (in Å) with the TZ basis set

Atom	x	y	z
C	−0.250901	0.781870	−0.024895
C	−1.547468	0.186649	−0.359618
C	−1.422827	−1.314060	−0.432466
C	−0.124090	−1.582449	0.342633
C	0.757903	−0.345849	0.097982
C	1.555919	−0.478816	−1.192046
H	2.232575	−1.331970	−1.120704
H	0.898297	−0.643499	−2.046037
H	2.149846	0.414706	−1.371885
O	1.621266	−0.045956	1.189916
H	2.275369	−0.751099	1.242531
H	−0.331988	−1.632847	1.408183
H	0.371060	−2.502917	0.034507
H	−2.282290	−1.824498	0.000935
H	−1.341000	−1.621813	−1.476728
O	−1.292999	0.685257	0.969564
H	−2.301678	0.727579	−0.917076
C	0.184639	2.169807	−0.368139
H	0.640758	2.210347	−1.355892
H	−0.674676	2.836057	−0.351039
H	0.912754	2.516872	0.363866

Table 191: MP2 optimized geometry of cis-2,3-epoxy-1,2-dimethylcyclopentan-1-ol (H-bonded) (in Å) with the TZ basis set

Atom	x	y	z
C	−0.020445	−0.002940	0.021310
C	1.522366	−0.032406	0.012174
C	1.975240	1.433264	0.024066
C	0.851559	2.140495	−0.687775
C	−0.353801	1.314232	−0.661950
C	−1.757745	1.819656	−0.728508
H	−2.408078	1.053012	−1.148763
H	−2.128485	2.077756	0.262190
H	−1.801222	2.703076	−1.361304
O	0.422326	1.444971	−1.882478
H	0.806238	3.220066	−0.754637
H	2.050111	1.823294	1.041081
H	2.939063	1.580723	−0.462007
H	1.914490	−0.609570	0.847203
H	1.850204	−0.508823	−0.909421
O	−0.592472	−1.106943	−0.670991
H	−0.272987	−1.044052	−1.579916
C	−0.579521	−0.047353	1.428922
H	−0.290875	−0.984461	1.901653
H	−0.189562	0.779577	2.022401
H	−1.665925	0.009473	1.413966

Table 192: MP2 optimized geometry of trans-2,3-epoxy-1,2-dimethylcyclopentan-1-ol (in Å) with the TZ basis set

Atom	x	y	z
C	0.724709	−0.411326	0.005455
C	−0.228458	−1.587610	0.280719
C	−1.570646	−1.183894	−0.348661
C	−1.566362	0.318299	−0.217065
C	−0.199550	0.800157	−0.015101
O	−1.124149	0.764707	1.066146
H	−2.328335	0.932729	−0.679865
H	−1.604658	−1.442717	−1.407632
H	−2.422662	−1.648393	0.146468
H	0.175099	−2.511972	−0.132194
H	−0.343889	−1.717250	1.354971
C	0.324530	2.141380	−0.418269
H	−0.491807	2.858745	−0.477817
H	1.044317	2.503844	0.317371
H	0.820879	2.072957	−1.384305
C	1.845109	−0.293867	1.021539
H	2.415062	−1.224395	1.073482
H	2.525265	0.511382	0.744558
H	1.435716	−0.089996	2.010890
O	1.240252	−0.481898	−1.322745
H	1.870299	−1.206222	−1.364824

Table 193: MP2 optimized geometry of cis-2,3-epoxy-1-ethyl-2-methylcyclopentan-1-ol (not H-bonded) (in Å) with the TZ basis set

Atom	x	y	z
C	−0.914661	0.589153	−0.104990
C	−1.700862	−0.547520	−0.592209
C	−0.894959	−1.815630	−0.476451
C	0.190837	−1.433005	0.540491
C	0.449945	0.067483	0.313812
C	1.460821	0.317347	−0.809476
C	2.882300	−0.114927	−0.469707
H	2.947760	−1.179262	−0.249443
H	3.552707	0.087819	−1.302866
H	3.263556	0.437843	0.388300
H	1.117543	−0.199830	−1.709024
H	1.463864	1.384121	−1.033600
O	0.831267	0.759048	1.499507
H	1.619806	0.328457	1.845452
H	−0.194050	−1.549925	1.550427
H	1.091837	−2.034794	0.438407
H	−1.495838	−2.662459	−0.146081
H	−0.466949	−2.066547	−1.448986
O	−1.976628	0.030585	0.699472
H	−2.487423	−0.431220	−1.327133
C	−1.109013	2.015227	−0.510782
H	−0.559907	2.252522	−1.420088
H	−2.166917	2.199602	−0.683040
H	−0.763506	2.673810	0.285237

Table 194: MP2 optimized geometry of cis-2,3-epoxy-1-ethyl-2-methylcyclopentan-1-ol (H-bonded) (in Å) with the TZ basis set

Atom	x	y	z
C	0.429518	0.099923	0.657952
C	−0.644184	0.601437	−0.294617
C	−1.367963	−0.543167	−0.845787
C	−0.755153	−1.830145	−0.363654
C	0.078743	−1.395220	0.851736
H	0.974238	−1.999713	0.988496
H	−0.515841	−1.482271	1.758516
H	−0.133919	−2.246806	−1.157253
H	−1.510933	−2.572030	−0.106480
O	−1.948436	0.229254	0.230879
H	−1.906545	−0.483813	−1.783478
C	−0.562691	1.960882	−0.907450
H	−1.481883	2.178759	−1.446599
H	−0.427153	2.709175	−0.126910
H	0.276645	2.031305	−1.597962
O	0.376456	0.824494	1.884254
H	−0.539719	0.761274	2.184167
C	1.833786	0.326004	0.120213
C	2.123125	−0.384301	−1.195846
H	1.419647	−0.092311	−1.975411
H	3.124778	−0.142772	−1.546871
H	2.068139	−1.466211	−1.085343
H	1.984165	1.401214	0.014056
H	2.528982	−0.010911	0.890898

Table 195: MP2 optimized geometry of trans-2,3-epoxy-1-ethyl-2-methylcyclopentan-1-ol (in Å) with the TZ basis set

Atom	x	y	z
C	−0.467700	−0.013654	0.207084
C	0.882089	0.635251	−0.054516
C	1.900011	−0.415073	−0.026042
C	1.259718	−1.742787	0.269965
C	−0.213775	−1.501402	−0.089762
H	−0.892433	−2.135417	0.479614
H	−0.375724	−1.693606	−1.148551
H	1.376115	−1.958154	1.332803
H	1.699832	−2.561513	−0.298078
O	1.459464	0.150338	−1.285237
H	2.930341	−0.204049	0.229669
C	1.115862	2.072721	0.275979
H	2.162345	2.324245	0.118436
H	0.510697	2.715631	−0.362990
H	0.848624	2.263869	1.313001
C	−1.582771	0.598131	−0.627247
C	−2.930855	−0.088587	−0.441106
H	−2.888132	−1.137278	−0.731524
H	−3.266934	−0.035771	0.595327
H	−3.697691	0.391747	−1.045943
H	−1.669053	1.652584	−0.359827
H	−1.278893	0.548629	−1.674635
O	−0.681333	0.194440	1.609724
H	−1.501407	−0.254792	1.840617

Table 196: MP2 optimized geometry of cis-2-ethyl-2,3- epoxy-1-methylcyclopentan-1-ol (not H-bonded) (in Å) with the TZ basis set

Atom	x	y	z
C	−0.397907	−0.288417	0.369767
C	0.081462	−1.563664	−0.174268
C	1.414953	−1.366858	−0.850458
C	1.912055	−0.051097	−0.234312
C	0.643257	0.771852	0.053123
C	0.232093	1.598109	−1.157121
H	1.017368	2.320548	−1.387504
H	0.086687	0.970259	−2.036384
H	−0.686427	2.145966	−0.956692
O	0.766866	1.609013	1.199246
H	1.394853	2.305901	0.979994
H	2.398338	−0.248361	0.717333
H	2.605597	0.484687	−0.881917
H	2.098519	−2.196650	−0.673066
H	1.266186	−1.278227	−1.928497
O	0.150094	−1.307227	1.241012
H	−0.595361	−2.357145	−0.465133
C	−1.841248	0.068834	0.600540
C	−2.710144	0.014165	−0.654042
H	−2.700905	−0.985808	−1.085756
H	−3.742857	0.263696	−0.414481
H	−2.369860	0.709222	−1.417933
H	−2.225133	−0.634013	1.339416
H	−1.881894	1.059834	1.055493

Table 197: MP2 optimized geometry of cis-2-ethyl-2,3- epoxy-1-methylcyclopentan-1-ol (H-bonded) (in Å) with the TZ basis set

Atom	x	y	z
C	−1.844531	−0.023744	0.599392
C	−0.392164	−0.307668	0.342810
C	0.138149	−1.569574	−0.172890
C	1.428013	−1.321231	−0.910855
C	1.892041	0.027468	−0.346798
C	0.610854	0.797943	0.042421
C	0.145840	1.716281	−1.068713
H	−0.779406	2.218616	−0.793691
H	0.907878	2.474599	−1.241452
H	−0.010521	1.159508	−1.992255
O	0.801758	1.629337	1.182363
H	1.035818	1.033530	1.905539
H	2.479795	−0.134770	0.554062
H	2.500619	0.596946	−1.046574
H	2.158819	−2.115003	−0.759151
H	1.216583	−1.260130	−1.980355
O	0.236015	−1.271013	1.238287
H	−0.503642	−2.408433	−0.411419
H	−2.200140	−0.783909	1.295007
H	−1.924118	0.938112	1.109682
C	−2.708700	−0.038856	−0.659494
H	−2.399106	0.718330	−1.375461
H	−2.652268	−1.008600	−1.152516
H	−3.751768	0.147564	−0.408494

Table 198: MP2 optimized geometry of trans-2-ethyl-2,3- epoxy-1-methylcyclopentan-1-ol (in Å) with the TZ basis set

Atom	x	y	z
C	0.556540	0.796479	−0.022995
C	1.887837	0.079471	−0.304794
C	1.502989	−1.318243	−0.809218
C	0.221590	−1.593717	−0.069993
C	−0.379606	−0.334957	0.376426
C	−1.851559	−0.137219	0.596361
H	−2.006602	0.748131	1.216642
C	−2.663545	−0.026050	−0.691512
H	−2.515675	−0.908697	−1.312853
H	−3.725749	0.049898	−0.463222
H	−2.365353	0.843147	−1.268822
H	−2.192550	−0.992071	1.181651
O	0.289791	−1.204921	1.321782
H	−0.377503	−2.473879	−0.266501
H	1.296930	−1.310793	−1.880261
H	2.273680	−2.062004	−0.610494
H	2.476348	0.643835	−1.027760
H	2.460987	−0.000895	0.615882
C	0.673562	1.872024	1.038198
H	0.967069	1.428800	1.988745
H	1.430211	2.606377	0.755476
H	−0.275840	2.390145	1.163350
O	0.005230	1.325403	−1.236437
H	0.571469	2.057991	−1.504133

Table 199: MP2 optimized geometry of cis-2,3- aziridine-1,2-dimethylcyclopentan-1-ol (not H-bonded) (in Å) with the TZ basis set

Atom	x	y	z
C	0.972861	0.006021	0.223197
C	−0.321019	0.610065	−0.291120
C	−1.097081	−0.475700	−0.930889
C	−0.339685	−1.776638	−0.812112
C	0.646660	−1.493931	0.331719
C	−0.366473	2.045494	−0.716673
N	−1.492967	0.031201	0.407678
C	2.104716	0.250106	−0.766400
O	1.277146	0.607572	1.478750
H	3.007897	−0.259432	−0.426388
H	1.852766	−0.137408	−1.753948
H	2.323640	1.312524	−0.849753
H	2.112595	0.234520	1.780208
H	0.158873	−1.654889	1.289341
H	1.544316	−2.110159	0.279558
H	−0.992719	−2.621834	−0.595352
H	0.184628	−1.984557	−1.746516
H	−1.745004	−0.275550	−1.773686
H	0.332486	2.248800	−1.526582
H	−1.366294	2.304927	−1.065658
H	−0.115616	2.692794	0.123726
H	−2.263641	0.682854	0.280466

Table 200: MP2 optimized geometry of cis-2,3- aziridine-1,2-dimethylcyclopentan-1-ol (H-bonded) (in Å) with the TZ basis set

Atom	x	y	z
C	0.294684	−0.753316	−0.059863
C	−0.789268	0.301478	0.115259
C	0.003105	1.621486	0.243700
C	1.312311	1.412524	−0.528184
C	1.558337	−0.069324	−0.391383
C	−0.058147	−2.145530	−0.480574
C	−1.739194	0.329316	−1.064549
N	1.318625	−0.549805	0.999816
O	−1.582729	0.033169	1.265740
H	−0.778947	−2.577523	0.214319
H	−0.495358	−2.161119	−1.477856
H	0.830521	−2.777182	−0.493723
H	2.325007	−0.566319	−0.970759
H	1.193696	1.658365	−1.585035
H	2.129842	2.014527	−0.131969
H	−0.575980	2.469703	−0.117088
H	0.221510	1.784320	1.296690
H	−0.947720	−0.062460	1.988243
H	−2.468388	1.124950	−0.922021
H	−1.193482	0.515763	−1.989688
H	−2.274426	−0.613927	−1.152938
H	1.818429	−1.429042	1.109821

Table 201: MP2 optimized geometry of trans-2,3- aziridine-1,2-dimethylcyclopentan-1-ol (in Å) with the TZ basis set

Atom	x	y	z
N	−1.171495	0.680614	1.115587
C	−1.564277	0.251986	−0.256699
C	−1.474014	−1.242755	−0.424716
C	−0.134207	−1.582452	0.245202
C	0.760921	−0.357474	0.002043
C	−0.216013	0.804593	−0.009893
C	1.865878	−0.207272	1.027894
O	1.291355	−0.381771	−1.331283
C	0.238804	2.156086	−0.464060
H	−2.312125	0.833106	−0.778899
H	−1.447668	−1.480003	−1.488479
H	−2.310502	−1.772271	0.030461
H	0.328686	−2.482235	−0.160498
H	−0.276084	−1.719290	1.314643
H	−0.609864	2.836609	−0.538082
H	0.954047	2.583915	0.240025
H	0.716745	2.086106	−1.438621
H	2.492635	−1.100732	1.053124
H	2.497590	0.645867	0.784251
H	1.434344	−0.063283	2.017293
H	1.915918	−1.114330	−1.370163
H	−1.565159	1.605379	1.273608

Table 202: MP2 optimized geometry of cis-2,3- aziridine-1-ethyl-2-methylcyclopentan-1-ol (not H-bonded) (in Å) with the TZ basis set

Atom	x	y	z
N	−2.001035	−0.016196	0.690641
C	−0.913349	0.602392	−0.105393
C	0.442352	0.045768	0.298370
C	0.169340	−1.462795	0.440143
C	−0.894554	−1.782745	−0.621223
C	−1.704215	−0.510210	−0.677458
C	−1.056186	2.035589	−0.519657
C	1.472125	0.343589	−0.796222
C	2.878056	−0.152657	−0.479974
O	0.825420	0.666530	1.523413
H	2.915757	−1.233348	−0.353609
H	3.564389	0.105804	−1.284469
H	3.260633	0.312764	0.427961
H	1.122000	−0.095363	−1.733927
H	1.509772	1.423314	−0.940824
H	1.586179	0.185770	1.865385
H	−0.242857	−1.626960	1.432501
H	1.068760	−2.065535	0.327985
H	−1.496706	−2.650387	−0.351666
H	−0.439258	−1.975201	−1.594389
H	−2.427775	−0.335383	−1.462418
H	−0.460650	2.266893	−1.401092
H	−2.097780	2.255518	−0.755809
H	−0.741021	2.692958	0.290916
H	−2.800983	0.609333	0.631562

Table 203: MP2 optimized geometry of cis-2,3- aziridine-1-ethyl-2-methylcyclopentan-1-ol (H-bonded) (in Å) with the TZ basis set

Atom	x	y	z
N	−1.957282	0.060652	0.736250
C	−0.918788	0.612722	−0.176605
C	0.434059	0.054724	0.251074
C	0.174071	−1.464679	0.371871
C	−0.967878	−1.785435	−0.600771
C	−1.765123	−0.506395	−0.628978
C	−1.050374	2.024604	−0.656640
C	1.513464	0.393822	−0.768083
C	2.899309	−0.112736	−0.387807
O	0.845339	0.631624	1.486789
H	2.943275	−1.199915	−0.362880
H	3.640366	0.228921	−1.108743
H	3.178405	0.259701	0.594738
H	1.209074	−0.009410	−1.737671
H	1.550979	1.479207	−0.869015
H	0.097765	0.493174	2.083955
H	−0.145935	−1.669712	1.391013
H	1.069307	−2.050834	0.180201
H	−1.557410	−2.644265	−0.280059
H	−0.588640	−1.991744	−1.603402
H	−2.551940	−0.350905	−1.355116
H	−0.508137	2.183474	−1.587417
H	−2.098342	2.268349	−0.834192
H	−0.657135	2.715030	0.090280
H	−2.757288	0.687913	0.697029

Table 204: MP2 optimized geometry of trans-2,3- aziridine-1-ethyl-2-methylcyclopentan-1-ol (in Å) with the TZ basis set

Atom	x	y	z
N	1.473318	0.092099	−1.313359
C	0.888302	0.640430	−0.067143
C	−0.461339	−0.000783	0.206057
C	−0.208108	−1.497866	−0.040126
C	1.257815	−1.730265	0.357300
C	1.914760	−0.419146	0.014839
C	−1.573539	0.584438	−0.651124
C	−2.925588	−0.087851	−0.442776
O	−0.688276	0.245808	1.601715
C	1.124646	2.070117	0.307835
H	−0.900270	−2.113033	0.534523
H	−0.347484	−1.718881	−1.096318
H	1.345810	−1.902558	1.430295
H	1.698306	−2.576926	−0.168638
H	2.919728	−0.190121	0.342553
H	2.176730	2.325996	0.178730
H	0.534866	2.741757	−0.317560
H	0.849456	2.238304	1.346750
H	−2.886504	−1.147019	−0.693325
H	−3.263625	0.005872	0.590136
H	−3.688978	0.372146	−1.067759
H	−1.655759	1.648643	−0.422001
H	−1.265100	0.493955	−1.694062
H	−1.494438	−0.223173	1.842034
H	2.170282	0.760603	−1.633602

Table 205: MP2 optimized geometry of cis-2,3- aziridine-1-ethyl-2-methylcyclopentan-1-ol (not H-bonded) (in Å) with the TZ basis set

Atom	x	y	z
N	−0.253206	−1.407695	−1.169627
C	0.388399	−0.333889	−0.365492
C	−0.625927	0.754162	−0.053987
C	−1.885183	−0.034625	0.349430
C	−1.365297	−1.310497	1.028893
C	−0.074455	−1.578585	0.292089
C	1.836573	0.014207	−0.608128
C	2.714577	0.007157	0.641610
C	−0.155932	1.647931	1.085404
O	−0.805421	1.531823	−1.235492
H	−0.927851	2.387481	1.307166
H	0.025213	1.071994	1.992900
H	0.754342	2.178680	0.813233
H	−1.414103	2.246198	−1.017729
H	−2.417011	−0.291789	−0.562528
H	−2.541638	0.550871	0.993325
H	−2.070998	−2.137429	0.949766
H	−1.157526	−1.141760	2.087176
H	0.629600	−2.320123	0.644924
H	2.711215	−0.976879	1.108688
H	3.745378	0.252434	0.387715
H	2.373746	0.726143	1.381889
H	2.249029	−0.698503	−1.325655
H	1.873225	0.988836	−1.098635
H	0.497531	−1.911524	−1.635111

Table 206: MP2 optimized geometry of cis-2,3- aziridine-1-ethyl-2-methylcyclopentan-1-ol (H-bonded) (in Å) with the TZ basis set

Atom	x	y	z
N	0.274593	−1.308842	1.247180
C	−0.408447	−0.333982	0.346174
C	0.605835	0.761053	0.029202
C	1.859525	−0.022320	−0.426119
C	1.356335	−1.356883	−0.990251
C	0.096244	−1.608611	−0.200330
C	−1.865153	−0.021136	0.561403
C	−2.699799	0.003403	−0.717353
C	0.132390	1.722740	−1.040827
O	0.857052	1.553276	1.184914
H	−0.773507	2.238553	−0.728154
H	0.907647	2.468470	−1.210028
H	−0.058170	1.197904	−1.976470
H	1.070256	0.913214	1.877884
H	2.480112	−0.204538	0.448055
H	2.445618	0.549890	−1.143262
H	2.089271	−2.156428	−0.882921
H	1.102178	−1.271149	−2.048461
H	−0.582026	−2.407888	−0.468714
H	−2.284193	−0.773977	1.233840
H	−1.938090	0.933305	1.087540
H	−2.360791	0.770150	−1.408359
H	−2.643379	−0.956536	−1.229330
H	−3.746468	0.199702	−0.487955
H	−0.451994	−1.809368	1.753094

Table 207: MP2 optimized geometry of trans-2,3- aziridine-1-ethyl-2-methylcyclopentan-1-ol (in Å) with the TZ basis set

Atom	x	y	z
N	0.265768	−1.175693	1.385123
C	0.069565	−1.610822	−0.023814
C	1.329712	−1.450368	−0.834927
C	1.849190	−0.076962	−0.385419
C	0.591589	0.753738	−0.078322
C	−0.411845	−0.280754	0.409126
C	0.839836	1.862212	0.924935
O	0.039533	1.278304	−1.295201
C	−1.868548	0.044601	0.603517
C	−2.671511	0.191448	−0.686810
H	−1.957269	0.952032	1.205959
H	−2.576394	−0.705925	−1.297177
H	−3.727463	0.335975	−0.460335
H	−2.317593	1.032621	−1.272877
H	−2.304869	−0.760716	1.201482
H	−0.628246	−2.414114	−0.219381
H	1.074392	−1.442882	−1.894950
H	2.053488	−2.244964	−0.655480
H	2.454550	0.417735	−1.145357
H	2.445295	−0.181450	0.517767
H	1.148684	1.435682	1.877931
H	1.630315	2.527995	0.573049
H	−0.063115	2.453634	1.070990
H	0.673328	1.922736	−1.629940
H	−0.465521	−1.607059	1.945225

Table 208: MP2 optimized geometry of cis-2,3- phosphirane-1,2-dimethylcyclopentan-1-ol (not H-bonded) (in Å) with the TZ basis set

Atom	x	y	z
C	0.982539	−0.011737	0.246814
C	−0.333205	0.593398	−0.235412
C	−1.103524	−0.503469	−0.895890
C	−0.282096	−1.780567	−0.841585
C	0.691800	−1.520714	0.312110
C	−0.326755	2.002887	−0.762697
P	−1.809559	0.000277	0.777423
C	2.102202	0.276646	−0.747447
O	1.305401	0.549674	1.516398
H	3.012488	−0.237194	−0.432141
H	1.845515	−0.078599	−1.745620
H	2.309800	1.343111	−0.796610
H	2.196212	0.257634	1.740205
H	0.215866	−1.731723	1.270280
H	1.601913	−2.117129	0.247505
H	−0.887524	−2.673004	−0.687224
H	0.260964	−1.903091	−1.781541
H	−1.706829	−0.290071	−1.768662
H	0.310635	2.088136	−1.644541
H	−1.331259	2.312230	−1.046083
H	0.040409	2.692790	−0.003187
H	−2.676909	0.967295	0.217471

Table 209: MP2 optimized geometry of cis-2,3- phosphirane-1,2-dimethylcyclopentan-1-ol (H-bonded) (in Å) with the TZ basis set

Atom	x	y	z
C	0.972489	−0.000656	0.236688
C	−0.330592	0.599953	−0.302503
C	−1.096741	−0.499302	−0.947578
C	−0.279743	−1.778357	−0.865605
C	0.678936	−1.511539	0.298048
C	−0.313693	2.003427	−0.843163
P	−1.827652	0.021104	0.711697
C	2.130710	0.284445	−0.705125
O	1.360475	0.545878	1.489510
H	3.024438	−0.208974	−0.326302
H	1.918677	−0.090267	−1.706153
H	2.327510	1.352455	−0.760522
H	0.576851	0.505334	2.053420
H	0.191023	−1.735316	1.248472
H	1.593246	−2.100406	0.251590
H	−0.890979	−2.667372	−0.715485
H	0.275294	−1.906436	−1.797545
H	−1.702033	−0.300362	−1.822749
H	0.333797	2.070212	−1.718924
H	−1.312723	2.318869	−1.139138
H	0.056231	2.699646	−0.090627
H	−2.681500	0.998838	0.154166

Table 210: MP2 optimized geometry of trans-2,3- phosphirane-1,2-dimethylcyclopentan-1-ol (in Å) with the TZ basis set

Atom	x	y	z
C	0.700014	−0.384733	0.076517
C	−0.244832	−1.581730	0.257501
C	−1.554483	−1.155454	−0.418850
C	−1.593962	0.352883	−0.252379
C	−0.213762	0.836416	0.057593
H	−2.220519	0.933047	−0.919089
H	−1.505388	−1.375872	−1.486426
H	−2.425987	−1.663396	−0.007696
H	0.186964	−2.471845	−0.200519
H	−0.392182	−1.801686	1.313077
C	0.318757	2.108193	−0.554297
H	−0.437671	2.891950	−0.521221
H	1.192108	2.468944	−0.010875
H	0.601734	1.934716	−1.590991
C	1.819033	−0.322300	1.097250
H	2.438101	−1.220024	1.039769
H	2.453873	0.541860	0.908003
H	1.416726	−0.250390	2.106433
O	1.246425	−0.414119	−1.255513
H	1.845177	−1.168800	−1.292561
P	−1.428450	1.090629	1.468399
H	−1.206015	−0.135433	2.131106

Table 211: MP2 optimized geometry of cis-2,3- phosphirane-1-ethyl-2-methylcyclopentan-1-ol (not H-bonded) (in Å) with the TZ basis set

Atom	x	y	z
C	0.398618	−1.466133	0.347704
C	0.639086	0.053187	0.293939
C	−0.671813	0.601410	−0.271085
C	−1.354812	−0.525486	−0.975378
C	−0.485786	−1.766021	−0.866365
C	1.797933	0.413074	−0.644565
C	3.161258	−0.096555	−0.189067
O	0.849943	0.629569	1.580404
C	−0.707142	2.011600	−0.798205
P	−2.180846	−0.057228	0.652299
H	3.197024	−1.182165	−0.119532
H	3.933587	0.210866	−0.892107
H	3.438287	0.317556	0.780785
H	1.573008	0.027397	−1.642010
H	1.840963	1.498867	−0.722903
H	1.681726	0.280452	1.918530
H	−0.136801	−1.685952	1.272180
H	1.321456	−2.043134	0.354264
H	−1.060830	−2.683944	−0.748683
H	0.122382	−1.865219	−1.768644
H	−1.911138	−0.336541	−1.884306
H	−0.044665	2.128907	−1.657220
H	−1.713984	2.274976	−1.117509
H	−0.400419	2.716471	−0.025568
H	−3.054265	0.873450	0.042649

Table 212: MP2 optimized geometry of cis-2,3- phosphirane-1-ethyl-2-methylcyclopentan-1-ol (H-bonded) (in Å) with the TZ basis set

Atom	x	y	z
C	−0.358932	1.470869	0.740358
C	−0.573803	−0.056853	0.668962
C	0.465455	−0.524227	−0.356162
C	0.952109	0.663390	−1.105637
C	0.209384	1.897103	−0.619939
O	−0.410169	−0.690965	1.933589
C	−1.995538	−0.432686	0.256084
C	−2.491449	0.189202	−1.042168
C	0.313712	−1.883683	−0.982653
P	2.211175	0.043253	0.150440
H	−1.277396	1.993873	1.004727
H	0.365368	1.671699	1.531259
H	−0.589758	2.130147	−1.323237
H	0.853893	2.772398	−0.544745
H	1.227130	0.576197	−2.149309
H	1.147222	−2.099480	−1.649252
H	0.281692	−2.656659	−0.214920
H	−0.607641	−1.943472	−1.564246
H	0.533951	−0.642214	2.135737
H	−1.819278	−0.015365	−1.875360
H	−3.470498	−0.211561	−1.300214
H	−2.596187	1.269095	−0.953858
H	−2.048410	−1.520308	0.202555
H	−2.643424	−0.134093	1.082180
H	2.845758	−0.848661	−0.742558

Table 213: MP2 optimized geometry of trans-2,3- phosphirane-1-ethyl-2-methylcyclopentan-1-ol (in Å) with the TZ basis set

Atom	x	y	z
P	1.645722	0.077625	−1.764594
C	0.886648	0.630195	−0.143863
C	−0.473132	−0.013960	0.150446
C	−0.223074	−1.517825	−0.052992
C	1.224531	−1.729506	0.407830
C	1.917667	−0.437432	0.013601
C	−1.615878	0.552796	−0.684750
C	−2.971376	−0.070735	−0.371425
O	−0.691498	0.242910	1.541335
C	1.109133	2.034941	0.353394
H	−0.937376	−2.120884	0.507289
H	−0.334274	−1.773734	−1.108706
H	1.256628	−1.826419	1.494301
H	1.679932	−2.616650	−0.029609
H	2.874634	−0.196084	0.455311
H	2.147308	2.330901	0.207497
H	0.480124	2.749812	−0.178349
H	0.872428	2.093823	1.415459
H	−2.963215	−1.148434	−0.539700
H	−3.269019	0.112598	0.662650
H	−3.748979	0.355119	−1.003715
H	−1.658836	1.630209	−0.512098
H	−1.374355	0.403802	−1.738651
H	−1.480115	−0.233217	1.814408
H	2.641383	1.082697	−1.750097

Table 214: MP2 optimized geometry of cis-2-ethyl-2,3-phosphirane-methylcyclopentan-1-ol (not H-bonded) (in Å) with the TZ basis set

Atom	x	y	z
C	−0.315436	1.019677	−0.308004
C	0.334535	−0.359806	−0.192116
C	−0.284535	−1.037503	0.989188
C	−1.272577	−0.082203	1.637040
C	−1.617733	0.884804	0.500602
C	1.796825	−0.515437	−0.565782
C	2.769252	−0.299771	0.593766
P	−0.890002	−1.727772	−0.653114
C	0.562411	2.114850	0.287525
O	−0.540061	1.291590	−1.689759
H	1.512949	2.183818	−0.237127
H	0.048971	3.074465	0.193735
H	0.751362	1.941947	1.346266
H	−0.812583	2.213573	−1.758885
H	−2.378889	0.453804	−0.150517
H	−1.981714	1.849650	0.853974
H	−2.150531	−0.585140	2.041233
H	−0.781617	0.447448	2.457063
H	0.305770	−1.696738	1.611333
H	1.966671	−1.512418	−0.970268
H	2.007392	0.178415	−1.382304
H	2.687134	0.694727	1.024213
H	2.586369	−1.022408	1.388260
H	3.796751	−0.434045	0.256105
H	0.120394	−2.716439	−0.702316

Table 215: MP2/cc-pVTZ optimized geometry of cis-2-ethyl-2,3-phosphirane-methylcyclopentan-1-ol (H-bonded) (in Å)

Atom	x	y	z
C	−0.361744	1.002098	−0.260897
C	0.309154	−0.373996	−0.134071
C	−0.288789	−1.056208	1.045860
C	−1.265273	−0.103396	1.715763
C	−1.638788	0.865669	0.591246
C	1.761337	−0.532260	−0.538264
C	2.752262	−0.272309	0.596333
P	−0.935636	−1.747571	−0.582201
C	0.513234	2.122597	0.276781
O	−0.623463	1.359077	−1.611750
H	1.451470	2.181967	−0.270257
H	−0.017871	3.064406	0.145061
H	0.722742	1.982914	1.336103
H	−1.038633	0.585676	−2.016361
H	−2.430786	0.439779	−0.027170
H	−1.986297	1.833216	0.949279
H	−2.130188	−0.609483	2.143054
H	−0.751017	0.423917	2.522620
H	0.304882	−1.729395	1.650361
H	1.931608	−1.540156	−0.915408
H	1.949431	0.141296	−1.377243
H	2.667374	0.734994	0.994540
H	2.589551	−0.971091	1.416192
H	3.774409	−0.408547	0.244025
H	0.071058	−2.732682	−0.687620

Table 216: MP2 optimized geometry of trans-2-ethyl-2,3-phosphirane-methylcyclopentan-1-ol (in Å) with the TZ basis set

Atom	x	y	z
C	−0.372774	−0.292285	0.235565
C	0.454980	0.993612	0.175382
C	1.690954	0.601361	−0.646705
C	1.164691	−0.416550	−1.662993
C	0.105830	−1.167354	−0.877842
C	0.786884	1.593346	1.527585
O	−0.348822	1.914581	−0.583922
P	0.685039	−1.751872	0.815475
C	−1.835296	−0.209082	0.627606
C	−2.766633	0.147049	−0.530623
H	−1.939825	0.526215	1.427978
H	−2.689044	−0.592828	−1.326868
H	−3.802060	0.158785	−0.190173
H	−2.520569	1.119538	−0.943822
H	−2.147882	−1.164727	1.049200
H	−0.592266	−1.809743	−1.397810
H	0.684186	0.101253	−2.494718
H	1.948121	−1.061599	−2.058638
H	2.143095	1.478829	−1.109132
H	2.439870	0.142633	0.001307
H	1.368757	0.895441	2.126806
H	1.370919	2.507654	1.402095
H	−0.126108	1.845396	2.065131
H	0.136917	2.747528	−0.609286
H	−0.435799	−2.589190	1.015574

Table 217: MP2 optimized geometry of cis-2,3-thiirane- 1,2-dimethylcyclopentan-1-ol (not H-bonded) (in Å) with the TZ basis set

Atom	x	y	z
C	−0.257869	0.789219	0.044515
C	−1.566790	0.193540	−0.319015
C	−1.408103	−1.307650	−0.456111
C	−0.120689	−1.607653	0.320751
C	0.749703	−0.352266	0.140472
C	1.557700	−0.438799	−1.151018
H	2.218851	−1.306153	−1.108973
H	0.907238	−0.556894	−2.018292
H	2.167613	0.451395	−1.285921
O	1.613793	−0.097414	1.241118
H	2.305575	−0.767941	1.224480
H	−0.332346	−1.724274	1.380468
H	0.384974	−2.504135	−0.037649
H	−2.268391	−1.859611	−0.080791
H	−1.289850	−1.550723	−1.515152
S	−1.489747	0.805008	1.403082
H	−2.248014	0.732978	−0.964907
C	0.192044	2.133670	−0.454353
H	0.550169	2.067977	−1.482260
H	−0.634536	2.839519	−0.417694
H	0.999144	2.513576	0.170842

Table 218: MP2 optimized geometry of cis-2,3-thiirane- 1,2-dimethylcyclopentan-1-ol (H-bonded) (in Å) with the TZ basis set

Atom	x	y	z
C	−0.010344	−0.025642	−0.014502
C	1.530768	−0.040910	0.028892
C	1.952652	1.430616	0.055867
C	0.856018	2.135709	−0.714181
C	−0.352951	1.286771	−0.729179
C	−1.746873	1.839415	−0.650456
H	−2.465165	1.096945	−0.996614
H	−1.995840	2.106675	0.376946
H	−1.836217	2.725678	−1.274608
S	0.485792	1.421078	−2.365982
H	0.764181	3.213894	−0.674360
H	1.952304	1.817274	1.078182
H	2.942548	1.599927	−0.364679
H	1.895105	−0.602369	0.887175
H	1.903331	−0.522925	−0.872404
O	−0.561347	−1.159055	−0.664414
H	−0.245106	−1.115801	−1.576928
C	−0.594129	−0.028400	1.388454
H	−0.289415	−0.942010	1.895828
H	−0.234109	0.827195	1.959985
H	−1.680722	−0.001019	1.354191

Table 219: MP2 optimized geometry of trans-2,3-thiirane- 1,2-dimethylcyclopentan-1-ol (in Å) with the TZ basis set

Atom	x	y	z
C	0.721318	−0.399852	0.058642
C	−0.221914	−1.588751	0.286354
C	−1.534269	−1.175207	−0.389512
C	−1.572264	0.325397	−0.204375
C	−0.200017	0.819319	0.058550
S	−1.306162	0.903813	1.516936
H	−2.263678	0.931573	−0.775376
H	−1.490632	−1.380420	−1.460733
H	−2.405211	−1.679890	0.024929
H	0.202345	−2.497769	−0.140267
H	−0.371774	−1.748557	1.351581
C	0.314612	2.119476	−0.489535
H	−0.481360	2.861179	−0.493549
H	1.130555	2.502965	0.122328
H	0.682546	1.970261	−1.503051
C	1.863558	−0.320254	1.049256
H	2.468172	−1.228021	1.004305
H	2.505936	0.528657	0.820334
H	1.478830	−0.215466	2.062274
O	1.218782	−0.429308	−1.290659
H	1.801345	−1.194484	−1.355312

Table 220: MP2 optimized geometry of cis-2,3-thiirane- 1-ethyl-2-methylcyclopentan-1-ol (not H-bonded) (in Å) with the TZ basis set

Atom	x	y	z
C	−0.936218	0.593377	−0.040947
C	−1.724656	−0.551754	−0.557649
C	−0.876365	−1.805058	−0.490340
C	0.209075	−1.452173	0.533162
C	0.438223	0.060112	0.361678
C	1.440552	0.358183	−0.762184
C	2.861776	−0.112238	−0.472742
H	2.921898	−1.189496	−0.329474
H	3.520821	0.144510	−1.300280
H	3.265481	0.375242	0.414615
H	1.078900	−0.099535	−1.686722
H	1.458169	1.435707	−0.921511
O	0.832114	0.714615	1.561849
H	1.672373	0.332225	1.836161
H	−0.152781	−1.624485	1.543765
H	1.118773	−2.031600	0.388145
H	−1.448094	−2.689112	−0.212005
H	−0.438801	−1.985070	−1.475733
S	−2.293653	0.054557	1.071062
H	−2.424584	−0.406645	−1.370930
C	−1.067759	1.983238	−0.600250
H	−0.540221	2.076321	−1.549765
H	−2.117519	2.217210	−0.762078
H	−0.655974	2.709765	0.099403

Table 221: MP2 optimized geometry of cis-2,3-thiirane- 1-ethyl-2-methylcyclopentan-1-ol (H-bonded) (in Å) with the TZ basis set

Atom	x	y	z
C	0.409554	0.097174	0.694536
C	−0.683537	0.619880	−0.245079
C	−1.373901	−0.539816	−0.845707
C	−0.715864	−1.821544	−0.382502
C	0.083686	−1.404660	0.858763
H	0.990826	−1.993040	0.991195
H	−0.520609	−1.525799	1.755147
H	−0.058664	−2.176644	−1.177661
H	−1.440328	−2.607028	−0.172483
S	−2.368998	0.307204	0.444989
H	−1.796116	−0.468837	−1.840649
C	−0.505340	1.935765	−0.946514
H	−1.402499	2.187304	−1.508025
H	−0.321031	2.724415	−0.217697
H	0.341098	1.898579	−1.632881
O	0.419243	0.792500	1.933049
H	−0.487324	0.738876	2.266046
C	1.801657	0.335714	0.115305
C	2.087819	−0.374598	−1.201141
H	1.354183	−0.126807	−1.968266
H	3.067935	−0.087721	−1.578509
H	2.090665	−1.456319	−1.078789
H	1.940099	1.411859	0.007764
H	2.511246	0.007590	0.876618

Table 222: MP2 optimized geometry of trans-2,3-thiirane- 1-ethyl-2-methylcyclopentan-1-ol (in Å) with the TZ basis set

Atom	x	y	z
C	−0.466783	−0.012036	0.137242
C	0.885233	0.649156	−0.130872
C	1.916274	−0.410691	−0.037344
C	1.248960	−1.723079	0.304701
C	−0.212459	−1.507304	−0.106986
H	−0.900438	−2.119506	0.475651
H	−0.354317	−1.743561	−1.159597
H	1.321139	−1.865245	1.384665
H	1.707465	−2.578768	−0.188032
S	1.621083	0.190069	−1.745742
H	2.897700	−0.175455	0.354002
C	1.119374	2.050672	0.356258
H	2.151437	2.339865	0.168741
H	0.471513	2.756904	−0.161802
H	0.910841	2.107389	1.423217
C	−1.605013	0.574978	−0.682518
C	−2.954232	−0.076348	−0.401659
H	−2.936988	−1.142217	−0.625551
H	−3.251128	0.050562	0.640071
H	−3.735449	0.376050	−1.009631
H	−1.662641	1.643859	−0.469400
H	−1.348606	0.463114	−1.737092
O	−0.665674	0.221026	1.542651
H	−1.458900	−0.263932	1.795789

Table 223: MP2 optimized geometry of cis-2-ethyl-2,3- thiirane-1-methylcyclopentan-1-ol (not H-bonded) (in Å) with the TZ basis set

Atom	x	y	z
C	−0.470766	−0.207793	0.062078
C	−0.002122	−1.382243	−0.715192
C	1.442585	−1.167775	−1.120431
C	1.941160	−0.106606	−0.133469
C	0.707310	0.762167	0.164185
C	0.571995	1.868479	−0.878647
H	1.461640	2.500334	−0.852138
H	0.485654	1.456963	−1.884861
H	−0.296250	2.490850	−0.676075
O	0.711025	1.323463	1.471519
H	1.386024	2.011006	1.486671
H	2.263629	−0.569863	0.795278
H	2.763268	0.485541	−0.535240
H	2.029092	−2.084860	−1.090154
H	1.465462	−0.788889	−2.145495
S	−0.239433	−1.727204	1.064831
H	−0.678784	−1.910046	−1.374961
C	−1.861665	0.373191	−0.039336
C	−2.997997	−0.638601	−0.043571
H	−3.043454	−1.182068	0.896105
H	−3.947801	−0.128530	−0.197632
H	−2.878861	−1.367625	−0.844478
H	−1.988427	1.073767	0.788735
H	−1.906692	0.960964	−0.959362

Table 224: MP2 optimized geometry of cis-2-ethyl-2,3- thiirane-1-methylcyclopentan-1-ol (H-bonded) (in Å) with the TZ basis set

Atom	x	y	z
C	−1.855254	0.001840	0.579612
C	−0.382635	−0.309381	0.431102
C	0.122496	−1.574219	−0.143715
C	1.374140	−1.290855	−0.947729
C	1.884753	0.033175	−0.374131
C	0.625444	0.797874	0.085287
C	0.112664	1.699327	−1.024848
H	−0.808526	2.196818	−0.729817
H	0.865140	2.461794	−1.219942
H	−0.059214	1.134963	−1.940452
O	0.867614	1.660927	1.184752
H	1.121587	1.082764	1.917147
H	2.520201	−0.150050	0.489334
H	2.455551	0.614646	−1.095927
H	2.107175	−2.093840	−0.888146
H	1.085069	−1.176722	−1.995837
S	0.336618	−1.476554	1.676565
H	−0.566521	−2.346469	−0.461358
H	−2.285121	−0.712240	1.280436
H	−1.948406	0.987617	1.039977
C	−2.639457	−0.063683	−0.730880
H	−2.291792	0.661420	−1.461098
H	−2.560796	−1.054803	−1.176396
H	−3.694911	0.130955	−0.544492

Table 225: MP2 optimized geometry of trans-2-ethyl-2,3- thiirane-1-methylcyclopentan-1-ol (in Å) with the TZ basis set

Atom	x	y	z
C	0.565237	0.805988	0.047825
C	1.870570	0.089211	−0.330283
C	1.426106	−1.276524	−0.864926
C	0.185820	−1.587475	−0.056514
C	−0.379447	−0.321996	0.470815
C	−1.870618	−0.084209	0.568258
H	−2.042608	0.877989	1.054656
C	−2.599266	−0.133589	−0.774079
H	−2.430133	−1.090767	−1.266780
H	−3.672378	−0.027565	−0.617669
H	−2.260454	0.654845	−1.437173
H	−2.281713	−0.845821	1.230444
S	0.376222	−1.400016	1.756862
H	−0.473276	−2.392756	−0.354198
H	1.132961	−1.197954	−1.913790
H	2.197098	−2.040084	−0.776613
H	2.418660	0.671296	−1.071217
H	2.501464	−0.029612	0.547370
C	0.744376	1.889524	1.091119
H	1.148077	1.469073	2.010198
H	1.438405	2.650129	0.727875
H	−0.207705	2.372708	1.305548
O	−0.045304	1.349808	−1.135472
H	0.518158	2.076055	−1.426740

Table 226: MP2 Frequencies and IR Intensities of cis-2,3-epoxy-1,2-dimethylcyclopentan-1-ol (not H-bonded) with the TZ basis set

Frequency	IR Intensity
130.9	0.8
193.8	1.5
212.0	0.7
228.6	85.2
242.0	8.7
268.9	1.9
300.7	0.5
356.7	2.4
375.5	2.4
417.2	1.4
471.8	12.8
527.4	13.6
580.8	5.6
654.7	1.6
677.4	4.2
797.2	6.0
833.0	3.2
876.9	21.7
940.3	5.4
943.3	8.7
951.3	1.8
991.2	3.1
1005.2	9.8
1037.7	4.5
1075.0	12.2
1094.7	48.5
1119.4	8.8
1160.9	23.3
1206.4	9.4
1224.5	43.7
1237.6	12.3
1259.4	17.6
1301.7	4.2
1328.9	3.4
1337.7	7.7
1398.3	20.8
1404.4	2.9
1408.1	7.1
1471.0	6.2
1486.6	4.0
1496.9	9.9
1502.9	6.3
1504.2	7.3
1512.9	7.4
1519.6	0.2
3063.6	12.7
3080.0	14.8
3083.2	23.8
3104.5	25.5
3139.4	15.5
3147.8	21.0
3166.7	11.3
3173.8	9.7
3175.8	10.7
3181.1	10.3
3197.0	20.3
3829.8	26.8

Table 227: MP2 Frequencies and IR Intensities of cis-2,3-epoxy-1,2-dimethylcyclopentan-1-ol (H-bonded) with a TZ basis set

Frequency	IR Intensity
112.2	3.3
200.3	0.1
209.6	1.2
232.8	1.3
262.7	0.8
292.0	0.6
349.0	4.3
368.8	2.4
409.8	37.7
430.8	38.0
472.6	30.7
532.3	14.2
587.4	4.7
657.0	1.5
671.5	5.2
790.6	3.8
828.2	3.6
876.5	18.9
937.7	5.0
948.5	4.2
956.6	7.6
990.2	19.4
1005.2	11.3
1040.1	14.2
1072.5	13.8
1109.2	2.9
1120.8	17.4
1163.3	41.4
1179.7	3.4
1215.8	39.3
1244.0	6.2
1292.5	13.3
1311.9	1.2
1331.2	2.0
1344.6	0.7
1386.3	32.5
1406.2	11.0
1423.5	30.6
1473.8	5.1
1487.0	7.6
1495.2	9.8
1500.8	5.6
1503.9	5.3
1511.7	5.0
1521.8	0.0
3075.5	8.0
3078.1	15.7
3083.5	24.0
3109.1	16.3
3139.8	16.3
3164.0	8.6
3168.0	12.5
3168.1	16.4
3177.0	15.5
3180.6	8.2
3198.6	19.5
3800.7	27.3

Table 228: MP2 Frequencies and IR Intensities of trans-2,3-epoxy-1,2-dimethylcyclopentan-1-ol with a TZ basis set

Frequency	IR Intensity
118.4	2.1
190.6	0.3
221.0	5.0
243.2	3.8
258.0	62.6
261.9	1.2
277.7	29.8
347.7	2.1
377.5	6.7
418.9	1.3
470.0	13.3
504.1	1.8
596.4	2.6
648.9	1.4
686.7	2.3
797.4	5.8
838.9	4.9
866.9	8.1
934.2	23.9
943.4	10.6
950.4	10.7
988.0	2.6
1011.9	5.9
1037.1	2.3
1079.6	31.3
1090.8	41.4
1117.1	4.0
1160.3	2.3
1209.1	10.4
1217.2	59.1
1239.9	3.4
1256.9	4.7
1301.3	4.1
1335.7	4.6
1346.8	2.8
1399.0	6.8
1403.0	12.3
1413.3	22.7
1473.2	4.1
1482.5	5.4
1493.4	6.7
1497.1	10.7
1503.6	3.5
1514.7	5.5
1517.8	2.2
3063.5	15.7
3078.9	16.3
3091.9	24.7
3103.9	24.4
3147.0	8.2
3149.6	17.3
3163.8	10.7
3166.9	11.8
3173.2	16.3
3186.6	5.8
3202.6	19.4
3826.3	25.9

Table 229: MP2 Frequencies and IR Intensities of cis-2,3-epoxy-1-ethyl-2-methylcyclopentan-1-ol (not H-bonded) with a TZ basis set

Frequency	IR Intensity
80.0	0.2
126.3	0.5
174.5	13.8
193.8	77.4
202.3	2.7
225.3	4.0
269.6	14.6
282.4	0.1
315.3	0.7
354.3	1.3
387.9	2.8
418.6	4.2
474.9	10.2
539.2	11.9
584.0	4.6
656.6	1.5
694.6	3.1
781.1	5.7
792.9	2.3
839.4	4.2
875.4	20.5
942.7	0.9
946.7	14.9
971.5	6.5
999.6	19.1
1022.2	4.3
1035.8	18.3
1043.0	10.6
1081.9	11.8
1100.6	14.1
1119.7	8.1
1159.2	20.6
1191.3	4.8
1226.8	46.1
1234.9	5.7
1249.2	18.8
1297.6	1.4
1317.4	1.3
1329.3	3.5
1339.9	7.0
1359.3	4.3
1397.9	7.4
1406.8	7.3
1417.4	5.1
1472.2	5.1
1483.7	7.1
1487.6	9.3
1500.1	6.2
1505.7	7.2
1514.8	3.3
1517.2	5.6
1527.8	7.0
3073.1	6.9
3076.3	21.2
3080.1	17.9
3083.0	26.9
3115.0	21.6
3130.6	9.5
3139.2	14.4
3160.7	21.2
3165.4	22.8
3169.0	10.7
3177.5	11.7
3180.1	11.5
3196.5	20.9
3837.8	25.8

Table 230: MP2 Frequencies and IR Intensities of cis-2,3-epoxy-1-ethyl-2-methylcyclopentan-1-ol (H-bonded) with the TZ basis set

Frequency	IR Intensity
125.6	1.7
132.0	1.1
196.2	0.4
218.4	0.3
234.8	0.5
250.9	2.8
255.7	0.6
300.0	0.8
367.0	4.8
395.9	4.7
430.8	34.1
450.4	17.6
476.6	53.2
526.0	9.9
608.5	4.8
644.7	0.6
675.7	5.9
784.1	0.8
796.0	3.1
832.1	3.5
873.6	16.3
939.0	1.7
942.8	26.1
968.2	9.3
1004.0	3.4
1016.3	17.5
1049.2	11.5
1065.6	37.4
1080.3	3.6
1112.3	1.9
1128.7	24.9
1155.6	21.1
1182.2	11.0
1195.5	17.4
1243.7	1.6
1272.5	2.8
1289.3	7.0
1322.8	0.9
1329.7	7.0
1340.0	2.6
1372.9	12.2
1405.8	11.2
1407.5	30.9
1418.1	4.8
1473.3	5.2
1486.0	8.7
1497.9	5.2
1499.4	8.2
1504.5	6.6
1515.7	1.0
1517.6	10.2
1526.3	4.9
3074.9	11.7
3076.2	12.0
3083.1	19.9
3090.4	27.4
3106.3	18.0
3130.1	5.1
3140.8	12.6
3159.9	24.1
3162.6	7.2
3163.1	34.1
3166.0	14.9
3178.3	8.4
3194.2	19.3
3791.6	26.8

Table 231: MP2 Frequencies and IR Intensities of trans-2,3-epoxy-1-ethyl-2-methylcyclopentan-1-ol with the TZ basis set

Frequency	IR Intensity
98.5	0.4
119.0	1.9
168.8	1.2
193.5	0.6
237.1	13.5
244.8	6.4
261.8	81.3
267.1	6.5
295.5	1.3
357.6	1.9
398.9	2.6
428.9	3.5
478.4	10.9
513.7	2.1
584.7	1.8
672.3	0.4
688.5	2.8
787.0	6.5
804.2	2.5
838.2	6.8
874.2	9.6
944.1	5.7
959.9	33.8
975.6	10.2
986.7	17.3
1016.2	17.2
1028.3	1.6
1044.8	1.5
1083.3	15.1
1097.7	18.2
1119.6	6.0
1162.5	2.0
1203.6	6.3
1211.6	51.9
1239.4	4.7
1247.8	11.3
1299.1	4.3
1319.7	2.3
1332.5	5.1
1345.7	2.4
1355.8	2.4
1402.6	15.7
1407.3	3.7
1422.5	14.8
1474.5	3.5
1483.0	4.0
1487.7	9.2
1493.7	4.2
1500.2	5.5
1516.0	2.2
1516.6	7.9
1524.9	7.0
3069.7	16.0
3076.6	7.7
3079.4	26.5
3091.1	26.9
3102.9	21.0
3128.0	3.9
3145.7	8.3
3152.7	27.0
3164.1	13.6
3164.7	16.8
3167.2	22.2
3185.4	5.8
3201.7	20.4
3832.0	23.0

Table 232: MP2 Frequencies and IR Intensities of cis-2-ethyl-2,3- epoxy-1-methylcyclopentan-1-ol (not H-bonded) with the TZ basis set

Frequency	IR Intensity
82.2	0.2
114.0	1.3
184.0	0.4
200.6	2.8
226.0	21.7
234.1	67.1
272.9	2.3
302.2	2.3
317.4	0.7
358.4	2.1
400.5	3.0
437.3	2.7
476.8	13.0
548.6	5.2
589.1	14.4
644.5	1.6
686.7	2.3
769.4	1.8
813.0	5.0
844.3	8.8
909.2	17.4
938.5	16.7
949.0	1.1
953.6	1.7
975.1	12.7
1013.2	15.3
1021.8	5.8
1052.3	1.7
1089.1	10.9
1105.5	23.0
1117.5	14.1
1160.3	27.6
1206.1	10.6
1218.2	42.8
1235.1	6.2
1239.2	14.1
1292.8	4.8
1311.8	0.8
1327.5	5.7
1335.8	7.8
1365.9	2.5
1399.8	16.5
1409.8	0.5
1413.6	6.6
1467.4	9.4
1486.1	1.2
1495.2	2.0
1498.9	15.0
1505.1	1.9
1509.5	2.8
1520.9	8.0
1524.6	6.9
3061.4	12.4
3079.4	21.0
3082.0	25.1
3088.0	11.7
3104.4	25.7
3138.3	12.2
3138.8	4.9
3145.5	28.6
3161.1	24.5
3171.7	12.4
3175.5	13.2
3178.9	16.3
3196.1	20.1
3827.7	26.6

Table 233: MP2 Frequencies and IR Intensities of cis-2-ethyl-2,3-epoxy-1-methylcyclopentan-1-ol (H-bonded) with the TZ basis set

Frequency	IR Intensity
93.6	0.1
100.6	2.8
170.1	0.6
199.0	0.4
225.6	1.6
270.4	0.3
296.1	1.6
309.6	0.9
354.8	2.0
394.5	7.5
430.2	27.2
444.5	36.4
477.0	43.3
550.9	6.8
594.7	10.4
648.3	3.4
682.4	3.6
770.3	2.1
812.0	2.2
838.6	8.5
914.9	10.8
931.8	13.3
950.4	1.7
959.3	7.1
972.7	19.2
1013.0	31.4
1024.8	2.1
1052.6	11.2
1090.3	4.7
1108.6	7.4
1122.9	9.8
1166.8	33.6
1179.7	6.0
1214.4	39.7
1237.9	7.2
1268.2	0.6
1296.4	9.6
1314.0	7.0
1331.9	1.7
1344.7	0.4
1368.8	4.8
1391.0	23.7
1413.4	6.3
1424.5	34.2
1471.8	8.1
1487.5	2.3
1493.5	2.8
1498.7	15.3
1502.1	0.1
1512.4	2.0
1521.1	6.4
1525.8	4.2
3074.8	8.3
3079.8	17.7
3082.3	20.8
3082.9	20.9
3109.7	16.6
3134.3	3.2
3138.8	17.2
3161.6	23.3
3167.5	14.3
3168.5	17.0
3175.0	20.9
3181.7	12.1
3195.9	19.3
3796.4	28.1

Table 234: MP2 Frequencies and IR Intensities of trans-2-ethyl-2,3-epoxy-1-methylcyclopentan-1-ol with the TZ basis set

Frequency	IR Intensity
48.9	0.0
123.3	1.8
175.3	1.4
217.8	0.9
228.8	3.8
263.7	9.9
268.4	20.6
280.2	56.7
304.9	4.1
371.4	7.7
391.7	3.8
427.6	1.6
471.8	14.0
542.1	5.7
589.6	2.6
642.7	2.7
695.4	0.3
775.3	2.2
812.4	4.5
852.4	6.2
887.8	2.2
939.3	25.7
943.9	4.0
958.7	23.7
986.9	2.7
1004.9	19.8
1029.1	8.9
1054.7	5.4
1089.7	15.8
1102.7	26.8
1121.1	3.1
1160.3	3.0
1206.1	7.9
1215.9	57.8
1235.6	1.8
1241.7	3.6
1289.3	4.5
1313.9	6.7
1333.7	5.4
1345.9	2.9
1361.8	1.1
1399.3	10.4
1411.8	1.2
1419.8	21.9
1468.8	4.9
1483.5	3.9
1492.0	4.9
1493.8	4.6
1505.5	4.9
1510.7	3.0
1514.1	6.6
1528.3	5.7
3063.1	14.8
3076.5	17.8
3080.5	29.4
3090.9	24.6
3103.6	24.9
3127.0	11.5
3146.2	9.6
3149.2	16.0
3159.0	29.6
3167.3	9.7
3173.8	17.0
3198.2	11.8
3199.4	14.0
3820.3	24.9

Table 235: MP2 Frequencies and IR Intensities of cis-2,3- thiirane-1,2-dimethylcyclopentan-1-ol (not H-bonded) with the TZ basis set

Frequency	IR Intensity
123.0	0.4
209.5	2.1
219.9	7.8
236.0	39.5
247.4	41.5
272.7	2.1
321.3	2.0
325.5	1.2
347.8	0.6
375.2	1.0
427.5	5.8
490.0	10.9
568.1	2.7
604.9	0.8
622.4	1.7
676.7	10.1
760.5	0.7
833.7	4.7
911.7	4.0
939.5	6.2
946.5	1.0
980.9	2.5
988.9	8.4
1015.9	7.4
1060.2	1.5
1074.2	17.2
1097.4	35.0
1138.8	16.0
1187.1	33.4
1203.8	13.2
1230.0	38.1
1237.5	5.7
1266.0	0.9
1322.1	10.4
1323.6	10.2
1372.3	0.3
1394.1	28.7
1401.7	2.9
1410.0	7.0
1483.6	4.6
1495.9	13.8
1497.1	7.0
1502.3	2.8
1509.6	5.0
1519.5	1.4
3062.5	11.5
3070.0	21.6
3072.1	27.2
3103.3	23.6
3140.3	14.6
3146.0	19.6
3155.1	10.8
3172.1	8.7
3175.6	7.4
3177.6	12.5
3196.4	7.2
3825.9	27.6

Table 236: MP2 Frequencies and IR Intensities of cis-2,3- thiirane-1,2-dimethylcyclopentan-1-ol (H-bonded) with the TZ basis set

Frequency	IR Intensity
119.8	3.0
211.4	0.9
229.2	0.1
233.4	0.2
264.9	0.6
314.1	0.6
319.3	0.6
342.1	1.4
371.4	2.4
419.6	21.0
435.4	62.0
497.7	20.8
568.9	5.0
599.6	0.1
623.9	4.2
674.4	7.9
754.6	1.1
830.6	2.3
912.8	0.2
944.8	2.2
953.8	3.6
979.4	14.4
991.0	4.0
1018.0	29.7
1058.6	7.6
1078.5	10.2
1102.0	5.5
1141.9	29.7
1169.1	9.4
1210.9	14.9
1232.1	41.4
1251.6	12.8
1269.8	0.7
1320.0	1.2
1336.6	6.8
1378.7	41.8
1392.1	15.5
1411.2	7.4
1414.8	23.5
1484.4	7.8
1491.0	4.5
1496.7	12.0
1501.6	5.4
1507.1	4.9
1521.5	0.5
3069.7	15.1
3072.9	18.7
3076.3	17.9
3110.2	13.1
3141.8	14.5
3154.9	8.5
3167.1	10.1
3168.1	16.6
3174.7	8.4
3181.5	11.9
3196.7	5.9
3775.5	19.0

Table 237: MP2 Frequencies and IR Intensities of trans-2,3- thiirane-1,2-dimethylcyclopentan-1-ol with the TZ basis set

Frequency	IR Intensity
121.3	1.1
213.0	1.0
221.3	1.9
239.0	4.1
252.4	0.3
262.6	79.3
289.4	9.5
324.1	2.3
333.6	2.8
385.6	5.2
407.9	2.8
458.5	2.1
578.2	6.3
610.1	0.3
627.1	3.1
679.5	21.3
774.0	6.3
828.6	2.5
901.6	7.2
931.8	7.5
943.8	19.6
978.5	5.1
995.1	0.7
1016.1	5.7
1062.3	19.0
1075.0	34.2
1092.4	8.1
1140.8	1.8
1180.9	31.1
1210.1	7.6
1227.7	0.9
1235.7	13.3
1259.4	11.2
1322.8	4.4
1340.7	4.5
1371.7	10.1
1397.2	7.9
1402.6	25.4
1408.8	3.5
1476.1	5.2
1491.4	6.3
1493.3	7.6
1502.1	3.6
1508.9	6.8
1511.1	3.1
3064.1	16.4
3076.0	17.5
3085.8	22.1
3101.7	23.6
3147.9	8.3
3149.7	17.4
3162.6	10.2
3166.1	10.5
3174.7	11.9
3178.1	6.7
3201.9	5.8
3820.7	26.5

Table 238: MP2 Frequencies and IR Intensities of cis-2,3-thiirane-1-ethyl-2-methylcyclopentan-1-ol (not H-bonded) with the TZ basis set

Frequency	IR Intensity
73.6	0.2
123.5	0.1
176.4	0.3
201.6	79.7
221.6	1.6
228.7	4.2
271.3	14.1
285.1	2.6
323.3	1.3
341.9	2.3
345.0	0.4
366.6	0.8
423.6	7.2
514.8	6.8
567.0	2.6
613.7	2.4
641.3	0.3
677.1	12.0
741.0	1.2
794.6	1.6
834.6	4.4
911.7	3.4
937.4	4.0
959.4	6.6
989.3	9.5
1009.9	16.2
1019.4	4.7
1039.2	16.1
1068.4	6.6
1075.2	8.1
1107.0	12.9
1138.8	14.7
1183.0	11.2
1196.0	20.8
1224.3	37.0
1232.5	6.9
1267.9	1.1
1308.1	7.0
1319.5	9.5
1325.8	0.3
1355.7	5.8
1374.2	0.2
1396.5	21.7
1407.8	2.8
1419.0	8.4
1479.7	2.0
1486.1	13.9
1499.1	7.4
1505.0	3.0
1508.7	6.6
1516.1	4.7
1527.1	8.5
3069.3	7.0
3070.2	23.2
3073.5	17.9
3076.1	31.7
3116.1	18.5
3134.9	8.7
3139.8	11.7
3156.1	21.4
3157.7	7.0
3167.8	23.9
3174.7	8.3
3176.4	15.6
3195.5	7.5
3833.9	26.4

Table 239: MP2 Frequencies and IR Intensities of cis-2,3-thiirane-1-ethyl-2-methylcyclopentan-1-ol (H-bonded) with the TZ basis set

Frequency	IR Intensity
126.4	1.8
148.1	0.4
210.5	0.4
231.6	0.6
236.6	1.0
251.0	0.2
271.9	0.1
311.7	1.1
333.4	0.6
347.2	0.3
408.7	10.6
429.2	32.5
458.8	40.5
492.5	20.8
574.9	5.6
600.9	0.2
628.3	2.7
674.8	9.7
744.1	0.1
794.3	2.9
833.2	1.8
913.2	1.1
933.2	16.0
960.5	4.9
987.1	0.9
1004.7	10.9
1037.3	15.6
1059.5	27.1
1071.9	16.6
1080.8	7.4
1113.3	8.9
1139.5	13.4
1173.2	14.1
1191.7	21.6
1223.8	9.5
1242.8	2.8
1257.5	5.7
1312.8	1.6
1318.8	0.3
1325.8	1.0
1363.6	29.3
1392.6	13.6
1395.2	37.0
1411.4	5.1
1416.6	4.0
1483.4	4.5
1491.9	12.8
1497.4	9.4
1503.5	5.6
1511.2	1.9
1517.1	9.4
1527.8	3.6
3069.8	16.1
3074.6	11.7
3085.4	15.5
3089.5	29.1
3105.9	15.4
3132.9	4.0
3142.5	11.8
3154.3	14.9
3159.3	21.4
3162.4	20.8
3166.6	19.9
3173.3	6.6
3193.1	5.6
3764.0	19.4

Table 240: MP2 Frequencies and IR Intensities of trans-2,3-thiirane-1-ethyl-2-methylcyclopentan-1-ol with the TZ basis set

Frequency	IR Intensity
92.8	0.1
116.3	1.0
169.5	0.9
213.9	0.2
235.5	7.6
241.2	20.1
267.4	68.2
283.6	6.3
291.4	4.2
324.7	0.9
342.1	2.8
408.3	4.0
414.2	1.7
478.2	1.6
571.4	5.2
611.6	1.1
646.2	3.0
678.4	19.6
768.1	8.2
793.3	0.5
839.6	1.9
910.6	4.0
948.5	21.7
969.1	11.3
983.0	18.1
993.1	19.2
1016.4	5.9
1032.6	0.6
1067.2	6.8
1078.2	17.1
1104.6	2.9
1143.8	1.5
1181.1	28.0
1201.4	3.6
1225.3	1.8
1232.4	17.0
1255.7	7.9
1314.7	5.2
1316.8	3.0
1339.5	3.4
1346.1	5.2
1377.8	9.8
1398.7	17.5
1407.7	2.4
1418.8	3.5
1476.8	4.7
1486.3	6.4
1491.5	4.6
1494.9	4.4
1509.9	6.8
1516.3	6.9
1525.8	6.4
3069.8	16.6
3075.6	10.2
3078.8	23.1
3084.7	23.9
3101.6	20.8
3129.6	2.6
3146.5	9.1
3154.3	25.5
3162.6	11.6
3163.4	14.1
3165.9	21.4
3176.9	5.7
3201.0	6.2
3825.6	23.8

Table 241: MP2 Frequencies and IR Intensities of cis-2-ethyl-2,3-thiirane-1-methylcyclopentan-1-ol (not H-bonded) with the TZ basis set

Frequency	IR Intensity
83.9	0.2
109.0	0.5
185.5	1.0
205.9	0.6
228.8	5.3
245.8	78.0
275.3	5.1
290.2	2.6
329.9	1.8
346.8	1.1
360.5	1.4
379.8	1.0
439.0	4.5
497.0	9.9
563.5	2.4
614.3	2.7
630.1	0.7
710.2	10.0
748.4	1.2
802.3	4.1
835.9	7.9
909.6	9.5
943.8	2.0
948.1	0.8
966.0	6.8
994.4	5.2
1017.4	3.0
1025.6	5.1
1068.8	2.8
1085.9	11.7
1104.9	30.8
1139.0	17.3
1184.1	34.6
1203.0	13.6
1211.5	8.0
1228.9	26.8
1260.8	1.8
1314.7	2.9
1318.3	5.7
1322.2	13.4
1353.3	3.9
1376.4	0.5
1394.4	26.3
1399.9	0.8
1410.7	9.1
1483.2	4.9
1492.9	2.5
1497.9	14.0
1504.5	1.7
1509.4	5.6
1520.0	6.8
1523.4	5.6
3061.0	13.9
3069.8	26.4
3079.9	22.8
3087.1	11.0
3103.2	23.9
3139.0	14.5
3140.7	1.0
3144.9	28.2
3158.1	22.2
3172.9	8.4
3176.9	13.3
3185.4	16.2
3201.1	7.3
3824.1	26.8

Table 242: MP2 Frequencies and IR Intensities of cis-2-ethyl-2,3-thiiranene-1-methylcyclopentan-1-ol (H-bonded) with the TZ basis set

Frequency	IR Intensity
96.8	1.8
108.1	0.9
184.1	0.8
205.0	0.0
226.2	1.2
273.9	0.1
286.4	0.6
322.1	0.1
342.7	8.4
360.7	1.0
373.5	6.8
392.9	78.0
435.3	4.0
500.1	13.1
565.6	3.6
614.9	1.9
633.1	1.8
710.2	9.3
752.5	0.6
804.0	2.6
831.9	5.3
911.2	2.1
943.3	2.8
954.2	1.0
970.2	6.6
995.4	15.6
1020.9	18.9
1026.9	13.8
1069.3	9.4
1089.1	5.5
1101.5	9.0
1145.6	22.8
1164.5	8.0
1206.1	4.3
1224.3	24.7
1241.0	43.8
1250.7	1.1
1314.9	7.9
1317.5	4.2
1338.7	2.4
1352.5	1.3
1373.0	48.6
1391.5	5.5
1410.0	18.5
1412.3	8.5
1484.1	6.1
1490.5	1.8
1496.7	17.0
1501.2	0.0
1512.5	4.4
1520.5	5.7
1525.5	2.8
3069.8	10.3
3071.1	19.7
3074.5	24.4
3082.4	11.9
3110.1	13.1
3137.8	2.4
3142.9	16.2
3153.3	21.7
3164.0	15.6
3166.3	15.0
3172.2	17.5
3180.4	12.2
3203.5	6.3
3809.1	20.7

Table 243: MP2 Frequencies and IR Intensities of trans-2-ethyl-2,3-thiirane-1-methylcyclopentan-1-ol with the TZ basis set

Frequency	IR Intensity
53.1	0.1
115.3	0.9
185.7	0.8
215.0	0.1
225.8	2.4
254.9	0.4
266.5	0.5
278.1	73.8
296.7	14.5
326.4	1.6
363.0	5.4
401.5	6.2
404.8	1.9
473.0	3.9
572.2	5.9
615.9	0.9
630.6	1.6
709.2	17.1
764.4	3.4
806.5	16.5
832.0	0.7
898.2	11.6
936.1	2.3
949.9	19.6
969.2	1.7
994.8	10.1
1014.3	1.6
1034.1	4.0
1069.2	0.4
1092.5	35.2
1098.3	12.8
1141.2	1.8
1179.3	26.6
1207.2	6.4
1210.9	1.4
1231.2	11.9
1253.9	9.9
1316.0	10.0
1319.7	4.2
1339.2	2.8
1352.6	1.0
1380.1	3.9
1395.8	1.0
1403.1	19.0
1407.8	18.7
1475.5	4.7
1487.9	5.8
1491.3	5.3
1502.5	4.0
1507.5	2.5
1509.7	7.6
1526.8	4.2
3063.2	16.3
3078.4	27.6
3081.1	17.1
3083.1	22.1
3102.0	23.1
3132.0	8.4
3146.6	10.5
3148.6	16.0
3154.5	29.7
3166.6	9.8
3176.3	12.8
3201.7	4.7
3206.4	6.7
3814.2	24.9

Table 244: MP2 Frequencies and IR Intensities of cis-2,3-phosphirane-1,2-dimethylcyclopentan-1-ol (not H-bonded) with the TZ basis set

Frequency	IR Intensity
114.4	3.1
153.0	88.9
203.7	1.7
225.5	1.2
235.7	2.2
278.4	1.3
301.5	0.7
317.6	1.4
346.1	0.0
372.3	0.8
421.9	2.3
472.2	8.5
556.2	1.4
587.7	0.2
600.6	0.9
653.2	0.7
719.1	1.3
754.8	1.7
831.5	3.8
887.7	8.3
916.9	11.1
941.7	8.2
955.9	0.4
974.5	0.1
989.1	7.9
1011.9	7.6
1056.6	5.1
1081.6	23.3
1098.5	21.8
1135.8	22.1
1168.8	29.7
1199.7	9.8
1225.3	36.4
1233.6	10.3
1258.7	2.9
1314.4	24.8
1321.2	2.8
1359.9	2.7
1387.2	23.0
1397.1	7.7
1406.9	4.8
1490.2	2.8
1497.6	4.4
1501.1	11.5
1507.2	10.8
1510.6	1.3
1521.7	1.4
2441.4	73.2
3055.3	13.6
3057.0	31.0
3070.6	33.1
3088.0	13.0
3132.9	17.0
3136.0	14.1
3140.9	20.9
3147.7	24.4
3160.8	11.6
3168.7	12.2
3207.1	5.3
3856.5	26.4

Table 245: MP2 Frequencies and IR Intensities of cis-2,3-phosphirane-1,2-dimethylcyclopentan-1-ol (H-bonded) with a TZ basis set

Frequency	IR Intensity
118.9	2.9
202.3	1.7
224.8	0.0
233.6	0.2
263.3	6.3
300.8	0.8
311.6	8.2
318.0	61.0
344.0	7.6
372.6	3.3
421.5	2.1
470.4	11.1
558.6	1.8
586.8	0.2
601.7	5.2
647.1	1.2
717.0	1.4
749.4	1.1
827.5	0.4
879.2	8.3
921.9	1.4
946.5	2.7
960.4	3.7
974.9	0.5
989.5	8.6
1010.8	45.3
1054.9	3.4
1086.9	8.1
1094.4	8.0
1136.0	32.4
1163.3	6.2
1204.4	6.8
1229.6	56.4
1240.5	14.9
1255.3	2.2
1317.7	3.1
1329.3	8.3
1370.2	47.0
1377.8	9.1
1407.7	10.2
1408.9	17.7
1489.7	5.7
1491.6	2.7
1501.5	12.4
1506.5	10.8
1508.3	1.2
1523.6	0.9
2453.5	62.6
3057.3	24.5
3070.3	9.6
3074.0	31.3
3085.0	10.7
3137.0	19.1
3137.7	11.8
3152.9	19.0
3159.9	11.4
3163.0	17.1
3174.7	12.4
3205.9	4.8
3820.0	17.1

Table 246: MP2 Frequencies and IR Intensities of trans-2,3-phosphirane-1,2-dimethylcyclopentan-1-ol with the TZ basis set

Frequency	IR Intensity
117.4	7.8
139.5	88.2
208.1	1.6
213.3	0.2
238.3	1.0
259.6	0.3
288.5	1.8
312.0	0.8
320.0	2.7
379.6	4.3
403.4	2.3
445.1	2.3
557.7	1.8
603.1	4.2
604.9	0.5
652.4	9.6
722.1	1.4
772.0	2.7
818.3	7.5
885.8	7.9
905.5	6.3
944.1	0.6
952.4	24.6
976.0	11.4
989.0	2.0
1014.1	4.6
1060.1	3.6
1073.4	56.9
1093.4	0.5
1136.7	1.9
1167.7	28.9
1205.3	4.8
1219.9	0.8
1234.7	13.4
1252.5	23.3
1314.0	4.4
1335.0	2.3
1358.3	10.0
1385.6	9.7
1402.2	20.1
1406.1	7.6
1482.6	4.8
1494.1	7.0
1498.2	4.1
1502.1	3.6
1508.4	2.6
1514.1	8.4
2446.0	67.0
3055.7	18.3
3064.0	25.7
3083.4	16.5
3086.1	26.4
3136.9	10.3
3141.0	25.2
3147.9	13.9
3148.7	18.2
3160.7	8.1
3165.9	13.9
3211.1	4.4
3855.5	25.5

Table 247: MP2 Frequencies and IR Intensities of cis-2,3-phosphirane-1-ethyl-2-methylcyclopentan-1-ol (not H-bonded) with the TZ basis set

Frequency	IR Intensity
78.6	1.4
108.5	91.4
125.0	5.1
170.5	0.4
222.1	1.5
227.2	0.3
266.8	3.5
282.1	2.7
306.2	0.9
331.7	2.4
340.4	0.8
361.0	0.3
419.7	2.5
497.3	6.0
554.4	1.6
584.6	0.5
630.5	1.1
659.1	0.8
718.9	1.7
733.5	0.6
796.3	1.3
830.1	4.3
889.1	7.9
918.7	12.3
940.3	5.4
960.5	4.7
982.6	4.9
1006.6	18.7
1017.6	2.6
1044.0	19.6
1063.4	5.6
1084.4	7.1
1107.2	11.6
1135.2	20.4
1168.4	14.7
1189.6	9.0
1220.4	41.9
1228.9	3.9
1262.6	2.9
1298.6	21.5
1310.7	4.1
1326.0	0.2
1350.8	8.2
1364.8	0.9
1389.6	19.4
1406.3	5.1
1416.3	3.5
1480.5	2.8
1493.8	10.4
1504.3	7.0
1507.6	2.1
1513.2	8.2
1516.9	5.1
1527.9	9.0
2442.3	74.5
3057.1	25.5
3066.4	10.7
3068.0	25.9
3072.1	33.0
3094.4	9.9
3129.2	8.6
3133.9	16.0
3138.8	13.3
3149.0	15.5
3154.2	29.2
3160.7	15.0
3163.1	25.6
3206.3	5.7
3863.6	25.3

Table 248: MP2 Frequencies and IR Intensities of cis-2,3-phosphirane-1-ethyl-2-methylcyclopentan-1-ol (H-bonded) with the TZ basis set

Frequency	IR Intensity
121.6	1.7
145.6	0.2
217.4	0.2
221.1	1.0
229.8	0.2
248.5	0.5
285.4	0.1
307.2	7.0
311.9	57.6
326.8	16.3
345.7	1.3
409.4	4.1
436.8	7.0
465.0	5.5
564.2	1.4
590.8	0.4
597.2	3.7
653.8	1.3
715.4	1.4
736.2	2.1
795.7	2.1
828.3	0.6
878.4	8.8
923.2	4.9
934.9	8.9
960.4	3.0
978.4	10.6
1005.0	8.1
1035.0	20.9
1058.9	10.2
1075.6	31.9
1083.5	9.4
1105.3	9.2
1136.9	18.6
1166.2	9.7
1189.3	16.7
1216.8	17.1
1233.9	6.1
1247.2	5.4
1306.8	2.1
1315.3	0.8
1319.7	3.0
1359.3	41.6
1380.4	17.9
1382.9	11.5
1407.0	9.8
1416.5	3.8
1486.7	3.1
1495.3	12.2
1500.6	9.3
1505.5	5.5
1515.7	5.6
1517.2	8.0
1530.7	3.1
2453.7	61.2
3057.6	24.7
3064.8	13.1
3082.9	16.0
3086.1	13.5
3091.5	32.1
3127.9	1.7
3137.1	17.1
3137.2	11.1
3148.2	19.2
3150.3	40.7
3158.2	16.0
3159.2	13.9
3202.9	4.2
3816.9	16.7

Table 249: MP2 Frequencies and IR Intensities of trans-2,3-phosphirane-1-ethyl-2-methylcyclopentan-1-ol with the TZ basis set

Frequency	IR Intensity
91.9	3.7
114.6	17.9
134.3	80.5
170.0	3.2
211.5	0.2
227.4	0.5
254.0	2.6
282.6	2.3
291.1	1.2
312.4	0.4
333.8	3.7
401.3	2.2
409.4	1.9
458.4	1.3
551.7	2.3
597.4	3.7
635.0	0.8
649.9	8.4
722.3	1.6
766.8	3.4
793.2	1.9
831.6	6.4
883.7	6.7
922.3	6.7
948.8	6.3
975.0	11.1
978.0	11.6
989.9	46.9
1014.4	3.0
1032.4	1.9
1063.6	2.4
1085.8	21.7
1102.5	1.2
1138.9	1.4
1168.4	25.0
1197.0	2.6
1219.0	1.6
1231.9	19.1
1247.4	15.3
1302.7	8.2
1314.8	2.2
1334.7	2.6
1341.2	5.4
1365.4	5.3
1392.1	15.5
1404.7	6.9
1415.5	3.1
1483.3	3.7
1486.3	5.5
1495.8	4.2
1498.1	4.1
1513.5	8.5
1517.1	6.6
1527.1	5.7
2445.4	68.4
3061.0	18.3
3064.1	22.4
3074.2	19.3
3080.8	12.8
3084.9	28.2
3122.3	2.0
3136.3	16.1
3145.9	32.2
3147.3	15.1
3148.8	16.9
3156.6	22.6
3159.9	9.2
3210.4	4.7
3861.2	23.0

Table 250: MP2 Frequencies and IR Intensities of cis-2-ethyl-2,3-phosphirane-1-methylcyclopentan-1-ol (not H-bonded) with the TZ basis set

Frequency	IR Intensity
88.8	1.2
111.9	0.5
157.7	87.0
193.5	3.0
204.6	0.7
222.6	1.9
280.0	2.3
286.1	1.1
307.5	0.4
347.4	0.3
355.7	2.8
374.0	0.3
438.0	1.4
481.0	7.0
552.8	1.7
590.4	0.4
615.8	0.7
687.0	1.0
718.2	1.1
746.0	3.9
796.1	2.7
832.9	4.7
896.0	12.1
913.3	17.0
946.8	0.8
958.6	0.4
963.0	3.6
992.8	1.0
1011.2	8.7
1022.4	1.3
1067.4	2.3
1084.2	15.1
1102.3	24.8
1135.3	25.3
1166.3	27.9
1199.3	1.8
1202.6	23.0
1229.4	28.6
1252.5	5.0
1309.5	17.0
1313.6	5.0
1318.3	7.7
1350.4	2.7
1363.6	6.3
1381.5	18.4
1398.3	9.7
1409.7	7.3
1488.7	2.0
1493.7	4.8
1502.2	11.3
1507.3	5.2
1511.4	3.5
1518.6	6.8
1522.7	4.2
2439.2	72.5
3055.3	16.4
3068.8	32.7
3071.7	24.9
3081.5	14.1
3088.6	13.1
3129.5	4.1
3132.5	17.6
3139.6	29.4
3147.9	23.1
3150.1	25.4
3169.2	12.2
3173.7	18.7
3212.0	5.0
3856.6	25.7

Table 251: MP2 Frequencies and IR Intensities of cis-2-ethyl-2,3-phosphirane-1-methylcyclopentan-1-ol (H-bonded) with TZ basis

Frequency	IR Intensity
95.2	1.1
113.1	1.2
192.4	1.0
203.1	0.3
219.2	0.7
273.7	3.3
284.4	0.7
304.2	2.4
323.5	49.5
345.5	29.3
352.6	0.3
375.2	3.1
436.1	1.8
478.5	9.3
556.1	2.0
590.5	0.7
616.2	4.6
681.3	1.5
715.5	1.4
743.1	1.8
795.6	3.0
828.7	0.4
887.6	10.9
919.7	3.8
950.5	0.4
957.9	0.8
968.5	4.7
992.6	4.7
1009.0	40.5
1023.5	6.9
1066.0	3.3
1088.6	2.3
1097.7	13.5
1139.8	25.2
1161.8	7.6
1199.3	1.4
1217.9	30.4
1231.0	43.6
1243.7	5.6
1311.2	2.5
1316.2	14.0
1332.9	1.0
1351.4	3.2
1370.0	30.9
1373.5	22.3
1408.0	23.4
1410.0	7.6
1488.3	2.9
1491.8	5.1
1498.2	13.0
1506.3	3.7
1513.3	4.5
1517.6	5.1
1524.4	2.8
2454.3	61.5
3070.5	10.3
3072.3	15.3
3073.0	38.3
3079.1	13.9
3086.9	10.0
3127.8	5.3
3136.5	19.9
3151.0	23.2
3152.8	19.3
3164.0	24.1
3172.7	14.1
3177.1	12.3
3209.7	4.6
3818.0	17.4

Table 252: MP2 Frequencies and IR Intensities of trans-2-ethyl-2,3-phosphirane-1-methylcyclopentan-1-ol with the TZ basis set

Frequency	IR Intensity
78.2	0.3
105.6	1.9
160.8	85.6
198.5	5.1
209.9	1.0
214.2	1.1
258.4	0.3
280.4	0.4
287.9	2.6
305.6	0.2
353.6	4.3
391.1	2.5
407.2	2.9
466.1	4.0
554.1	1.9
603.8	3.1
612.7	0.4
684.0	7.3
720.7	2.3
753.1	1.9
808.0	12.9
817.5	4.6
895.5	9.0
900.7	9.7
948.5	3.6
961.4	15.6
963.5	4.2
997.3	6.8
1009.1	4.3
1030.1	1.9
1066.4	1.2
1084.5	36.0
1099.9	11.9
1135.9	2.9
1164.2	23.2
1200.1	4.6
1203.0	3.0
1230.5	10.0
1247.2	27.7
1309.7	5.6
1316.5	8.5
1333.5	1.6
1352.0	1.6
1361.8	6.3
1380.4	9.1
1403.7	4.9
1405.9	26.6
1481.3	5.4
1485.8	4.4
1497.5	4.4
1501.8	4.9
1506.3	1.7
1510.0	7.9
1525.6	3.0
2447.2	65.0
3055.2	18.4
3070.4	31.2
3079.7	19.6
3082.1	29.1
3084.0	13.6
3125.8	10.1
3137.2	9.5
3140.3	24.8
3147.4	29.5
3147.6	21.8
3167.7	13.2
3188.0	7.5
3215.3	4.0
3851.0	23.6

Table 253: MP2 Frequencies and IR Intensities of cis-2,3-aziridine-1,2-dimethylcyclopentan-1-ol (not H-bonded) with the TZ basis set

Frequency	IR Intensity
80.7	93.4
133.5	7.9
214.2	3.3
217.8	0.1
241.2	0.8
277.6	0.5
320.7	0.6
360.1	1.6
374.6	0.9
417.9	4.9
466.5	8.9
512.8	15.7
580.2	3.9
648.9	1.5
662.4	2.7
805.7	1.7
844.1	1.3
861.2	29.6
926.1	6.8
942.9	12.2
950.9	12.4
969.9	8.5
1000.5	19.1
1013.7	14.1
1042.4	16.0
1085.9	31.3
1091.5	1.1
1120.6	38.9
1146.8	21.5
1159.6	13.1
1206.8	17.9
1236.9	26.3
1239.5	10.0
1290.3	15.2
1311.5	12.3
1331.7	1.4
1346.6	7.4
1397.8	22.8
1407.3	3.2
1412.3	6.4
1466.5	5.2
1484.3	4.8
1497.9	9.4
1500.1	8.2
1504.6	0.9
1513.2	6.3
1525.8	1.6
3056.2	13.7
3061.4	21.9
3080.2	29.6
3099.4	29.7
3135.3	18.6
3140.7	23.3
3148.1	13.9
3152.0	14.8
3164.6	15.1
3171.5	11.1
3200.1	16.4
3544.8	1.8
3859.2	25.8

Table 254: MP2 Frequencies and IR Intensities of cis-2,3-aziridine-1,2-dimethylcyclopentan-1-ol (H-bonded) with the TZ basis set

Frequency	IR Intensity
106.1	2.7
217.9	0.4
226.2	0.3
230.6	1.0
274.3	0.9
313.1	1.2
348.0	7.2
368.2	2.4
417.3	17.2
428.7	55.4
466.7	26.0
517.4	15.3
588.8	3.7
653.7	7.8
658.7	1.6
804.7	0.1
836.0	0.7
867.5	19.0
926.8	12.5
940.5	12.6
958.8	9.6
975.4	2.6
1001.1	30.4
1019.1	29.2
1041.4	20.6
1089.2	7.1
1103.4	10.4
1128.9	15.9
1129.9	13.8
1177.3	3.4
1196.3	17.6
1223.3	69.1
1250.0	1.3
1301.2	9.4
1331.9	2.6
1344.8	0.9
1351.4	5.0
1387.7	34.1
1410.7	10.0
1422.2	33.4
1471.1	4.2
1486.3	7.9
1495.2	4.1
1499.2	9.6
1504.9	1.8
1512.5	5.8
1526.8	1.4
3058.8	20.7
3066.5	11.8
3080.4	30.5
3106.6	18.1
3134.8	19.0
3144.5	12.6
3151.8	13.3
3158.9	16.8
3164.5	16.4
3169.2	16.6
3200.1	16.5
3541.6	2.1
3814.0	26.7

Table 255: MP2 Frequencies and IR Intensities of trans-2,3-aziridine-1,2-dimethylcyclopentan-1-ol with the TZ basis set

Frequency	IR Intensity
120.2	4.5
160.4	95.0
215.2	1.7
227.9	3.9
245.1	0.8
268.7	0.8
284.9	2.5
346.3	0.2
377.5	5.5
422.2	0.9
460.6	16.9
492.2	1.2
594.8	4.2
642.6	0.1
675.1	0.4
807.7	3.0
843.0	4.1
861.9	17.8
920.4	7.4
941.5	26.0
955.6	6.9
964.2	23.6
1003.3	20.7
1017.4	24.2
1038.7	18.4
1087.0	27.6
1093.6	5.4
1124.6	4.7
1135.1	6.4
1164.5	5.9
1208.4	6.1
1232.7	74.7
1240.4	4.3
1290.2	13.2
1313.5	6.0
1342.3	1.2
1350.6	2.5
1397.5	12.4
1405.9	11.1
1415.1	11.2
1470.0	3.6
1478.6	5.6
1494.5	7.6
1496.3	4.9
1504.4	3.0
1513.8	3.8
1522.4	4.2
3054.6	19.2
3059.0	26.0
3088.8	29.7
3099.1	29.8
3136.7	22.9
3140.1	15.2
3143.2	11.9
3162.3	3.4
3166.6	22.9
3170.2	7.2
3204.5	15.8
3541.6	2.0
3861.9	26.6

Table 256: MP2 Frequencies and IR Intensities of cis-2,3-aziridine-1-ethyl-2-methylcyclopentan-1-ol (not H-bonded) with the TZ basis set

Frequency	IR Intensity
−160.6	111.6
85.9	0.7
130.9	0.3
182.8	0.4
216.7	1.1
226.3	1.0
269.3	3.0
290.9	0.7
324.2	0.5
355.4	0.5
380.8	1.6
420.9	8.0
471.1	3.7
526.7	14.5
581.8	2.5
643.9	2.2
687.9	2.1
788.6	1.2
797.4	2.1
846.2	0.9
861.0	29.4
927.4	12.5
949.1	1.9
967.2	39.2
975.0	16.5
1008.7	15.5
1021.3	2.3
1042.6	17.7
1050.2	25.0
1093.0	2.8
1100.2	7.1
1119.0	36.6
1145.1	14.4
1157.2	4.2
1191.0	7.8
1229.0	28.1
1249.6	11.1
1273.1	22.9
1302.0	0.3
1321.6	2.8
1336.1	0.9
1349.4	6.0
1363.4	7.7
1398.6	9.0
1410.7	5.4
1416.1	4.8
1467.2	5.3
1482.5	3.2
1485.7	10.9
1500.2	6.8
1504.3	2.4
1518.7	5.6
1520.7	6.3
1527.1	6.3
3061.4	17.0
3066.0	12.6
3068.8	28.1
3080.6	32.5
3111.0	23.4
3123.6	8.5
3135.7	17.9
3147.0	14.1
3152.5	26.6
3155.2	2.7
3158.7	37.1
3172.7	13.7
3199.8	16.9
3545.6	1.7
3870.0	27.1

Table 257: MP2 Frequencies and IR Intensities of cis-2,3-aziridine-1-ethyl-2-methylcyclopentan-1-ol (H-bonded) with the TZ basis set

Frequency	IR Intensity
78.7	1.5
123.7	1.1
180.7	1.0
220.9	0.6
230.2	0.1
243.4	1.6
284.4	2.5
316.6	1.4
348.2	3.8
377.8	5.6
417.7	71.4
422.4	10.0
469.1	11.1
529.5	16.4
590.1	2.5
647.9	7.7
686.0	1.4
791.6	0.4
799.9	0.4
838.4	1.2
867.1	17.0
927.6	17.3
948.7	5.4
969.4	57.8
986.2	1.9
1014.8	13.9
1031.1	9.9
1040.9	15.0
1050.0	17.4
1095.9	6.6
1103.2	11.3
1128.0	5.4
1130.5	19.7
1168.5	7.6
1189.6	6.3
1214.0	59.6
1247.7	1.4
1291.2	5.3
1320.5	1.9
1332.5	6.2
1338.7	1.8
1352.1	3.3
1362.3	18.9
1410.3	7.2
1412.1	18.3
1416.8	21.2
1471.1	4.6
1483.2	7.3
1487.1	6.8
1500.0	5.7
1503.5	2.2
1518.0	7.8
1520.5	6.3
1524.6	5.5
3058.7	16.8
3062.4	21.9
3076.3	25.0
3079.8	30.6
3115.0	13.8
3119.1	11.7
3135.3	19.8
3144.0	14.0
3153.3	8.6
3157.5	38.8
3171.7	4.6
3175.8	23.3
3199.7	16.9
3541.7	2.0
3815.6	29.0

Table 258: MP2 Frequencies and IR Intensities of trans-2,3-aziridine-1-ethyl-2-methylcyclopentan-1-ol with the TZ basis set

Frequency	IR Intensity
99.6	1.4
122.3	23.2
145.4	79.8
172.6	3.9
215.9	0.5
246.2	1.7
256.2	1.4
276.7	5.2
294.8	1.5
357.3	0.5
400.5	1.4
428.2	3.1
473.1	14.2
497.9	2.5
581.3	3.2
662.9	0.6
683.3	0.3
791.6	3.7
819.8	1.8
839.3	5.1
863.7	17.7
928.1	13.9
961.0	0.7
964.5	37.3
986.5	23.4
995.9	44.8
1017.7	16.5
1037.5	0.5
1042.3	8.7
1092.3	1.5
1103.3	8.2
1123.5	5.5
1133.8	6.6
1167.6	4.2
1201.5	4.9
1223.6	63.6
1242.0	5.7
1274.0	22.1
1310.7	6.5
1322.9	0.5
1337.8	0.8
1350.3	2.2
1360.7	3.6
1404.6	15.6
1409.5	1.8
1421.7	8.1
1471.4	2.6
1479.1	4.6
1489.0	7.3
1495.7	2.9
1497.7	3.8
1517.6	6.0
1521.1	6.2
1525.5	5.2
3059.1	23.2
3059.8	17.7
3071.0	23.2
3088.0	32.2
3097.5	26.5
3121.3	1.5
3137.4	22.3
3141.7	10.6
3143.8	26.3
3156.6	25.3
3160.7	18.3
3169.6	6.2
3204.0	16.5
3541.5	1.9
3868.2	23.5

Table 259: MP2 Frequencies and IR Intensities of cis-2-ethyl-2,3-aziridine-1-methylcyclopentan-1-ol (not H-bonded) with the TZ basis set

Frequency	IR Intensity
79.6	64.3
94.2	20.8
118.5	13.1
190.8	2.3
213.4	0.6
233.6	0.8
278.9	2.3
301.9	0.6
336.7	0.6
361.9	1.3
405.6	4.1
435.5	3.1
470.0	12.2
536.3	5.5
587.8	8.0
640.2	1.5
670.3	4.1
768.9	2.5
811.0	3.8
856.4	4.6
873.7	25.5
931.7	40.3
939.0	3.4
961.7	7.1
978.6	2.4
1002.9	3.0
1013.9	13.6
1018.1	11.7
1068.9	12.9
1090.2	16.6
1105.1	8.4
1113.4	6.4
1139.5	67.3
1156.1	6.3
1207.1	17.1
1234.8	23.5
1236.9	12.1
1259.9	8.1
1307.3	9.8
1321.3	4.8
1328.6	3.7
1344.5	8.7
1363.4	2.8
1399.3	19.5
1410.5	3.1
1416.3	9.5
1459.5	3.6
1483.2	3.9
1492.4	4.5
1499.4	11.7
1505.5	1.0
1510.5	4.2
1520.1	7.8
1524.6	4.3
3054.2	13.5
3068.4	19.4
3071.6	24.9
3079.2	29.6
3099.4	29.5
3114.0	7.8
3134.6	18.8
3138.8	32.3
3151.6	26.3
3160.8	15.6
3170.8	16.6
3172.2	11.1
3200.0	16.0
3546.4	1.7
3858.6	25.6

Table 260: MP2 Frequencies and IR Intensities of cis-2-ethyl-2,3-aziridine-1-methylcyclopentan-1-ol (H-bonded) with the TZ basis set

Frequency	IR Intensity
96.0	1.8
105.0	0.5
173.2	0.6
212.5	0.5
232.6	1.2
275.2	0.3
296.4	2.5
332.1	1.2
356.5	2.9
399.0	6.5
435.1	5.4
439.9	52.7
472.2	44.5
538.4	5.9
595.5	5.4
644.9	7.1
668.8	4.8
772.0	2.6
811.9	1.1
851.4	2.4
881.9	14.0
928.2	36.0
940.4	4.0
966.4	13.6
980.7	3.3
1007.1	3.3
1014.6	24.5
1024.3	27.2
1068.8	16.5
1096.5	5.7
1104.6	15.4
1118.4	1.6
1133.9	15.2
1172.8	7.0
1195.3	18.5
1223.1	71.5
1246.3	1.0
1273.0	0.2
1311.7	11.7
1328.4	5.9
1341.4	1.4
1356.0	9.4
1365.6	3.7
1393.1	25.3
1414.2	7.4
1422.5	38.6
1465.2	2.9
1485.6	4.5
1490.5	4.6
1497.4	12.5
1503.2	1.1
1512.9	3.2
1520.6	5.3
1526.5	2.7
3060.7	21.5
3065.9	10.4
3072.8	24.6
3079.2	30.3
3107.1	11.4
3107.9	17.3
3134.2	20.3
3152.4	25.2
3160.3	17.5
3163.0	14.8
3166.8	23.2
3175.9	12.1
3197.0	16.2
3544.1	2.1
3810.3	27.6

Table 261: MP2 Frequencies and IR Intensities of trans-2-ethyl-2,3-aziridine-1-methylcyclopentan-1-ol with the TZ basis set

Frequency	IR Intensity
58.8	0.2
121.2	2.8
171.3	34.8
193.8	61.3
228.3	0.8
229.8	3.8
268.6	0.1
278.7	0.5
302.9	0.9
371.8	3.8
393.4	3.9
430.5	0.5
462.6	16.4
531.2	2.9
590.4	4.2
635.8	0.8
681.7	1.0
773.2	2.8
812.5	4.0
858.3	3.8
876.3	16.8
922.1	19.9
935.7	32.5
959.9	20.0
985.4	0.6
1001.5	3.9
1017.4	27.1
1024.9	9.1
1074.9	10.3
1086.4	28.9
1105.2	8.8
1122.1	2.6
1131.2	0.3
1163.3	7.8
1205.2	6.2
1231.0	80.4
1238.0	1.7
1263.7	3.1
1308.4	13.1
1321.8	8.0
1342.2	3.1
1348.8	2.1
1362.6	3.1
1397.0	14.4
1411.8	8.0
1420.6	11.2
1462.4	2.0
1478.6	5.1
1485.0	5.0
1495.7	4.4
1504.9	2.9
1509.0	4.7
1513.0	4.7
1528.4	4.0
3053.8	23.3
3055.5	23.3
3071.6	29.6
3087.5	29.5
3099.1	26.3
3102.0	24.2
3139.6	17.3
3142.3	13.9
3148.9	32.3
3163.9	4.3
3168.3	22.1
3194.9	5.7
3199.7	15.6
3545.7	2.0
3856.4	25.0
